# Copper-Mediated Divergent Reactivity of Allene-Tethered Carbamates under Radical Conditions

**DOI:** 10.1021/acs.orglett.5c01990

**Published:** 2025-06-12

**Authors:** Mireia Toledano-Pinedo, M. Teresa Quirós, Ignacio Padrón, Dawid Halka, Xenios Georgiou, Teresa Martínez del Campo, Amparo Luna, Pedro Almendros

**Affiliations:** † 201430 Instituto de Química Orgánica General, IQOG, CSIC, Juan de la Cierva 3, 28006 Madrid, Spain; ‡ Grupo de Lactamas y Heterociclos Bioactivos, Unidad Asociada al CSIC por el IQOG, Departamento de Química Orgánica, Facultad de Química, 16734 Universidad Complutense de Madrid, 28040 Madrid, Spain; § Departamento de Química Orgánica y Química Inorgánica, Facultad de Farmacia, Universidad de Alcalá, Instituto de Investigación Química Andrés M. del Río (IQAR), 28805 Alcalá de Henares, Madrid, Spain

## Abstract

By judicious selection of reaction conditions, the controllable copper-promoted bromoheterocyclization or the sulfonylation/dimerization of allenyl carbamates to access 5-bromo-6-methylene-1,3-oxazinan-2-ones or bis­(γ-amino vinyl sulfones) has been implemented. The described method, which is easy to scale up and exhibits good functional group tolerance, displays exquisite selectivity control for the preparation of two structurally different compounds starting from the same precursors under free radical conditions.

## Graphical abstract

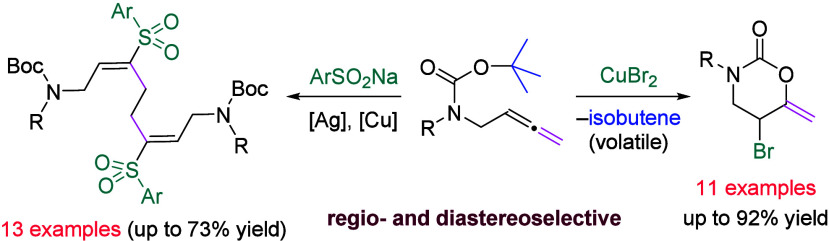

γ-Amino-functionalized vinyl sulfones are a relevant subclass of the important family of vinyl sulfones,^1^ which have promising activities as enzyme inhibitors (Scheme 1, top).^2^ The 1,3-oxazinan-2-one heterocyclic motif is extensively distributed in pharmaceutically active small molecules and natural products, such as the anti-HIV drug efavirenz and the natural product ansacarbamitocin (Scheme 1, top).^3^ Besides, 1,3-oxazinan-2-ones are frequently used as building blocks in organic synthesis.^4^ Consequently, numerous efforts have been devoted to these privileged scaffolds as synthetic targets (Scheme 1a and 1b).^5,6^ The allene (1,2-diene) moiety is a relevant building block in organic synthesis due to its amazing reactivity and its ability to engage as a precursor in the preparation of complex scaffolds.^7^ In this context, the synthesis of *N*-allyl sulfonamides from aminoallenes and sulfinic sodium salts has been recently developed (Scheme 1c),^8^ while we described a cyclization/sulfonylation sequence by a copper-catalyzed reaction between α-allenols and sulfinates (Scheme 1d).^9^ In 2013, we reported the gold-catalyzed cyclization of allenyl carbamates for the synthesis of naked 1,3-oxazinan-2-ones (Scheme 1e).^10^ In continuation of our work on the formation of C–heteroatom bonds from allenes, we report herein both a sulfonylation/dimerization sequence of allenyl carbamates by using aryl sulfinates as well as a CuBr_2_-mediated protocol for the preparation of the bromo-decorated 1,3-oxazinan-2-one nucleus (Scheme 1f).

**Scheme 1:**
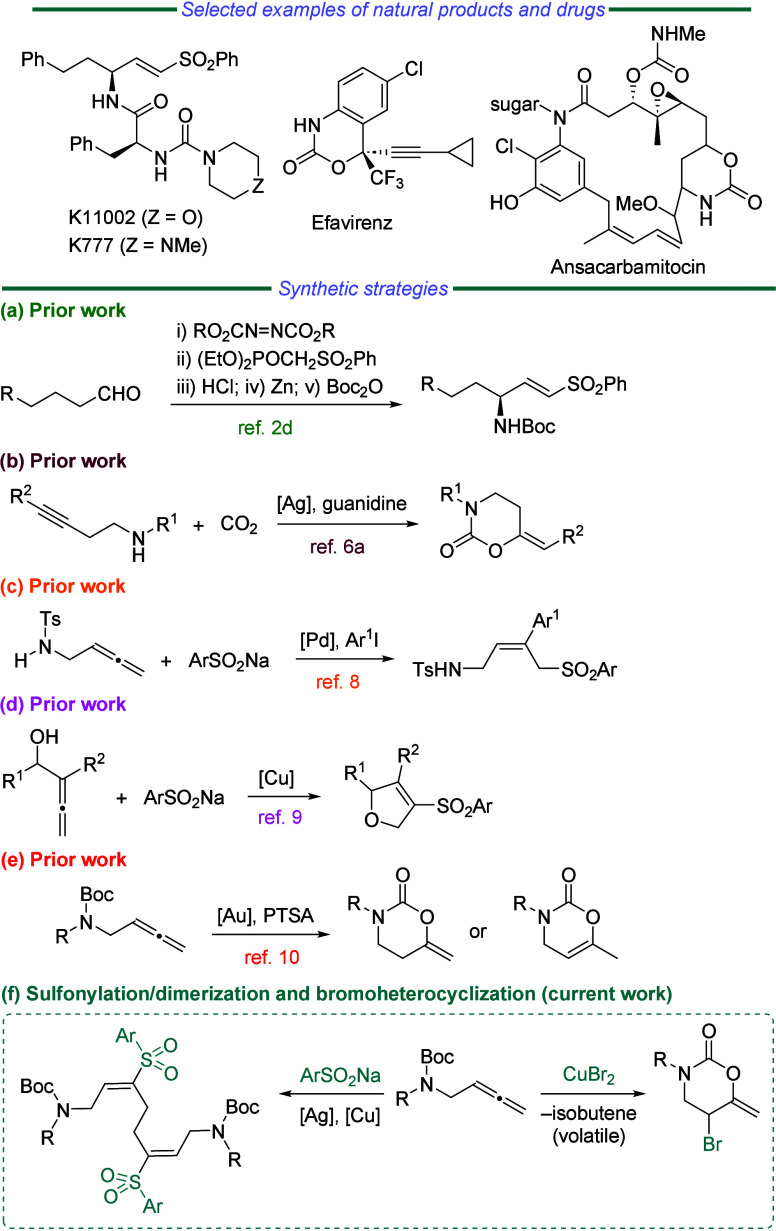
Selected Examples of Drugs and Schematic Representation of Previous Work

Because of the stability, low toxicity, reasonable abundance, and moderate price, the use of copper-based mineral salts in organic synthesis is widespread. Inspired by the recent report on the CuBr_2_/AgF-mediated aminofluorination of dienamides^11^ and our persistent activity in allene chemistry, we were interested in studying the possible intramolecular oxyfluorination of allenyl carbamates. We began our study by testing allenyl carbamate **1a** as a model substrate. Happily, the heterocyclization reaction did take place using the CuBr_2_/AgF-bimetallic system but delivering the 5-bromo-1,3-oxazinan-2-one **2a** (Scheme 2a) rather than the initially expected fluoroderivative. We soon realized that the silver salt AgF was not necessary for the bromo-oxycyclization reaction. The solvent screening revealed that among the different tested solvents, such as CH_3_CN, 1,4-dioxane, CH_2_Cl_2_ and CH_3_NO_2_, nitromethane provided the best performance (92% yield). As the total consumption of the starting material cannot be accomplished at rt, the reaction was conducted at 70 °C. Reducing the amount of the copper salt promoter from 2.5 to 1.5 equiv resulted in a less efficient reaction and the oxazinone **2a** was obtained in a slightly diminished yield of 82% (Table S1, see the Supporting Information).

**Scheme 2:**
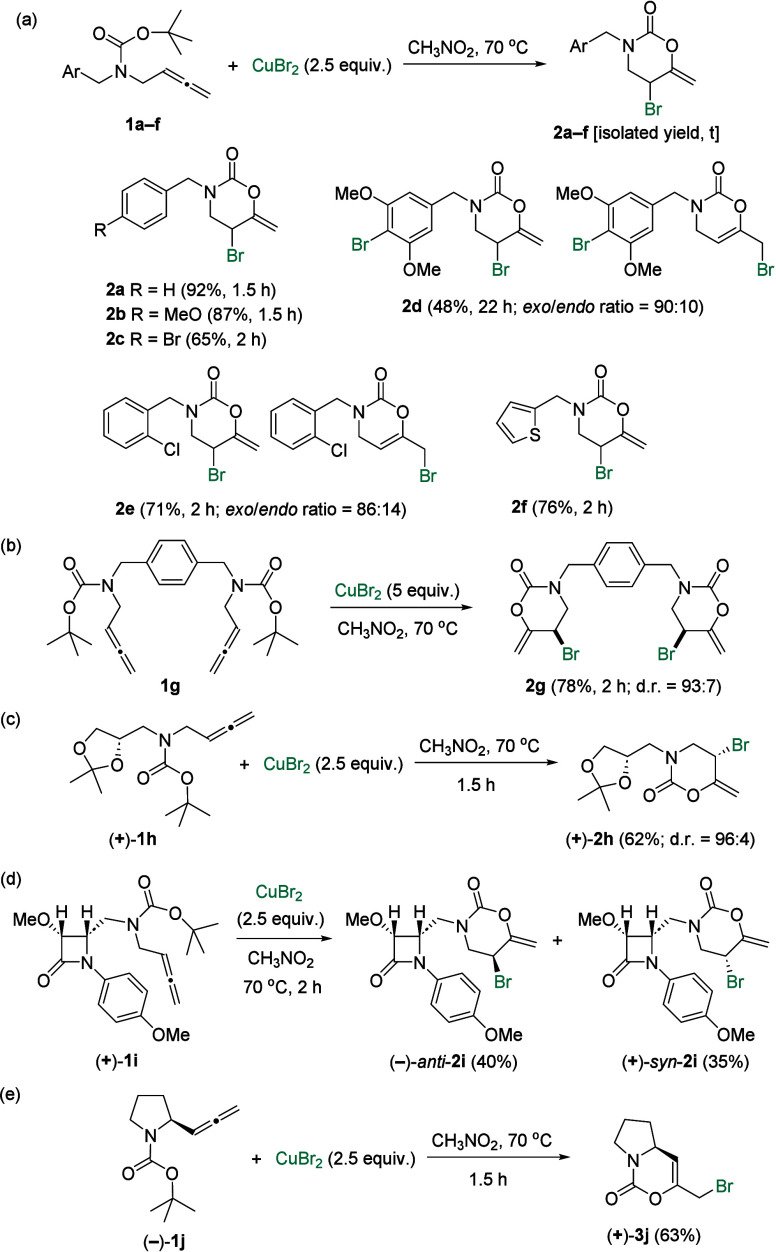
Bromoheterocyclization of Racemic and Enantioenriched Allenes **1**

Taking into account that very few protocols have been conducted for the preparation of the heteroatom-decorated 1,3-oxazinan-2-one nucleus, we were determined to develop a CuBr_2_-mediated synthesis of 5-bromo-1,3-oxazinan-2-ones. The reaction scope was studied through the use of different allenyl carbamates **1**, which were available following the previously reported protocol.^10^ First, benzylic-type *N*-substituted allenes **1a**–**f** were submitted to the CuBr_2_-mediated reaction to provide the corresponding heterocycles **2a**–**f** in good yields (Scheme 2a). The 3,5-dimethoxy and 2-chlorobenzyl derivatives **2d** and **2e** were obtained as a mixture containing 10% and 14% of the endocyclic isomers *endo*-**2d** and *endo*-**2e**, respectively. Surprisingly, a second bromine incorporation occurred in the aromatic nucleus of adduct **2d**.^12^ Interestingly, the presence of a heteroaryl ring such as thiophene in **2f** was well tolerated.

More importantly, the 2-fold reaction of bis­(allene) **1g** smoothly gave rise to bis­(5-bromo-1,3-oxazinan-2-one) **2g** in a reasonable 78% yield with good diastereoselectivity (93:7) in favor of the *syn*-isomer (Scheme 2b). To test the reactivity of the allenyl carbamate functionality toward the asymmetric preparation of 1,3-oxazinan-2-ones, optically active allene (+)-**1h** was chosen in the first place (Scheme 2c). Fortunately, enantioenriched 1,3-oxazinan-2-one (+)-**2h** was obtained in fair yield with excellent diastereoselectivity (96:4). The stereochemistry of the newly formed Br-containing stereocenter was assigned taking into account selected NOE experiments. Because the β-lactam nucleus is relevant from both chemical and pharmacological points of view,^13^ we feel it is interesting to use 2-azetidinone-based allene (+)-**1i** as a cyclization precursor (Scheme 2d). However, the diastereoselectivity of the functionalization/cyclization sequence was not as rewarding as previously. In the event, we obtained a mixture of diastereoisomers, namely, (−)-*anti*-**2i** and (+)-*syn*-**2i** (dr = 53:47), which were epimers at the carbon bearing the halogen atom. Fortunately, the functionalized heterocycles (−)-*anti*-**2i** and (+)-*syn*-**2i** were amenable to separation by flash column chromatography. When the applicability of the protocol was screened in allene (−)-**1j** derived from l-proline, a nonexpected outcome was observed because the fused enantioenriched 2*H*-1,3-oxazin-2-one (+)-**3j** bearing an endocyclic double bond and an exocyclic bromomethyl moiety was achieved as the sole product (Scheme 2e). As depicted in Scheme 2, the asymmetric synthesis of bromofunctionalized 1,3-oxazinan-2-one derivatives is possible but challenging. We feel that *exo*- and *endo*-products **2** are not interconvertible because neither the treatment of oxazinone **2a** (*exo* adduct) with CuBr_2_ in nitromethane at 70 °C formed its isomeric *endo*-**2a**, nor the treatment of oxazinone **2e** (*exo*/*endo* ratio = 90:10) under related conditions resulted in any change in the *exo*/*endo* ratio. Adducts **2**, both *exo*- and *endo*-derivatives, are moderately stable under heating conditions. Oxazinones **2a** and **2e** just decomposed in solutions of toluene or dioxane after heating for several hours at temperatures higher than 200 °C in a sealed tube.

We next explored the putative incorporation of different heteroatoms through the replacement of CuBr_2_ with distinct copper salts. After several experiments using Cu­(OAc)_2_, Cu­(OAc), CuI, Cu­(CF_3_SO_3_)_2_ and CuCl_2_, we figured out that the only productive test was the employment of CuCl_2_. The reaction of allenyl carbamate **1a** with CuCl_2_ instead of CuBr_2_ under otherwise identical reaction conditions resulted in the nonselective formation of 5-chloro-6-methylene-1,3-oxazinan-2-one **2a-Cl** and 6-(chloromethyl)-3,4-dihydro-2*H*-1,3-oxazin-2-one *endo*-**2a-Cl** (isomeric ratio 60:40) (Scheme 3a). As a result of the precedent ability of gold salts to provide 5-alkylidene 2-oxazolidinones from *N*-Boc protected alkynylamines,^14^ we also considered the construction of bromo-functionalized 2-oxazolidinones through our CuBr_2_-mediated protocol. It was noticed that alkynes could be tolerated in the bromoheterocyclization reaction because alkynyl carbamate **alkyne-e** proved amenable to this transformation and provided 5-(bromomethyl)-oxazol-2­(3*H*)-one **4e** in a fair yield with total selectivity (Scheme 3b). To disclose the possible usefulness of our protocol, a gram-scale experiment was run using 5 mmol of allenyl carbamate **1a**. Interestingly, the method is amenable to scale-up because similar figures were attained, and bromo-heterocycle **2a** was formed in 90% yield (Scheme 3c). Previous contributions have reported that heating solutions of CuX_2_ (X = Br, Cl) in organic solvents can either generate molecular X_2_ ^15^ or radical X•.^16^ The treatment of allenyl carbamate **1a** with Br_2_ in nitromethane at 70 °C was derived in a complex reaction mixture. When precursor **1a** was reacted with CuBr_2_ under optimized conditions but with the inclusion of acetanilide as a bromine test, no brominated acetanilide was detected. A radical scavenger test was carried out when **1a** was treated with CuBr_2_ under standard conditions but with the inclusion of TEMPO (Scheme 3d). The formation of cyclic carbamate **2a** was suppressed, which should imply the participation of radicals in the bromoannulation sequence rather than the potential formation of bromine. Besides, 5-bromo-1,3-oxazinan-2-one **2a** was not formed when nonbrominated 1,3-oxazinan-2-one **2a-deBr** was exposed to CuBr_2_ (Scheme 3e), which should discard an initial copper-catalyzed oxycyclization ionic path followed by radical allylic bromination. We carried out the CuBr_2_-mediated reaction of **1b** but with the incorporation of either KI, NaN_3_ or MeOH. In the event, NaN_3_ and MeOH were not effective as trapping agents, and oxazinones **2b**/*endo*-**2b** were achieved from complex reaction mixtures. Interestingly, the addition of KI resulted in the isolation of iodinated product **2b-I** along with **2b**/*endo*-**2b** (Scheme 3f), which proves the existence of an intermediate that could be trapped by I^–^ (see Scheme 4).

**Scheme 3:**
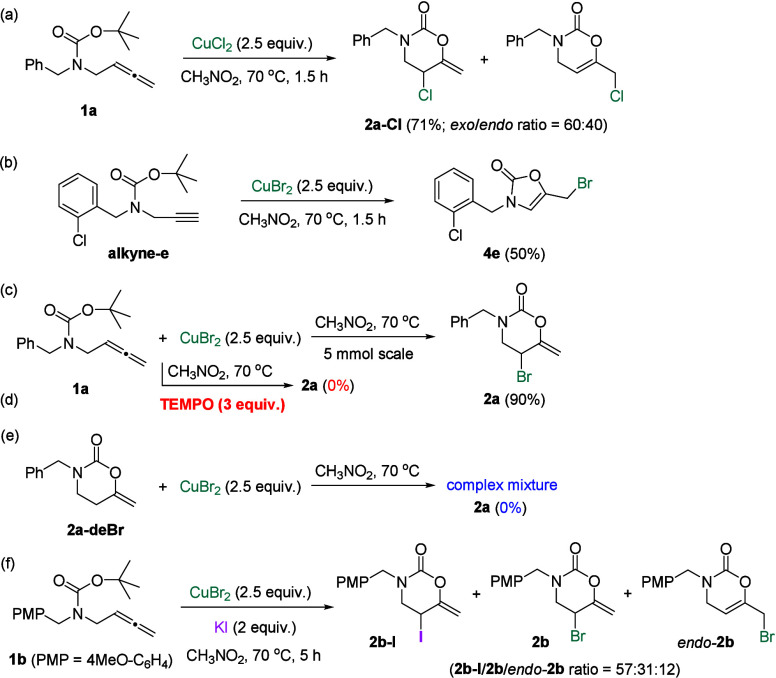
Haloheterocyclization, Scale-up and Control Experiments

**Scheme 4:**
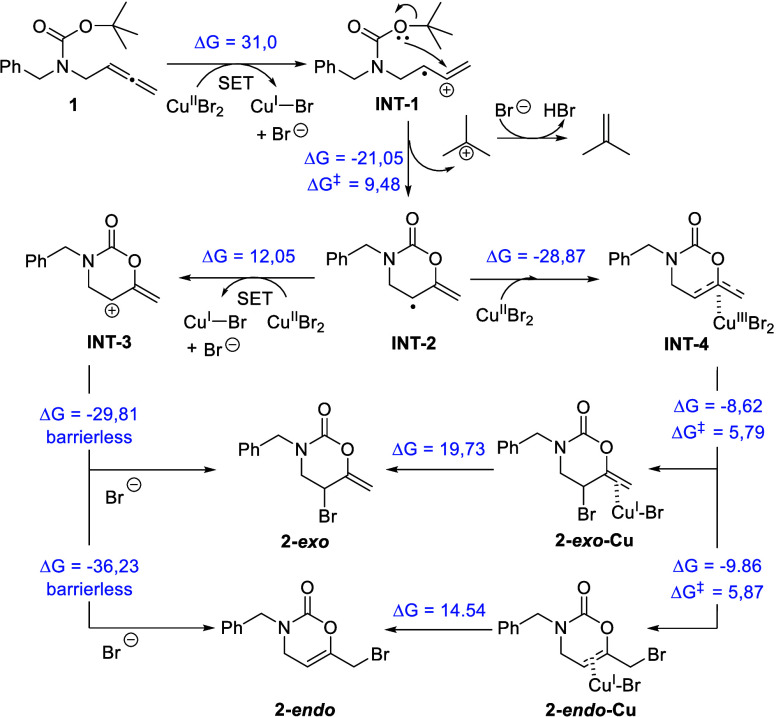
Proposed Reaction Pathway for the CuBr_2_-Promoted Construction of 5-Bromo-1,3-oxazinan-2-ones **2** and DFT Analysis Including Reaction Free Energies and Activation Energies (kcal/mol) Calculated at the M06/6-31G­(d) (C,H,N,O), LANL2DZ (Cu,Br) Level in CH_3_NO_2_ (PCM)

Based on the above control experiments, a reasonable pathway for the formation of bromo-heterocycles **2** has been proposed and computationally studied though DFT calculations using **1a** as the model substrate (Scheme 4). Initially, a single electron transfer (SET) process should take place from the allene moiety of carbamates **1** to CuBr_2._ Although this redox process is endergonic, the fact that the reaction is carried out at 70 °C can explain its occurrence.^17^ In this way, cation radical **INT-1** is built but rapidly evolves through oxycyclization with release of proton and isobutene to form cyclic radicals **INT-2**. This step has an activation energy of 9.5 kcal/mol and is highly exergonic. From this intermediate, two alternative pathways could occur. On the one hand, another SET process from **INT-2** would result in cationic intermediate **INT-3**, which would suffer an electrophilic addition by a bromide anion to form 5-bromo-6-methylene-1,3-oxazinan-2-ones **2**. While this route is energetically achievable under the reaction conditions, it does not explain the preferential formation of the **2**-*exo* product, since the trapping of the cationic intermediate **INT-3** by the Br^–^ seems to occur without activation energy and the **2**-*endo* product is more stable. On the other hand, **INT-2** could recombine with the CuBr_2_ forming allyl-Cu complex **INT-4**, which, after reductive elimination, would form products **2**. This second pathway is energetically more favored, and it would better explain the regioselectivity of the process, with the *exo*-isomer of **2** being kinetically favored (see the Supporting Information for more details). Both mechanisms would explain the formation of the iodinated product **2b-I** after treatment with KI. In the first mechanism trapping of **INT-3** by I^–^ could occur, and in the second, the iodinated product could arise through a Br/I ligand exchange from **INT-4**.

Allenyl carbamate **1b** and 4-chlorobenzenesulfinate **5a** were selected as model substrates to determine the copper-catalyzed viability of the oxycyclization/sulfonylation sequence. We were surprised to find that the combination of Cu­(OAc)_2_ and AgNO_3_ in acetonitrile at 100 °C formed the sulfonylation/self-coupling product **6ba** as a single (*E*,*E*)-isomer in 66% yield. Interestingly, compound **6ba** is a dimeric^18^ γ-amino vinyl sulfone. Taking into account the potential interest of this type of molecules,^19^ we decided to explore the reaction in more depth. Cu­(OAc)_2_ could be safely replaced by CuBr_2_, but the yield of **6ba** dropped to 53%. The use of different oxidants such as TBPB (*tert*-butyl peroxybenzoate) or K_2_S_2_O_8_ instead of AgNO_3_ was also less efficient (for details see Table S2, Supporting Information). Having optimized reaction conditions in hand, the scope of the copper-catalyzed sulfonylation/homodimerization of allenyl carbamates was explored. Allenyl carbamates **1** and sulfinates **5** with electron-donating (MeO, Me) or moderately electron-withdrawing (F, Cl, Br) substituents at different positions of the phenyl ring provided the required homodimeric γ-amino vinyl sulfones **6** in fair yields (Scheme 5). A heterocyclic ring was also well accommodated, providing product **6fa** in similar figures.

**Scheme 5:**
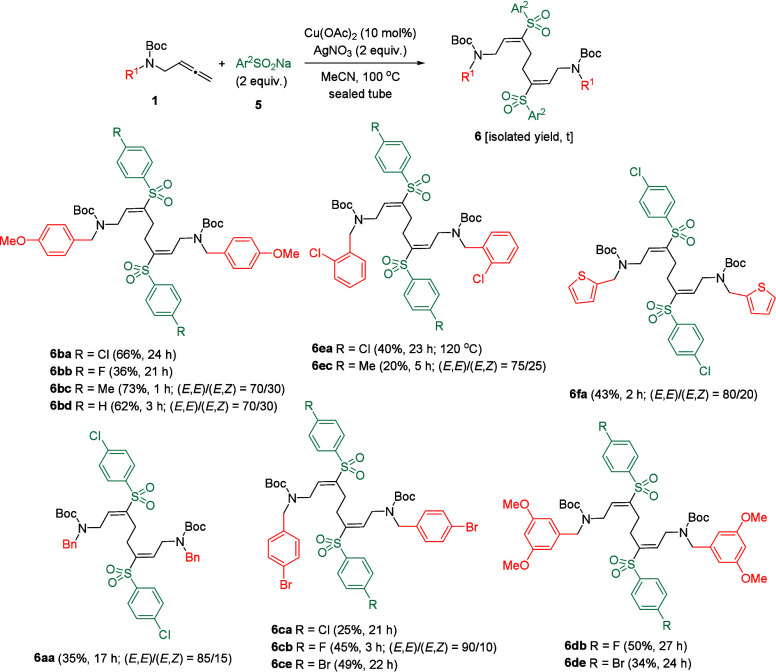
Sulfonylation/Self-Coupling of Allenes **1**

When TEMPO was added as a radical trapping agent to the standard reaction of allenyl carbamate **1b** and 4-chlorobenzenesulfinate **5a**, the formation of **6ba** was suppressed (Scheme 6a), suggesting the involvement of radical species during the process. A competitive reaction of allenyl carbamate **1b**, allenyl carbamate **1d**, and 4-chlorobenzenesulfinate **5a** revealed a complex mixture. Fortunately, the cross-reactions between allenyl carbamate **1b** and allenyl carbamate **1d** with both 4-chlorobenzenesulfinate **5a** and 4-bromobenzenesulfinate **5e** afforded mixed γ-amino vinyl sulfones **7bae** and **7dae** in fair yields (Scheme 6b). This result is informative from a mechanistic point of view and interesting for synthetic purposes. A possible pathway for the generation of bis­(γ-amino vinyl sulfones) **6** from allenes **1** through copper catalysis has been computed using **1a** as the model substrate, and it is delineated in Scheme 6c. First, allenes **1** should coordinate with the metal in a regioselective manner giving rise to π-activated allenes **1-Cu**. Allene-copper complexes **1-Cu** experience radical addition of the sulfur species obtained from sulfinates **5** at the central allene carbon forming intermediates **INT-A**. This step is thermodynamically favored and has a moderate activation energy that is easily achievable at 100 °C. Next, the liberation of the radical from the copper complex should generate radicals **INT-B** and regenerate the copper­(II) catalyst. This step is slightly endergonic and forms free radicals **INT-B**, whose recombination to furnish final products **6** is a highly exergonic and apparently barrierless step (see the Supporting Information for more details).

**Scheme 6:**
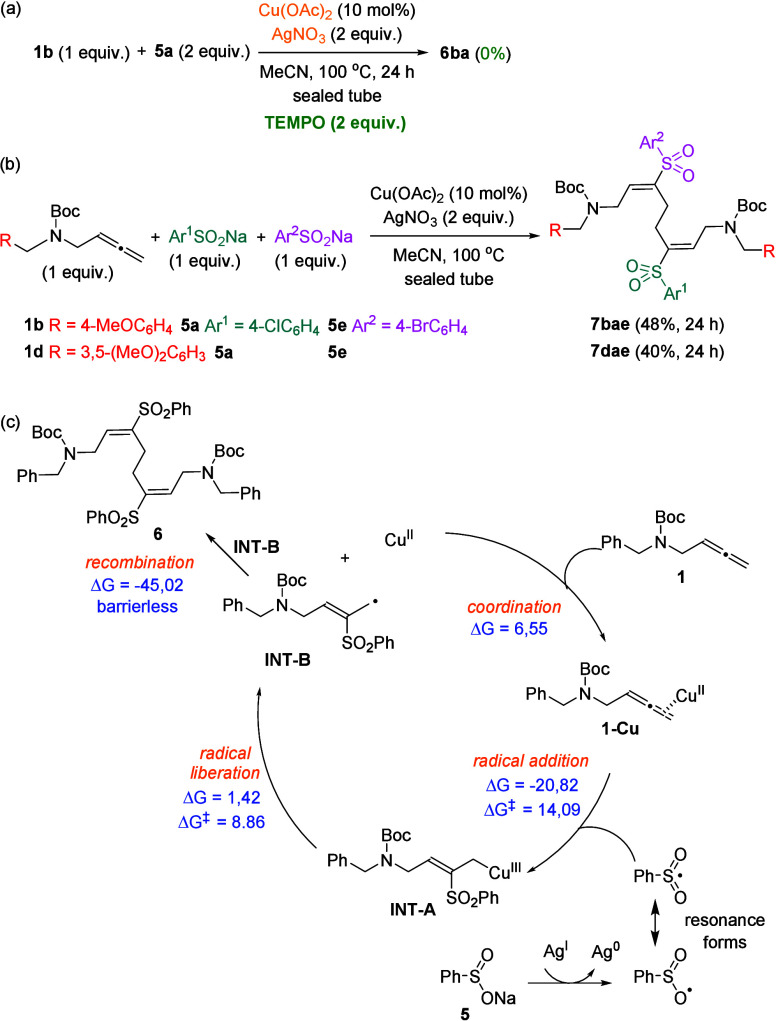
Control Experiments, Synthesis of Mixed γ-Amino Vinyl Sulfones **7**, and Plausible Reaction Pathway Including Reaction Free Energies and Activation Energies (kcal/mol) Calculated at the M06/6-31G­(d) (C,H,N,O,S), LANL2DZ (Cu) Level in MeCN (PCM)

To sum up, despite the multiple reactive sites in allene-tethered carbamate precursors, we were able to control potential competing paths and divert their reactivity toward the formation of 5-bromo-6-methylene-1,3-oxazinan-2-ones and bis­(γ-amino-functionalized vinyl sulfones) in the presence of copper salts under radical conditions.

## Supplementary Material



## Data Availability

The data underlying this study are available in the published article and its Supporting Information.

## Notes

Experimental procedures, characterization data of new compounds, copies of NMR spectra, and computational details (PDF)
