# Supplementary material for: Copper-Mediated Divergent Reactivity of Allene-Tethered Carbamates under Radical Conditions
Source: Org Lett. 2025 Jun 12;27(25):6823–9. doi: 10.1021/acs.orglett.5c01990 (PMC12210270; doi:10.1021/acs.orglett.5c01990)

Supporting Information for the Paper

Copper-Mediated Divergent Reactivity of Allene-Tethered Carbamates Under Radical Conditions

Mireia Toledano-Pinedo,[†,‡] M. Teresa Quirós,[§] Ignacio Padrón,[‡] Dawid Halka,[‡] Xenios Georgiou,[‡] Teresa Martínez del Campo,[*,‡] Amparo Luna,[*,‡] and Pedro Almendros[*,†]

[†]Instituto de Química Orgánica General, IQOG, CSIC, Juan de la Cierva 3, 28006-Madrid, Spain

[‡]Grupo de Lactamas y Heterociclos Bioactivos, Unidad Asociada al CSIC por el IQOG, Departamento de Química Orgánica, Facultad de Química, Universidad Complutense de Madrid, 28040-Madrid, Spain

[§]Departamento de Química Orgánica y Química Inorgánica, Facultad de Farmacia, Universidad de Alcalá, Instituto de Investigación Química Andrés M. del Río (IQAR), 28805-Alcalá de Henares, Madrid, Spain

E-mails: tmcampo@quim.ucm.es; aluna@ quim.ucm.es; palmendros@iqog.csic.es

Table of Contents

1.- General Methods S1

2.- Table S1 and Table S2 S2–S3

3.- Experimental Section S4–S24

4.- NMR Spectra S25–S72

5.- Computational Study S73–S106

1.- General Methods: ^{1}H NMR and ^{13}C NMR spectra were recorded on a Bruker Avance-300 spectrometer. NMR spectra were recorded in $CDCl_3$ or $(CCl_2D)_2$, solutions, except otherwise stated. Chemical shifts are given in ppm relative to TMS (^{1}H, 0.0 ppm), $CDCl_3$ (^{1}H, 7.27 ppm; ^{13}C, 76.9 ppm) and $(CCl_2D)_2$ (^{1}H, 5.91 ppm; ^{13}C, 74.2 ppm). Low- and high-resolution mass spectra were taken on an

AGILENT 6520 Accurate-Mass QTOF LC/MS spectrometer using the electrospray mode (ES) unless otherwise stated. IR spectra were recorded on a Bruker Tensor 27 spectrometer. For reactions that require heating, a heat-on block was used. All commercially available compounds were used without further purification. Structural assignments were made with additional information from gCOSY, gHSQC, and gHMBC experiments.

2.- Table S1 and Table S2

Table S1. Optimization of the Bromoheterocyclization Reaction Conditions of Allene 1a[a]

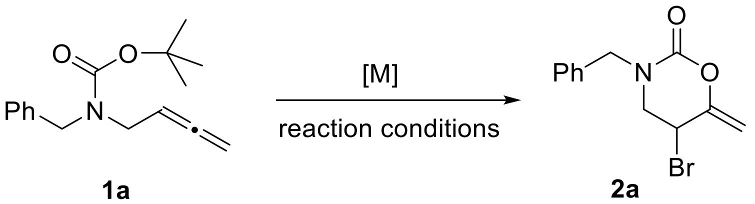

entry	reaction conditions	yield **2a** (%)[c]
1	$CuBr_2$, AgF, CH_3CN, rt, 24 h	60
2	$CuBr_2$, CH_3CN, rt, 24 h	56
3	$CuBr_2$, CH_3CN, 70 ^{0}C, 1.5 h	70
4	$CuBr_2$, CH_3NO_2, 70 ^{0}C, 1.5 h	92
5	$CuBr_2$, CH_2Cl_2, 70 ^{0}C, 1.5 h	40
6	$CuBr_2$, 1,4-dioxane, 70 ^{0}C, 1.5 h	43
7	$CuBr_2$,[b] CH_3NO_2, 70 ^{0}C, 1.5 h	82

[a]Unless otherwise noted, all reactions were carried out using 0.3 mmol of allene **1a** and 0.75 mmol of $CuBr_2$. [b]The reaction was carried out using 0.3 mmol of allene **1a** and 0.45 mmol of $CuBr_2$. [c]Yield of pure, isolated product.

Table S2. Optimization of the Sulfonylation/Dimerization of Allenyl Carbamate 2b and 4-Chlorobenzenesulfinate 5a

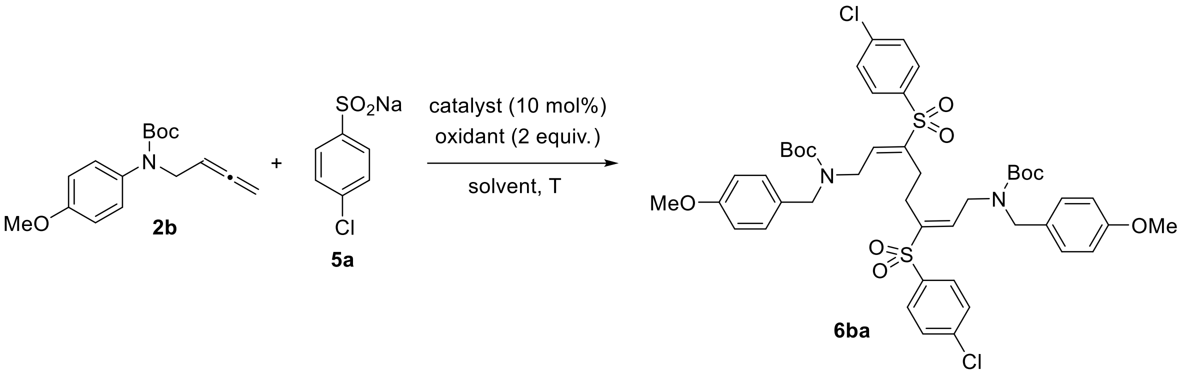

entry	catalyst	oxidant	solvent	T (°C)	time (h)[a]	yield (%)[b]
1	$Cu(OAc)_2$	$AgNO_3$	MeCN	40	40	trace
2	$Cu(OAc)_2$	$AgNO_3$	MeCN	100	24	66
3	$CuBr_2$	$AgNO_3$	MeCN	100	24	53
4	$CuCl_2$	$AgNO_3$	MeCN	100	24	48
5	$Cu(OAc)_2$	Ag_2CO_3	MeCN	100	40	45
6	$Cu(OAc)_2$	AgOAc	MeCN	100	40	40
7	$Cu(OAc)_2$	$AgNO_3$	DMSO	100	24	---
8	$Cu(OAc)_2$	$AgNO_3$	DCE	100	24	trace
9	$Cu(OAc)_2$	$AgNO_3$	1,4-dioxane	100	24	21
10	$Cu(OAc)_2$	TBPB	MeCN	100	24	23
11	$Cu(OAc)_2$	$K_2S_2O_8$	MeCN	100	24	27

[a]Reactions were carried out in a sealed tube using **2b** (0.10 mmol), **5a** (0.20 mmol), oxidant (200 mol%) and catalyst (10 mol%) in the specified solvent (1 mL). Reaction progress was followed by TLC. [b]Yield of pure, isolated product with correct analytical and spectral data

3.- Experimental Section

These precursors were readily obtained as described in the literature: 1a, 1b, 1g, (+)-**1h**, (+)-**1i** and (–)-**1j** (B. Alcaide, P. Almendros, M. T. Quirós and I. Fernández, *Beilstein J. Org. Chem.,* 2013, **9**, 818–826).

Non-brominated 1,3-oxazinan-2-one 2a-deBr was prepared following our previous protocol (*Beilstein J. Org. Chem.,* 2013, **9**, 818–826).

General procedure for the synthesis of *tert*-butyl (prop-2-ynyl)carbamates. A solution of propargylamine (5 mmol) in dichloromethane (5 mL) was added to a well stirred suspension of the corresponding aldehyde (5 mmol) and $MgSO_4$ (40 mmol) in dichloromethane (5 mL) at room temperature until complete conversion (product monitored by TLC). After the solution was filtered and the solvent was evaporated under vacuum, the residue was used for the next step without further purification.

Sodium borohydride (10 mmol) was slowly added to a solution of the appropriate propargylic imine (5 mmol) in methanol (50 mL) at –20 ºC. The reaction mixture was stirred at –20 ºC until disappearance of the starting material (product monitored by TLC). The residue was diluted with acetone (25 mL), filtered through a pad of celite and extracted with dichloromethane (3 x 100 mL). The organic layer, which was washed with brine, dried over $MgSO_4$ and concentrated under reduced pressure was used for next step without further treatment.

A solution of di-*tert*-butyl dicarbonate (5.5 mmol) in dichloromethane (5 mL) was added to a cooled (0 ºC) solution of the appropriate propargylic amine (5 mmol) and triethylamine (5.5 mmol) in dichloromethane (25 mL). The reaction was stirred at room temperature until disappearance of the starting material (product monitored by TLC). Afterwards, the resulting mixture was extracted with dichloromethane (3 x 100 mL), washed with brine, dried over $MgSO_4$ and concentrated under reduced pressure. After the solution was filtered and the solvent was evaporated under vacuum, the residue was purified by flash chromatography on silica gel eluting with ethyl acetate/hexanes mixtures to afford the corresponding *tert*-butyl (prop-2-ynyl)carbamate. Spectroscopic and analytical data for alkynyl carbamate **alkyne-e** follow.

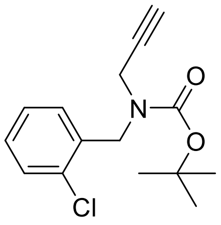

alkyne-e

Alkyne-e. From 500 mg (3.56 mmol) of 2-chlorobenzaldehyde, and after chromatography of the residue using hexanes/ethyl acetate (15:1) as eluent gave compound **alkyne-e** (410 mg, 41%) as a colorless oil; ^{1}H NMR (300 MHz, $CDCl_3$, 25 ºC): δ 7.28 (m, 1H, ArH), 7.15 (m, 3H, ArH), 4.59 (s, 2H, C*H*$_2$-C≡CH), 3.96 (m, 2H, N-CH_2), 2.13 (t, 1H, J = 2.3 Hz, ≡CH), 1.38 (s, 9H, *t*-Bu); ^{13}C{1H} NMR (75 MHz, $CDCl_3$, 25 ºC): δ 155.1, 135.0, 129.6 (Ar, 2CH), 129.0, 128.5 (Ar, CH), 126.8 (Ar, CH), 80.8 (≡CH), 79.2, 77.2, 47.5 (CH_2), 35.9 (CH_2), 28.3 ($3CH_3$); IR ($CHCl_3$): ν 2363 (≡CH), 1701 (C=O) cm^{-1}; HRMS (ESI) *m/z*: $[M + Na]^+$ calcd for $C_{15}H_{18}ClNNaO_2$: 302.0918; found 302.0923.

General procedure for the preparation of allenyl carbamates 1. A well stirred solution of $(CH_2O)_n$ (2 mmol), CuI (0.4 mmol), the appropriate alkyne (0.8 mmol), and *N*,*N*-diisopropylethylamine (Hüning's base) (1.45 mmol) in dioxane (4 mL) was refluxed under argon atmosphere. When the reaction was completed as monitored by TLC, it was cooled to rt. Water (20 mL) was added before being extracted with ethyl acetate (3 x 60 mL). The organic phase was washed with water (2 x 20 mL), dried ($MgSO_4$) and concentrated under reduced pressure. Chromatography of the residue eluting with hexanes/ethyl acetate mixtures gave analytically pure compounds **1**. Spectroscopic and analytical data for allenes **1c-f** follow.

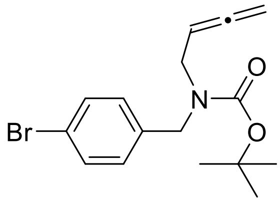

1c

Allenyl carbamate 1c. From 1.24 g (6.7 mmol) of the corresponding alkyne, and after chromatography of the residue using hexanes/ethyl acetate (9:1) as eluent gave compound **1c** (983 mg, 81%) as a pale orange oil; ^{1}H NMR (300 MHz, $CDCl_3$, 25 ºC): δ 7.44 (d, 2H, J = 8.7 Hz, ArH),

7.12 (d, 2H, J = 8.7 Hz, ArH), 5.07(s ,1H, CH), 4.75 (dt, 2H, J = 6.6, 2.8 Hz, =CH_2), 4.38 (s, 2H, CH_2-CH), 3.78 (m, 2H, NCH_2), 1.47 (s, 9H, *t*-Bu); $^{13}C\{1H\}$ NMR (75 MHz, $CDCl_3$, 25 ºC): δ 209.0, 155.5, 137.3, 131.5 (Ar, 2CH), 129.6 (Ar, CH), 129.1 (Ar, CH),, 120.9, 86.7, 80.1, 76.3 (CH_2), 49.0 (CH_2), 45.1 (CH_2), 28.3 ($3CH_3$); IR ($CHCl_3$): ν 1955 (=·=), 1692 (C=O) cm^{-1}; HRMS (ESI) *m/z*: $[M + Na]^+$ calcd for $C_{16}H_{20}BrNNaO_2$: 360.0570; found 360.0569.

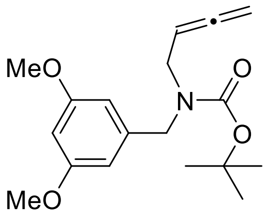

1d

Allenyl carbamate 1d. From 1.46 g (5 mmol) of the corresponding alkyne, and after chromatography of the residue using hexanes/ethyl acetate (9:1) as eluent gave compound **1d** (841 mg, 58%) as a colorless oil; ^1H NMR (300 MHz, $CDCl_3$, 25 ºC): δ 6.37 (m, 3H, ArH), 5.10 (s, 1H, CH), 4.77 (m, 2H, =CH_2), 4.37 (s, 2H, CH_2-CH), 3.84 (m, 2H, NCH_2), 3.77 (s, 6H, O-CH_3), 1.48 (s, 9H, *t*-Bu); $^{13}C\{1H\}$ NMR (75 MHz, $CDCl_3$, 25 ºC): δ 209.1, 160.8 (2C), 140.6, 133.6, 105.8 (Ar, CH), 99.0 (Ar, 2CH), 86.8, 79.8, 76.1 (CH_2), 55.2 ($2CH_3$-O), 49.4 (CH_2), 44.8 (CH_2), 28.4 ($3CH_3$); IR ($CHCl_3$): ν 1956 (=·=), 1690 (C=O) cm^{-1}; HRMS (ESI) *m/z*: $[M + Na]^+$ calcd for $C_{18}H_{25}NNaO_4$: 342.1676; found 342.1676.

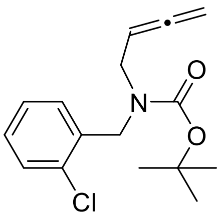

1e

Allenyl carbamate 1e. From 190 mg (0.68 mmol) of the corresponding alkyne, and after chromatography of the residue using hexanes/ethyl acetate (15:1) as eluent gave compound **1e** (176 mg, 88%) as a colorless oil; ^1H NMR (300 MHz, $CDCl_3$, 25 ºC): δ 7.26 (m, 1H, ArH), 7.13 (m, 3H, ArH), 5.04 (s, 1H, CH), 4.67 (dt, 2H, J = 6.4, 2.7 Hz, =CH_2), 4.48 (m, 2H, CH_2-CH), 3.75 (m, 2H, NCH_2), 1.37 (s, 9H, *t*-Bu); $^{13}C\{1H\}$ NMR (75 MHz, $CDCl_3$, 25 ºC): δ 208.9, 155.5, 135.6, 129.4 (Ar, 2CH), 128.9, 128.2 (Ar, CH), 126.8 (Ar, CH), 86.8 (CH), 80.0, 76.3 (CH_2), 47.1 (CH_2), 45.6 (CH_2), 28.3 ($3CH_3$); IR ($CHCl_3$): ν 1955 (=·=), 1699 (C=O) cm^{-1}; HRMS (ESI) *m/z*: $[M + Na]^+$ calcd for $C_{16}H_{20}ClNNaO_2$: 316.1075; found 316.1077.

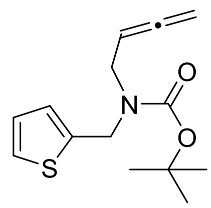

1f

Allenyl carbamate 1f. From 500 mg (1.99 mmol) of the corresponding alkyne, and after chromatography of the residue using hexanes/ethyl acetate (9:1) as eluent gave compound **1f** (366 mg, 69%) as a colorless oil; ^{1}H NMR (300 MHz, $CDCl_3$, 25 ºC): δ 7.21 (m, 1H, ArH), 6.92 (m, 2H, ArH), 5.09 (s, 1H, CH), 4.78 (m, 2H, $=CH_2$), 4.56 (s, 2H, C*H*$_2$-CH), 3.86 (s, 2H, NCH_2), 1.50 (s, 9H, *t*-Bu); ^{13}C{1H} NMR (75 MHz, $CDCl_3$, 25 ºC): δ 209.2, 154.7, 140.9, 126.3 (Ar, CH), 126.1 (Ar, CH), 125.1 (Ar, CH), 86.8 (CH), 80.1, 76.1 (CH_2), 67.0 (CH_2), 44.3 (CH_2), 28.3 ($3CH_3$); IR ($CHCl_3$): ν 1952 (=·=), 1699 (C=O) cm^{-1}; HRMS (ESI) *m/z*: $[M + Na]^+$ calcd for $C_{14}H_{19}NNaO_2S$: 288.1029; found 288.1038.

General procedure for the bromoheterocyclization of racemic and enantioenriched allenes 1. $CuBr_2$ or $CuCl_2$ (2.5 mmol or 5 mmol for **1g**) was added under argon atmosphere to a solution of the appropriate allenyl or alkynyl carbamate **1** or **alkyne-e** (1.0 mmol) in nitromethane (5 mL). The reaction was stirred at 70 ºC until disappearance of the starting material (TLC), and then the mixture was concentrated under reduced pressure. Chromatography of the residue eluting with hexanes/ethyl acetate mixtures gave analytically pure compounds. Spectroscopic and analytical data for heterocycles **2, 3** and **4c** follow.

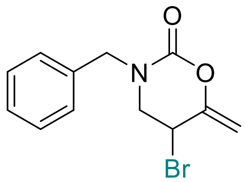

2a

5-Bromo-1,3-oxazinan-2-one 2a. From 25 mg (0.10 mmol) of allene **1a**, and after chromatography of the residue using hexanes/ethyl acetate (8:2) as eluent gave compound **2a** (25 mg, 92%) as a colorless solid; mp 74–76 ºC; ^{1}H NMR (300 MHz, $CDCl_3$, 25 ºC): δ 7.26 (m, 5H, ArH), 6.03 (dd, 1H, $J = 2.3$, 1.1 Hz, =C*H*H), 5.59 (d, 1H, $J = 5.6$ Hz, =CH*H*), 4.89 (dd, 1H, $J = 9.0$, 6.2 Hz, Br-CH), 4.38 (q, 2H, $J = 14.9$ Hz, N-CH_2), 3.51 (t, 1H, $J = 9.1$ Hz, N-C*H*H-CH), 3.25 (dd, 1H, J =9.0, 6.1 Hz, N-CH*H*-CH); ^{13}C{1H} NMR (75 MHz, $CDCl_3$, 25 ºC): δ 156.9, 135.2, 129.0, 128.9 (Ar, 2CH), 128.1

(Ar, 3CH), 118.8 (CH_2), 74.6 (CH), 48.7 (CH_2), 48.3 (CH_2); IR ($CHCl_3$): ν 1759 (C=O), 1219 cm^{-1}; HRMS (ESI) *m/z*: $[M + H]^+$ calcd for $C_{12}H_{13}BrNO_2$: 284.0105; found 284.0096.

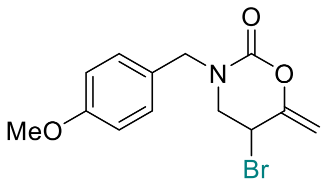

2b

5-Bromo-1,3-oxazinan-2-one 2b. From 50 mg (0.17 mmol) of allene **1b**, and after chromatography of the residue using hexanes/ethyl acetate (8:2) as eluent gave compound **2b** (45 mg, 87%) as a colorless solid; mp 89–91 ºC; ^{1}H NMR (300 MHz, $CDCl_3$, 25 ºC): δ 7.20 (d, 2H, *J* = 8.6 Hz, ArH), 6.88 (d, 2H, *J* = 8.6 Hz, ArH), 6.10 (dd, 1H, *J* = 2.4, 1.2 Hz, =C*H*H), 5.66 (dd, 1H, *J* = 2.4, 0.7 Hz, =CH*H*), 4.95 (dd, 1H, *J* = 9.0, 6.2 Hz, Br-CH), 4.39 (q, 2H, *J* = 14.7 Hz, N-CH_2), 3.81 (s, 3H, O-CH_3), 3.57 (t, 1H, *J* = 9.1 Hz, N-C*H*H-CH), 3.30 (dd, 1H, *J* = 9.0, 6.1 Hz, N-CH*H*-CH); ^{13}C{1H} NMR (75 MHz, $CDCl_3$, 25 ºC): δ 159.4, 156.9, 129.5 (Ar, 2CH), 129.1, 127.2, 118.7 (CH_2), 114.2 (Ar, 2CH), 74.6 (CH), 55.3 (CH_3-O), 48.5 (CH_2), 47.7 (CH_2); IR ($CHCl_3$): ν 1757 (C=O), 1248, 1035 cm^{-1}; HRMS (ESI) *m/z*: $[M + Na]^+$ calcd for $C_{13}H_{14}BrNNaO_3$: 334.0049; found 334.0055.

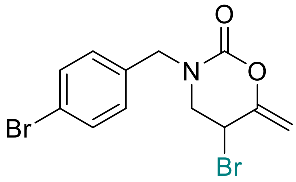

2c

5-Bromo-1,3-oxazinan-2-one 2c. From 110 mg (0.32 mmol) of allene **1c**, and after chromatography of the residue using hexanes/ethyl acetate (3:1) as eluent gave compound **2c** (75 mg, 65%) as a white solid; mp 120–122 ºC; ^{1}H NMR (300 MHz, $CDCl_3$, 25 ºC): δ 7.49 (d, 2H, *J* = 8.5 Hz, ArH), 7.16 (d, 2H, *J* = 8.5 Hz, ArH), 6.10 (dd, 1H, *J* = 2.4, 1.2 Hz, =C*H*H), 5.67 (d, 1H, *J* = 5.6 Hz, =CH*H*), 4.96 (ddt, 1H, *J* = 9.0, 6.0, 0.9 Hz, Br-CH), 4.40 (q, 2H, *J* = 15.1 Hz, N-CH_2), 3.59 (t, 1H, *J* = 9.0 Hz, N-C*H*H-CH), 3.31 (dd, 1H, *J* =9.0, 6.0 Hz, N-CH*H*-CH); ^{13}C{1H} NMR (75 MHz, $CDCl_3$, 25 ºC): δ 156.8, 134.2, 132.0 (Ar, 2CH), 129.7 (Ar, 2CH), 128.8, 122.1, 119.0 (CH_2), 74.6 (CH), 48.5 (CH_2), 47.6 (CH_2); IR ($CHCl_3$): ν 1734 (C=O), 1260 cm^{-1}; HRMS (ESI) *m/z*: $[M + H + 2]^+$ calcd for $C_{12}H_{12}Br_2NO_2$: 361.9209; found 361.9211.

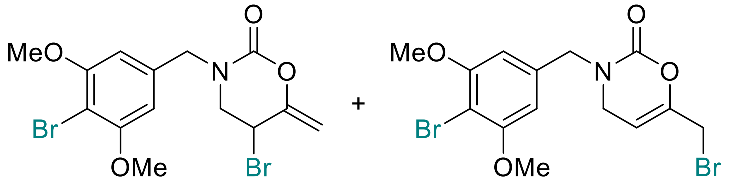

2d (*exo*/*endo* ratio = 90:10)

5-Bromo-1,3-oxazinan-2-one 2d. From 55 mg (0.17 mmol) of allene **1d**, and after chromatography of the residue using hexanes/ethyl acetate (4:1) as eluent gave compound **2d** (35 mg, 48%; *exo/endo* ratio = 90:10) as a pale orange oil; ^{1}H NMR (300 MHz, $CDCl_3$, 25 °C): δ 6.59 (d, 1H, *J* = 2.8 Hz, ArH, m), 6.52 (d, 1H, *J* = 2.8 Hz, ArH, M), 6.45 (d, 2H, *J* = 2.8 Hz, ArH, M + m), 6.11 (dd, 1H, *J* = 2.4, 1.2 Hz, =C*H*H, M), 5.68 (dd, 1H, *J* = 2.4, 1.2 Hz, =CH*H*, M), 5.25 (t, 1H, *J* = 3.4 Hz, Br-CH, m), 4.98 (m, 1H, Br-CH, M), 4.75 (s, 2H, N-CH_2, m), 4.59 (s, 2H, N-CH_2, M), 3.88 (s, 6H, O-CH_3, M + m), 3.82 (m, 2H, CH_2-Br, m), 3.81 (s, 3H, O-CH_3, m), 3.79 (s, 3H, O-CH_3, M), 3.66 (t, 3H, *J* = 9.1 Hz, N-C*H*H-CH, M + N-CH_2-CH, m), 3.42 (dd, 1H, *J* =9.1, 6.0 Hz, N-CH*H*-CH, M); ^{13}C{1H} NMR (75 MHz, $CDCl_3$, 25 °C): δ 160.1 (m), 160.0 (M), 156.9 (M + m), 156.8 (M), 156.7 (m), 136.4 (M + m), 129.0 (M + m), 118.9 (CH_2, M), 105.6 (Ar, CH, M), 105.3 (Ar, CH, m), 104.1 (M + m), 99.5 (Ar, CH, M), 99.2 (CH, m), 98.9 (Ar, CH, m) 74.7 (CH, M), 56.3 (CH_3-O, M + m), 55.6 (CH_3-O, M + m), 51.8 (CH_2, m), 49.0 (CH_2, M), 48.2 (CH_2, M), 45.1 (CH_2, m), 29.6 (CH_2, m); IR ($CHCl_3$): ν 1752 (C=O), 1160 cm^{-1}; HRMS (ESI) *m/z*: [*M* + Na]$^+$ calcd for $C_{14}H_{15}Br_2NNaO_4$: 441.9260; found 441.9263.

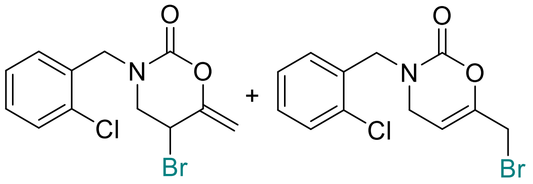

2e (*exo*/*endo* ratio = 86:14)

5-Bromo-6-methylene-1,3-oxazinan-2-one 2e. From 100 mg (0.34 mmol) of allene **1e**, and after chromatography of the residue using hexanes/ethyl acetate (8:2) as eluent gave compound **2e** (77 mg, 71%; *exo/endo* ratio = 86:14) as a yellow oil; ^{1}H NMR (300 MHz, $CDCl_3$, 25 °C): δ 7.37 (m, 4H, ArH, M + m), 7.26 (m, 4H, ArH, M + m), 6.09 (dd, 1H, *J* = 2.4, 1.2 Hz, =C*H*H, M), 5.66 (dd, 1H, *J* = 2.4, 0.6 Hz, =CH*H*, M), 5.24 (t, 1H, *J* = 3.4 Hz, Br-CH, m), 4.96 (m, 1H, Br-CH, M), 4.73 (s, 2H, N-CH_2, m), 4.58 (q, 2H, *J* = 15.2 Hz, N-CH_2, M), 3.86 (s, 2H, CH_2-Br, m), 3.80 (m, 2H, N-CH_2-CH, m), 3.64 (t, 1H, *J* = 9.1 Hz, N-C*H*H-CH, M), 3.38 (dd, 1H, *J* = 9.1, 6.1 Hz, N-CH*H*-CH, M); ^{13}C{1H} NMR (75 MHz, $CDCl_3$, 25 °C): δ 156.9 (M + m), 133.9 (m), 133.8 (M), 132.9 (M), 132.7 (m), 130.2

(Ar, CH, M), 130.0 (m), 129.8 (Ar, CH, M), 129.7 (Ar, CH, m), 129.5 (Ar, 2CH, M + m), 129.3 (Ar, CH, m), 129.0 (M), 127.4 (Ar, CH, m), 127.4 (Ar, CH, M), 119.0 (CH_2, M), 98.9 (CH, m), 74.7 (CH, M), 49.2 (CH_2, m), 49.0 (CH_2, M), 45.5 (CH_2, M), 45.2 (CH_2, m), 27.3 (CH_2, m); IR ($CHCl_3$): ν 1760 (C=O), 1257 cm^{-1}; HRMS (ESI) *m/z*: $[M + Na]^+$ calcd for $C_{12}H_{11}BrClNNaO_2$: 339.9532; found 339.9523.

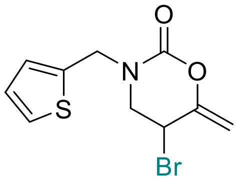

2f

5-Bromo-6-methylene-1,3-oxazinan-2-one 2f. From 50 mg (0.20 mmol) of allene **1f**, and after chromatography of the residue using hexanes/ethyl acetate (6:4) as eluent gave compound **2f** (44 mg, 76%) as a yellow oil; ^1H NMR (300 MHz, $CDCl_3$, 25 ºC): δ 7.29 (dd, 1H, *J* =5.0, 1.4 Hz, ArH), 7.01 (m, 2H, ArH), 6.10 (dd, 1H, *J* = 2.4 Hz, 1.2 Hz, =C*H*H), 5.68 (dd, 1H, *J* = 2.4 Hz, 0.7 Hz, =CH*H*), 4.97 (m, 1H, Br-CH), 4.64 (qd, 2H, *J* = 15.5 Hz, 0.5 Hz, N-CH_2), 3.68 (t, 1H, *J* = 9.0 Hz, N-C*H*H-CH), 3.41 (dd, 1H, *J* = 8.9, 6.1 Hz, N-CH*H*-CH); $^{13}C\{1H\}$ NMR (75 MHz, $CDCl_3$, 25 ºC): δ 156.5, 137.4, 128.8, 127.3 (Ar, CH), 127.1 (Ar, CH), 126.1 (Ar, CH), 118.9 (CH_2), 74.7 (CH), 48.5 (CH_2), 43.7 (CH_2); IR ($CHCl_3$): ν 1757 (C=O), 1255 cm^{-1}; HRMS (ESI) *m/z*: $[M + H]^+$ calcd for $C_{10}H_{11}BrNO_2S$: 289.9668; found: 289.9679.

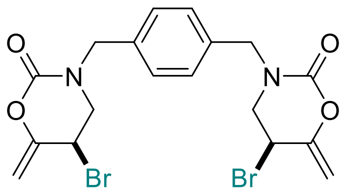

2g (d.r. = 93:7)

Bis(5-bromo-6-methylene-1,3-oxazinan-2-one) 2g. From 25 mg (0.06 mmol) of allene **1g**, and after chromatography of the residue using hexanes/ethyl acetate (1:1) as eluent gave compound **2g** (22 mg, 78%; d.r. = 93:7) as a colorless solid; mp 143–145 ºC; ^1H NMR (300 MHz, $CDCl_3$, 25 ºC): δ 7.29 (s, 4H, ArH), 6.12 (dd, 2H, *J* =2.3, 1.1 Hz, 2C*H*H), 5.69 (dd, 2H, *J* = 2.4, 0.6 Hz, 2CH*H*), 4.98 (dd, 2H, *J* = 9.1, 6.1 Hz, 2Br-CH), 4.45 (m, 4H, 2N-CH_2), 3.61 (t, 2H, *J* = 9.0 Hz, 2N-C*H*H-CH), 3.34 (ddd, 2H, *J* = 9.0, 6.1, 0.6 Hz, 2N-CH*H*-CH); $^{13}C\{1H\}$ NMR (75 MHz, $CDCl_3$, 25 ºC): δ 157.0, 135.3, 128.9, 128.6 (Ar, 4CH), 119.0 (2CH_2), 74.6 (2CH), 48.7 (2CH_2), 47.9 (2CH_2); IR ($CHCl_3$): ν 1753

(C=O), 1215 cm^{-1}; HRMS (ESI) *m/z*: $[M + NH_4]^+$ calcd for $C_{18}H_{22}Br_2N_3O_4$: 503.9952; found 503.9971.

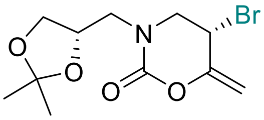

(+)-**2h** (d.r. = 96:4)

5-Bromo-6-methylene-1,3-oxazinan-2-one (+)-2h. From 140 mg (0.5 mmol) of allene (+)-**1h**, and after chromatography of the residue using hexanes/ethyl acetate (8:2) as eluent gave compound (+)-**2h** (95 mg, 62%; d.r. = 96:4) as a yellow oil; $[\alpha]_D$ = +57.3 (*c* 0.8, $CHCl_3$); ^{1}H NMR (300 MHz, $CDCl_3$, 25 °C): δ 6.11 (dd, 1H, *J* = 2.39, 1.16 Hz, =CH*H*), 5.70 (m, 1H, =C*H*H), 5.00 (m, 1H, Br-CH), 3.94 (m, 2H, OCH_2), 3.68 (m, 1H, CH), 3.43 (m, 2H, CH_2), 3.31 (m, 2H, CH_2), 1.20 (s, 6H, $2CH_3$); ^{13}C{1H} NMR (75 MHz, $CDCl_3$, 25 °C): δ 156.2 (m), 156.1 (M), 128.0 (M), 127.9 (m), 117.7 (CH_2, m), 117.5 (CH_2, M), 73.6 (CH, M + m), 72.2 (CH, M + m), 68.7 (m), 68.4 (M), 61.9 (CH_2, m), 61.8 (CH_2, M), 49.8 (CH_2, M), 49.5 (CH_2, m), 45.9 (CH_2, M + m), 26.2 ($2CH_3$, M + m); IR ($CHCl_3$): ν 1750 (C=O), 1261 cm^{-1}; HRMS (ESI) *m/z*: $[M + Na]^+$ calcd for $C_{11}H_{16}BrNNaO_4$: 328.0155; found 328.0162.

5-Bromo-6-methylene-1,3-oxazinan-2-ones (–)-*anti*-2i and (+)-*syn*-2i. From 100 mg (0.26 mmol) of allene (+)-**1i**, a mixture of two epimeric 1,3-oxazinan-2-ones **2i** in a ratio (53:47) was formed. After chromatography of the residue using hexanes/ethyl acetate (1:1) as eluent, a less polar compound (–)-*anti*-**2i** (42 mg, 40%) and a more polar compound (+)-*syn*-**2i** (37 mg, 35%) were obtained.

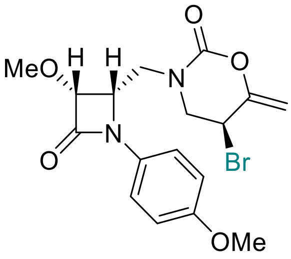

(–)-*anti*-**2i**

5-Bromo-6-methylene-1,3-oxazinan-2-one (–)-*anti*-2i. Colorless solid; mp 190–192 ºC; $[\alpha]_D$ = –75.4 (*c* 0.6, $CHCl_3$); ^{1}H NMR (300 MHz, $CDCl_3$, 25 °C): δ 7.44 (d, 2H, *J* = 9.0 Hz, ArH), 6.91 (d, 2H, *J* = 9.0 Hz, ArH), 6.09 (dd, 1H, *J* = 2.4, 0.9 Hz, =CH*H*), 5.70 (d, 1H, *J* = 2.3 Hz, =C*H*H), 4.89

(dd, 1H, J = 8.4, 6.3 Hz, CH-Br), 4.61 (m, 1H, O-CH), 4.47 (ddd, 1H, J = 8.9, 5.0, 4.0 Hz, N-CH), 3.88 (dt, 1H, J = 8.9, 5.0, 4.0 Hz, N-CH-C*H*H-N), 3.80 (s, 3H, O-CH_3), 3.68 (s, 3H, O-CH_3), 3.60 (m, 2H, CH_2), 3.45 (dd, 1H, J = 14.5, 8.1 Hz, N-CH-CH*H*-N); $^{13}C\{1H\}$ NMR (75 MHz, $CDCl_3$, 25 ºC): δ 164.1, 157.1, 156.6, 129.9, 129.2, 119.5 (CH_2), 118.6 (Ar, 2CH), 114.6 (Ar, 2CH), 82.3(CH), 75.1 (CH-Br), 59.2 (CH_3-O), 55.5 (CH_3-O), 55.1 (N-CH), 50.5 (*C*H_2), 41.3 (CH_2); IR ($CHCl_3$): ν 1749 (C=O), 1735 (C=O), 1248 cm^{-1}; HRMS (ESI) *m/z*: $[M + Na]^+$ calcd for $C_{17}H_{19}BrN_2NaO_5$: 435.0351; found 435.0347.

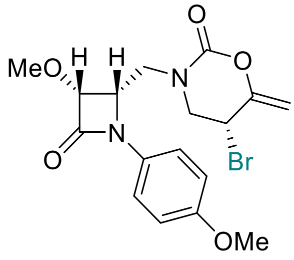

(+)-*syn*-**2i**

5-Bromo-6-methylene-1,3-oxazinan-2-one (+)-*syn*-2i. Colorless solid; mp 191–193 ºC; $[\alpha]_D$ = +16.9 (c 0.8, $CHCl_3$); ^1H NMR (300 MHz, $CDCl_3$, 25 ºC): δ 7.44 (m, 2H, Ar), 6.91 (m, 2H, Ar), 6.07 (d, 1H, J = 2.0 Hz, =C*H*H), 5.67 (d, 1H, J = 2.3 Hz, =CH*H*), 4.98 (m, 1H, CH-Br), 4.63 (m, 1H, O-CH), 4.45 (m, 1H, N-CH), 3.91 (m, 2H, N-CH-C*H*H-N + N-C*H*H-CHBr), 3.79 (s, 3H, O-CH_3), 3.70 (s, 3H, O-CH_3), 3.43 (m, 2H, N-CH-CH*H*-N + N-CH*H*-CHBr); $^{13}C\{1H\}$ NMR (75 MHz, $CDCl_3$, 25 ºC): δ 163.1, 157.3, 156.6, 129.9, 128.6, 118.9 (CH_2), 118.7 (Ar, 2CH), 114.7 (Ar, 2CH), 82.2 (CH), 75.1 (CH-Br), 59.1 (CH_3-O), 55.5 (CH_3-O), 55.2 (N-CH), 50.8 (*C*H_2), 41.4 (CH_2); IR ($CHCl_3$): ν 1750 (C=O), 1737 (C=O), 1249 cm^{-1}; HRMS (ESI) *m/z*: $[M + Na]^+$ calcd for $C_{17}H_{19}BrN_2NaO_5$: 435.0351; found 435.0361.

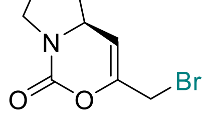

(+)-**3j**

2*H*-1,3-oxazin-2-one (+)-3j. From 100 mg (0.48 mmol) of allene (–)-**1j**, and after chromatography of the residue using hexanes/ethyl acetate (1:1) as eluent gave compound (+)-**3j** (70 mg, 63%) as a colorless oil; $[\alpha]_D$ = +10.3 (c 0.2, $CHCl_3$); ^1H NMR (300 MHz, $CDCl_3$, 25 ºC): δ 5.39 (d, 1H, J = 1.7 Hz, =CH), 4.11 (m, 1H, N-CH), 3.89 (s, 2H, CH_2-Br), 3.73 (m, 1H, N-C*H*H), 3.48 (m, 1H, N-CH*H*), 2.17 (m, 1H, NCH_2-C*H*H), 2.02 (m, 1H, NCH_2-CH*H*), 1.88 (m, 1H, NCH-C*H*H), 1.62 (m, 1H, NCH-

CH*H*); $^{13}C\{1H\}$ NMR (75 MHz, $CDCl_3$, 25 ºC): δ 148.8, 147.2, 102.7 (=CH), 56.1 (CH), 45.7 (CH_2), 32.7 (CH_2), 27.4 (CH_2), 21.9 (CH_2); IR ($CHCl_3$): ν 1726 (C=O), 1216 cm^{-1}; HRMS (ESI) *m/z*: [*M* + H]$^+$ calcd for $C_8H_{11}BrNO_2$: 231.9968; found 231.9965.

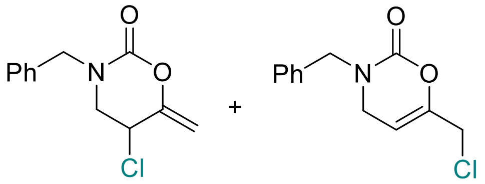

2a-Cl (*exo*/*endo* ratio = 60:40)

5-Chloro-6-methylene-1,3-oxazinan-2-one 2a-Cl. From 20 mg (0.08 mmol) of allene **1a**, and after chromatography of the residue using hexanes/ethyl acetate (8:2) as eluent gave compound **2a-Cl** (13 mg, 71%; *exo/endo* ratio = 60:40) as a yellow oil; ^1H NMR (300 MHz, $CDCl_3$, 25 ºC): δ 7.33 (m, 10H, ArH, M + m), 5.67 (dd, 1H, *J* = 2.0, 0.9 Hz, =C*H*H, M), 5.44 (d, 1H, *J* = 2.0 Hz, =CH*H*, M), 5.22 (t, 1H, *J* = 3.3 Hz, Br-CH, m), 4.97 (dd, 1H, *J* = 9.1, 6.1 Hz, Br-CH, M), 4.59 (s, 2H, N-CH_2, m), 4.46 (q, 2H, *J* = 14.9 Hz, N-CH_2, M), 4.00 (d, *J* = 0.5 Hz, 2H, CH_2-Br, m), 3.78 (dd, 2H, *J* = 2.0, 1.3 Hz, N-CH_2-CH, m), 3.61 (t, 1H, *J* = 9.1 Hz, N-C*H*H-CH, M), 3.37 (dd, 1H, *J* = 9.0, 6.0 Hz, N-CH*H*-CH, M); $^{13}C\{1H\}$ NMR (75 MHz, $CDCl_3$, 25 ºC): δ = 157.0 (M + m), 137.5 (M), 135.2 (M), 135.1 (m), 128.9 (Ar, 2CH, M), 128.8 (Ar, 2CH, m), 128.3 (Ar, 2CH, m), 128.1 (Ar, CH, M), 128.1 (m), 128.1 (Ar, 2CH, M), 128.1 (Ar, CH, m), 114.7 (CH_2, M), 98.5 (CH, m), 73.5 (CH, M), 52.3 (CH_2, m), 48.3 (CH_2, M), 47.9 (CH_2, M), 44.6 (CH_2, m), 41.1 (CH_2, m); IR ($CHCl_3$): ν 1757 (C=O), 1257 cm^{-1}; HRMS (ESI) *m/z*: [*M* + H]$^+$ calcd for $C_{12}H_{13}ClNO_2$: 238.0629; found 238.0632.

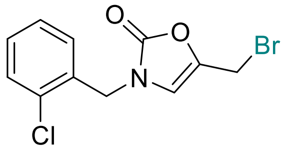

4e

5-(Bromomethyl)-oxazol-2(3*H*)-one 4e. From 100 mg (0.36 mmol) of alkyne **alkyne-e**, and after chromatography of the residue using hexanes/ethyl acetate (10:1) as eluent gave compound **4e** (55 mg, 50%) as a colorless solid; mp 82–84 ºC; ^1H NMR (300 MHz, $CDCl_3$, 25 ºC): δ 7.31 (m, 4H, ArH), 5.88 (t, 1H, *J* =2.8 Hz, CH), 4.61 (s, 2H, N-CH_2), 4.02 (d, 2H, *J* = 2.9 Hz, CH_2-Br); $^{13}C\{1H\}$ NMR (75 MHz, $CDCl_3$, 25 ºC): δ 154.8, 146.1, 133.9, 132.2, 130.4 (Ar, CH), 129.9 (Ar, CH), 129.9 (Ar, CH), 127.5 (Ar, CH), 83.4 (CH), 48.2 (CH_2), 45.4 (CH_2); IR ($CHCl_3$): ν 1790 (C=O), 1062 cm^{-1}; HRMS (ESI) *m/z*: [*M* + H]$^+$ calcd for $C_{11}H_{10}BrClNO_2$: 303.9556; found 303.9542.

Haloheterocyclization of allene 1b in presence of KI. Trapping of intermediate INT4. $CuBr_2$ (95 mg, 0.43 mmol) and KI (57 mg, 0.34 mmol) were sequentially added under argon atmosphere to a solution of the allenyl carbamate **1b** (50 mg, 0.17 mmol) in nitromethane (1 mL). The reaction was stirred at 70 ºC for 5 h (disappearance of the starting material by TLC), and then the mixture was concentrated under reduced pressure. Chromatography of the residue eluting with hexanes/ethyl acetate (8:2) gave 36 mg (62%) of a mixture of iodinated product **2b-I** along with **2b**/*endo*-**2b**.

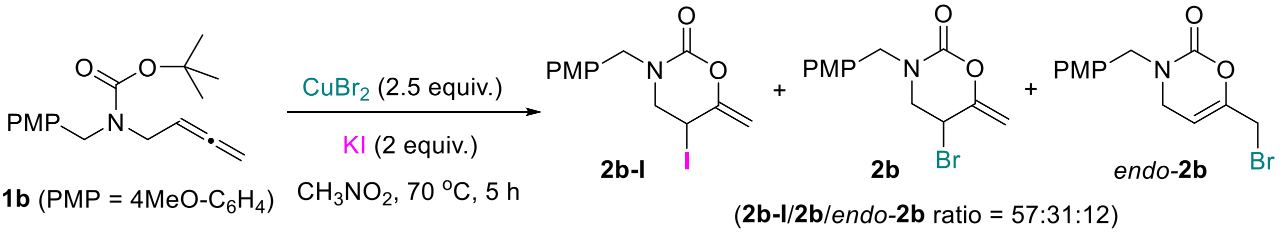

Typical procedure for the preparation of bis(γ-amino vinyl sulfones) 6.

To a solution of the appropriate allenyl carbamate **1** (1.0 mmol) in acetonitrile (10 mL) was added $Cu(OAc)_2$ (20 mol %), the corresponding sodium sulfinate **5** (2.0 mmol) and $AgNO_3$ (2.0 mmol). The reaction was stirred at 100 °C in a sealed tube until completion, which was monitored by TLC. The mixture was cooled to room temperature and was diluted with ethyl acetate (3 × 5 mL). The organic extract was washed with brine (3 × 3 mL), dried over anhydrous $MgSO_4$ and concentrated under reduced pressure. Chromatography of the residue using hexanes/ethyl acetate mixtures gave analytically pure compounds. Spectroscopic and analytical data for pure forms of compounds **6** follow.

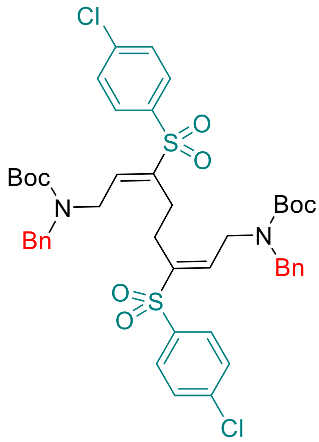

6aa ((*E*,*E*)/(*E*,*Z*) ratio = 85/15)

Bis(γ-amino vinyl sulfone) 6aa. From 20 mg (0.09 mmol) of allene **1a**, and after chromatography of the residue using hexanes/ethyl acetate (5:1) as eluent gave compound **6aa** (14 mg, 35%; (*E*,*E*)/(*E*,*Z*) ratio = 85:15) as a colorless oil; ^1H NMR (300 MHz, $(CCl_2D)_2$, 65 ºC): δ 7.62 (d, 4H, J = 8.6 Hz,

ArH, m), 7.56 (d, 4H, J = 8.6 Hz, ArH, M), 7.45 (m, 4H, ArH, m), 7.38 (m, 4H, ArH, M), 7.25 (m, 12H, ArH, M + m), 7.12 (m, 8H, ArH, M + m), 6.93 (t, 2H, J = 6.6 Hz, CH=, m), 6.63 (t, 2H, J = 6.2 Hz, CH=, M), 4.42 (s, 4H, N-CH_2, m), 4.38 (s, 4H, N-CH_2, M), 4.00 (m, 4H, N-CH_2-CH, m), 3.82 (d, 4H, J = 6.4 Hz, N-CH_2-CH, M), 2.19 (s, 8H, CH_2-CH$_2$, M + m), 1.37 (s, 36H, *t*-Bu, M + m); ^{13}C{1H} NMR (75 MHz, (CCl$_2$D)$_2$, 65 ºC): δ 155.4 (4C, M + m), 140.7 (4C, M + m), 140.5 (4C, M + m), 140.4 (4C, M + m), 137.8 (4C, M + m), 137.6 (4C, M + m), 129.9 (Ar, 8CH, M + m), 129.8 (Ar, 8CH, M + m), 129.1 (Ar, 8CH, M + m), 127.9 (Ar, 8CH, M + m), 127.8 (Ar, 4CH, M + m), 81.1 (4C, M + m), 51.7 (4CH$_2$, M + m), 44.9 (4CH$_2$, M + m), 28.6 (12CH$_3$, M + m), 26.2 (4CH$_2$, M + m); IR (CHCl$_3$): ν 1699 (C=O), 1154 cm^{-1}; HRMS (ESI) *m/z*: [*M* + Na]$^+$ calcd for $C_{44}H_{50}Cl_2N_2NaO_8S_2$: 891.2278; found 891.2258.

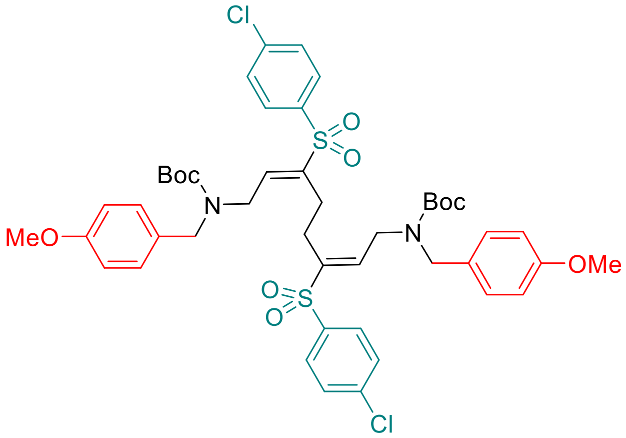

6ba

Bis(γ-amino vinyl sulfone) 6ba. From 25 mg (0.10 mmol) of allene **1b**, and after chromatography of the residue using hexanes/ethyl acetate (4:1) as eluent gave compound **6ba** (31 mg, 66%) as a colorless solid; mp 147–149 ºC; ^{1}H NMR (300 MHz, (CCl$_2$D)$_2$, 75 ºC): δ 7.56 (d, 4H, J = 8.7 Hz, ArH), 7.39 (d, 4H, J = 8.7 Hz, ArH), 7.06 (d, 4H, J = 8.7 Hz, ArH), 6.80 (d, 4H, J = 8.7 Hz, ArH), 6.61 (t, 2H, J = 6.3 Hz, CH=), 4.32 (s, 4H, N-CH_2), 3.83 (d, 4H, J = 6.3 Hz, N-CH_2-CH), 3.74 (s, 6H, O-CH$_3$), 2.21 (s, 4H, CH_2-CH$_2$), 1.39 (s, 18H, *t*-Bu); ^{13}C{1H} NMR (75 MHz, (CCl$_2$D)$_2$, 55 ºC): δ 159.4 (2C), 155.4 (2C), 141.0 (2C), 140.5 (2C), 140.2 (2C), 137.6 (2C), 129.9 (Ar, 4CH), 129.8 (Ar, 4CH), 129.7 (2C), 129.2 (Ar, 4CH), 114.6 (Ar, 4CH), 81.0 (2C), 55.7 (2CH$_3$-O), 51.2 (2CH$_2$), 44.8 (2CH$_2$), 28.7 (6CH$_3$), 26.2 (2CH$_2$); IR (CHCl$_3$): ν 1690 (C=O), 1611, 1247 cm^{-1}; HRMS (ESI) *m/z*: [*M* + Na]$^+$ calcd for $C_{46}H_{54}Cl_2N_2NaO_{10}S_2$: 951.2489; found 951.2517.

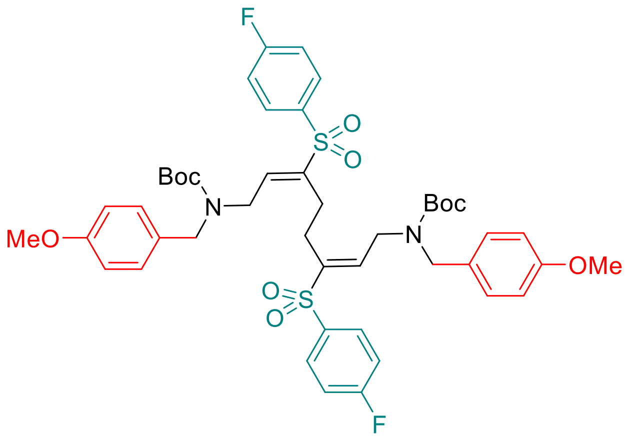

6bb

Bis(γ-amino vinyl sulfone) 6bb. From 48 mg (0.17 mmol) of allene **1b**, and after chromatography of the residue using hexanes/ethyl acetate (6:1) as eluent gave compound **6bb** (28 mg, 36%) as a yellow solid; mp 150.5–151.9 ºC; ^{1}H NMR (300 MHz, $(CCl_2D)_2$, 65 ºC): δ 7.65 (m, 4H, ArH), 7.07 (m, 8H, ArH), 6.79 (d, 4H, J = 8.7 Hz, ArH), 6.60 (t, 2H, J = 6.2 Hz, C*H*=), 4.32 (s, 4H, N-C*H*$_2$), 3.83 (d, 4H, J = 6.3 Hz, N-C*H*$_2$-CH), 3.73 (s, 6H, O-CH_3), 2.21 (s, 4H, C*H*$_2$-CH_2), 1.36 (s, 18H, *t*-Bu); ^{13}C{1H} NMR (75 MHz, $(CCl_2D)_2$, 65 ºC): δ 165.9 (d, J = 256.5 Hz, 2C), 159.5 (2C), 155.4 (2C), 140.7 (2C), 140.6 (2C), 135.2 (d, J = 3.1 Hz, 2C), 131.1 (d, J = 9.5 Hz, Ar, 4CH), 129.9 (2C), 129.2 (Ar, 4CH), 116.8 (d, J = 22.7 Hz, Ar, 4CH), 114.6 (Ar, 4CH), 80.9 (2C), 55.7 (2CH_3-O), 51.2 (2CH_2), 44.9 (2CH_2), 28.7 (6CH_3), 26.2 (2CH_2); ^{19}F NMR (282 MHz, $(CCl_2D)_2$, 65 ºC): δ –103.1 (s, 1F, C–F); IR ($CHCl_3$): ν 1691 (C=O), 1493, 1145 cm^{-1}; HRMS (ESI) *m/z*: $[M + Na]^+$ calcd for $C_{46}H_{54}F_2N_2NaO_{10}S_2$: 919.3086; found 919.3080.

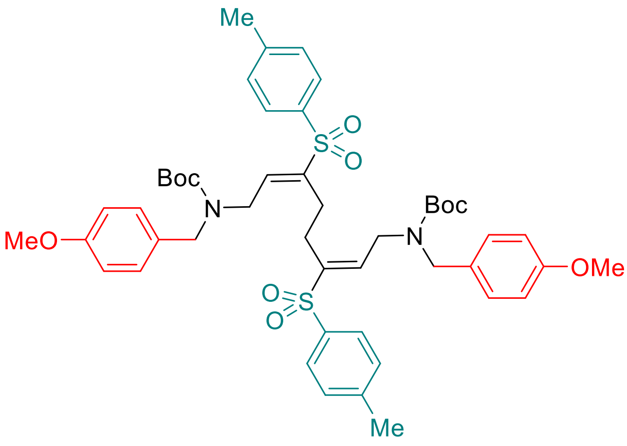

6bc ((*E*,*E*)/(*E*,*Z*) ratio = 70/30)

Bis(γ-amino vinyl sulfone) 6bc. From 42 mg (0.14 mmol) of allene **1b**, and after chromatography of the residue using hexanes/ethyl acetate [4:1]→[2:1] as eluent gave compound **6bc** (47 mg, 73%; (*E*,*E*)/(*E*,*Z*) ratio = 70:30) as a colorless oil; ^{1}H NMR (300 MHz, $(CCl_2D)_2$, 65 ºC): δ 7.60 (m, 4H,

ArH, m), 7.49 (d, 4H, J = 8.3 Hz, ArH, M), 7.25 (m, 4H, ArH, m), 7.18 (d, 4H, J = 8.0 Hz, ArH, M), 7.05 (d, 4H, J = 8.6 Hz, ArH, M), 6.97 (m, 4H, ArH, m), 6.79 (m, 8H, ArH, M + m), 6.58 (t, 2H, J = 6.1 Hz, C*H*=, M), 6.52 (m, 2H, C*H*=, m), 4.31 (m, 6H, N-CH_2, M + m), 4.23 (m, 2H, N-CH_2, m), 3.98 (m, 2H, N-CH_2-CH, m), 3.92 (d, 2H, J = 6.7 Hz, N-CH_2-CH, m), 3.79 (d, 4H, J = 6.7 Hz, N-CH_2-CH, M), 3.73 (s, 12H, O-CH_3, M + m), 2.36 (m, 12H, CH_3, M + m), 2.16 (s, 8H, CH_2-CH_2, M + m), 1.38 (s, 36H, *t*-Bu, M + m); ^{13}C{1H} NMR (75 MHz, $(CCl_2D)_2$, 65 °C): δ 159.5 (4C, M + m), 155.4 (4C, M + m), 144.8 (4C, M + m), 141.1 (4C, M + m), 139.8 (4C, M + m), 136.3 (4C, M + m), 130.1 (Ar, 8CH, M + m), 129.9 (4C, M + m), 129.2 (Ar, 8CH, M + m), 128.5 (Ar, 2CH, m), 128.3 (Ar, 6CH, M), 114.6 (Ar, 6CH, M), 114.3 (Ar, 2CH, m), 80.8 (4C, M + m), 55.7 (4CH_3-O, M + m), 51.1 (4CH_2, M + m), 44.9 (4CH_2, M + m), 28.7 (6CH_3, M), 28.6 (6CH_3, m), 26.3 (4CH_2, M + m), 21.8 (4CH_3, M + m); IR ($CHCl_3$): ν 1698 (C=O), 1152 cm^{-1}; HRMS (ESI) *m/z*: [*M* + Na]$^+$ calcd for $C_{48}H_{60}N_2NaO_{10}S_2$: 911.3582; found 911.3576.

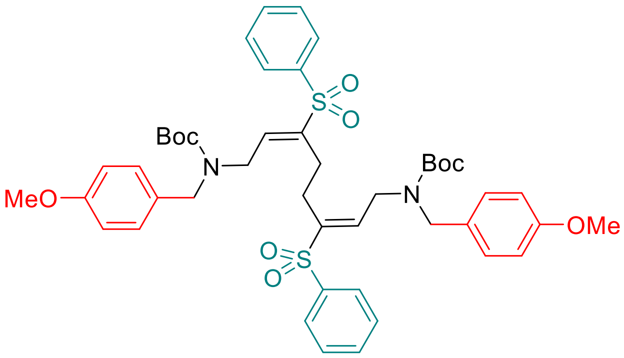

6bd ((*E*,*E*)/(*E*,*Z*) ratio = 70/30)

Bis(γ-amino vinyl sulfone) 6bd. From 31 mg (0.11 mmol) of allene **1b**, and after chromatography of the residue using hexanes/ethyl acetate [4:1]→[2:1] as eluent gave compound **6bd** (27 mg, 62%; (*E*,*E*)/(*E*,*Z*) ratio = 70:30) as a yellow oil; ^{1}H NMR (300 MHz, $(CCl_2D)_2$, 65 °C): δ 7.86-7.37 (m, 22H, ArH, M + m,), 7.05 (d, 4H, J = 8.7 Hz, ArH, M), 6.93 (m, 4H, ArH, m), 6.80 (m, 6H, ArH, M + m; 2H, C*H*=, m), 6.61 (t, 2H, J = 6.0 Hz, C*H*=, M), 4.32 (m, 8H, N-CH_2, M + m), 4.01 (m, 2H, N-CH_2-CH, m), 3.93 (d, 2H, J = 6.7 Hz, N-CH_2-CH, m), 3.80 (d, 4H, J = 6.7 Hz, N-CH_2-CH, M), 3.75 (s, 6H, O-CH_3, m), 3.73 (s, 6H, O-CH_3, M), 2.17 (s, 8H, CH_2-CH_2, M + m), 1.37 (s, 36H, *t*-Bu, M + m); ^{13}C{1H} NMR (75 MHz, $(CCl_2D)_2$, 65 °C): δ 159.6 (2C, m), 159.5 (2C, M), 155.4 (4C, M + m), 140.7 (4C, M + m), 140.5 (4C, M + m), 139.5 (4C, M + m), 134.5 (Ar, CH, m), 133.9 (Ar, CH, m), 133.7 (Ar, 2CH, M + m), 132.9 (2C, M + m), 129.9 (2C, M + m), 129.7 (Ar, 2CH, M + m), 129.6 (Ar, 4CH, m), 129.5 (Ar, 4CH, M), 129.4 (Ar, 2CH, m), 129.2 (Ar, 4CH, M), 128.6 (Ar, 2CH, m),

128.4, (Ar, 2CH, m), 128.2 (Ar, 4CH, M), 114.7 (Ar, 4CH, m), 114.6 (Ar, 4CH, M), 81.0 (2C, m) , 80.8 (2C, M), 55.7 (4CH$_3$-O, M + m), 54.1 (2CH$_2$, m), 51.1 (2CH$_2$, M), 46.0 (2CH$_2$, m), 44.9 (2CH$_2$, M), 28.7 (6CH$_3$, M), 28.6 (6CH$_3$, m), 26.2 (4CH$_2$, M + m); IR (CHCl$_3$): ν 1698 (C=O), 1612, 1155 cm^{-1}; HRMS (ESI) *m/z*: [M + Na]$^+$ calcd for $C_{46}H_{56}N_2NaO_{10}S_2$: 883.3269; found 883.3257.

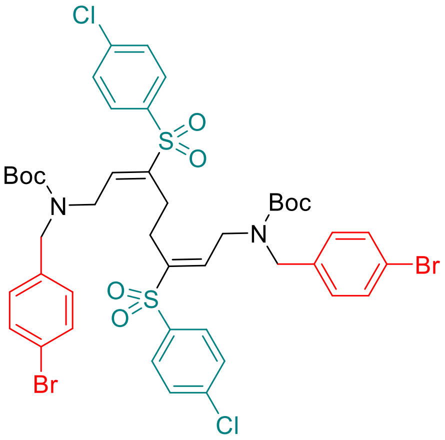

6ca

Bis(γ-amino vinyl sulfone) 6ca. From 45 mg (0.13 mmol) of allene **1c**, and after chromatography of the residue using hexanes/ethyl acetate [6:1]→[2:1] as eluent gave compound **6ca** (17 mg, 25%) as a colorless solid; mp 181–183 ºC; ^{1}H NMR (300 MHz, (CCl$_2$D)$_2$, 65 ºC): δ 7.58 (d, 4H, J = 8.3 Hz, ArH), 7.41 (m, 8H, ArH), 7.02 (d, 4H, J = 8.1 Hz, ArH), 6.63 (t, 2H, J = 6.3 Hz, C*H*=), 4.33 (s, 4H, N-C*H$_2$*), 3.87 (d, 4H, J = 6.4 Hz, N-C*H$_2$*-CH), 2.26 (s, 4H, C*H$_2$*-CH$_2$), 1.38 (s, 18H, *t*-Bu); ^{13}C{1H} NMR (75 MHz, (CCl$_2$D)$_2$, 65 ºC): δ 155.3 (2C), 140.9 (2C), 140.6 (2C), 140.4 (2C), 137.7 (2C), 137.0 (2C), 132.2 (Ar, 4CH), 129.9 (Ar, 4CH), 129.7 (Ar, 4CH), 129.5 (Ar, 4CH), 121.7 (2C), 81.3 (2C), 51.3 (2CH$_2$), 45.3 (2CH$_2$), 28.6 (6CH$_3$), 26.3 (2CH$_2$); IR (CHCl$_3$): ν 1690 (C=O), 1475, 1146 cm^{-1}; HRMS (ESI) *m/z*: [M + Na]$^+$ calcd for $C_{44}H_{48}Br_2Cl_2N_2NaO_8S_2$: 1047.0488; found 1047.0498.

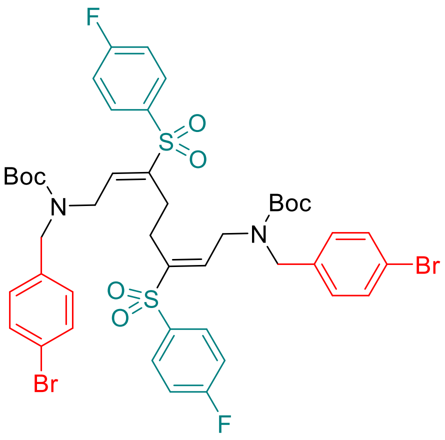

6cb ((*E*,*E*)/(*E*,*Z*) ratio = 90/10)

Bis(γ-amino vinyl sulfone) 6cb. From 44 mg (0.13 mmol) of allene **1c**, and after chromatography of the residue using hexanes/ethyl acetate [6:1]→[2:1] as eluent gave compound **6cb** (29 mg, 45%; (*E*,*E*)/(*E*,*Z*) ratio = 90:10) as a colorless solid; mp 151–153 °C; ^{1}H NMR (300 MHz, $(CCl_2D)_2$, 65 °C): δ 7.67 (m, 8H, ArH, M + m), 7.40 (m, 8H, ArH, M + m), 7.19 - 6.92 (m, 16H, ArH, M + m; 2H, C*H*=, m), 6.61 (t, 2H, *J* = 6.2 Hz, C*H*=, M), 4.37 (s, 4H, N-CH_2, m), 4.33 (s, 4H, N-CH_2, M), 4.07 (s, 2H, N-CH_2-CH, m), 4.02 (d, 2H, *J* = 6.3 Hz, N-CH_2-CH, m), 3.87 (d, 4H, *J* = 6.1 Hz, N-CH_2-CH, M), 2.27 (s, 8H, CH_2-CH_2, M + m), 1.37 (s, 36H, *t*-Bu, M + m); ^{13}C{1H} NMR (75 MHz, $(CCl_2D)_2$, 65 °C): δ 165.9 (d, *J* = 256.8 Hz, 4C, M + m), 155.3 (4C, M + m), 141.2 (4C, M + m), 140.0 (4C, M + m), 137.1 (4C, M + m), 135.2 (d, *J* = 3.2 Hz, 4C, M + m), 132.2 (Ar, 4CH, m), 132.1 (Ar, 4CH, M), 131.5 (d, *J* = 9.9 Hz, Ar, 4CH, m), 131.1 (d, *J* = 9.5 Hz, Ar, 4CH, M), 129.5 (Ar, 8CH, M + m), 121.7 (4C, M + m), 117.0 (d, *J* = 22.5 Hz, Ar, 4CH, m), 116. 9 (d, *J* = 22.7 Hz, Ar, 4CH, M), 81.3 (4C, M + m), 54.2 (2CH_2, m), 51.3 (2CH_2, M), 46.6 (2CH_2, m), 45.3 (2CH_2, M), 28.6 (12CH_3, M + m), 26.3 (4CH_2, M + m); ^{19}F NMR (282 MHz, $(CCl_2D)_2$, 65 °C): δ –102.8 (s, 1F, C–F); IR ($CHCl_3$): ν 1692 (C=O), 1590, 1145 cm^{-1}; HRMS (ESI) *m/z*: [*M* + Na]$^{+}$ calcd for $C_{44}H_{48}Br_2F_2N_2NaO_8S_2$: 1015.1079; found 1015.1077.

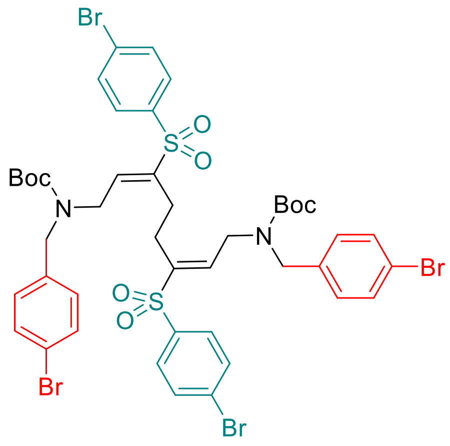

6ce

Bis(γ-amino vinyl sulfone) 6ce. From 57 mg (0.17 mmol) of allene **1c**, and after chromatography of the residue using hexanes/ethyl acetate [5:1]→[3:1] as eluent gave compound **6ce** (36 mg, 49%) as a colorless solid; mp 182–183 °C; ^{1}H NMR (300 MHz, $(CCl_2D)_2$, 65 °C): δ 7.59 (d, 4H, *J* = 8.6 Hz, ArH), 7.50 (d, 4H, *J* = 8.6 Hz, ArH), 7.40 (d, 4H, *J* = 8.4 Hz, ArH), 7.02 (d, 4H, *J* = 8.3 Hz, ArH), 6.63 (t, 2H, *J* = 6.3 Hz, C*H*=), 4.33 (s, 4H, N-CH_2), 3.86 (d, 4H, *J* = 6.4 Hz, N-CH_2-CH), 2.26 (s, 4H, CH_2-CH_2), 1.38 (s, 18H, *t*-Bu); ^{13}C{1H} NMR (75 MHz, $(CCl_2D)_2$, 65 °C): δ = 155.3 (2C), 140.9 (2C), 140.5 (2C), 138.3 (2C), 137.0 (2C), 133.0 (2C), 132.2 (Ar, 4CH), 129.8 (Ar, 4CH), 129.5 (Ar,

4CH), 129.2 (Ar, 4CH), 121.7 (2C), 81.3 (2C), 51.3 (2CH_2), 45.3 (2CH_2), 28.6 (6CH_3), 26.3 (2CH_2); IR ($CHCl_3$): ν 1691 (C=O), 1573, 1154 cm^{-1}; HRMS (ESI) *m/z*: $[M + Na +2]^+$ calcd for $C_{44}H_{48}Br_4N_2NaO_8S_2$: 1136.9460; found 1136.9436.

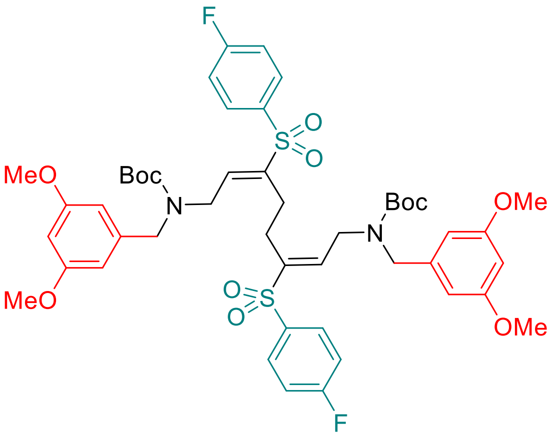

6db

Bis(γ-amino vinyl sulfone) 6db. From 58 mg (0.18 mmol) of allene **1d**, and after chromatography of the residue using hexanes/ethyl acetate (2:1) as eluent gave compound **6db** (43 mg, 50%) as a pale yellow solid; mp 140–142 ºC; ^1H NMR (300 MHz, $(CCl_2D)_2$, 65 ºC): δ 7.65 (m, 4H, ArH), 7.08 (t, 4H, J = 8.5 Hz, ArH), 6.64 (t, 2H, J = 6.3 Hz, C*H*=), 6.33 (m, 6H, ArH), 4.32 (s, 4H, N-C*H*$_2$), 3.87 (d, 4H, J = 6.2 Hz, N-C*H*$_2$-CH), 3.71 (s, 12H, O-CH_3), 2.23 (s, 4H, C*H*$_2$-CH_2), 1.38 (s, 18H, *t*-Bu); $^{13}C\{1H\}$ NMR (75 MHz, $(CCl_2D)_2$, 65 ºC): δ 165.9 (d, J = 256.5 Hz, 2C), 161.5 (4C), 155.4 (2C), 140.8 (2C), 140.4 (2C), 140.3 (2C), 135.3 (d, J = 3.2 Hz, 2C), 131.1 (d, J = 9.5 Hz, Ar, 4CH), 116.8 (d, J = 22.7 Hz, Ar, 4CH), 106.0 (Ar, 4CH), 100.1 (Ar, 2CH), 81.0 (2C), 55.7 (4CH_3-O), 51.9 (2CH_2), 45.2 (2CH_2), 28.6 (6CH_3), 26.2 (2CH_2); ^{19}F NMR (282 MHz, $(CCl_2D)_2$, 65 ºC): δ –103.2 (s, 1F, C–F); IR ($CHCl_3$): ν 1691 (C=O), 1493, 1145 cm^{-1}; HRMS (ESI) *m/z*: $[M + Na]^+$ calcd for $C_{48}H_{58}F_2N_2NaO_{12}S_2$: 979.3291; found 979.3294.

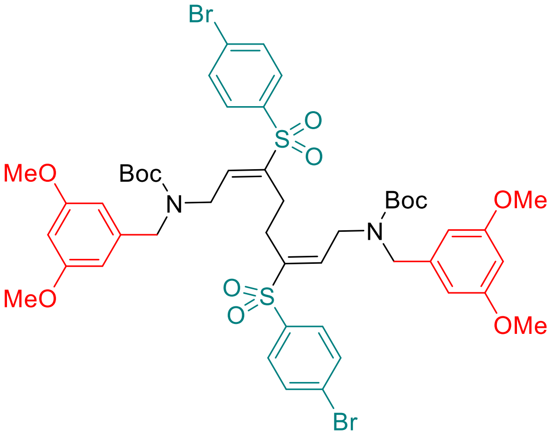

6de

Bis(γ-amino vinyl sulfone) 6de. From 49 mg (0.15 mmol) of allene **1d**, and after chromatography of the residue using hexanes/ethyl acetate (2:1) as eluent gave compound **6de** (28 mg, 34%) as a pale yellow solid; mp 140–142 ºC; ^{1}H NMR (300 MHz, $(CCl_2D)_2$, 65 ºC): δ 7.56 (d, 4H, *J* = 8.6 Hz, ArH), 7.48 (d, 4H, *J* = 8.6 Hz, ArH), 6.65 (t, 2H, *J* = 6.2 Hz, C*H*=), 6.43–6.22 (m, 6H, ArH), 4.32 (s, 4H, N-C*H*$_2$), 3.86 (d, 4H, *J* = 6.2 Hz, N-C*H*$_2$-CH), 3.71 (s, 12H, O-CH_3), 2.22 (s, 4H, C*H*$_2$-CH_2), 1.39 (s, 18H, *t*-Bu); ^{13}C{1H} NMR (75 MHz, $(CCl_2D)_2$, 65 ºC): δ 161.5 (4C), 155.4 (2C), 140.9 (2C), 140.5 (2C), 140.3 (2C), 138.4 (2C), 132.9 (Ar, 4CH), 129.8 (Ar, 4CH), 129.0 (2C), 105.9 (Ar, 4CH), 100.1 (Ar, 2CH), 81.1 (2C), 55.7 (4CH_3-O), 51.9 (2CH_2), 45.3 (2CH_2), 28.7 (6CH_3), 26.2 (2C); IR ($CHCl_3$): ν 1690 (C=O), 1456, 1156 cm^{-1}; HRMS (ESI) *m/z*: $[M + Na]^+$ calcd for $C_{48}H_{58}Br_2N_2NaO_{12}S_2$: 1099.1690; found 1099.1685.

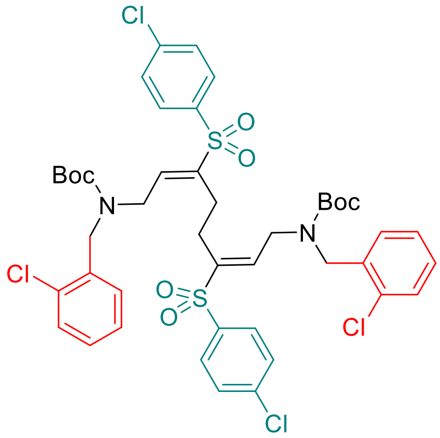

6ea

Bis(γ-amino vinyl sulfone) 6ea. From 30 mg (0.10 mmol) of allene **1e**, and after chromatography of the residue using hexanes/ethyl acetate (5:1) as eluent gave compound **6ea** (19 mg, 40%) as a colorless oil; ^{1}H NMR (300 MHz, $(CCl_2D)_2$, 55 ºC): δ 7.59 (d, 4H, *J* = 8.6 Hz, ArH), 7.39 (d, 4H, *J* = 8.7 Hz, ArH), 7.28 (m, 2H, ArH), 7.19 (m, 6H, ArH), 6.65 (t, 2H, *J* = 6.2 Hz, C*H*=), 4.49 (s, 4H, N-C*H*$_2$), 3.86 (m, 4H, N-C*H*$_2$-CH), 2.20 (s, 4H, C*H*$_2$-CH_2), 1.36 (s, 18H, *t*-Bu); ^{13}C{1H} NMR (75 MHz, $(CCl_2D)_2$, 55 ºC): δ 155.3 (2C), 140.8 (4C), 140.6 (2C), 137.7 (2C), 136.1 (2C), 133.5 (2C), 130.0 (Ar, 4CH), 129.9 (Ar, 4CH), 129.8 (Ar, 4CH), 129.2 (Ar, 2CH), 127.5 (Ar, 2CH), 81.3 (2C), 49.1 (2CH_2), 45.1 (2CH_2), 28.6 (6CH_3), 26.2 (2CH_2); IR ($CHCl_3$): ν 1691 (C=O), 1581, 1154 cm^{-1}; HRMS (ESI) *m/z*: $[M + Na]^+$ calcd for $C_{44}H_{48}C_{14}N_2NaO_8S_2$: 959.1498; found 959.1486.

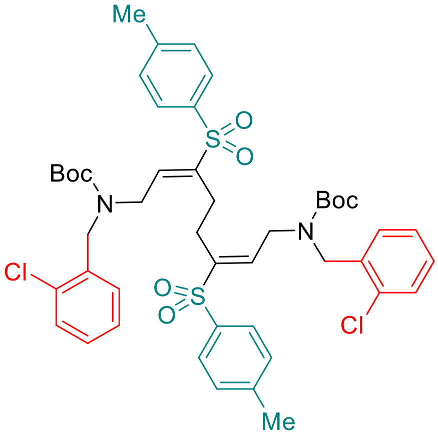

6ec ((*E*,*E*)/(*E*,*Z*) ratio = 75/25)

Bis(γ-amino vinyl sulfone) 6ec. From 76 mg (0.26 mmol) of allene **1e**, and after chromatography of the residue using hexanes/ethyl acetate [9:1]→[2:1] as eluent gave compound **6ec** (24 mg, 20%; (*E*,*E*)/(*E*,*Z*) ratio = 75:25) as a yellow solid; mp 184–186 ºC; ^{1}H NMR (300 MHz, $(CCl_2D)_2$, 55 ºC): δ 7.53 (m, 8H, ArH, M + m), 7.42–7.00 (m, 24H, ArH, M + m), 6.93 (t, 2H, *J* = 6.6 Hz, C*H*=, m), 6.61 (t, 2H, *J* = 6.0 Hz, C*H*=, M), 4.49 (m, 8H, N-CH_2, M + m), 3.85 (m, 8H, N-CH_2-CH, M + m), 2.37 (s, 6H, CH_3, m), 2.35 (s, 6H, CH_3, M), 2.15 (s, 8H, CH_2-CH_2, M + m), 1.34 (s, 36H, *t*-Bu, M + m); ^{13}C{1H} NMR (75 MHz, $(CCl_2D)_2$, 55 ºC): δ 155.3 (4C, M + m), 144.9 (4C, M + m), 141.4 (4C, M + m), 139.4 (4C, M + m), 136.1 (4C, M + m), 135.2 (4C, M + m), 133.5 (4C, M + m), 130.2 (Ar, 8CH, M + m), 130.0 (Ar, 4CH, M + m), 129.2 (Ar, 4CH, m), 129.1 (Ar, 4CH, M), 128.7 (Ar, 2CH, m), 129.1 (Ar, 2CH, m), 128.3 (Ar, 4CH, M), 127.5 (Ar, 4CH, M + m), 81.1 (4C, M + m), 49.0 (4CH_2, M + m), 45.2 (4CH_2, M + m), 28.6 (12CH_3, M + m), 26.3 (4CH_2, M + m), 21.9 (4CH_3, M + m); IR ($CHCl_3$): ν 1720 (C=O), 1581, 1279 cm^{-1}; HRMS (ESI) *m/z*: $[M + Na]^+$ calcd for $C_{46}H_{54}Cl_2N_2NaO_8S_2$: 919.2591; found 919.2603.

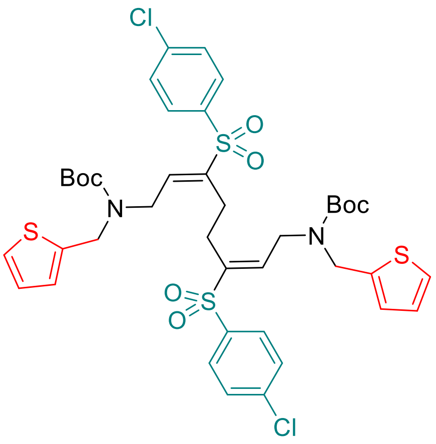

6fa ((*E*,*E*)/(*E*,*Z*) ratio = 80/20)

Bis(γ-amino vinyl sulfone) 6fa. From 40 mg (0.15 mmol) of allene **1f**, and after chromatography of the residue using hexanes/ethyl acetate (3:1) as eluent gave compound **6fa** (28 mg, 43%; (*E*,*E*)/(*E*,*Z*) ratio = 80:20) as a colorless oil; ^{1}H NMR (300 MHz, $(CCl_2D)_2$, 55 ºC): δ 7.62 (m, 8H, ArH, M + m), 7.40 (m, 8H, ArH, M + m), 7.20 (m, 4H, ArH, M + m), 6.86 (m, 8H, ArH, M + m; 2H, C*H*=, m), 6.63 (t, 2H, *J* = 6.2 Hz, C*H*=, M), 4.54 (m, 6H, N-CH_2, M + m), 4.11 (m, 2H, N-CH_2, m), 4.04 (d, 4H, *J* = 6.2 Hz, N-CH_2-CH, m), 3.89 (d, 4H, *J* = 6.1 Hz, N-CH_2-CH, M), 2.29 (s, 8H, CH_2-CH_2, M + m), 1.40 (s, 36H, *t*-Bu, M + m); ^{13}C{1H} NMR (75 MHz, $(CCl_2D)_2$, 55 ºC): δ 155.6 (4C, M + m), 141.4 (4C, M + m), 141.31 (4C, M + m), 141.2 (4C, M + m), 138.4 (4C, M + m), 130.71 (4C, M + m), 130.6 (Ar, 8CH, M + m), 130.5 (Ar, 8CH, M + m), 127.8 (Ar, 4CH, M + m), 127.3 (Ar, 4CH, M + m), 126.7 (Ar, 4CH, M + m), 82.1 (4C, M + m), 47.5 ($4CH_2$, M + m), 45.7 ($4CH_2$, M + m), 29.4 ($12CH_3$, M + m), 27.0 ($4CH_2$, M + m); IR ($CHCl_3$): ν 1692 (C=O), 1581, 1089 cm^{-1}; HRMS (ESI) *m/z*: [*M* + Na]$^+$ calcd for $C_{40}H_{46}Cl_2N_2NaO_8S_4$: 903.1406; found 903.1396.

General procedure for the preparation of mixed γ-amino vinyl sulfones 7.

To a solution of the appropriate allenyl carbamate **1** (1.0 mmol) in acetonitrile (10 mL) was added $Cu(OAc)_2$ (20 mol %), sodium sulfinate **5a** (1.0 mmol), sodium sulfinate **5e** (1.0 mmol) and $AgNO_3$ (2.0 mmol). The reaction was stirred at 100 °C in a sealed tube until completion (typically 24 h, monitored by TLC). The mixture was cooled to room temperature and was diluted with ethyl acetate (3 × 5 mL). The organic extract was washed with brine (3 × 3 mL), dried over anhydrous $MgSO_4$ and concentrated under reduced pressure. Chromatography of the residue using hexanes/ethyl acetate mixtures gave analytically pure compounds. Spectroscopic and analytical data for pure forms of compounds **7** follow.

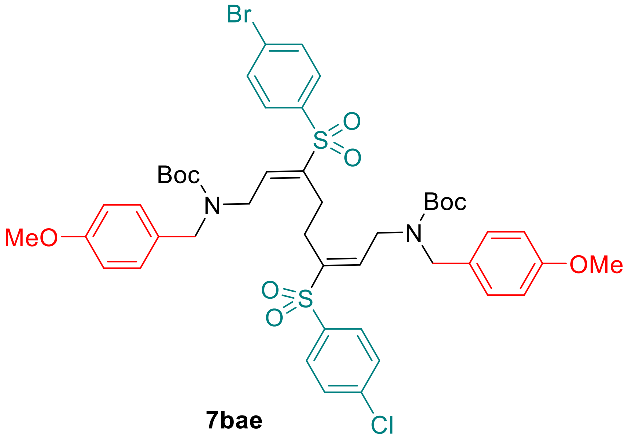

7bae

Mixed γ-amino vinyl sulfone 7bae. From 50 mg (0.17 mmol) of allene **1b**, and after chromatography of the residue using hexanes/ethyl acetate (8:1) as eluent gave compound **7bae** (38 mg, 48%) as a pale yellow solid; mp 114–116 ºC; ^{1}H NMR (300 MHz, $(CCl_2D)_2$, 65 ºC): δ 7.56 (d, 4H, *J* = 8.6 Hz, ArH), 7.47 (d, 2H, *J* = 8.6 Hz, ArH), 7.38 (d, 2H, *J* = 8.6 Hz, ArH), 7.05 (d, 4H, *J* = 8.6 Hz, ArH), 6.80 (d, 4H, *J* = 8.6 Hz, ArH), 6.61 (t, 2H, *J* = 6.2 Hz, C*H*=), 4.32 (s, 4H, N-CH_2), 3.82 (d, 4H, *J* = 6.3 Hz, N-CH_2-CH), 3.74 (s, 6H, O-CH_3), 2.19 (s, 4H, CH_2-CH_2), 1.39 (s, 18H, *t*-Bu); ^{13}C NMR (75 MHz, $(CCl_2D)_2$, 55 ºC): δ 159.4 (2C), 155.4 (2C), 141.1, 141.0, 140.5 (2C), 140.2, 140.1, 138.2, 137.7, 132.9 (Ar, 2CH), 129.9 (Ar, 4CH), 129.8 (Ar, 2CH), 129.7 (Ar, 2CH), 129.2 (Ar, 2CH), 129.1 (2C), 114.6 (Ar, 4CH), 81.0 (2C), 55.7 (2CH_3-O), 51.2 (2CH_2), 44.9 (2CH_2), 28.7 (6CH_3), 26.2 (2CH_2); IR ($CHCl_3$): ν 1690 (C=O), 1247 cm^{-1}; HRMS (ESI) *m/z*: [*M* + Na]$^{+}$ calcd for $C_{46}H_{54}BrClN_2NaO_{10}S_2$: 997.1969; found: 997.1975.

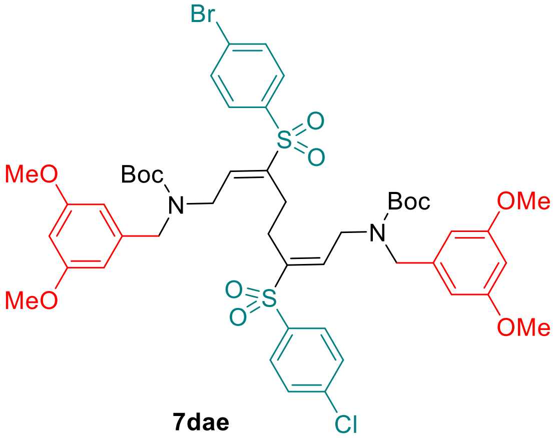

7dae

Mixed γ-amino vinyl sulfone 7dae. From 50 mg (0.16 mmol) of allene **1d**, and after chromatography of the residue using hexanes/ethyl acetate (6:1) as eluent gave compound **7dae** (30 mg, 40%) as a yellow solid; mp 93–95 ºC; ^{1}H NMR (300 MHz, $(CCl_2D)_2$, 65 ºC): δ 7.56 (d, 4H, J = 8.6 Hz, ArH), 7.48 (d, 2H, J = 8.6 Hz, ArH), 7.38 (d, 2H, J = 8.6 Hz, ArH), 6.65 (t, 2H, J = 6.2 Hz, C*H*=), 6.37–6.25 (m, 6H, ArH), 4.32 (s, 4H, N-CH_2), 3.86 (d, 4H, J = 6.0 Hz, N-CH_2-CH), 3.71 (s, 12H, O-CH_3), 2.22 (s, 4H, CH_2-CH_2), 1.38 (s, 18H, *t*-Bu); ^{13}C NMR (75 MHz, $(CCl_2D)_2$, 55 ºC): δ 161.4 (4C), 155.4 (2C), 140.8 (2C), 140.5, 140.4, 140.3 (2C), 138.2 (2C), 137.6 (2C), 132.9 (Ar, 2CH), 129.9 (Ar, 2CH), 129.8 (Ar, 2CH), 129.7 (Ar, 2CH), 105.8 (Ar, 4CH), 99.9 (Ar, 2CH), 81.1 (2C), 55.7 (4CH_3-O), 51.9 (2CH_2), 45.2 (2CH_2), 28.6 (6CH_3), 26.2 (2C); IR ($CHCl_3$): ν 1690 (C=O), 1455, 1142 cm^{-1}; HRMS (ESI) *m/z*: $[M + Na]^+$ calcd for $C_{48}H_{58}BrClN_2NaO_{12}S_2$: 1057.2181; found: 1057.2184.

4.- NMR Spectra

^{1}H NMR (300 MHz) spectrum of **alkyne-e** in $CDCl_3$ at 25 ºC

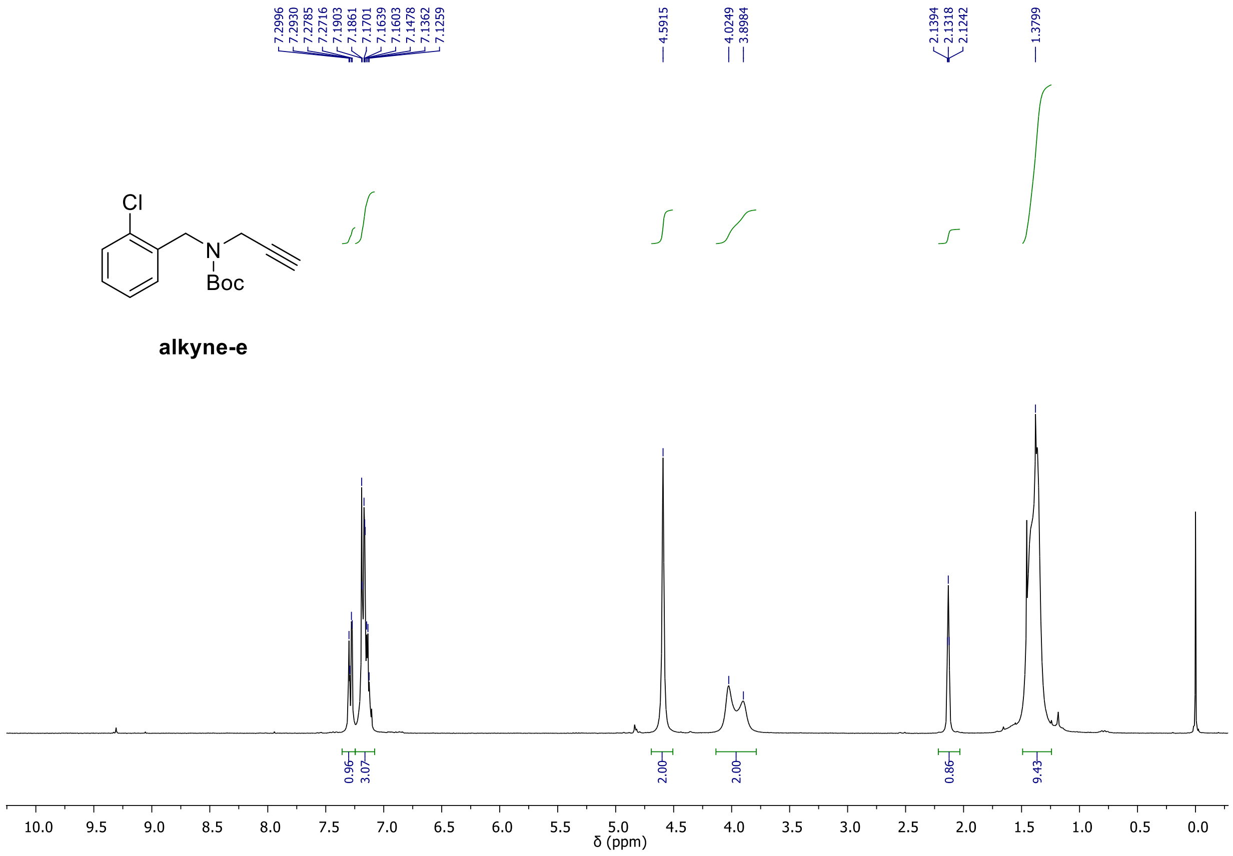

^{13}C NMR (75 MHz) spectrum of **alkyne-e** in $CDCl_3$ at 25 ºC

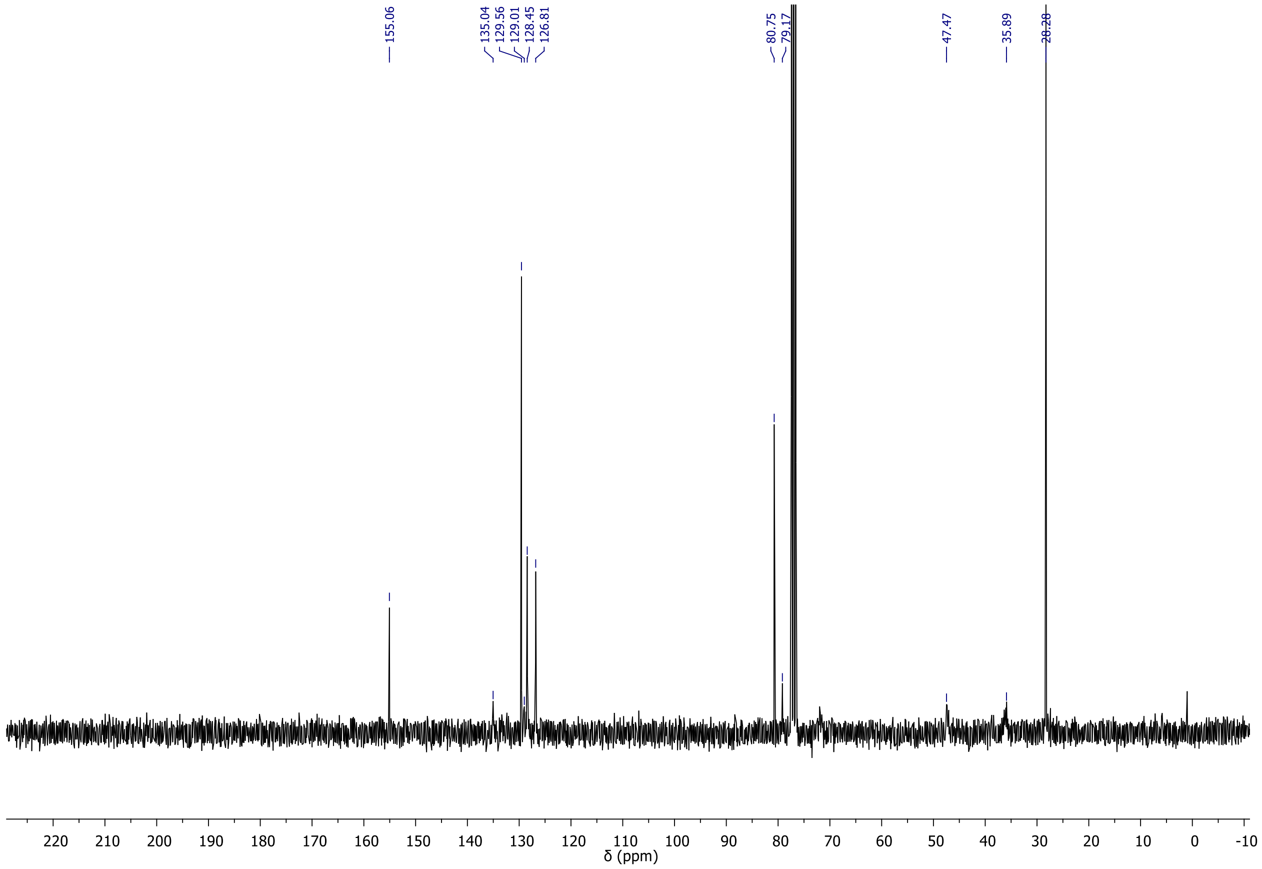

^{1}H NMR (300 MHz) spectrum of **1c** in $CDCl_3$ at 25 ºC

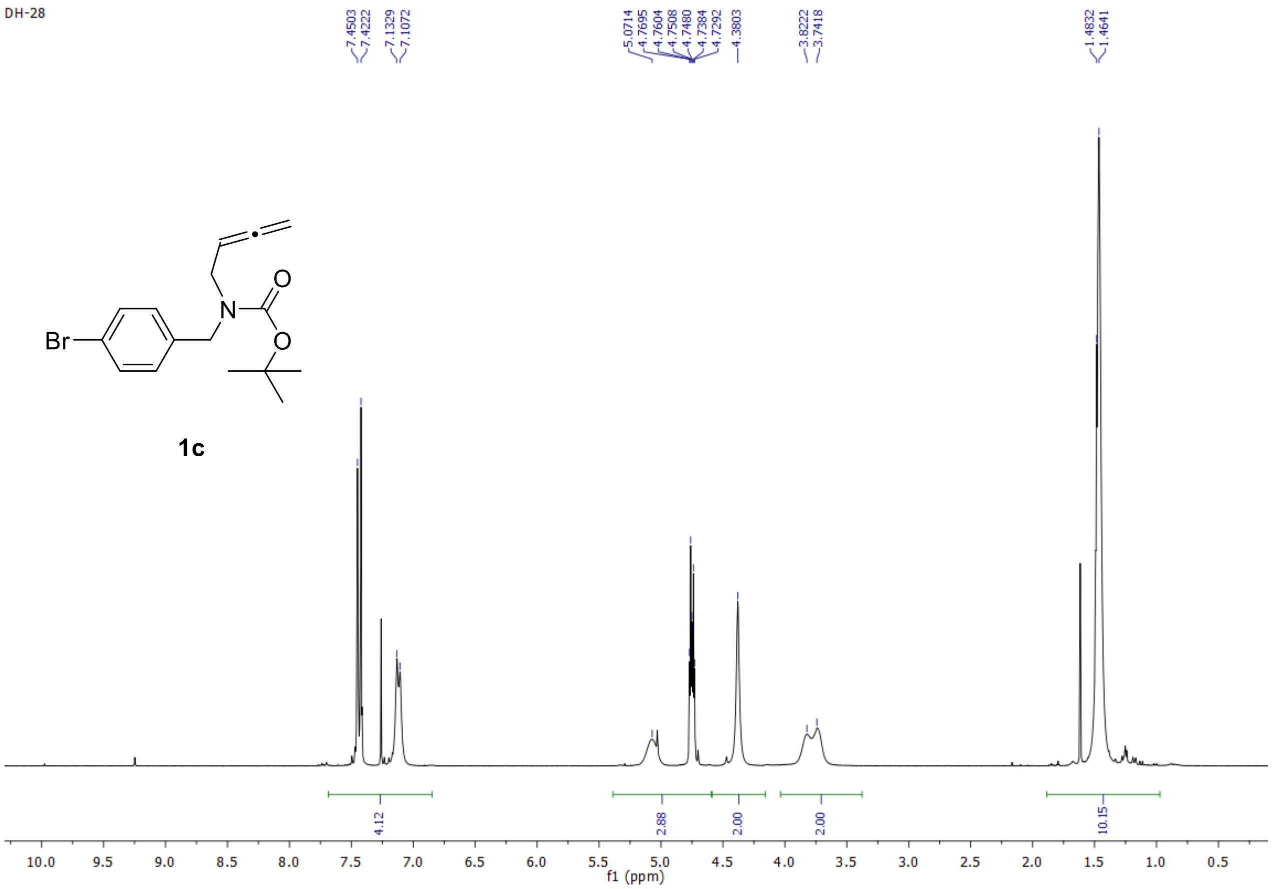

^{13}C NMR (75 MHz) spectrum of **1c** in $CDCl_3$ at 25 ºC

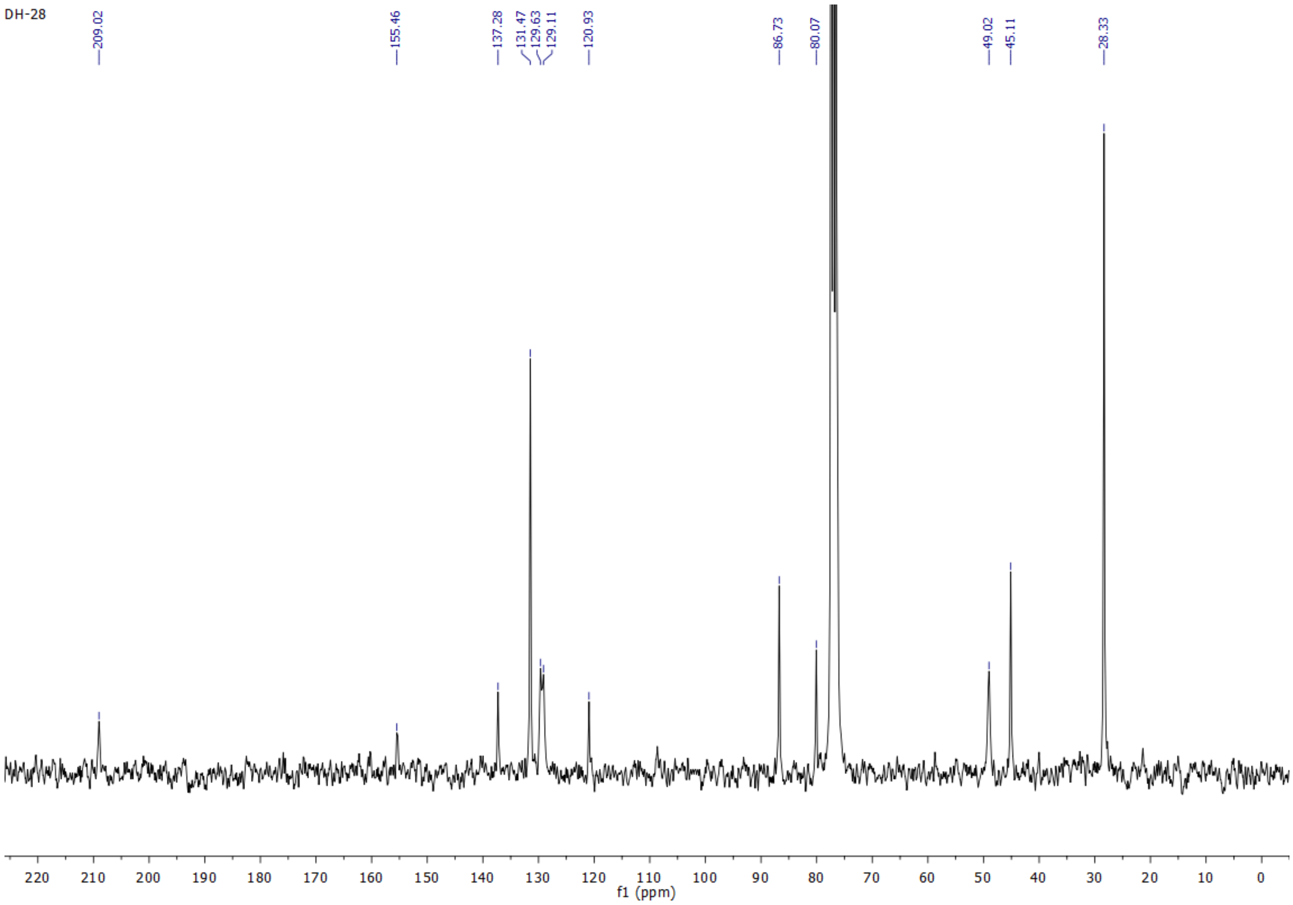

^{1}H NMR (300 MHz) spectrum of **1d** in $CDCl_3$ at 25 ºC

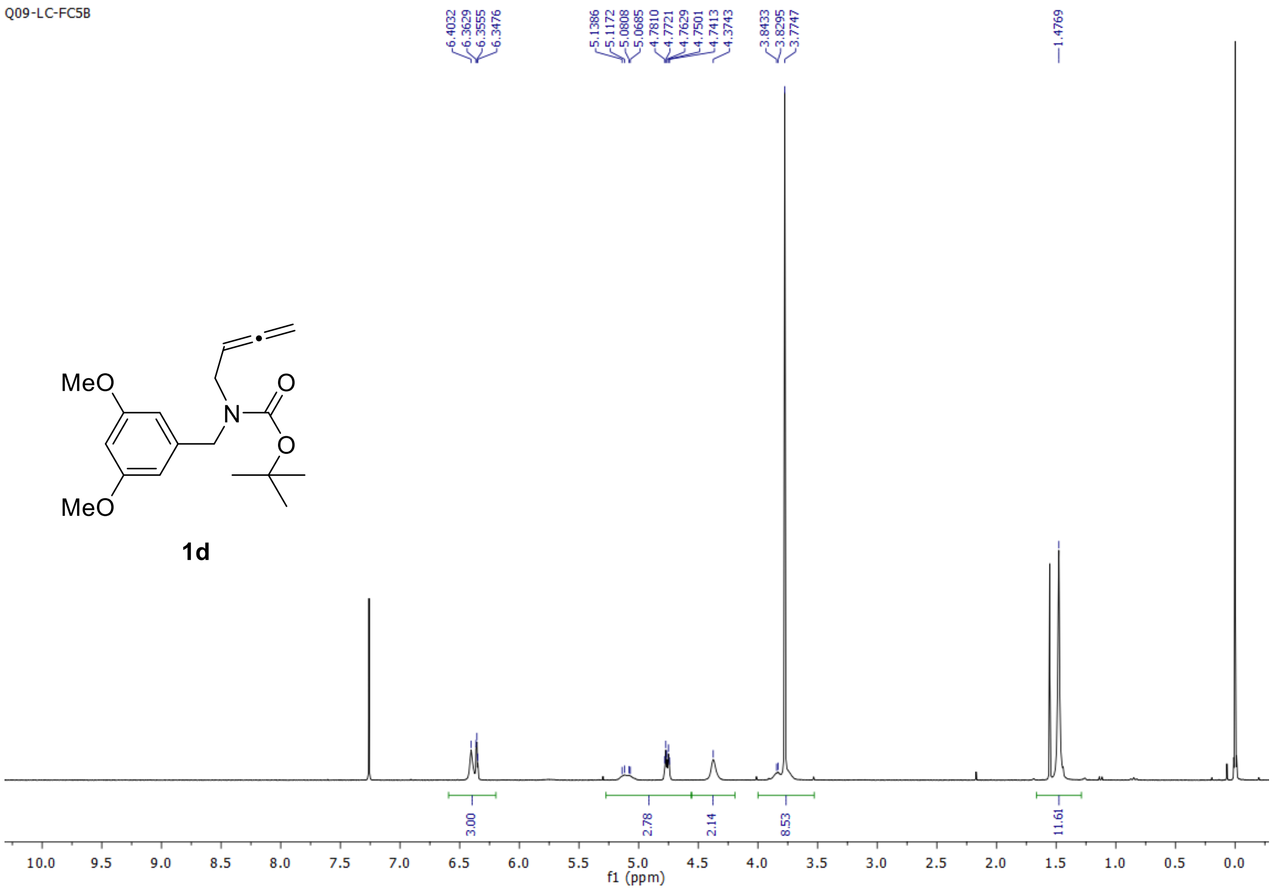

^{13}C NMR (75 MHz) spectrum of **1d** in $CDCl_3$ at 25 ºC

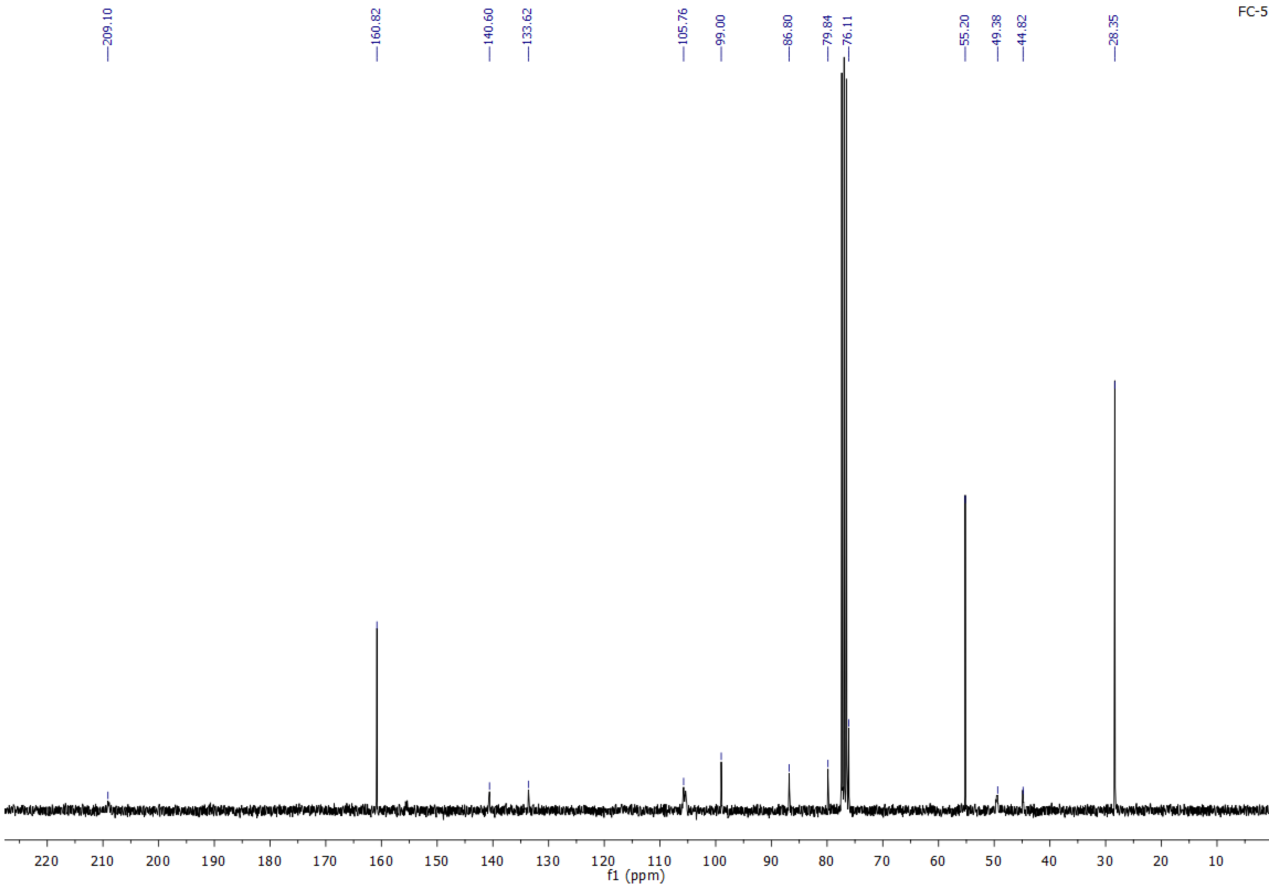

^{1}H NMR (300 MHz) spectrum of **1e** in $CDCl_3$ at 25 ºC

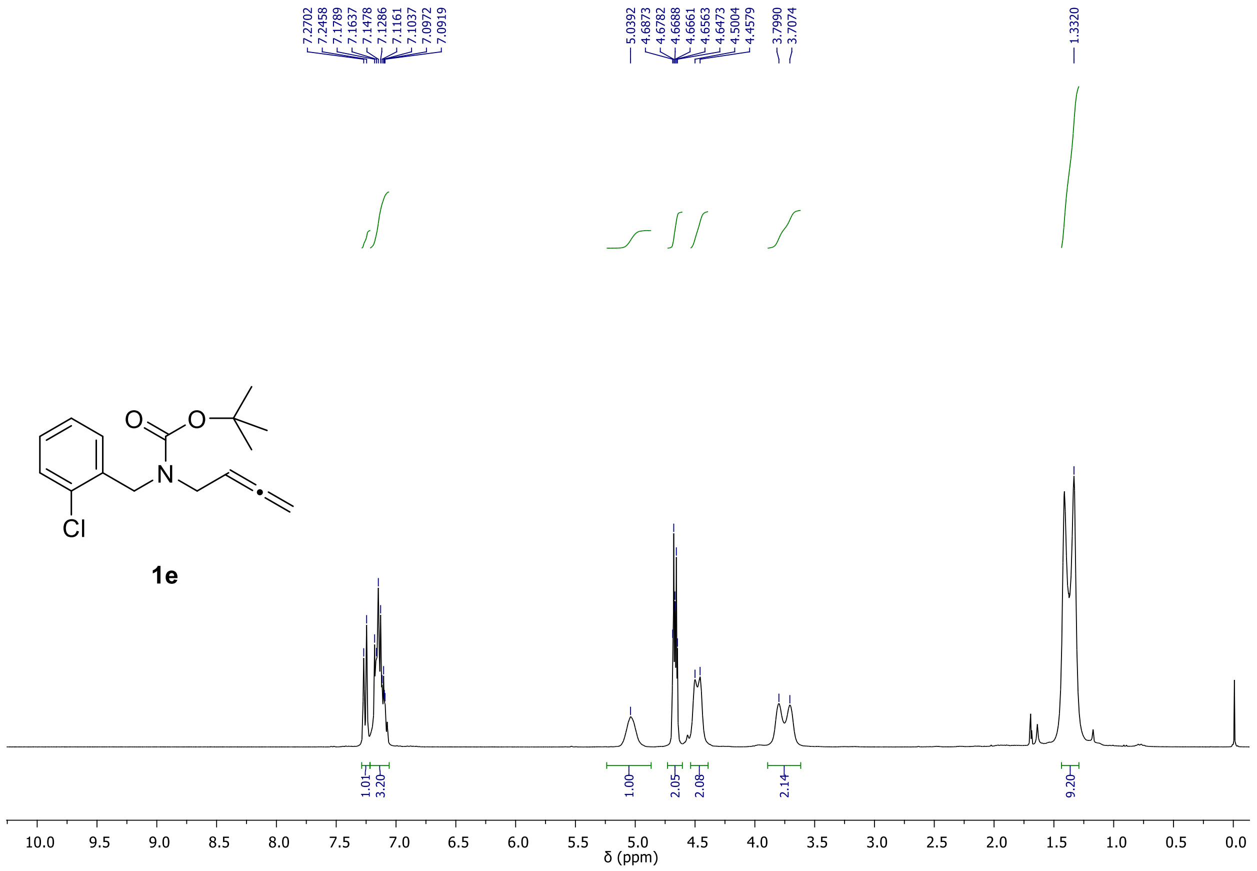

^{13}C NMR (75 MHz) spectrum of **1e** in $CDCl_3$ at 25 ºC

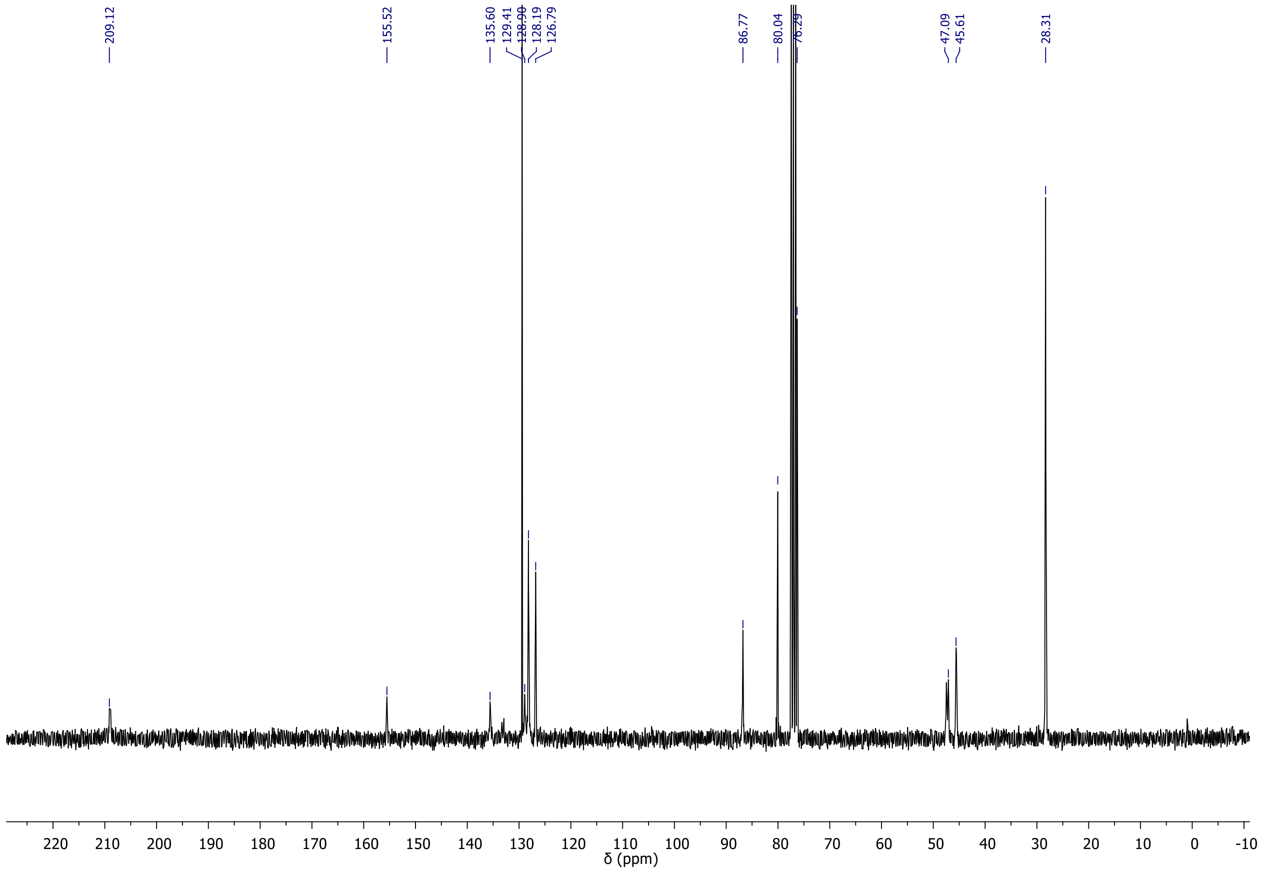

^{1}H NMR (300 MHz) spectrum of **1f** in $CDCl_3$ at 25 ºC

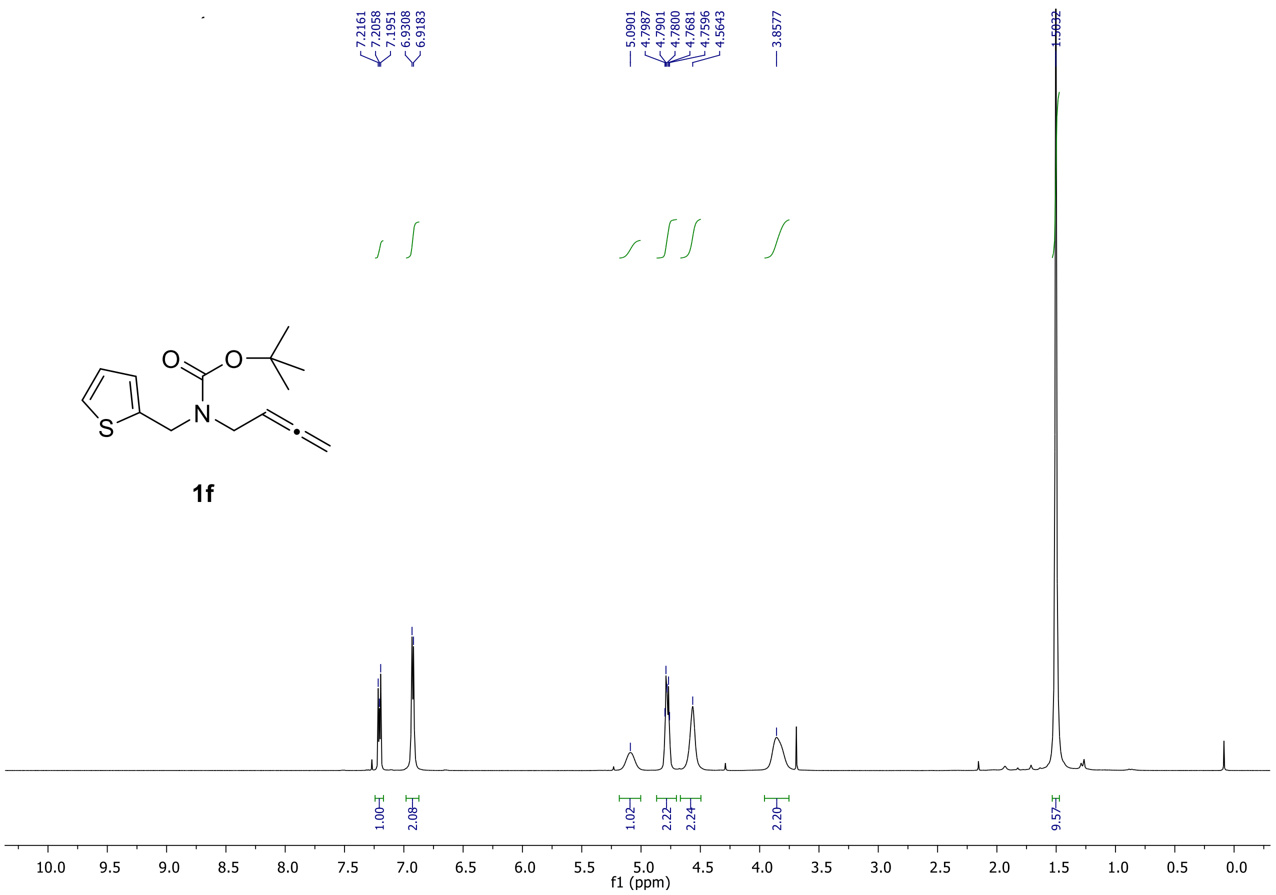

^{13}C NMR (75 MHz) spectrum of **1f** in $CDCl_3$ at 25 ºC

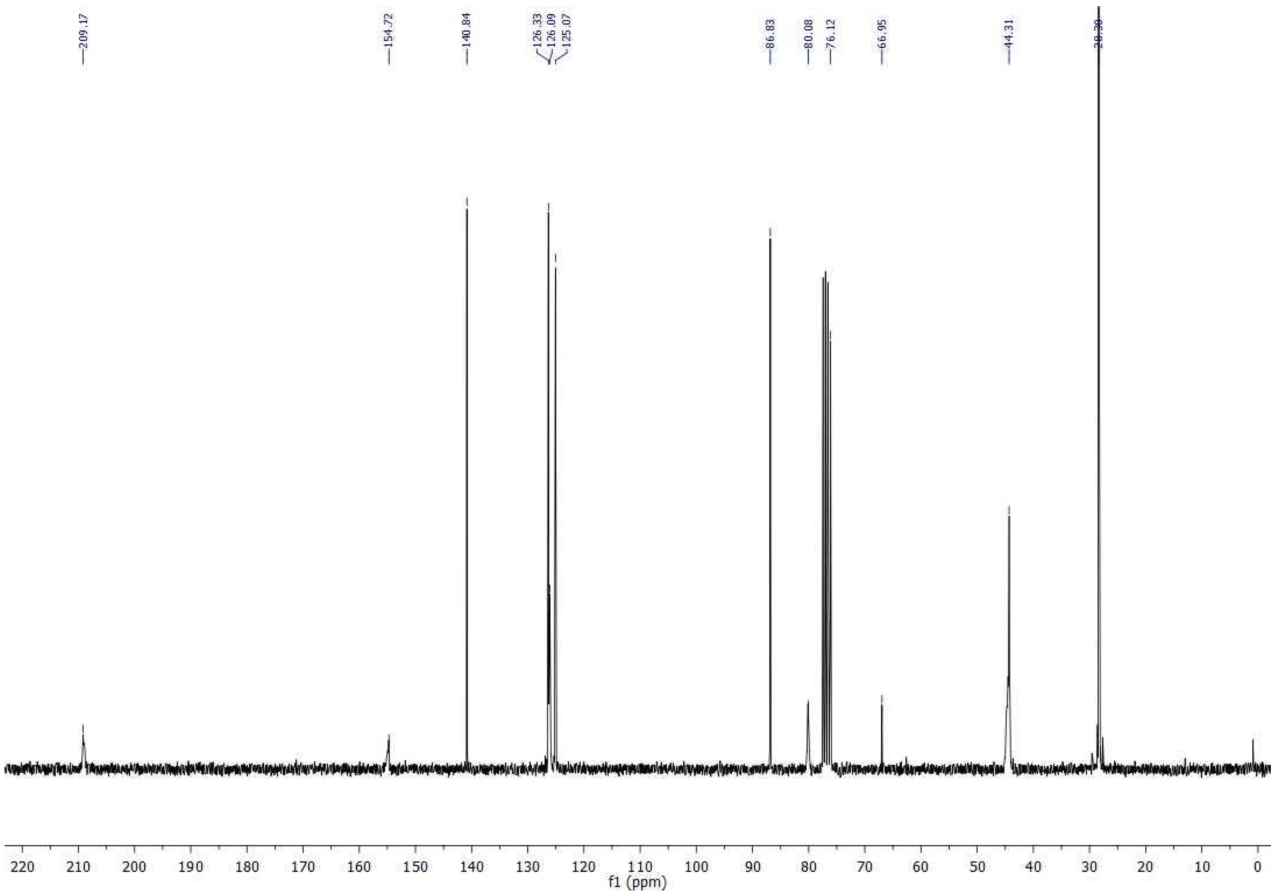

^{1}H NMR (300 MHz) spectrum of **2a** in $CDCl_3$ at 25 ºC

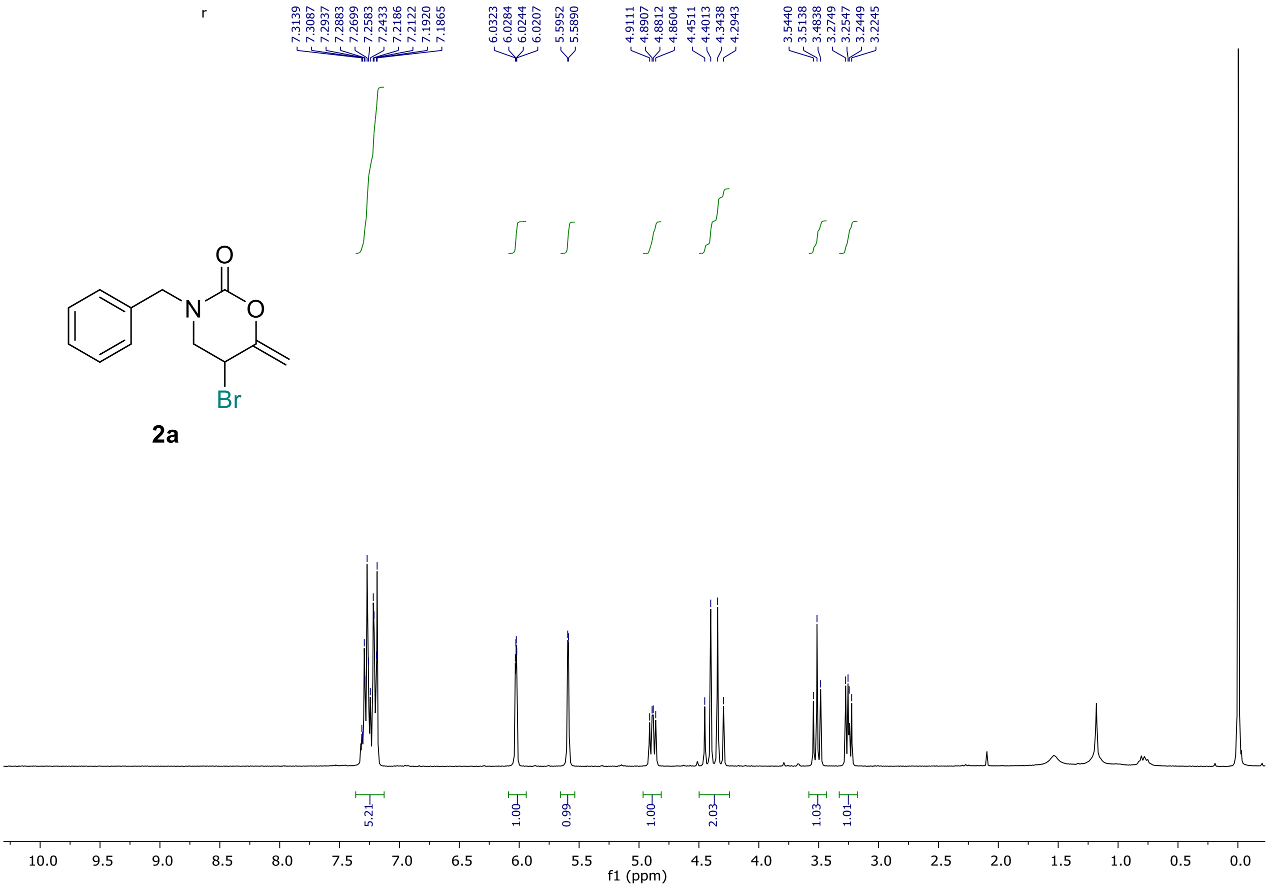

^{13}C NMR (75 MHz) spectrum of **2a** in $CDCl_3$ at 25 ºC

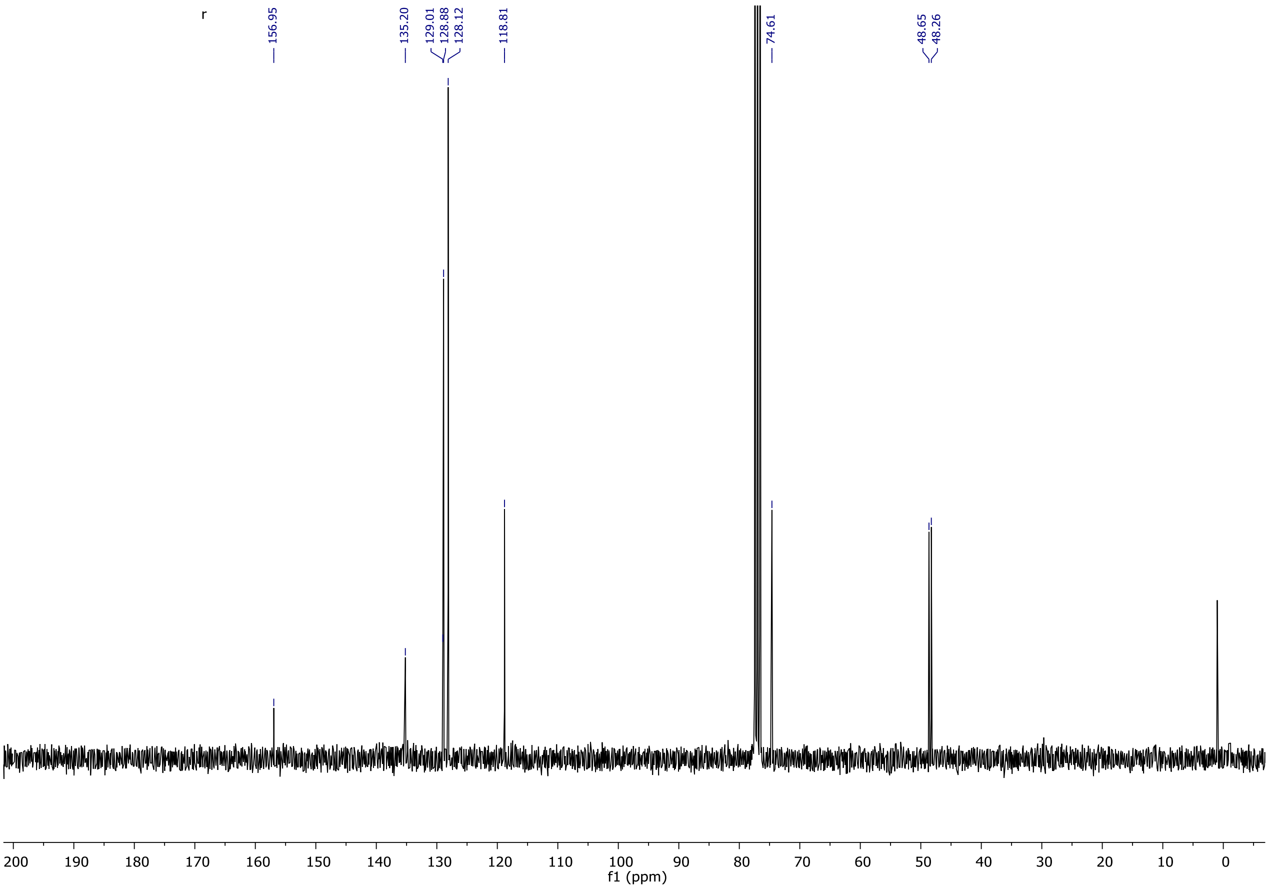

^{1}H NMR (300 MHz) spectrum of **2a-Cl** in $CDCl_3$ at 25 ºC

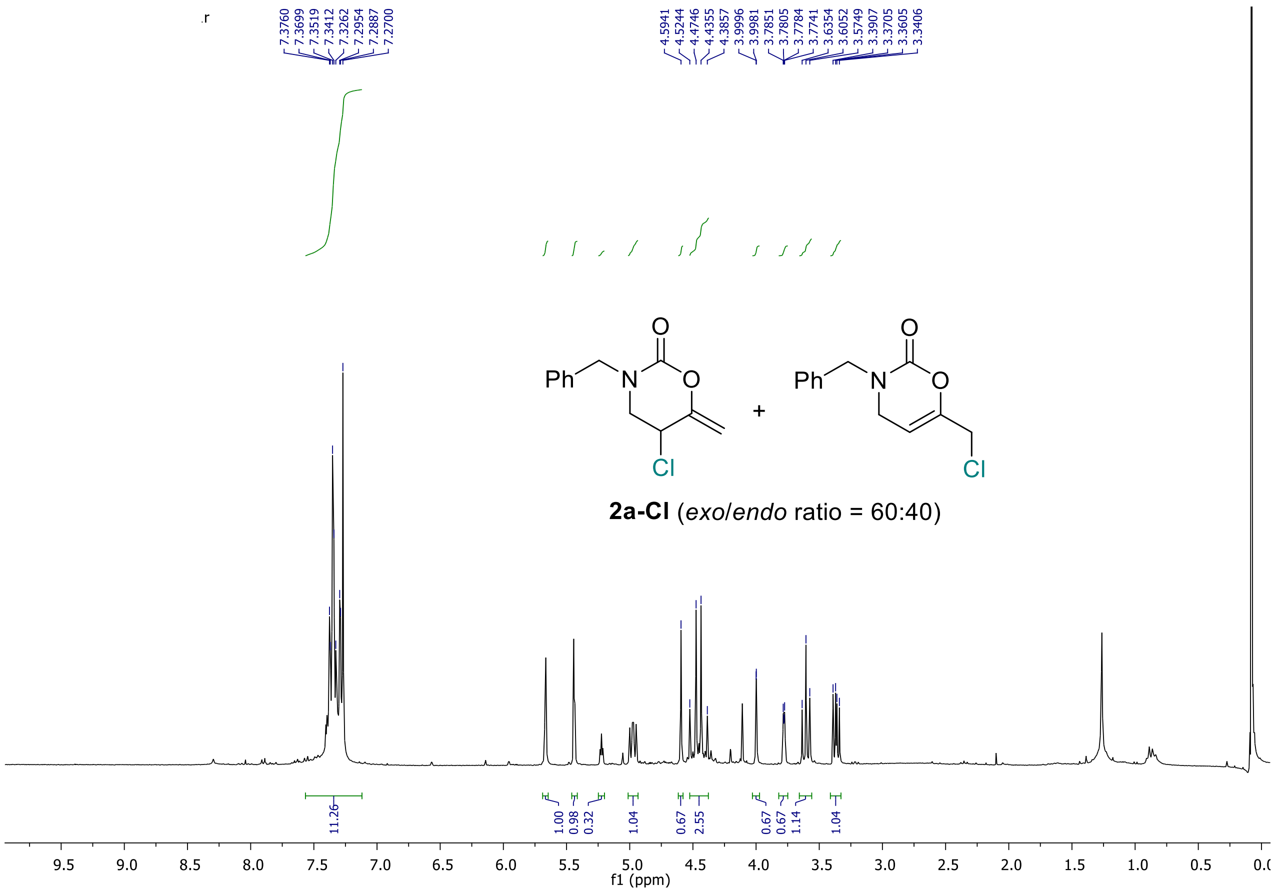

^{13}C NMR (75 MHz) spectrum of **2a-Cl** in $CDCl_3$ at 25 ºC

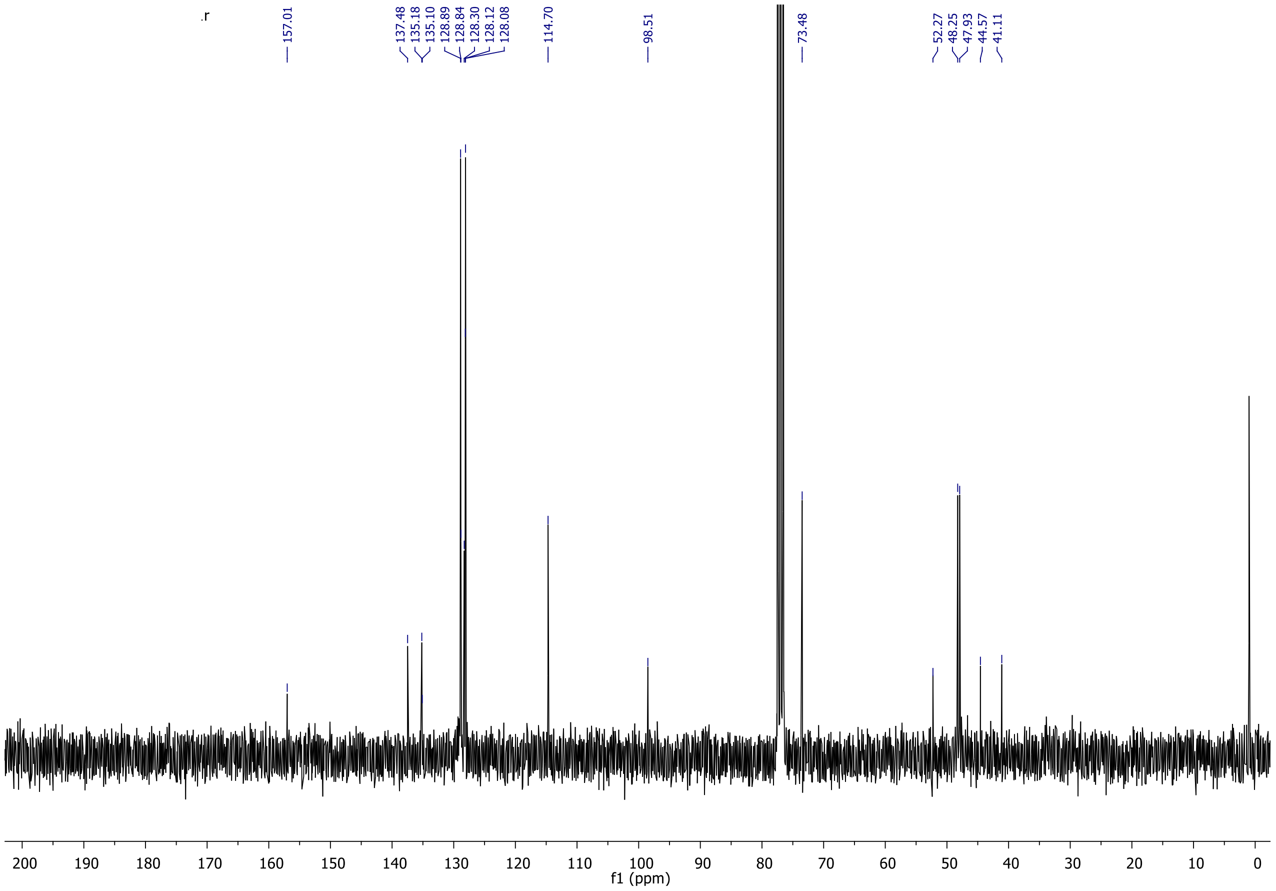

^{1}H NMR (300 MHz) spectrum of **2b** in $CDCl_3$ at 25 ºC

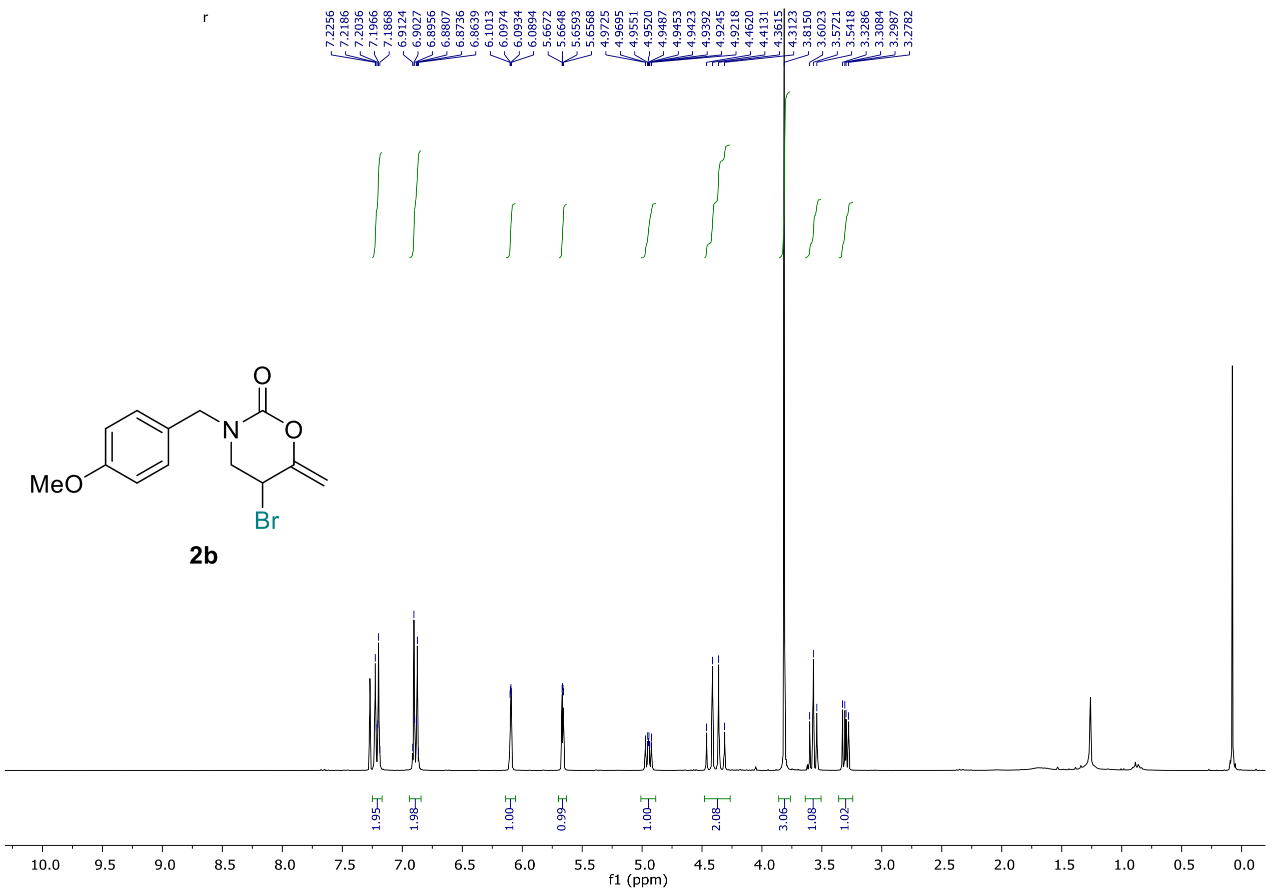

^{13}C NMR (75 MHz) spectrum of **2b** in $CDCl_3$ at 25 ºC

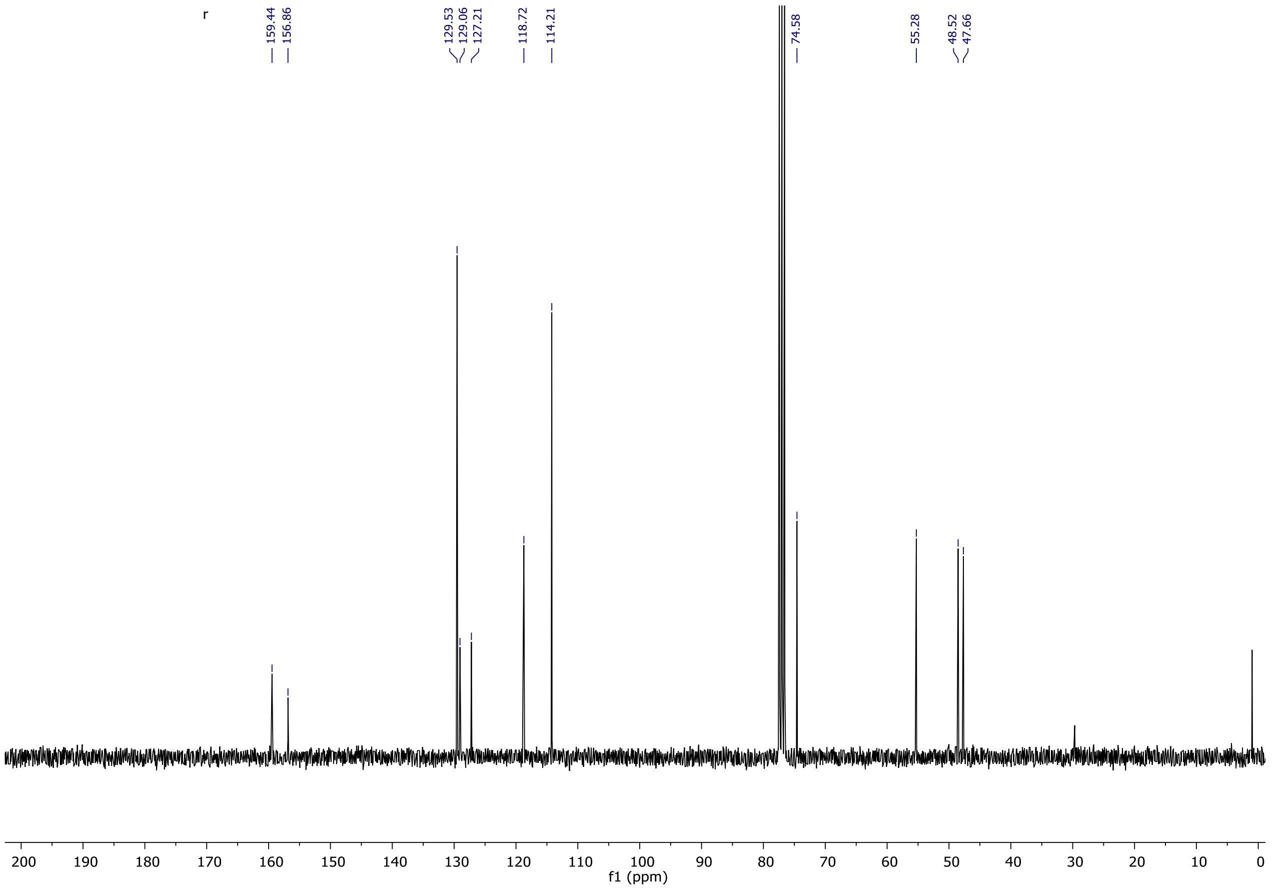

^{1}H NMR (300 MHz) spectrum of **2c** in $CDCl_3$ at 25 ºC

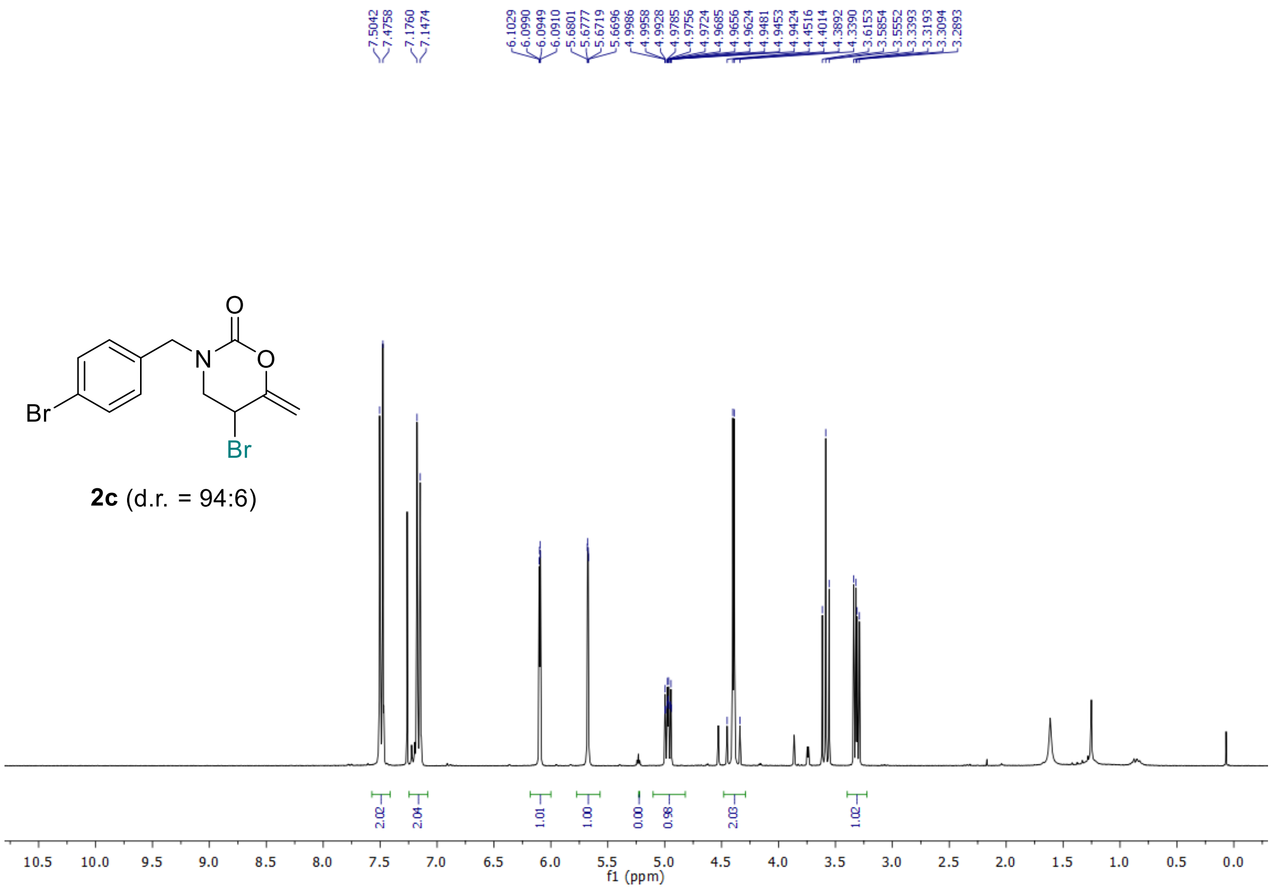

^{13}C NMR (75 MHz) spectrum of **2c** in $CDCl_3$ at 25 ºC

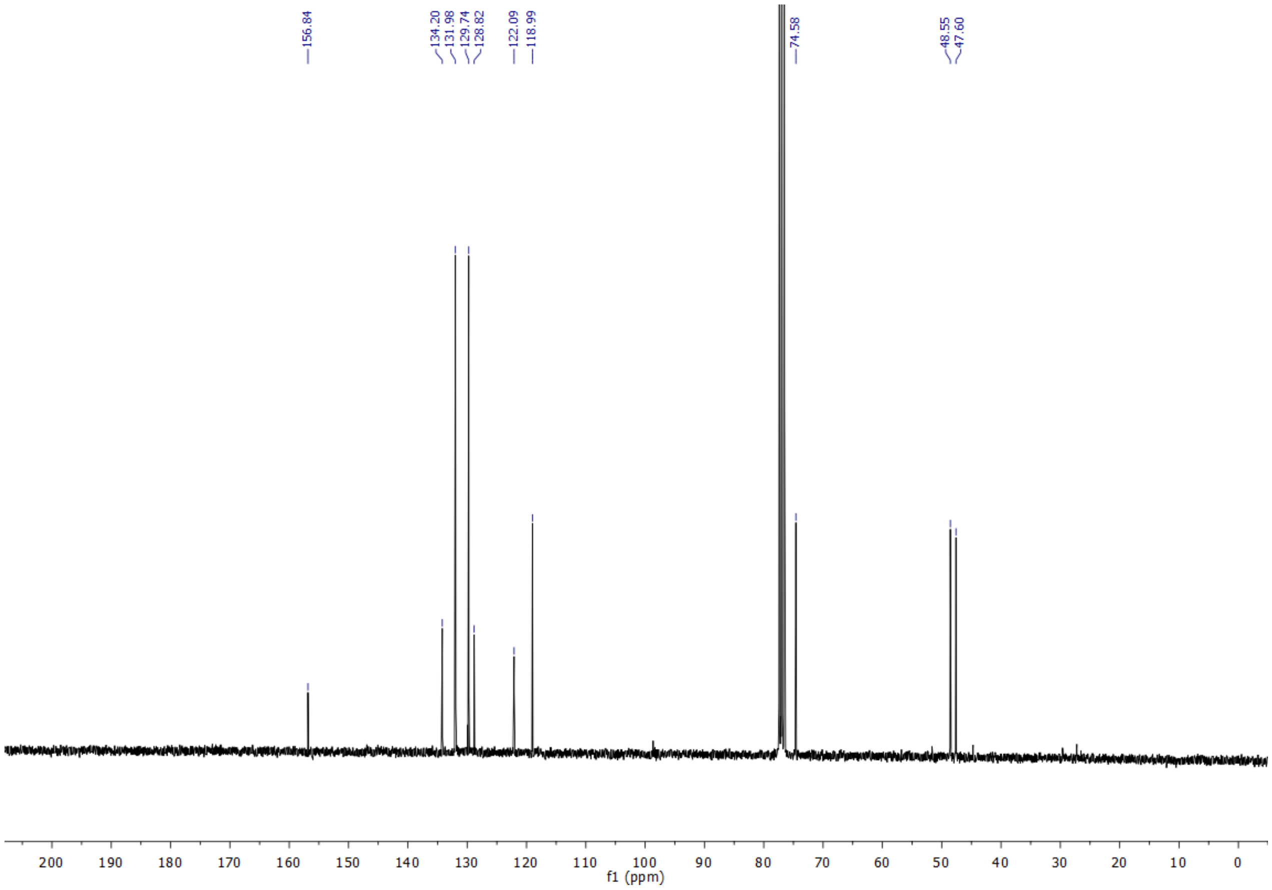

^{1}H NMR (300 MHz) spectrum of **2d** in $CDCl_3$ at 25 ºC

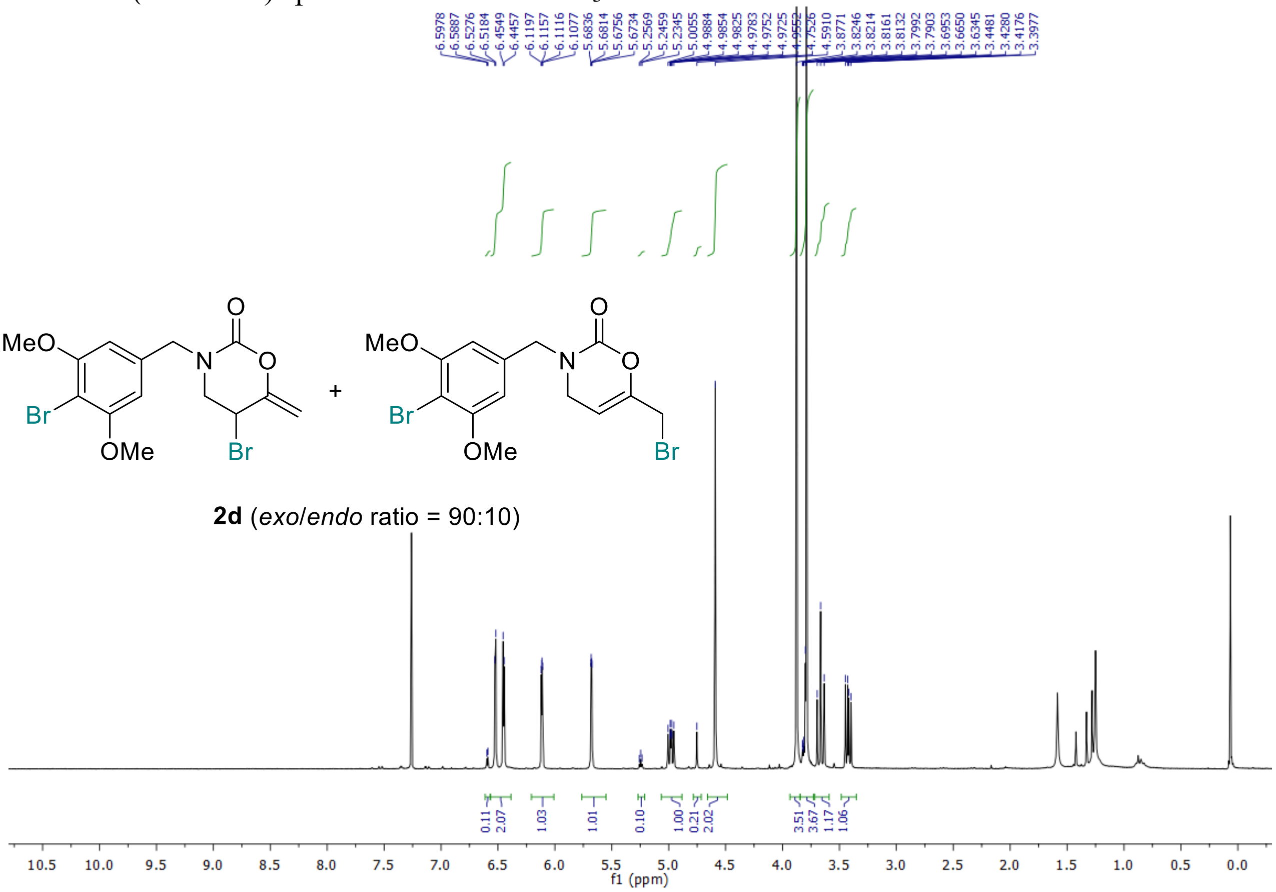

2d (*exo*/*endo* ratio = 90:10)

^{13}C NMR (75 MHz) spectrum of **2d** in $CDCl_3$ at 25 ºC

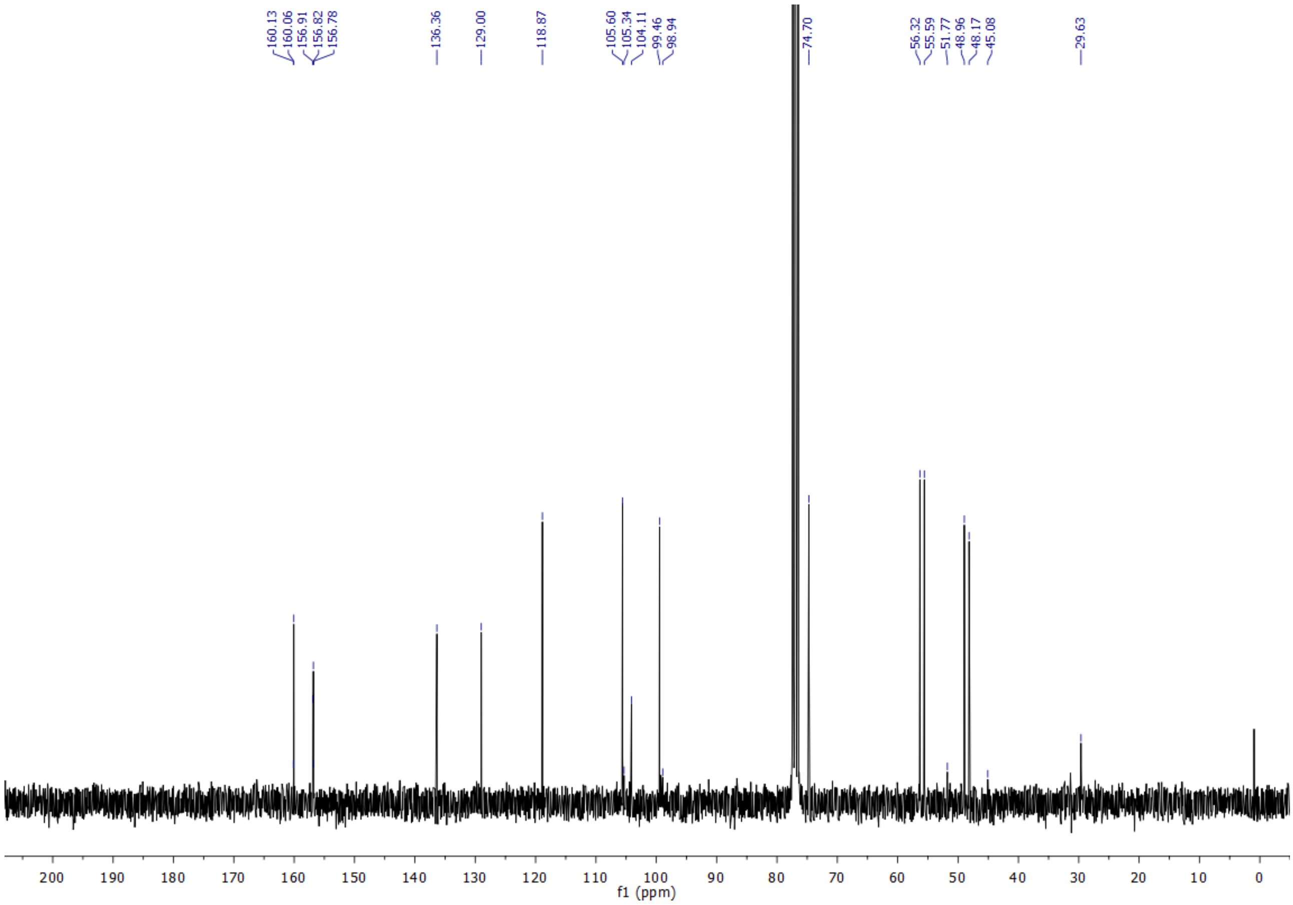

^{1}H NMR (300 MHz) spectrum of **2e** in $CDCl_3$ at 25 ºC

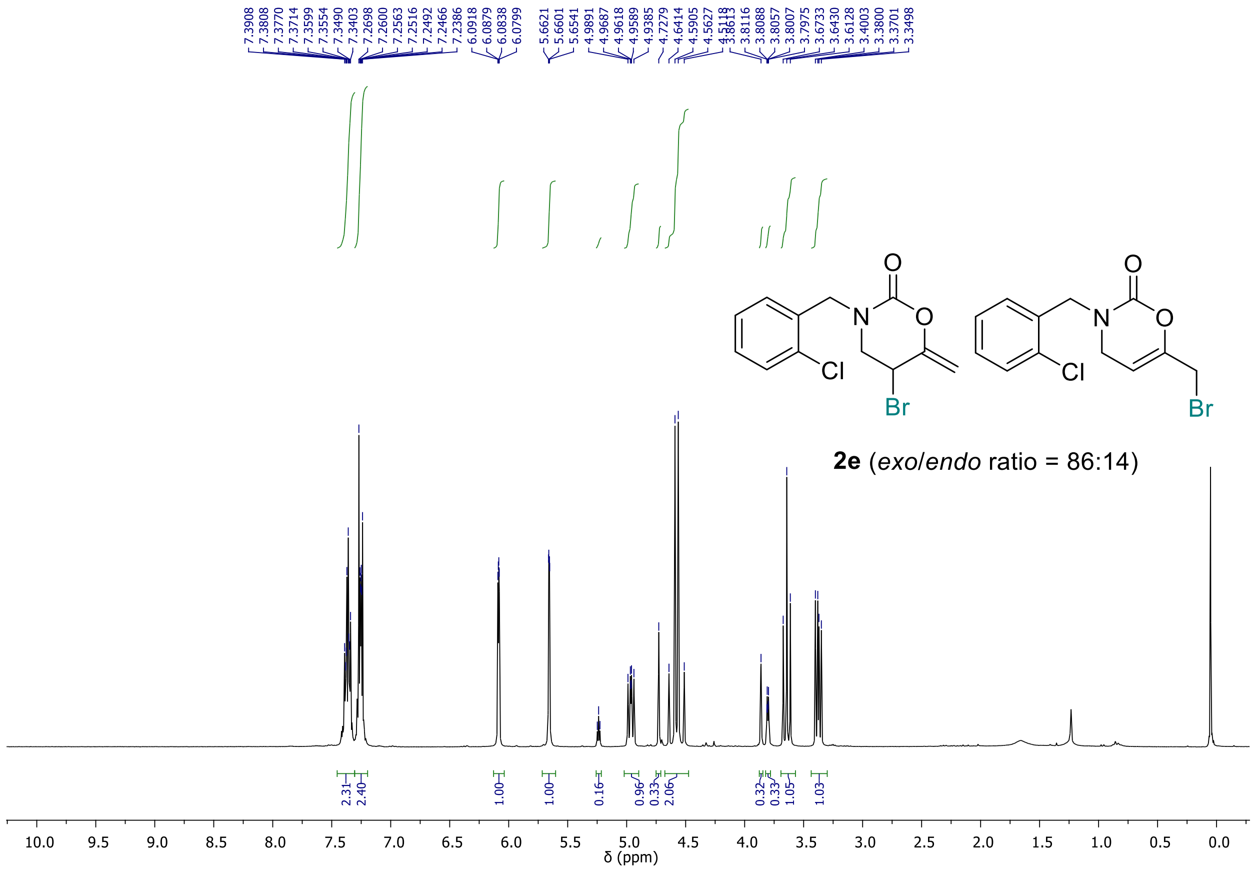

^{13}C NMR (75 MHz) spectrum of **2e** in $CDCl_3$ at 25 ºC

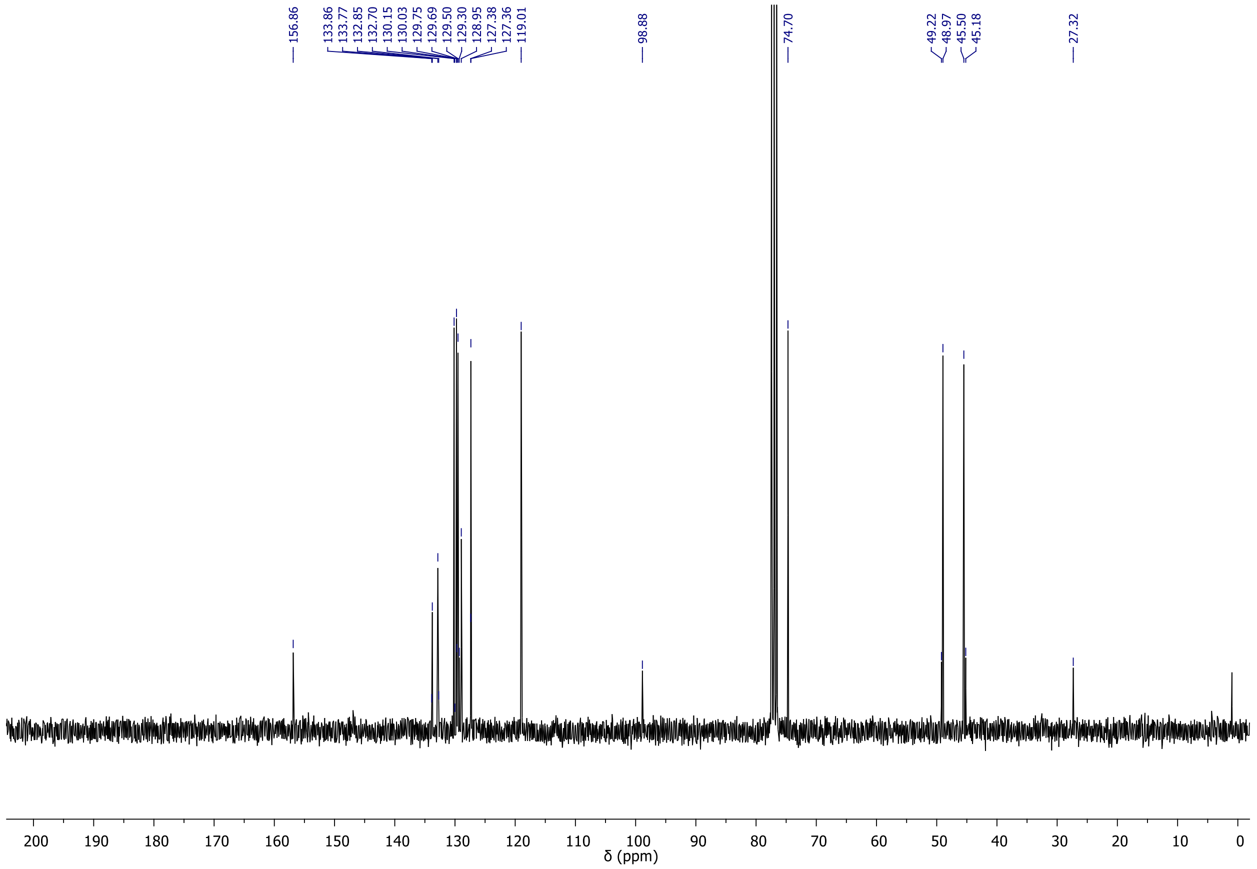

^{1}H NMR (300 MHz) spectrum of **2f** in $CDCl_3$ at 25 ºC

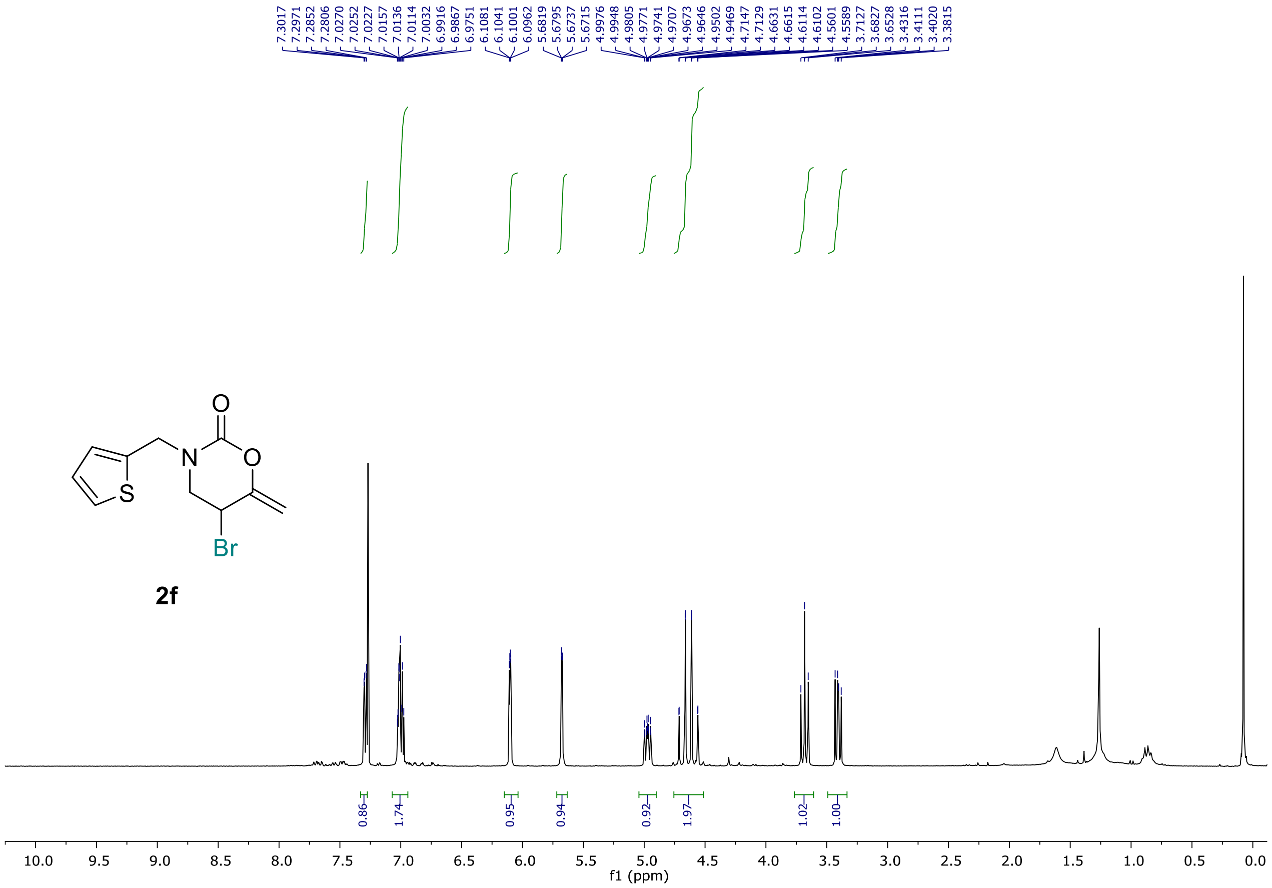

^{13}C NMR (75 MHz) spectrum of **2f** in $CDCl_3$ at 25 ºC

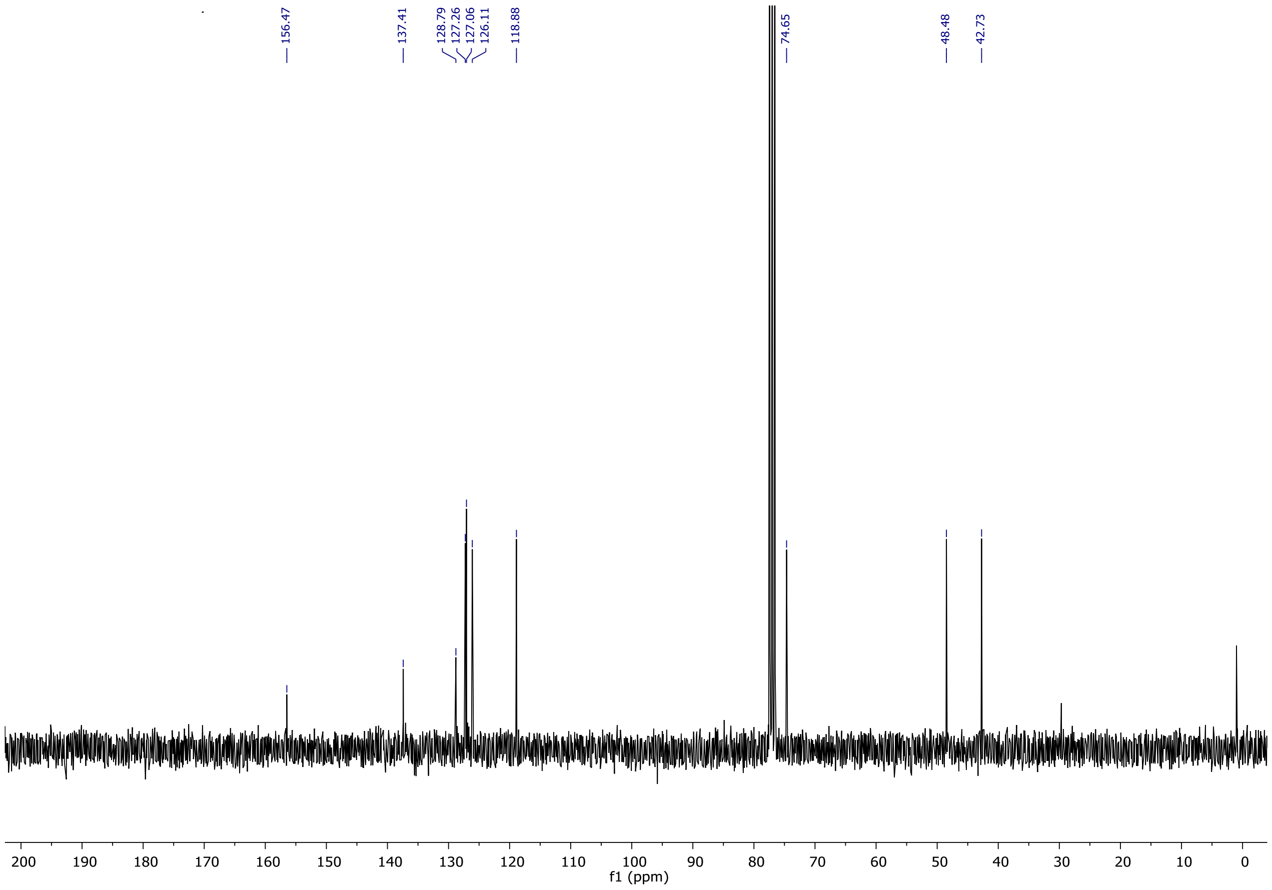

^{1}H NMR (300 MHz) spectrum of **2g** in $CDCl_3$ at 25 ºC

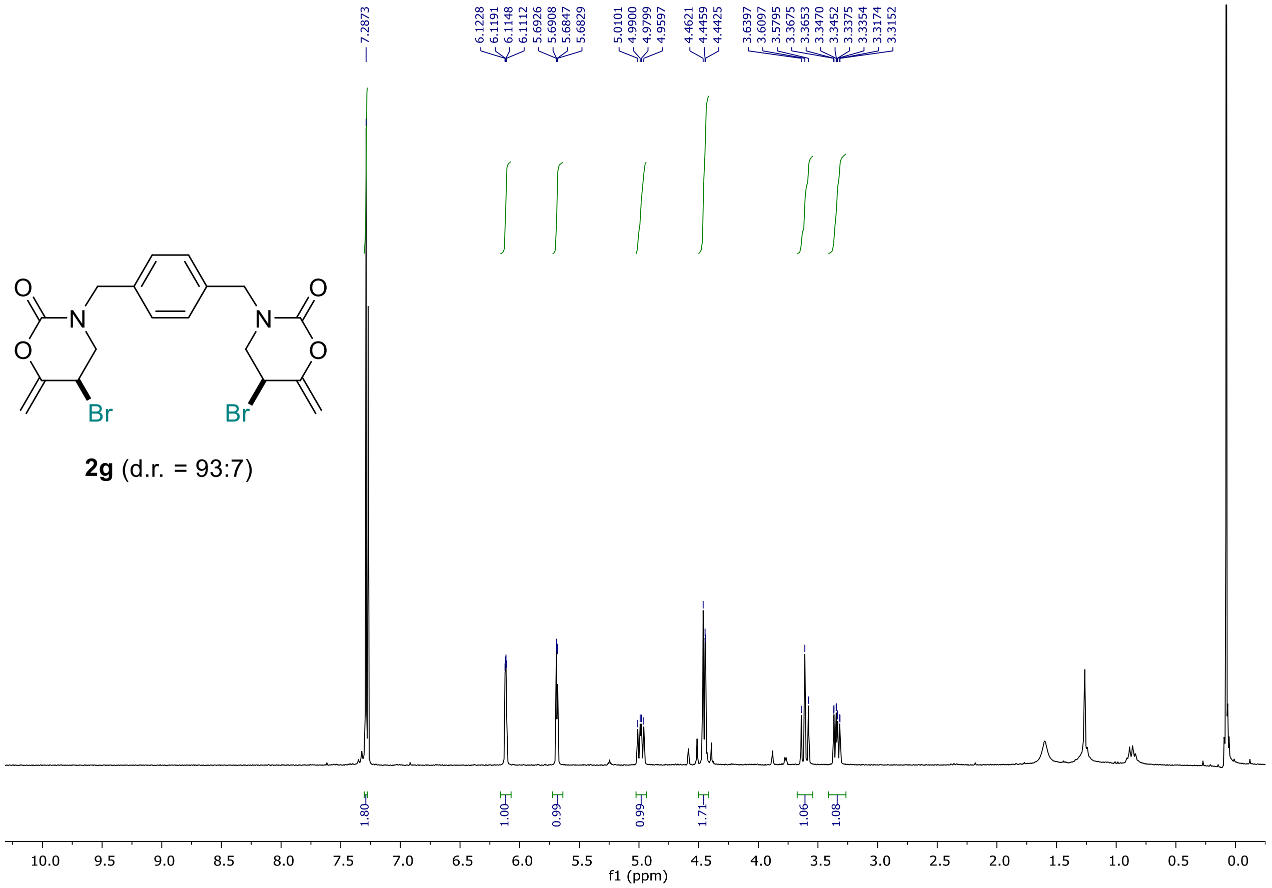

^{13}C NMR (75 MHz) spectrum of **2g** in $CDCl_3$ at 25 ºC

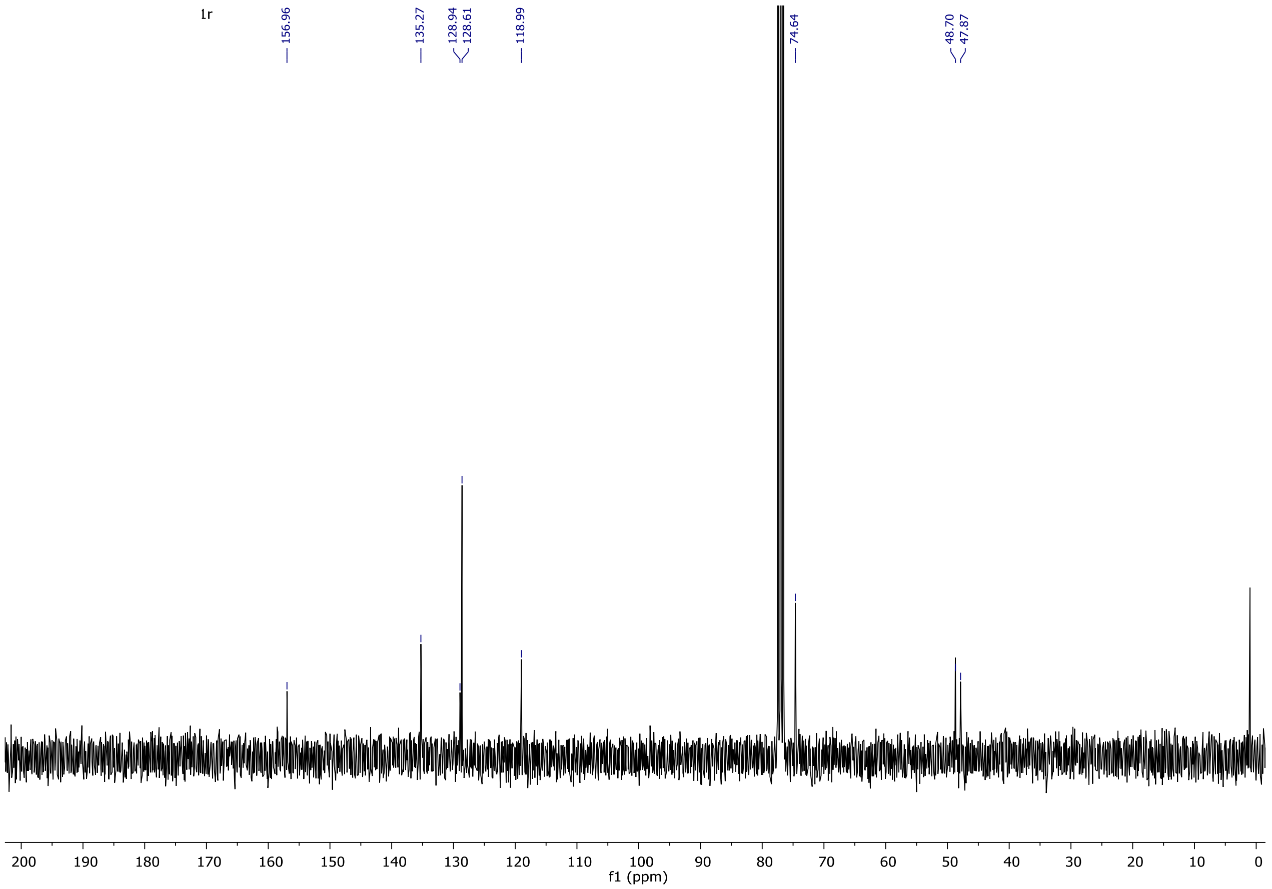

^{1}H NMR (300 MHz) spectrum of **(+)-2h** in $CDCl_3$ at 25 ºC

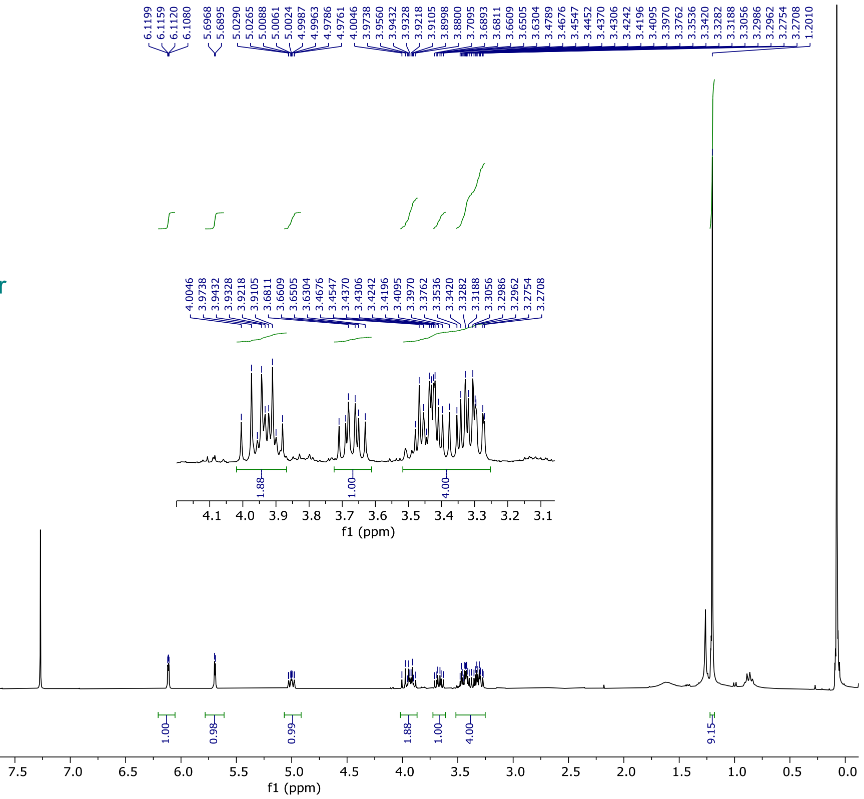

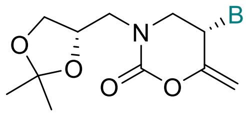

(**+**)-**2h** (d.r. = 96:4)

^{13}C NMR (75 MHz) spectrum of **(+)-2h** in $CDCl_3$ at 25 ºC

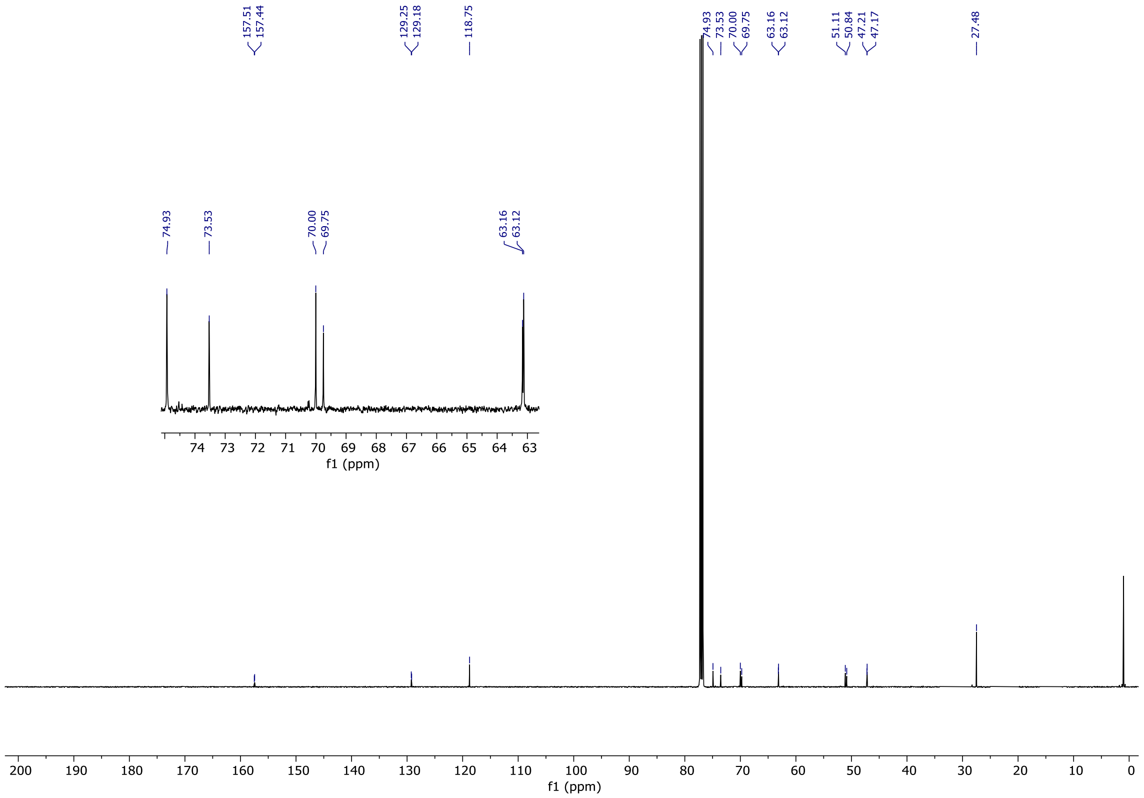

NOE spectrum of **(+)-2h** in $CDCl_3$ at 25 ºC

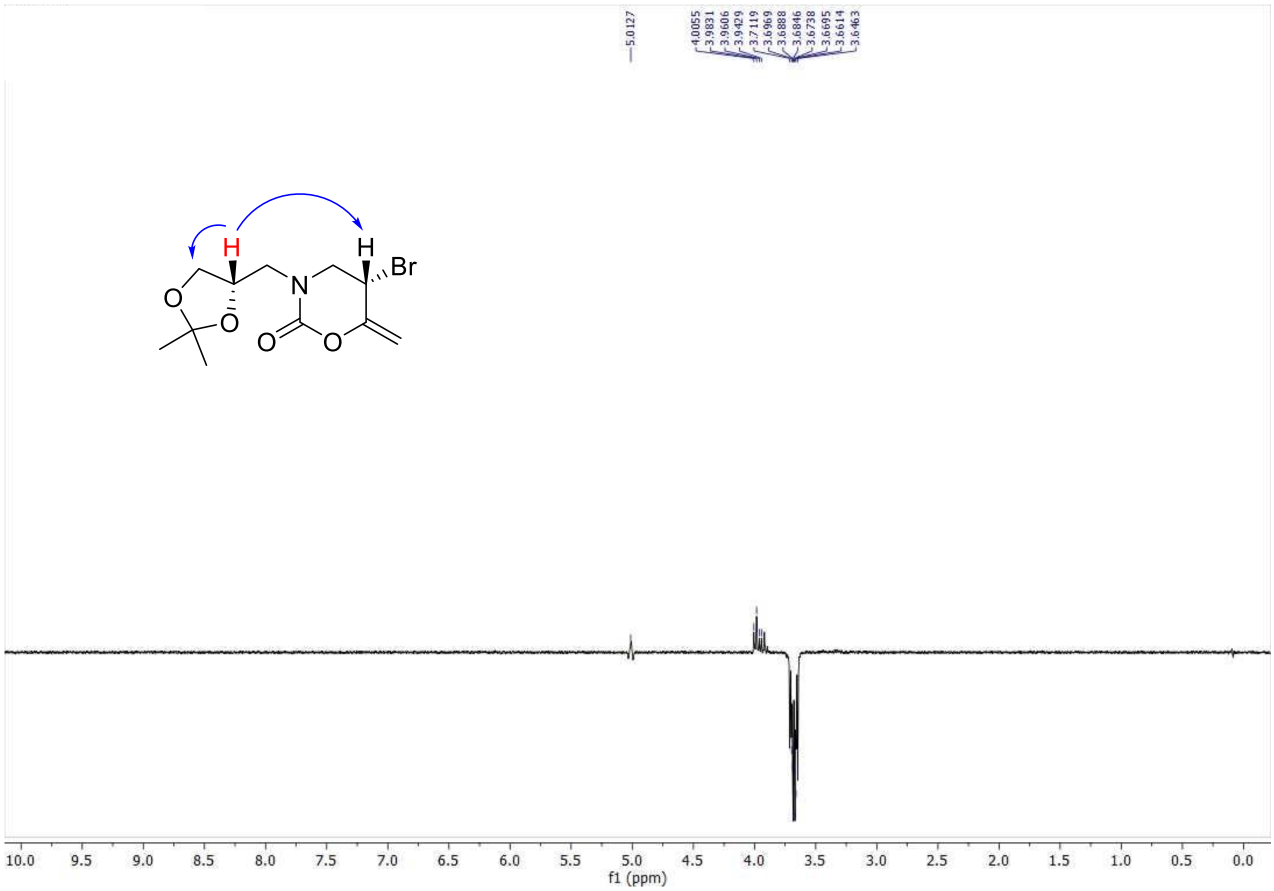

^{1}H NMR (300 MHz) spectrum of **(–)-*anti*-2i** in $CDCl_3$ at 25 ºC

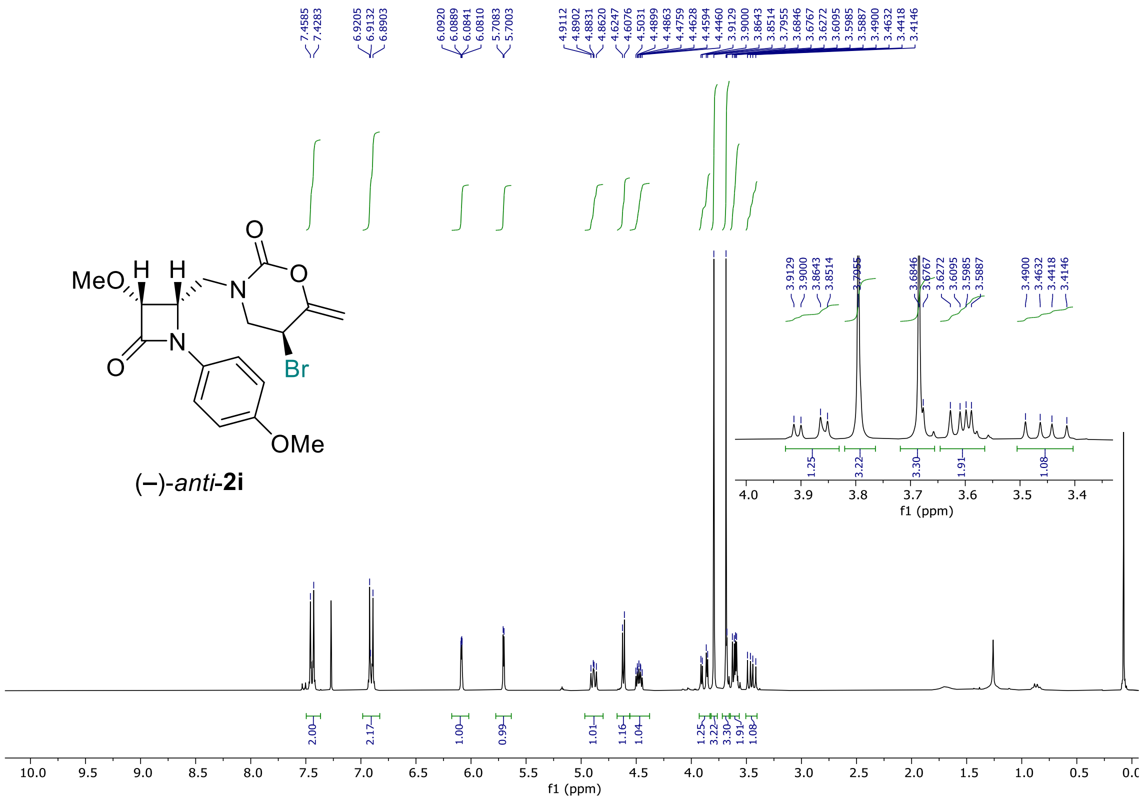

^{13}C NMR (75 MHz) spectrum of **(–)-*anti*-2i** in $CDCl_3$ at 25 ºC

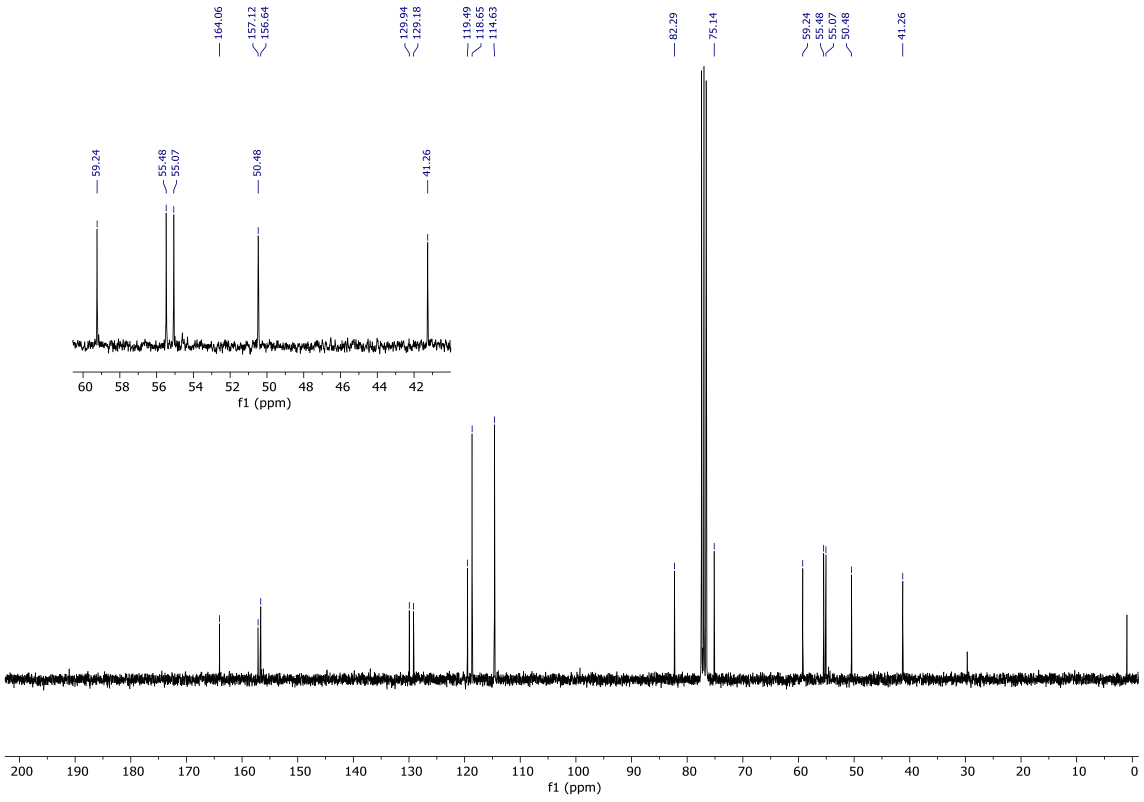

NOE spectrum of **(–)-***anti***-2i** in $CDCl_3$ at 25 ºC

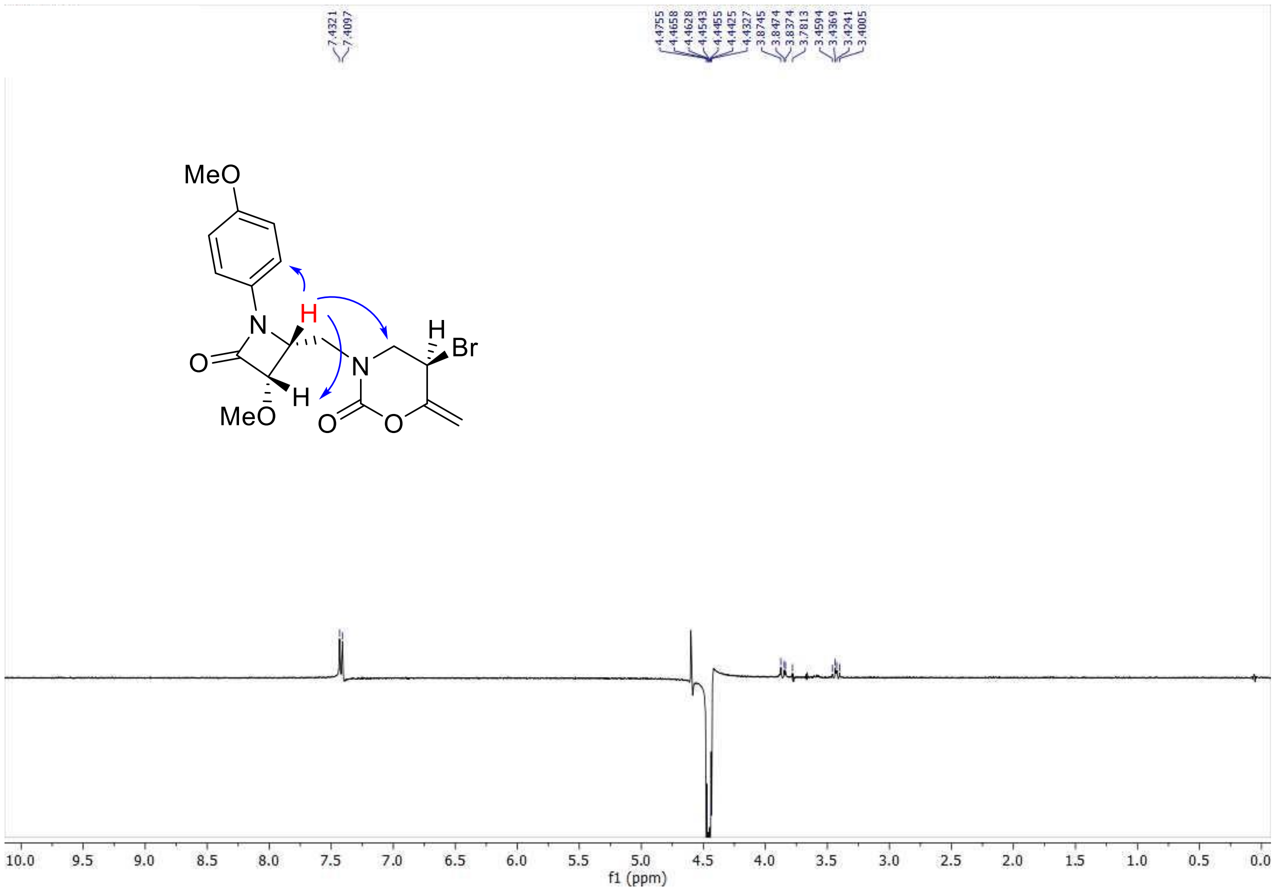

^{1}H NMR (300 MHz) spectrum of **(+)-*syn*-2i** in $CDCl_3$ at 25 ºC

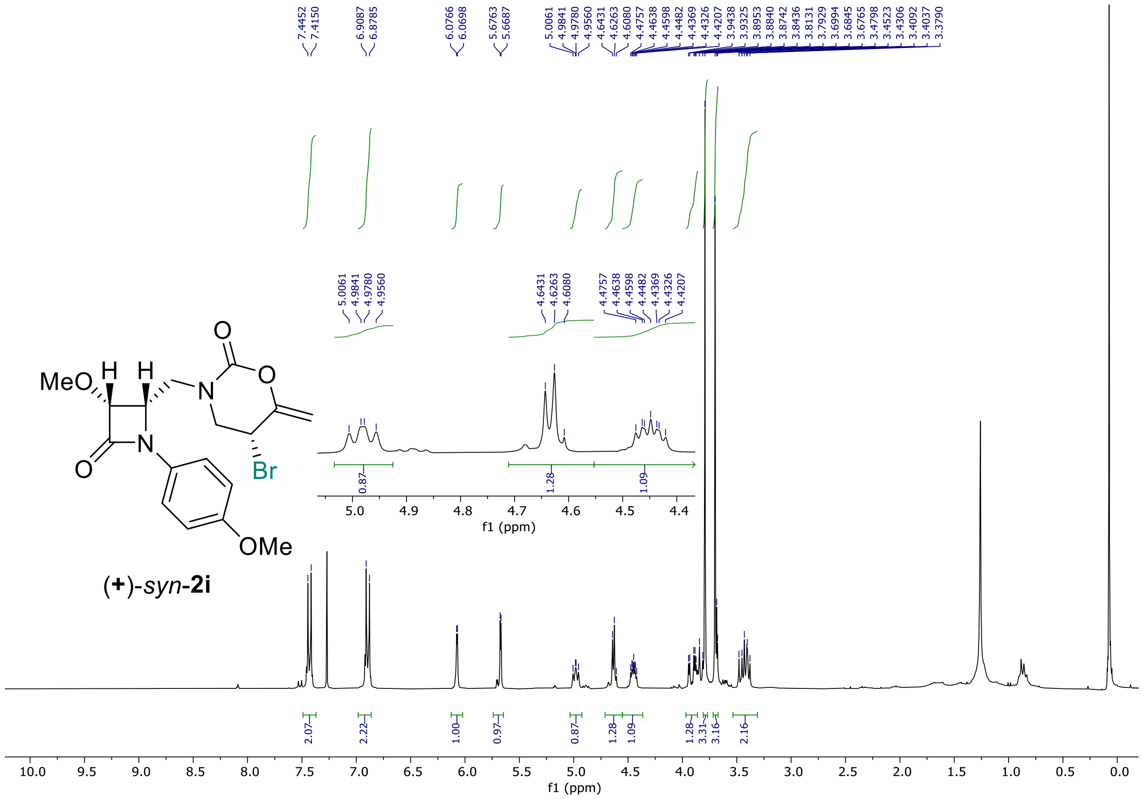

^{13}C NMR (75 MHz) spectrum of **(+)-*syn*-2i** in $CDCl_3$ at 25 ºC

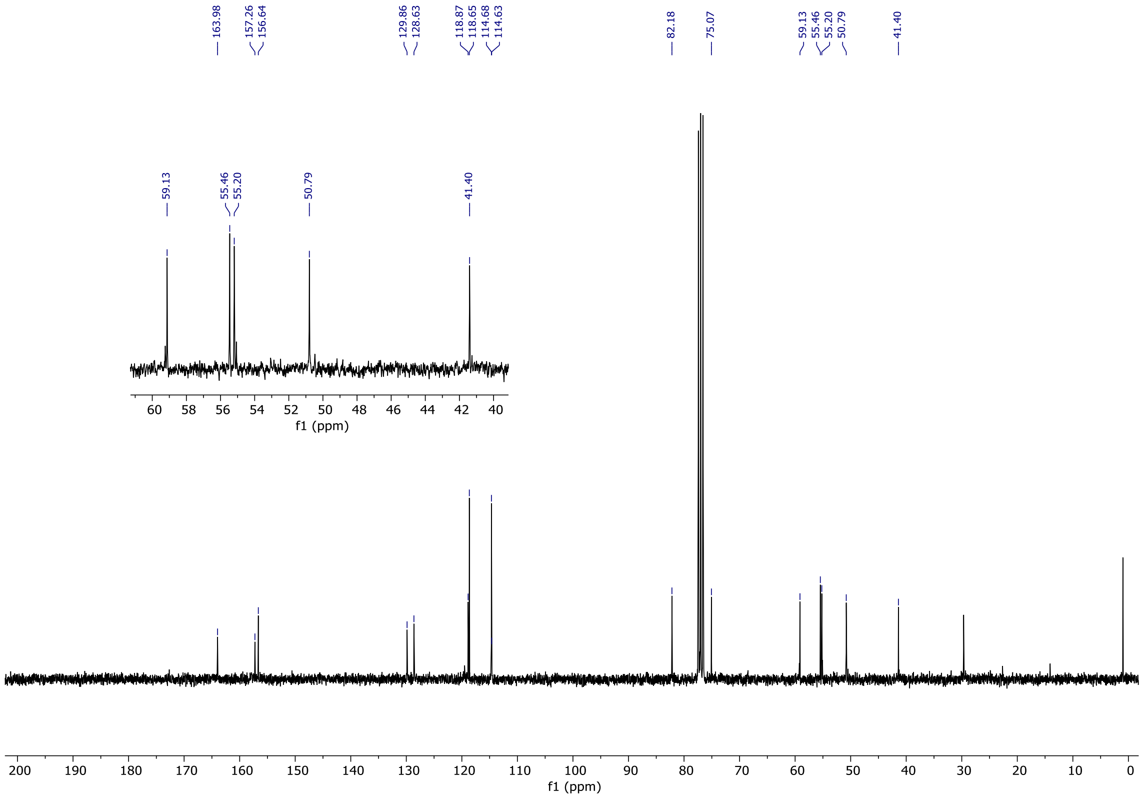

^{1}H NMR (300 MHz) spectrum of **(+)-3j** in $CDCl_3$ at 25 ºC

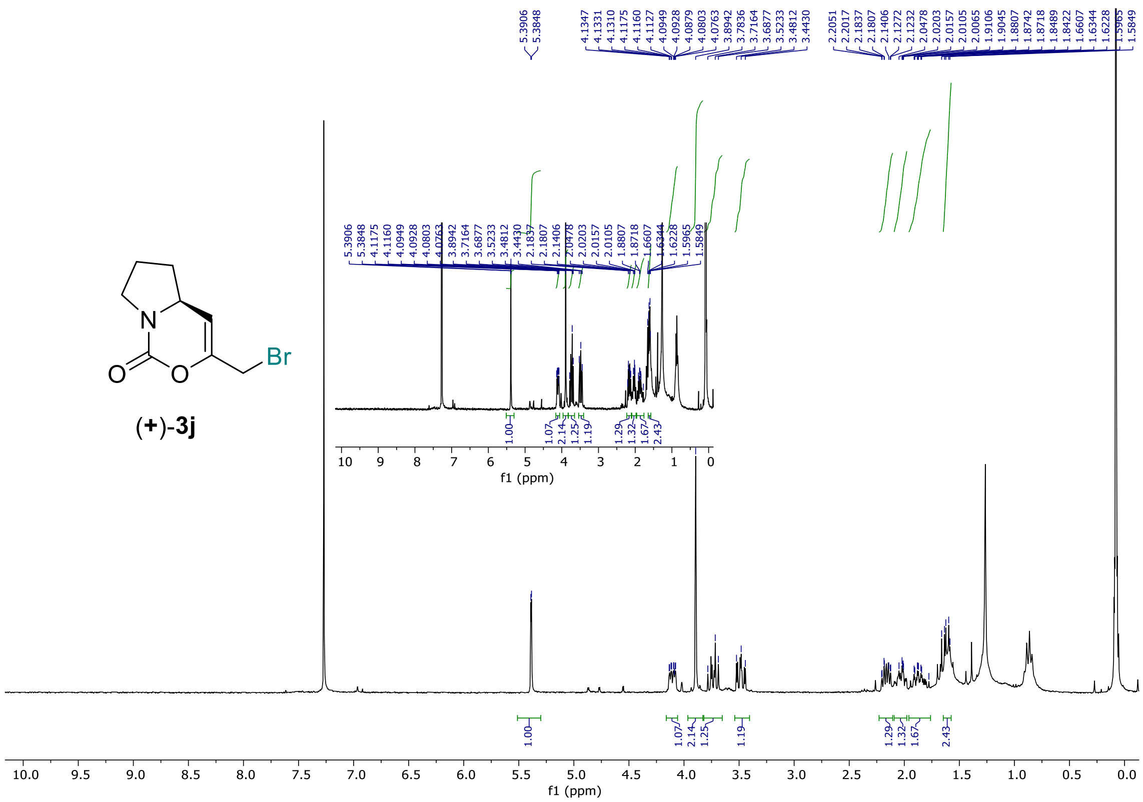

(**+**)-**3j**

^{13}C NMR (75 MHz) spectrum of **(+)-3j** in $CDCl_3$ at 25 ºC

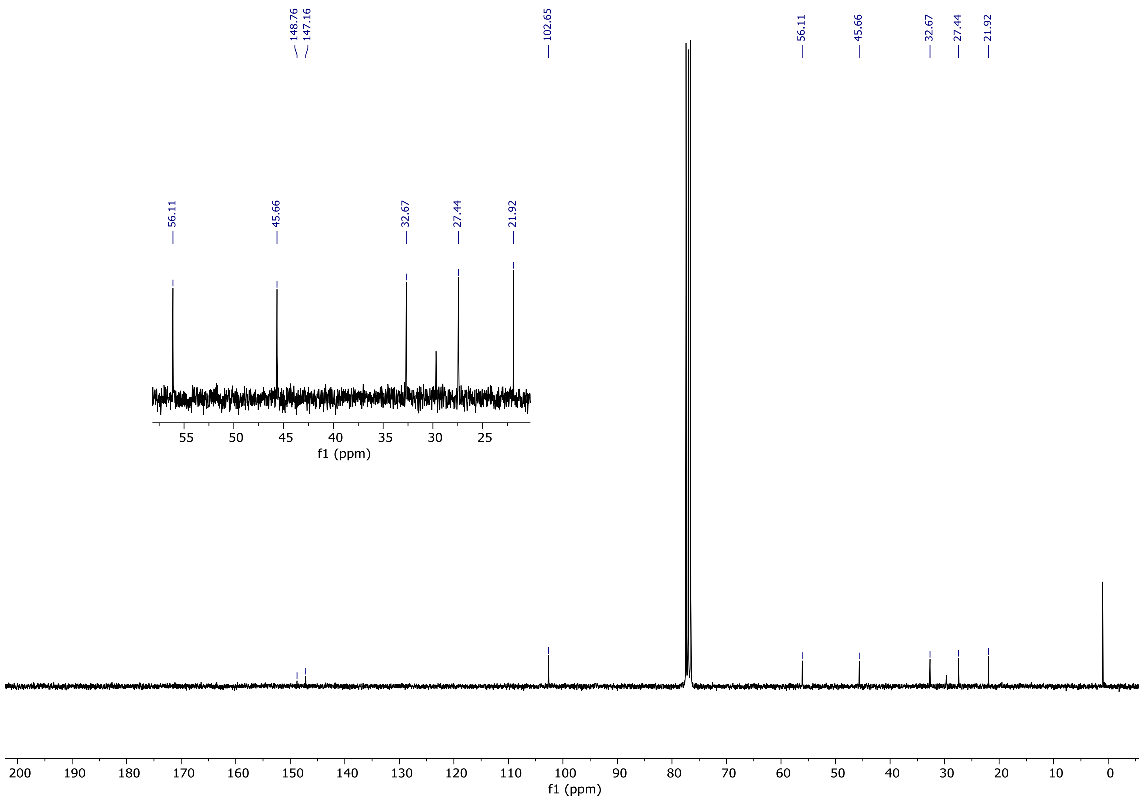

^{1}H NMR (300 MHz) spectrum of **4c** in $CDCl_3$ at 25 ºC

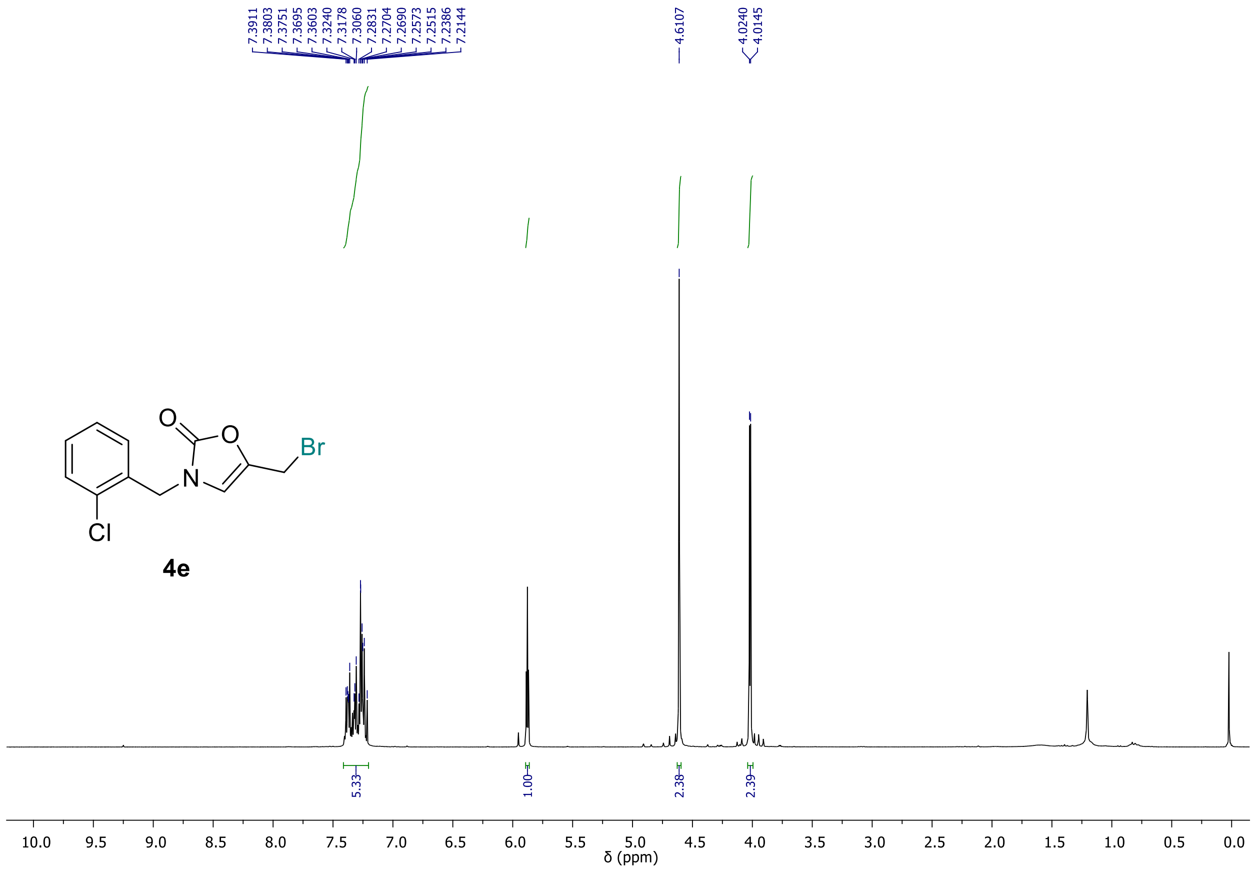

^{13}C NMR (75 MHz) spectrum of **4c** in $CDCl_3$ at 25 ºC

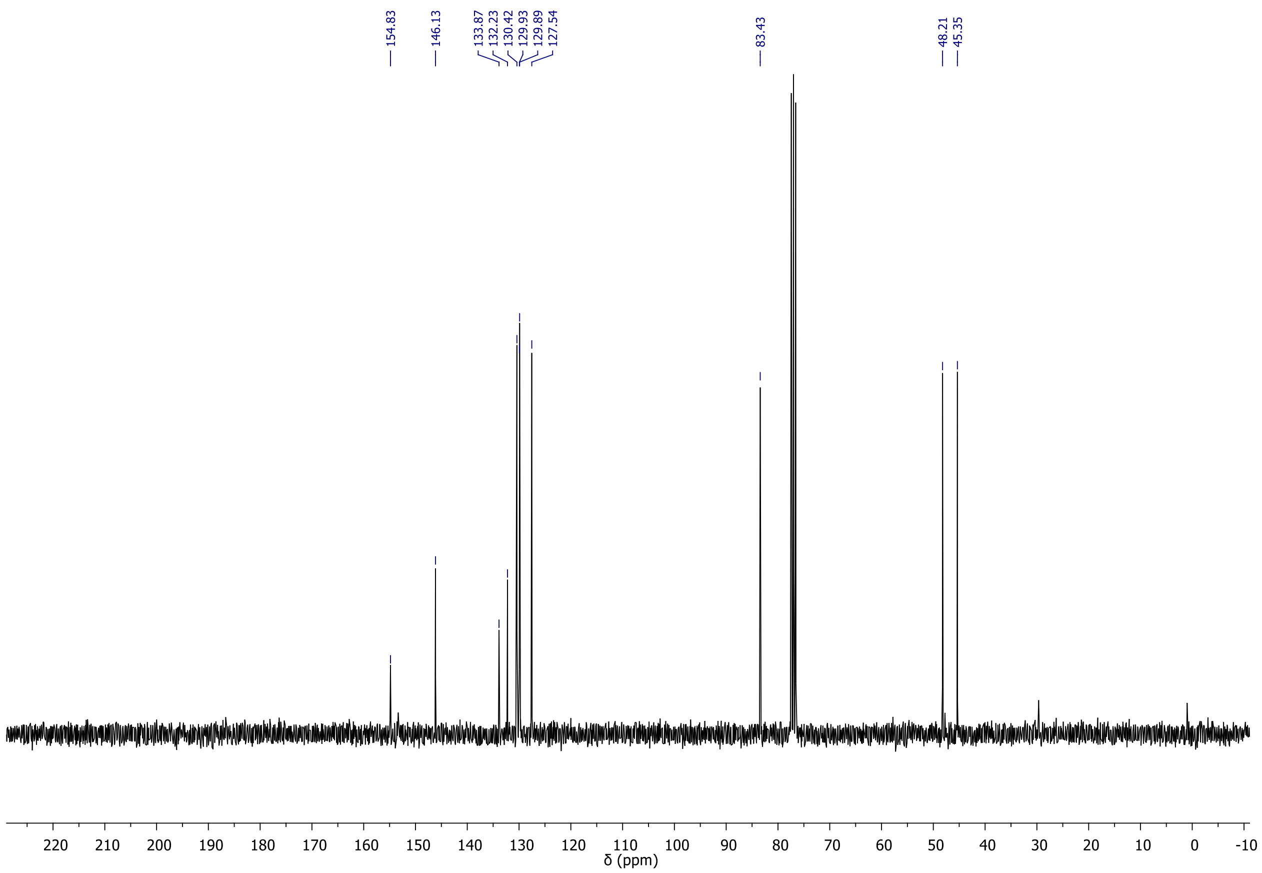

^{1}H NMR (300 MHz) spectrum of the haloheterocyclization reaction of allene **1b** in presence of KI in $CDCl_3$ at 25 ºC

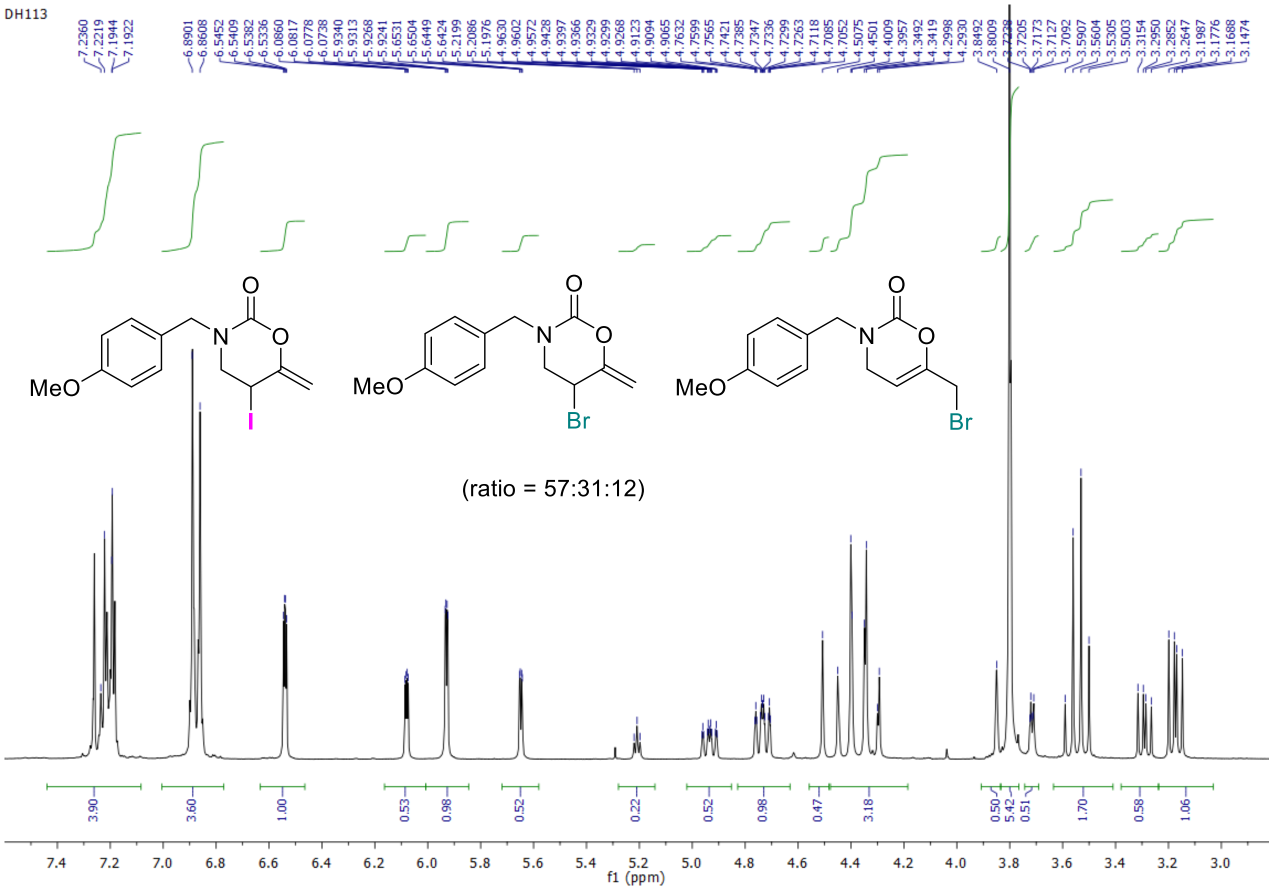

^{1}H NMR (300 MHz) spectrum of **6aa** in $C_2D_2Cl_4$ at 65 ºC

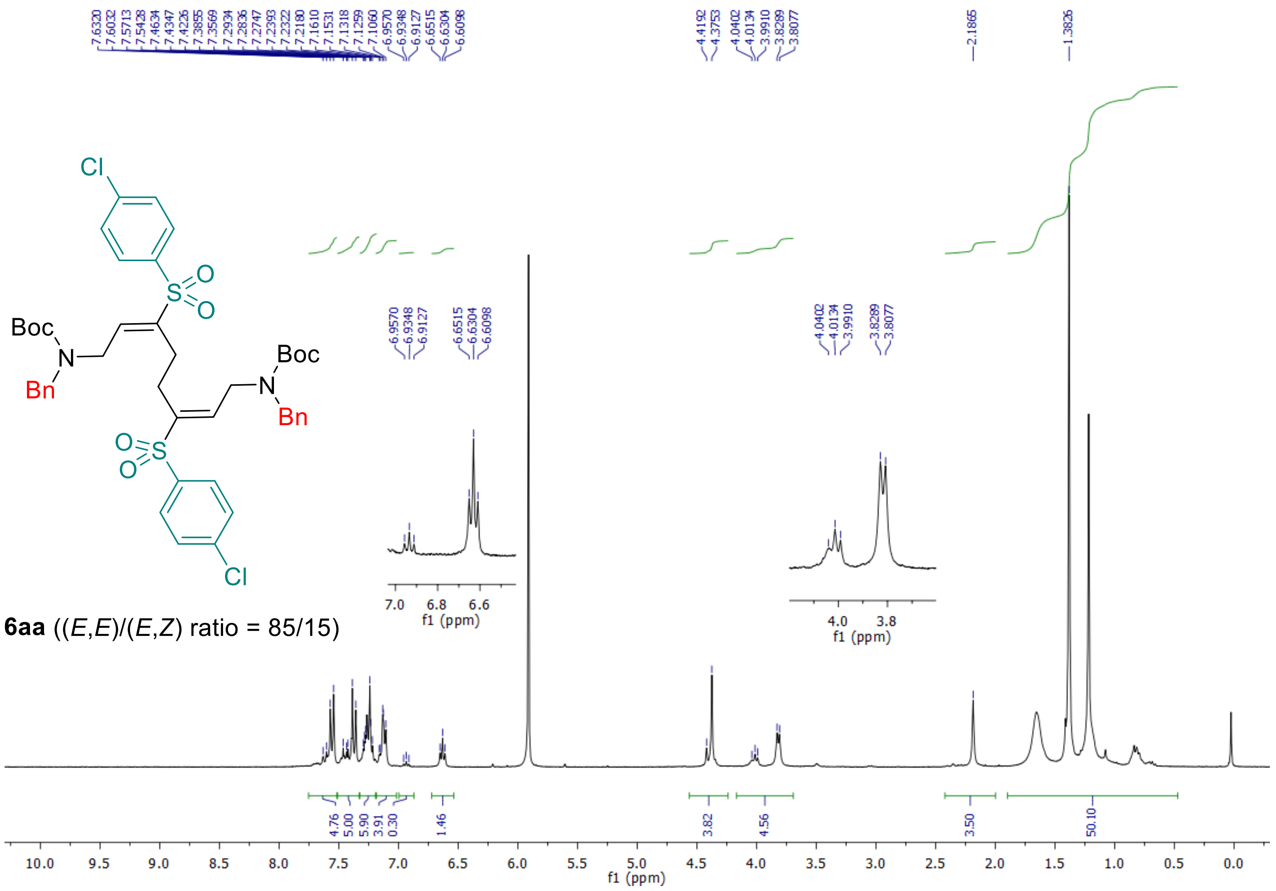

^{13}C NMR (75 MHz) spectrum of **6aa** in $C_2D_2Cl_4$ at 65 ºC

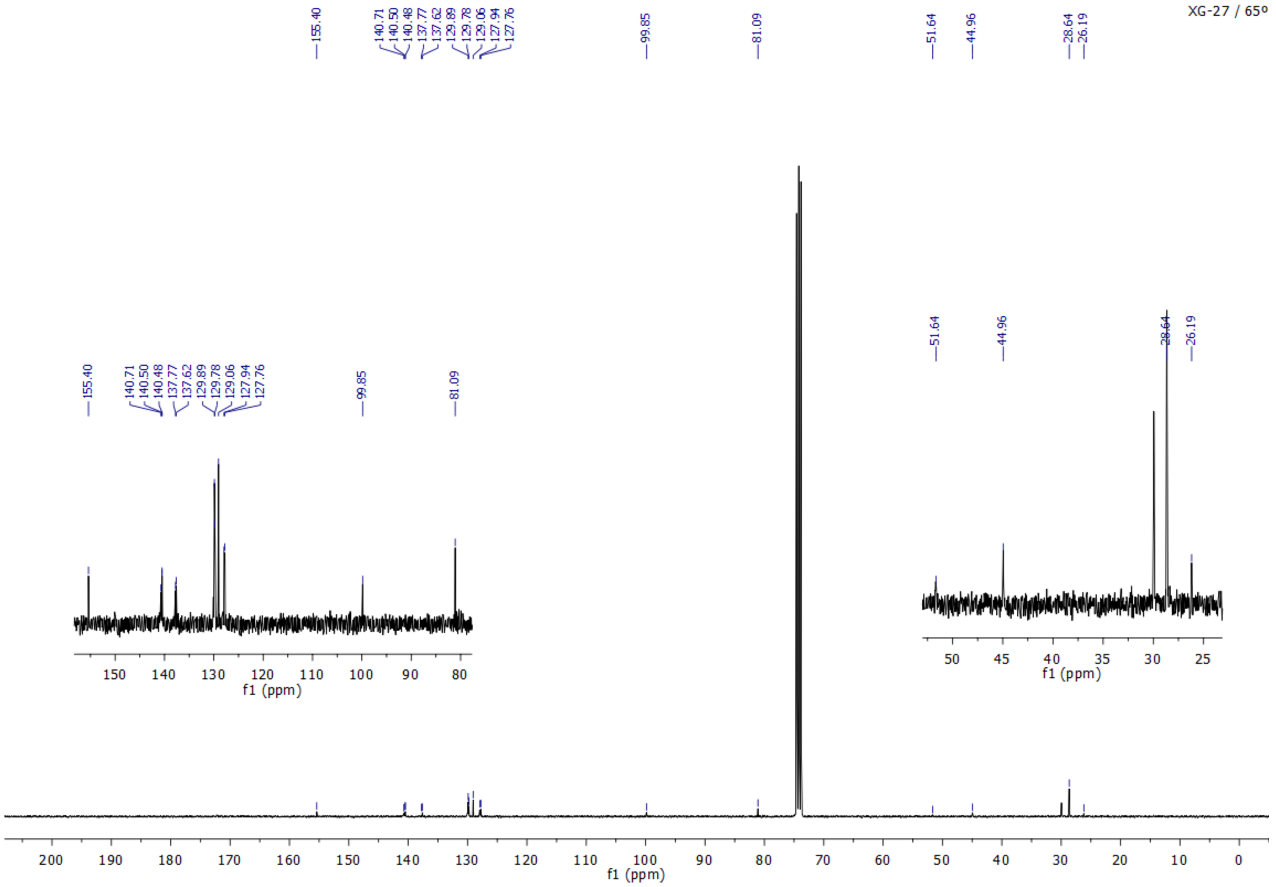

^{1}H NMR (300 MHz) spectrum of **6ba** in $CDCl_3$ at 25 ºC

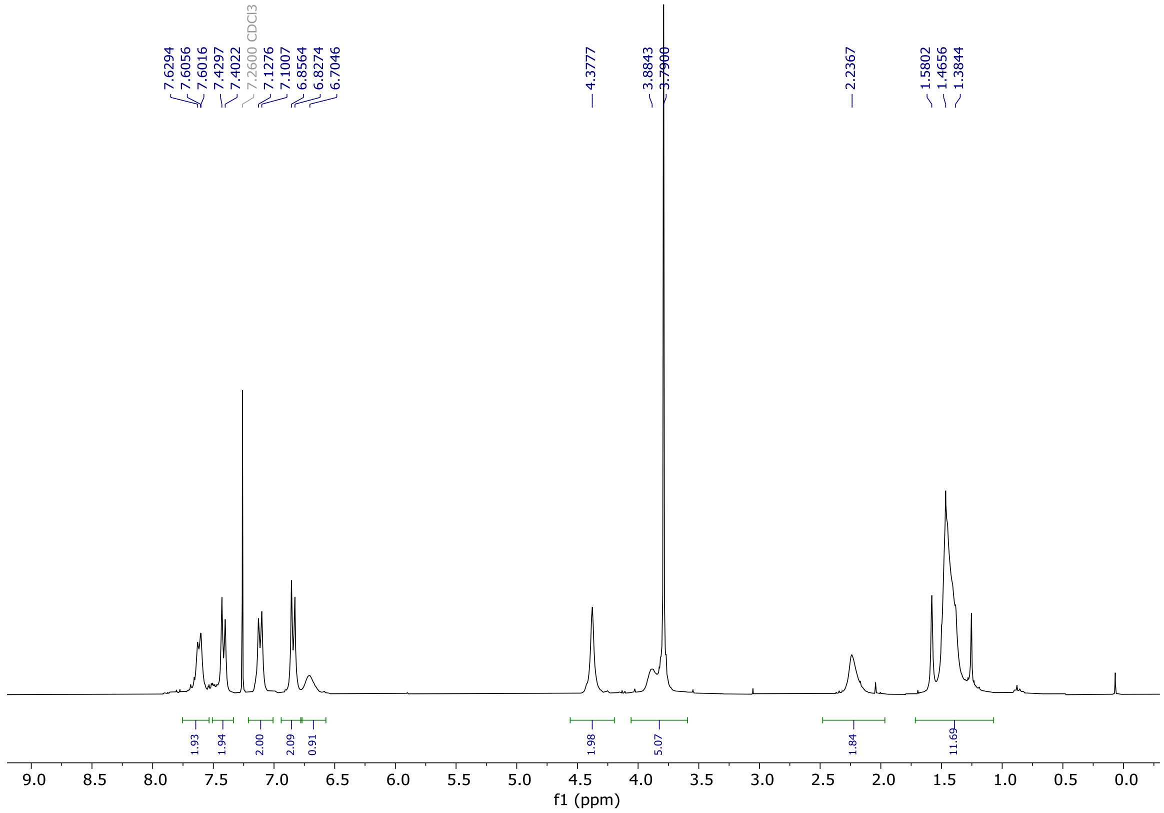

The structure of **6ba** was studied through variable-temperature ^{1}H NMR (300 MHz) spectroscopy at 25, 55, 65, and 75 °C in $C_2D_2Cl_4$. The higher resolution of the ^{1}H NMR spectrum at T>55 °C compared to 25 °C is clearly visible. The multiplet (6.61 ppm), well-resolved signal set in C_2D_2Cl (T>55ºC) indicates the presence of a double bond. This spectrum confirms a dimeric compound.

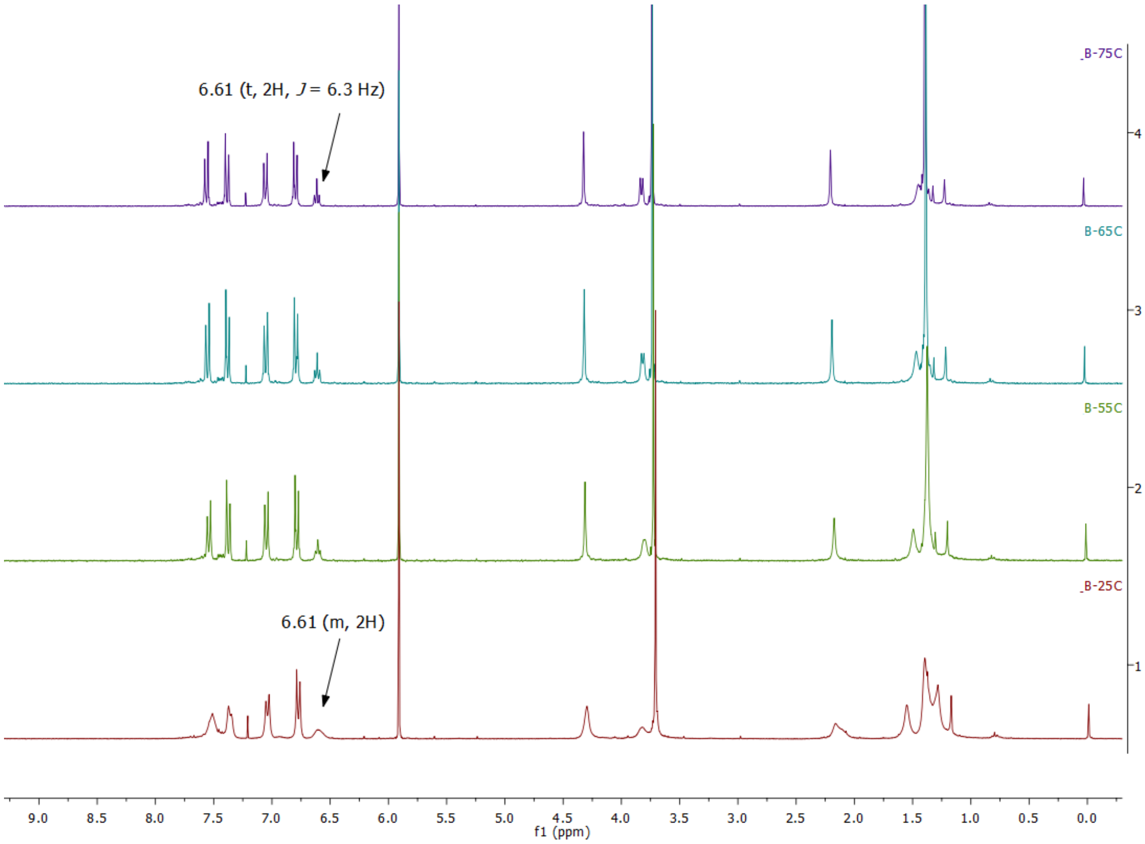

^{1}H NMR (300 MHz) spectrum of **6ba** in $C_2D_2Cl_4$ at 75 ºC

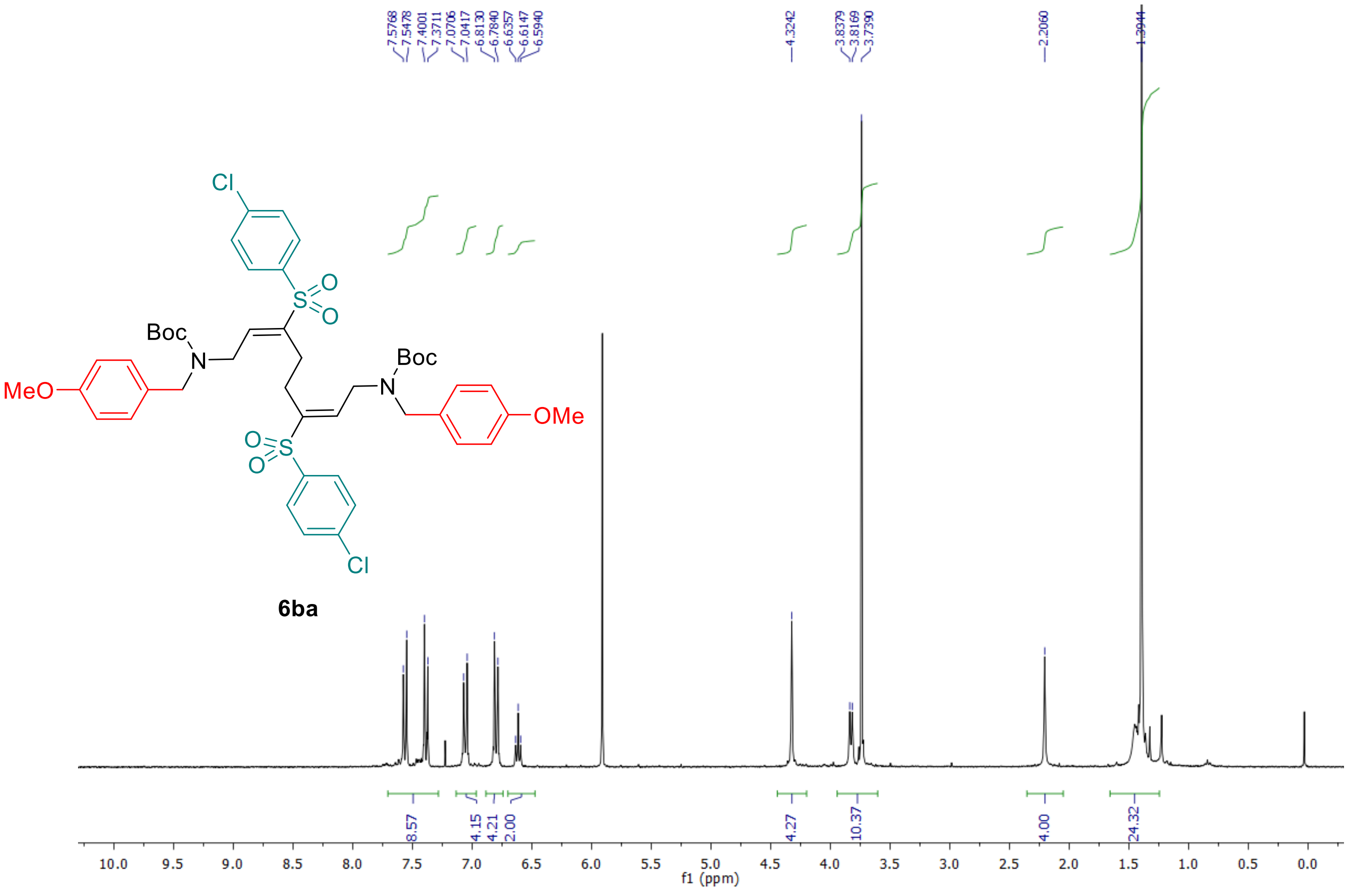

^{13}C NMR (75 MHz) spectrum of **6ba** in $C_2D_2Cl_4$ at 55 ºC

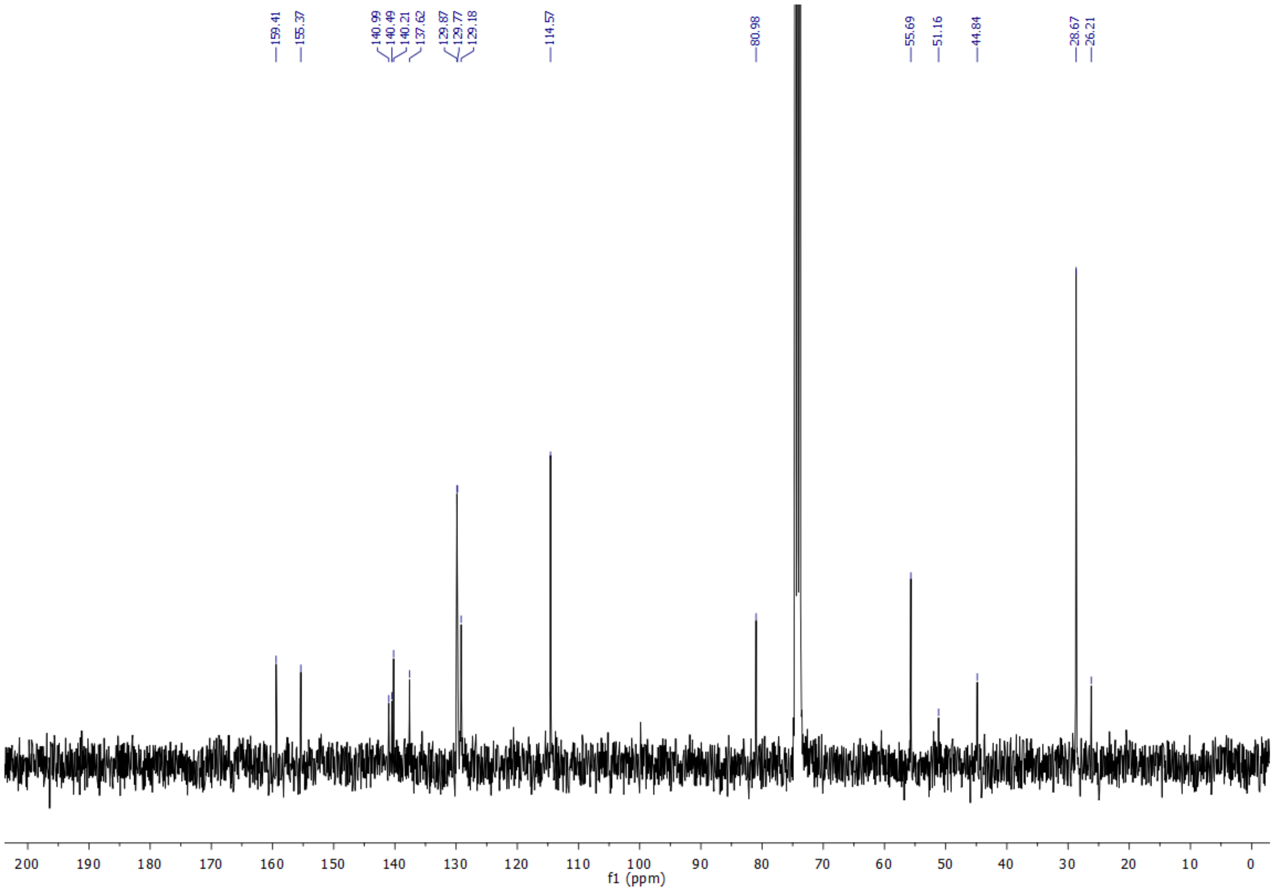

DEPT NMR (75 MHz) spectrum of **6ba** in $C_2D_2Cl_4$ at 55 ºC

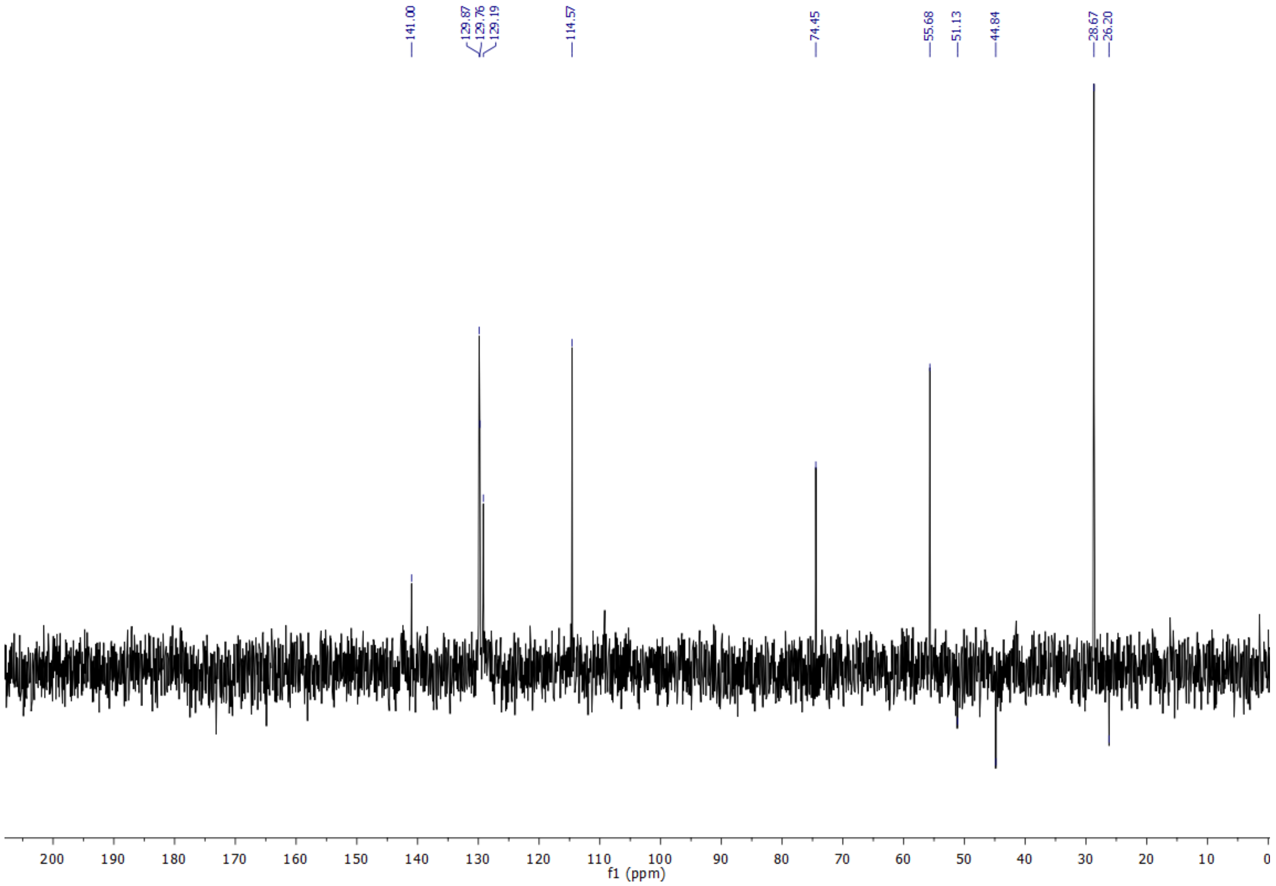

Two-dimensional HMQC NMR spectrum ^{1}H-^{13}C of **6ba** in $C_2D_2Cl_4$ at 55 ºC

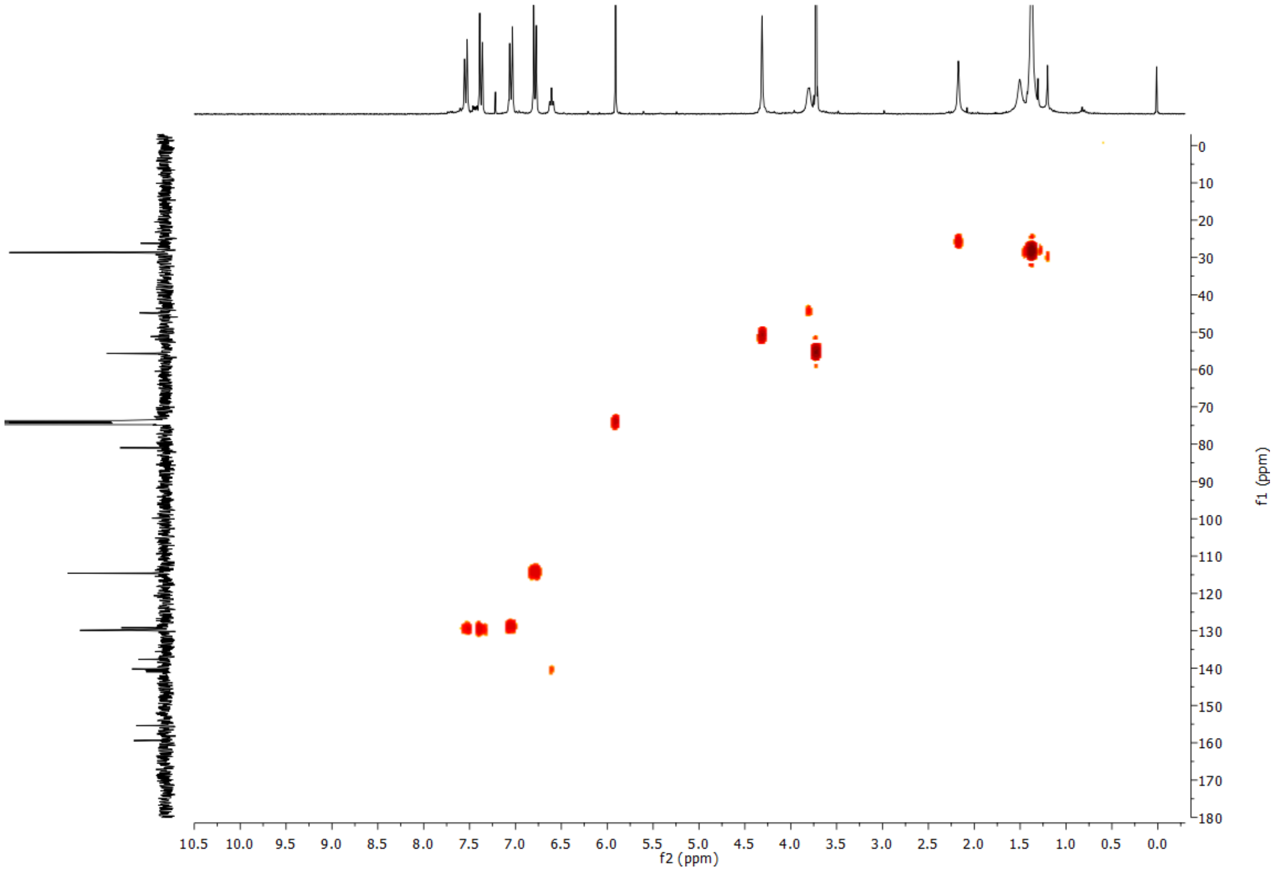

Two-dimensional HMBC NMR spectrum ^{1}H-^{13}C of **6ba** in $C_2D_2Cl_4$ at 55 ºC

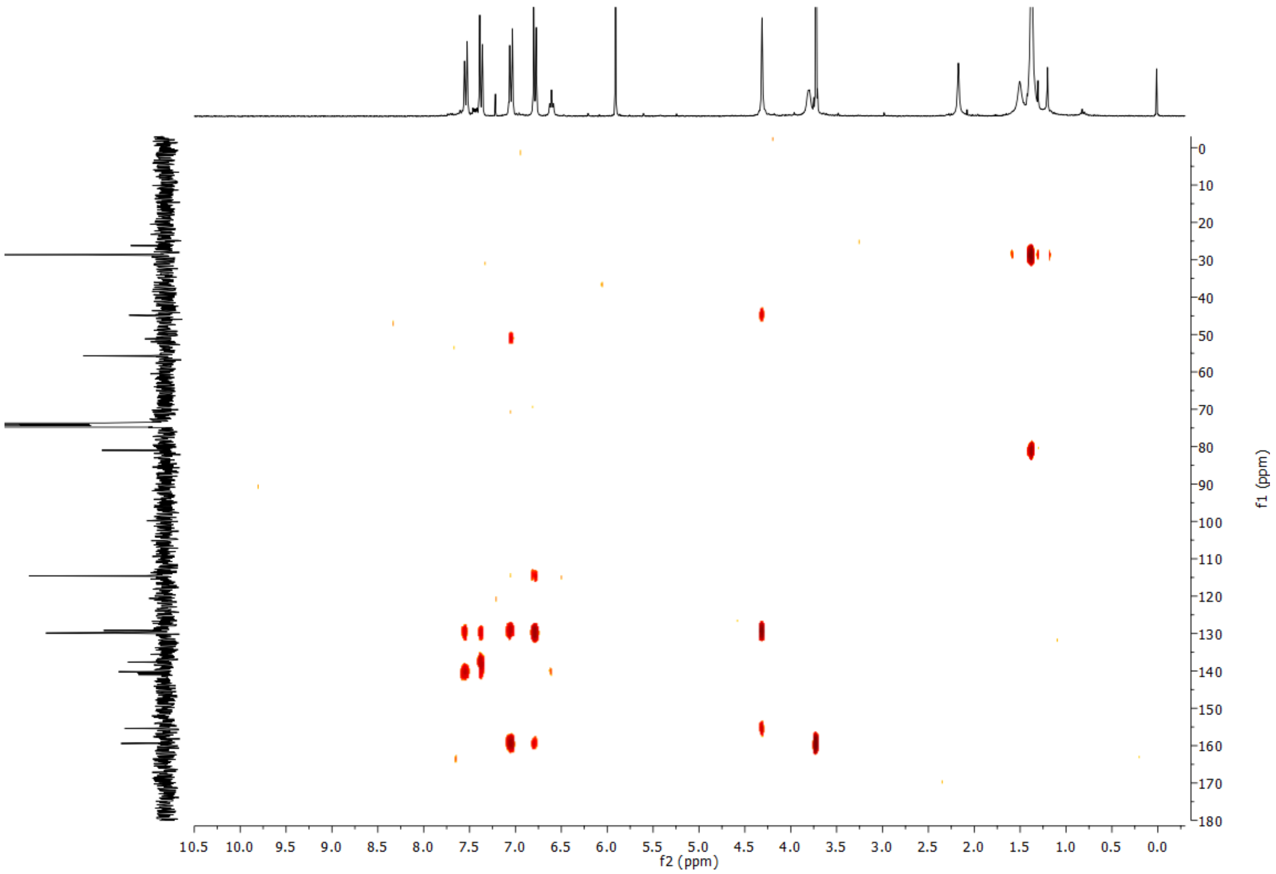

ROESY experiments of **6ba** in $C_2D_2Cl_4$ at 55 ºC

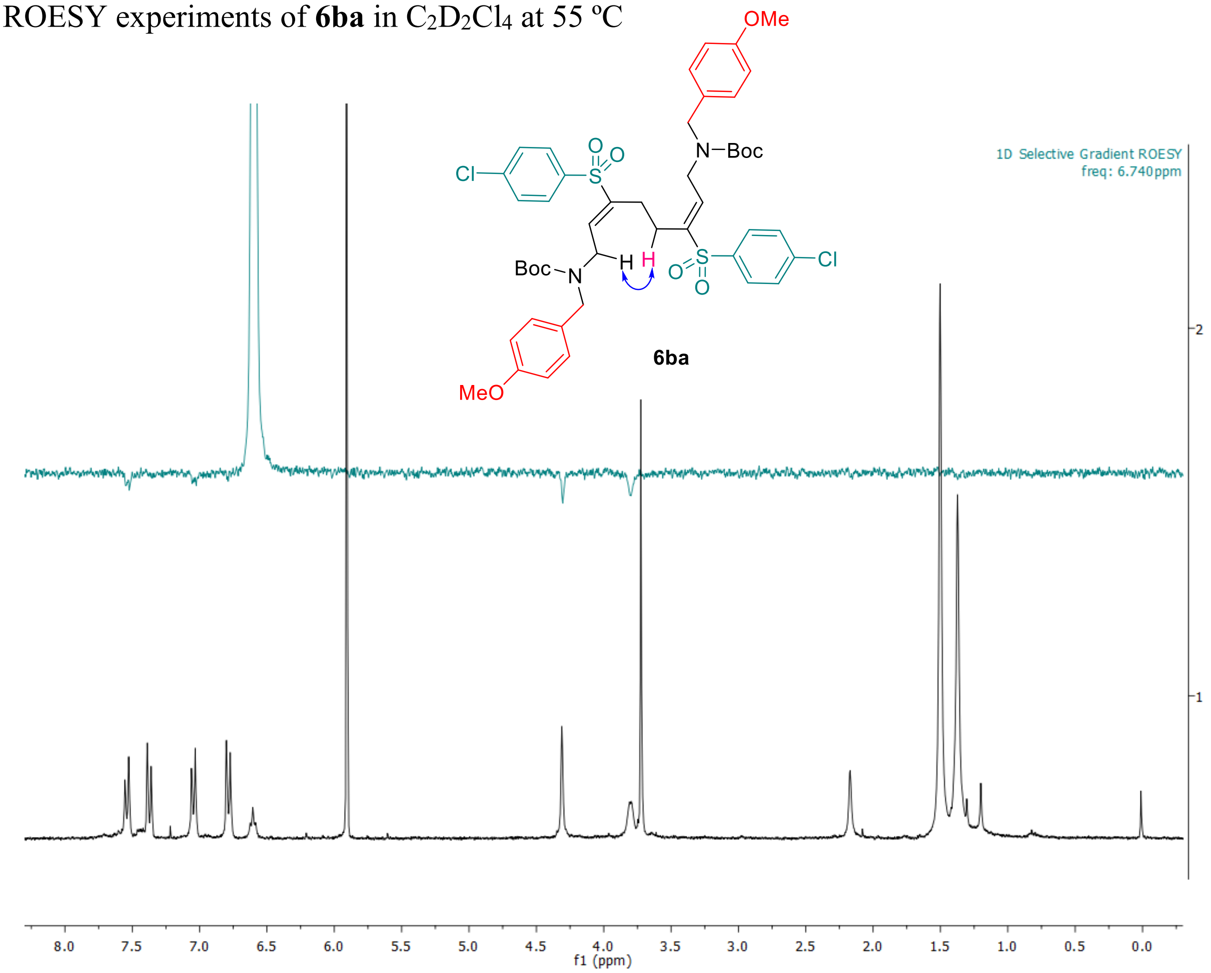

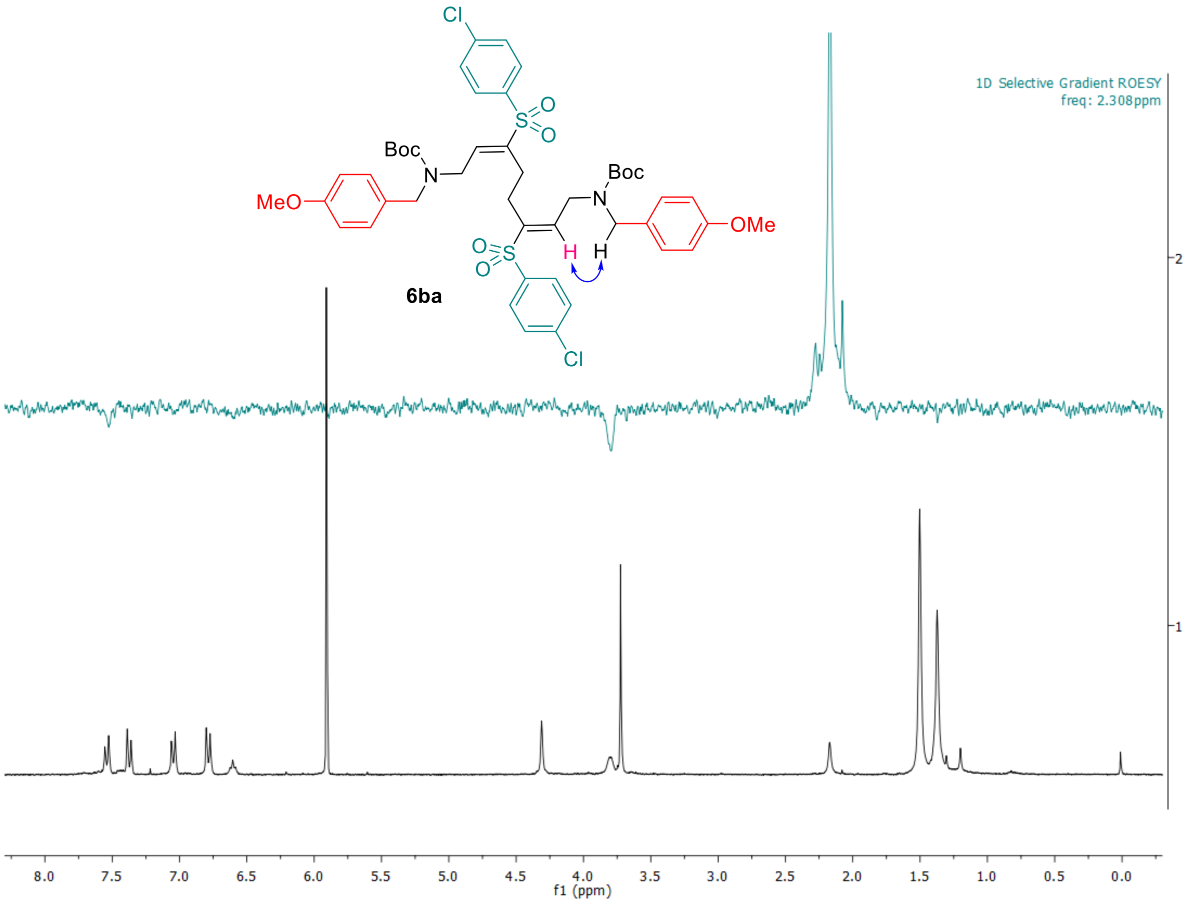

^{1}H NMR (300 MHz) spectrum of **6bb** in $C_2D_2Cl_4$ at 65 ºC

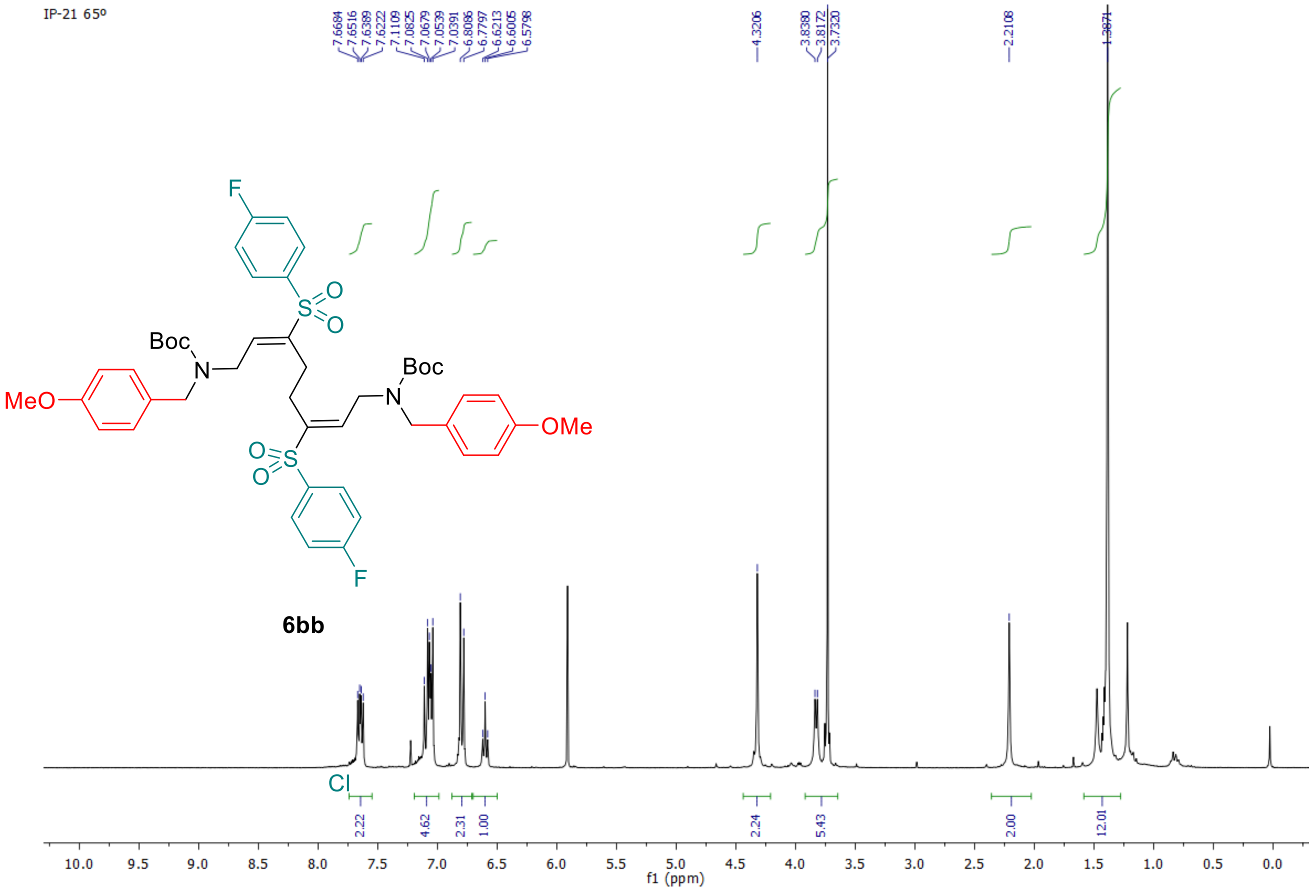

^{13}C NMR (75 MHz) spectrum of **6bb** in $C_2D_2Cl_4$ at 65 ºC

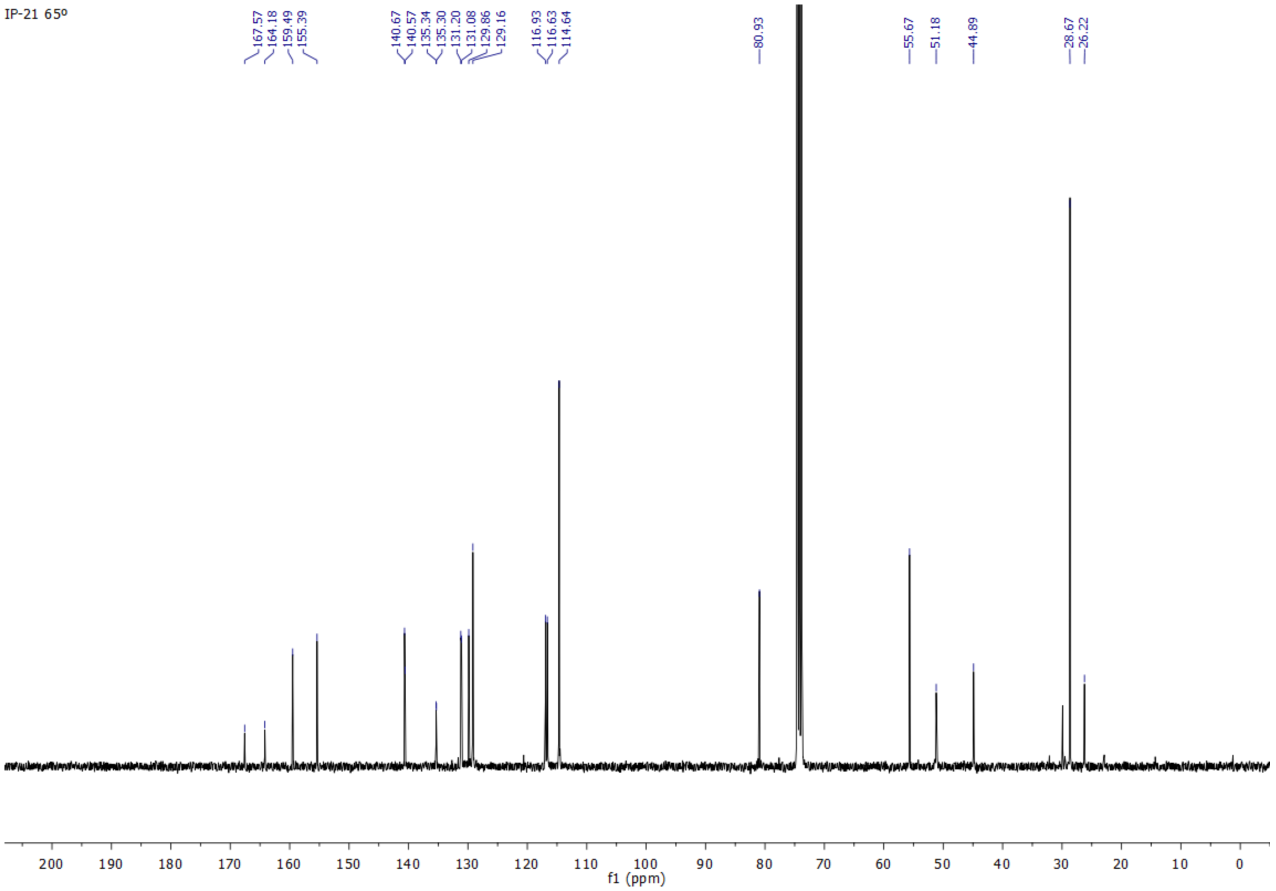

^{19}F NMR spectrum of **6bb** in $C_2D_2Cl_4$ at 65 ºC

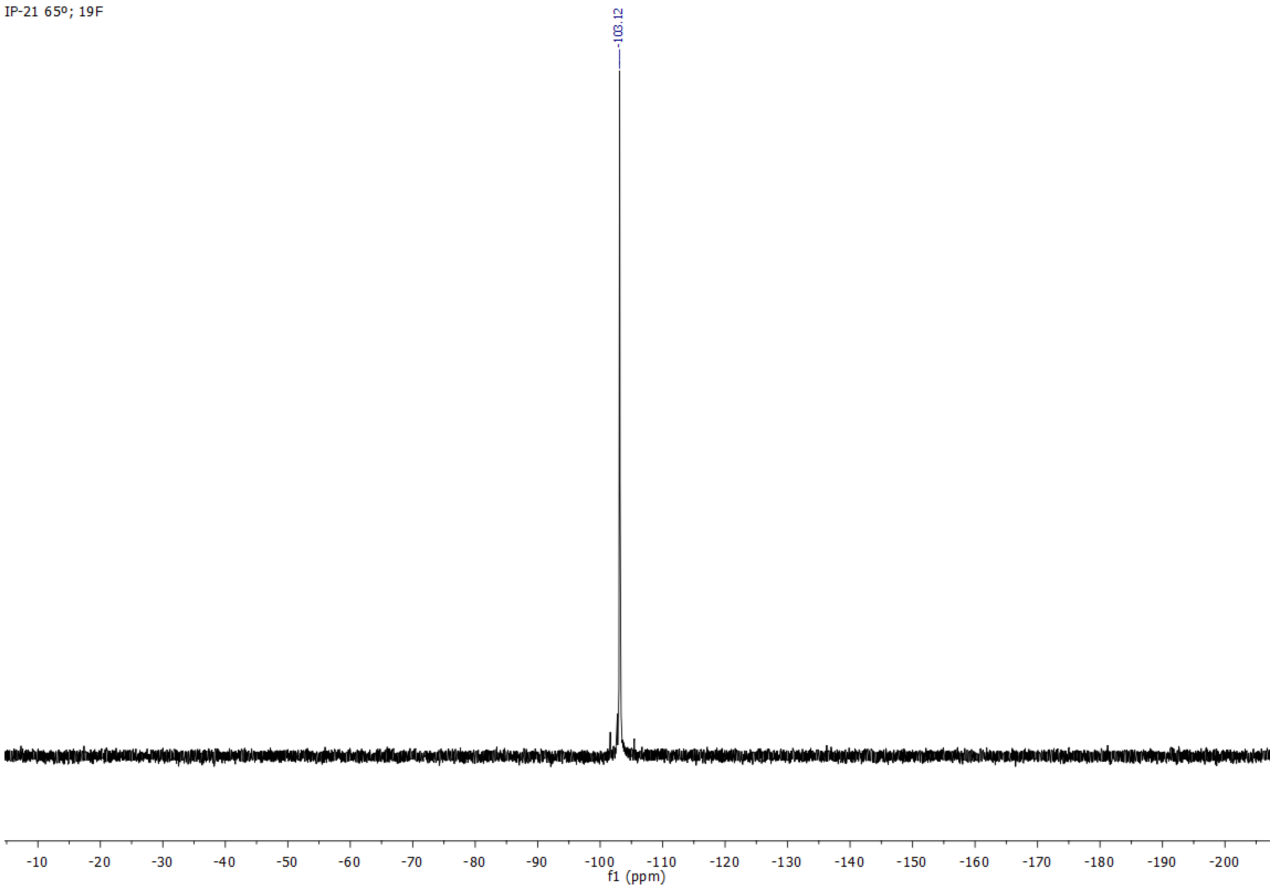

^{1}H NMR (300 MHz) spectrum of **6bc** in $C_2D_2Cl_4$ at 65 ºC

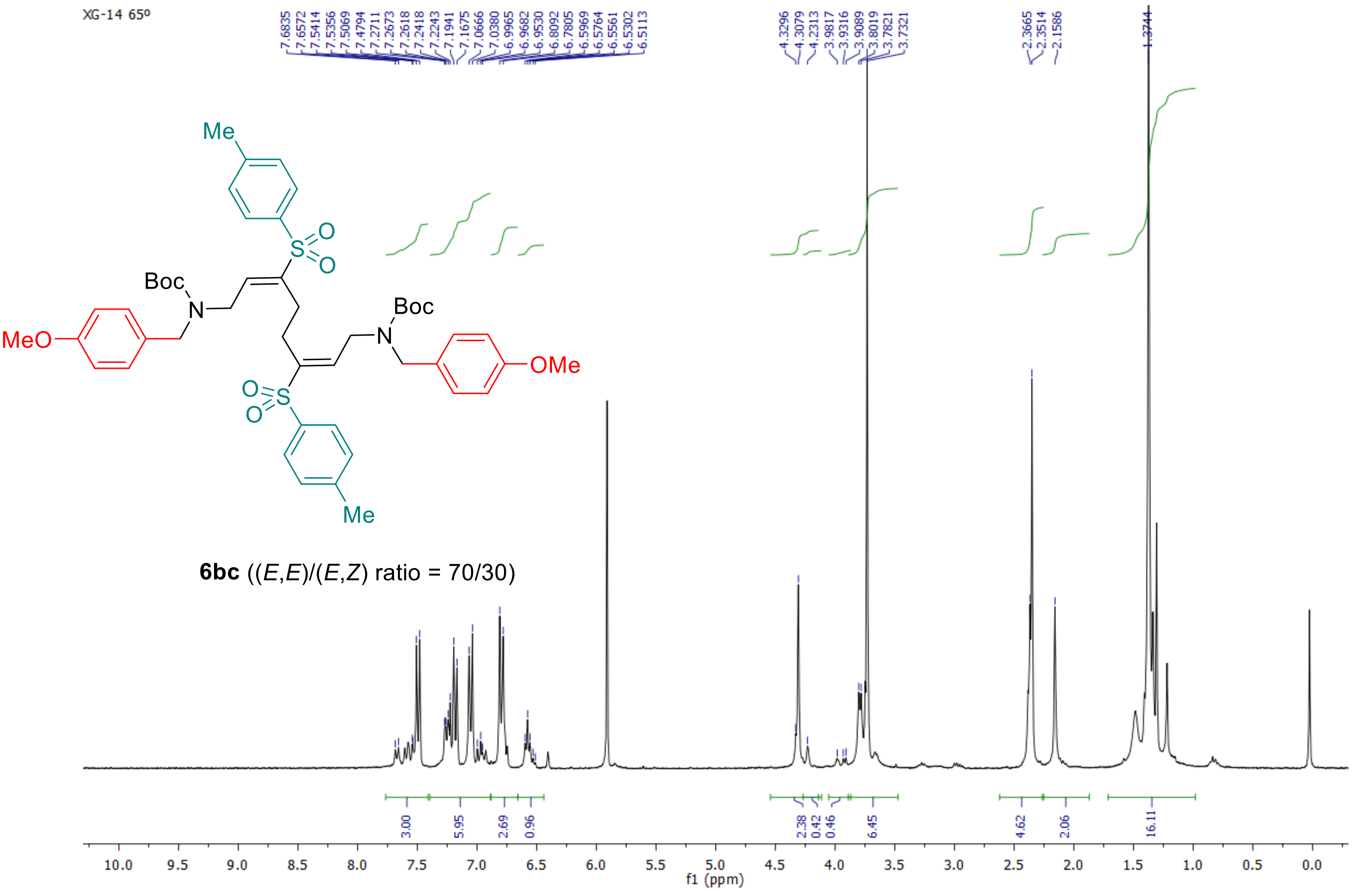

^{13}C NMR (75 MHz) spectrum of **6bc** in $C_2D_2Cl_4$ at 65 ºC

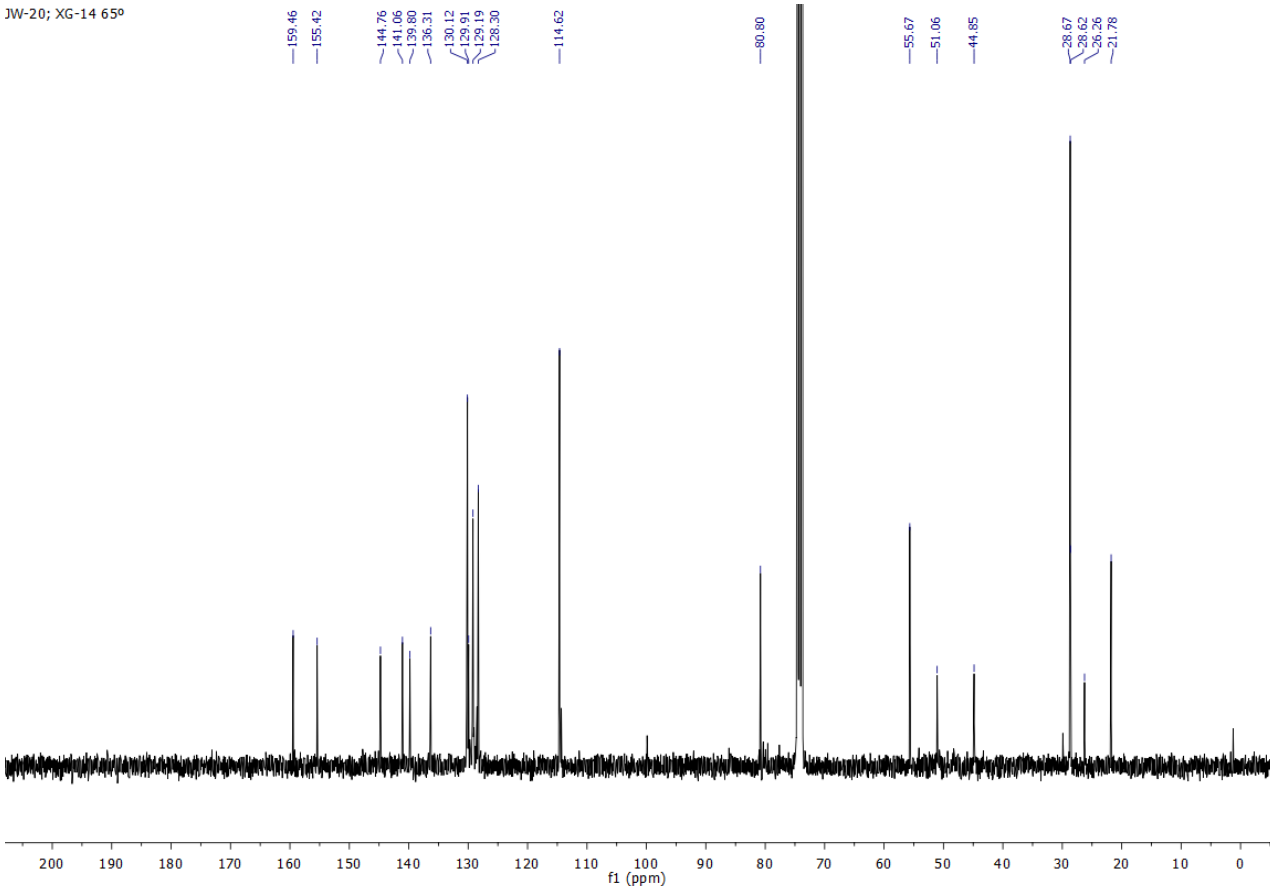

^{1}H NMR (300 MHz) spectrum of **6bd** in $C_2D_2Cl_4$ at 65 ºC

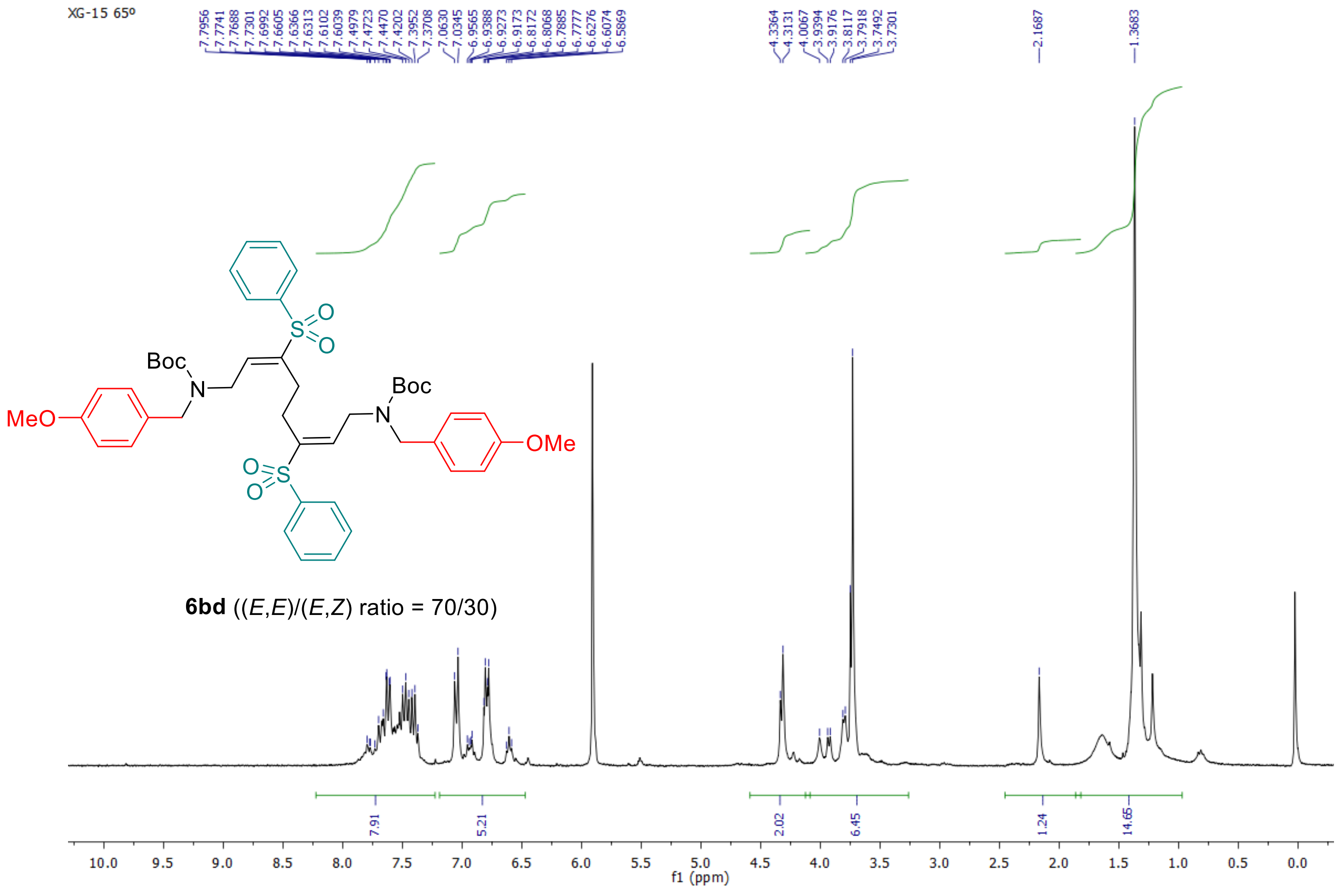

^{13}C NMR (75 MHz) spectrum of **6bd** in $C_2D_2Cl_4$ at 65 ºC

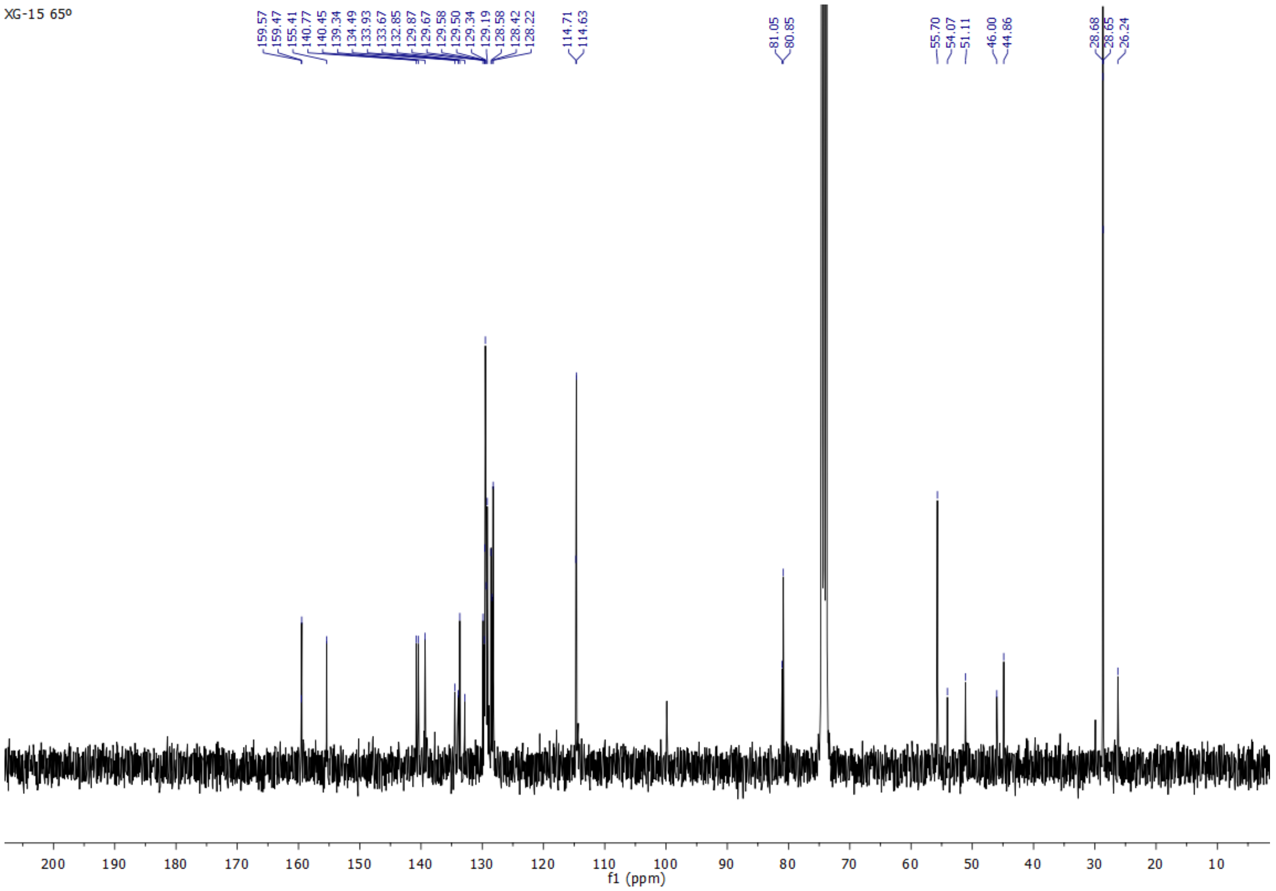

^{1}H NMR (300 MHz) spectrum of **6ca** in $C_2D_2Cl_4$ at 65 ºC

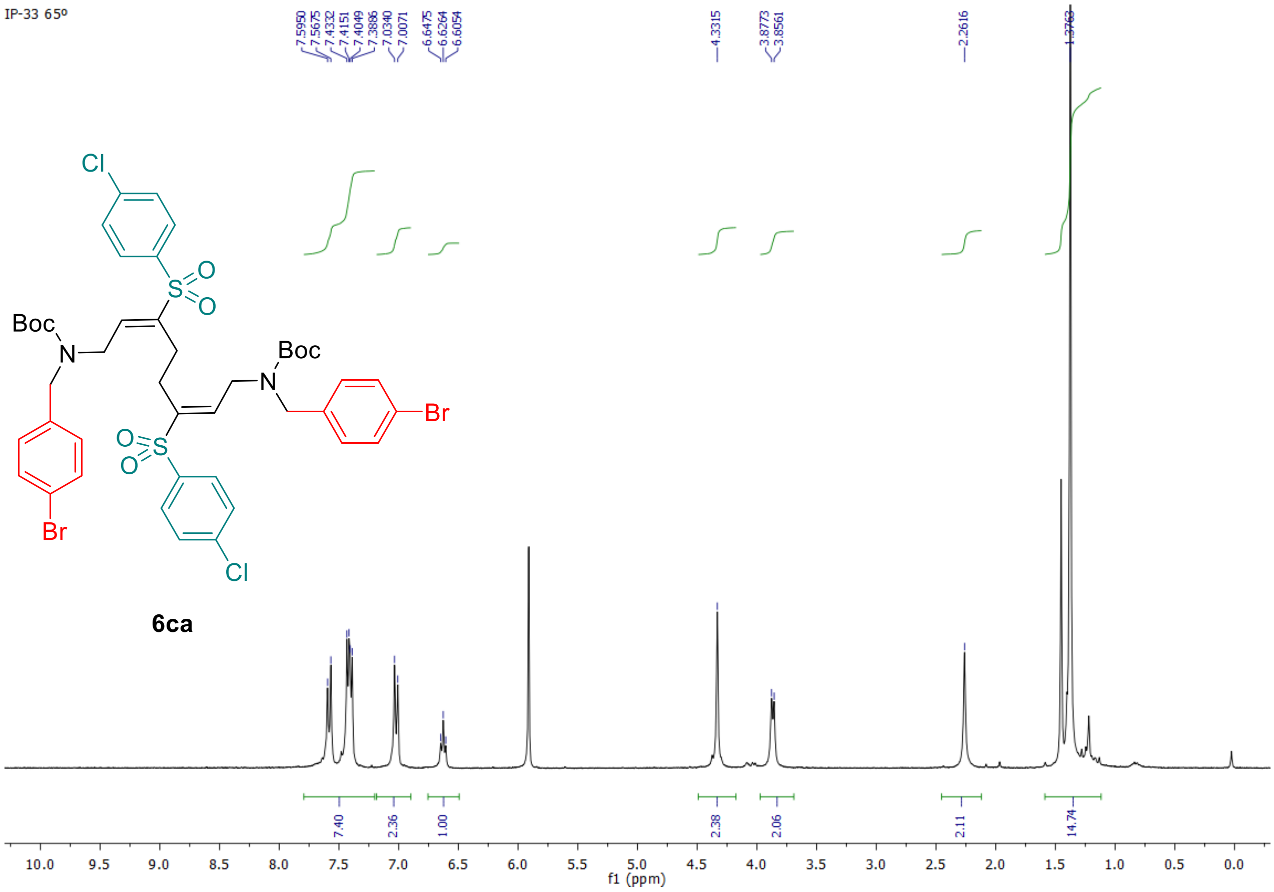

^{13}C NMR (75 MHz) spectrum of **6ca** in $C_2D_2Cl_4$ at 65 ºC

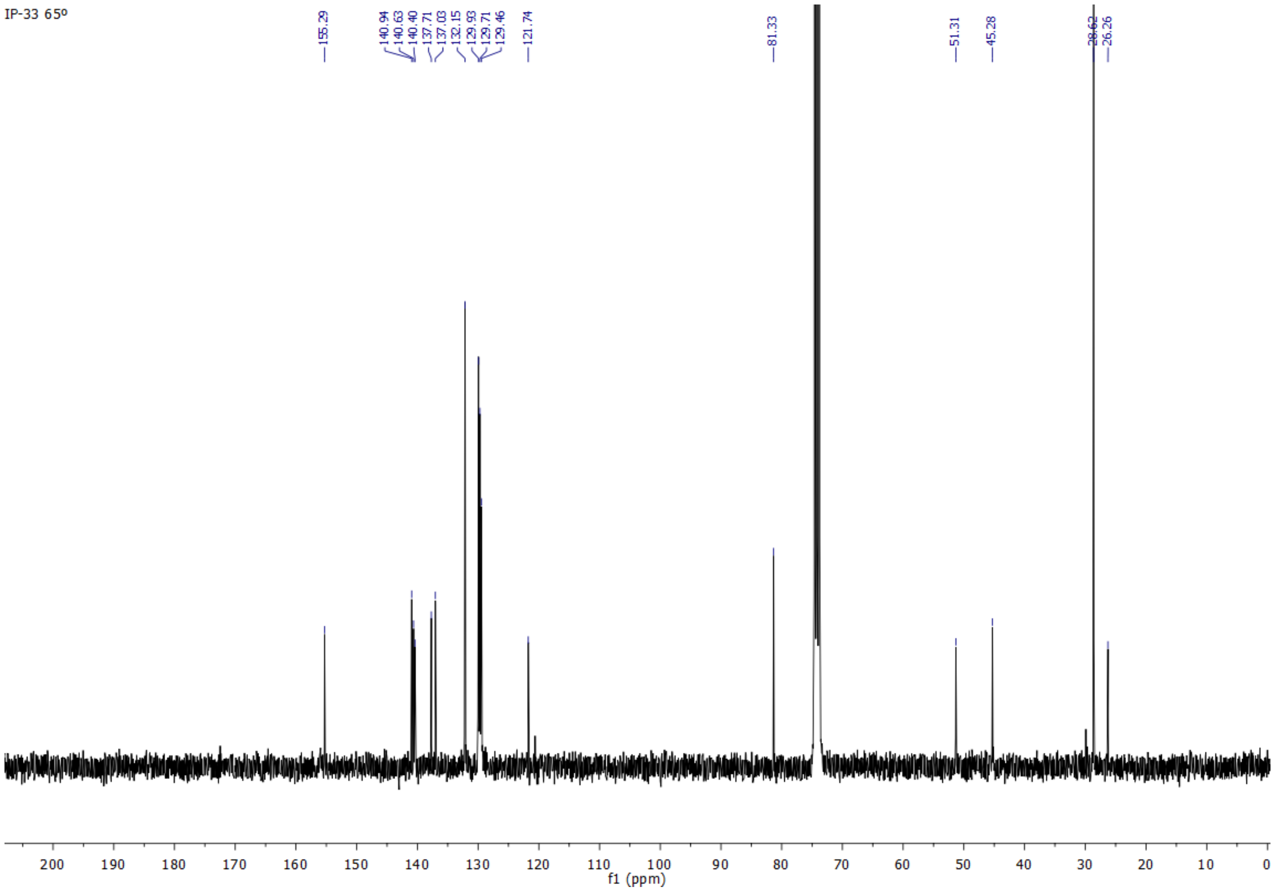

^{1}H NMR (300 MHz) spectrum of **6cb** in $C_2D_2Cl_4$ at 65 ºC

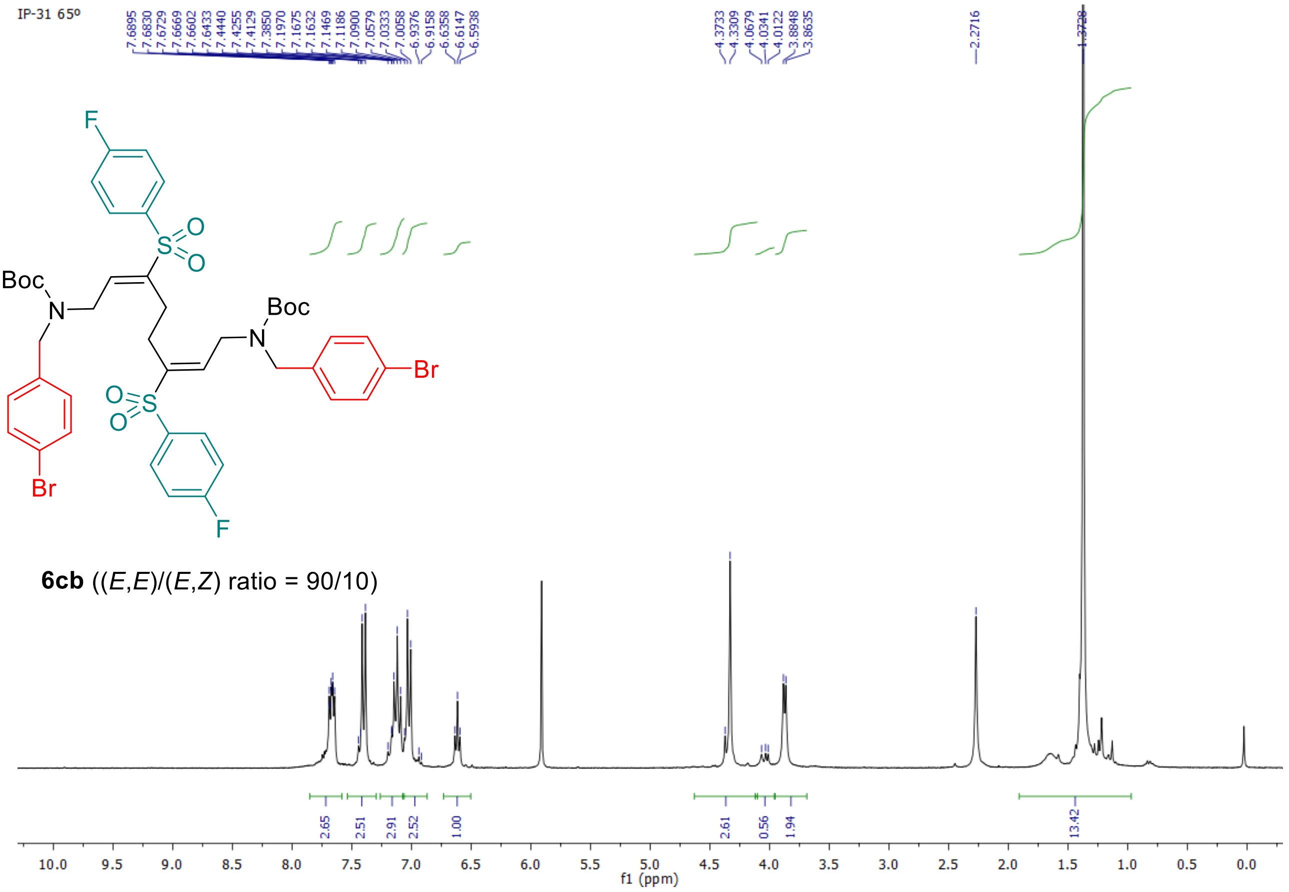

^{13}C NMR (75 MHz) spectrum of **6cb** in $C_2D_2Cl_4$ at 65 ºC

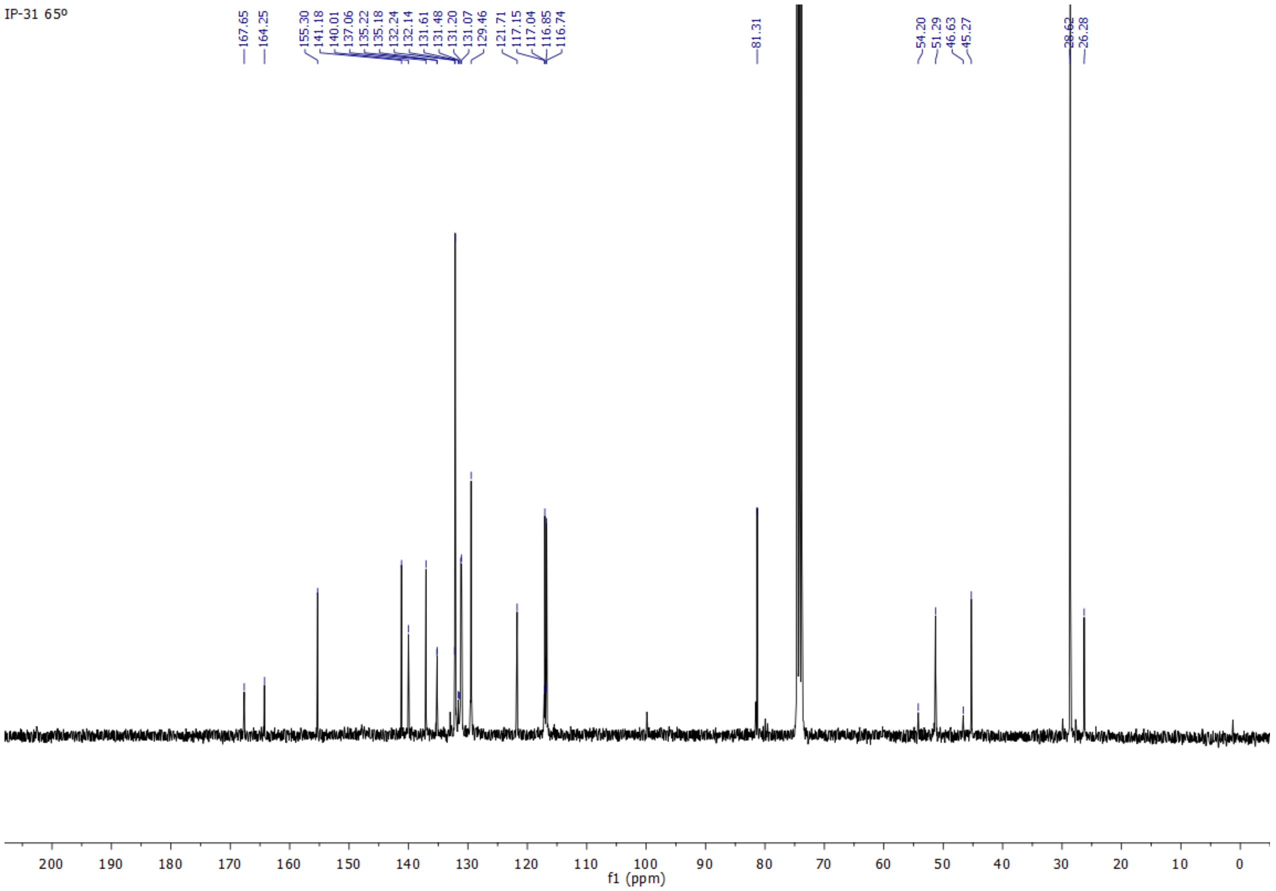

^{19}F NMR spectrum of **6cb** in $C_2D_2Cl_4$ at 65 ºC

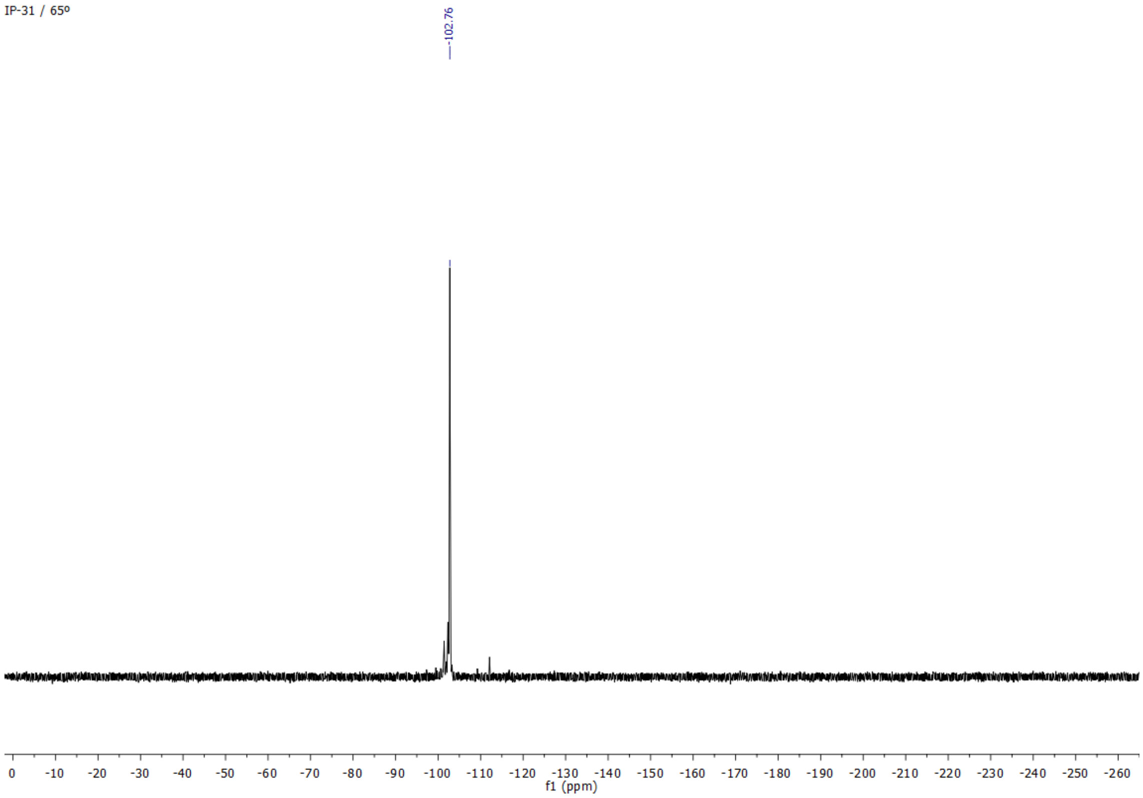

^{1}H NMR (300 MHz) spectrum of **6ce** in $C_2D_2Cl_4$ at 65 ºC

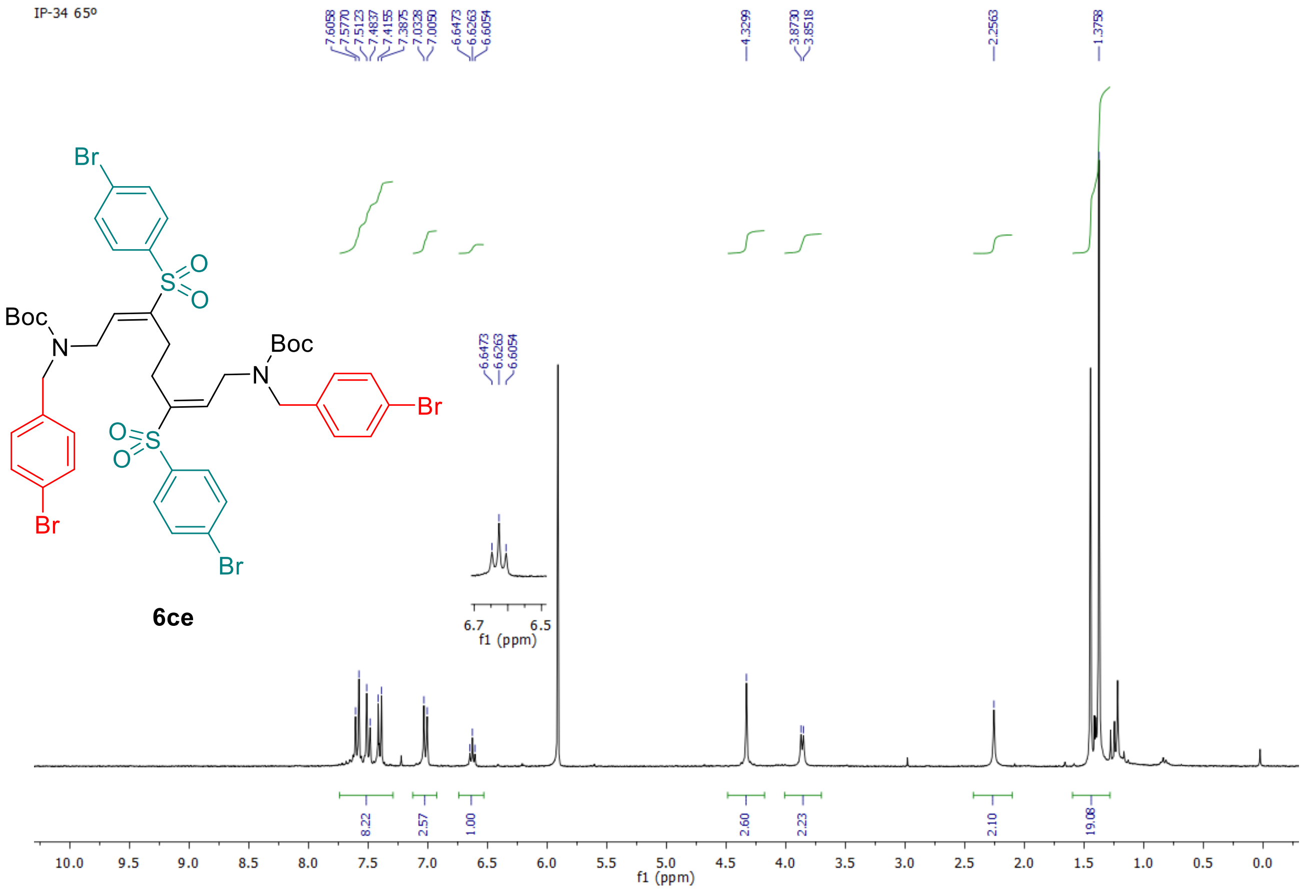

6ce

^{13}C NMR (75 MHz) spectrum of **6ce** in $C_2D_2Cl_4$ at 65 ºC

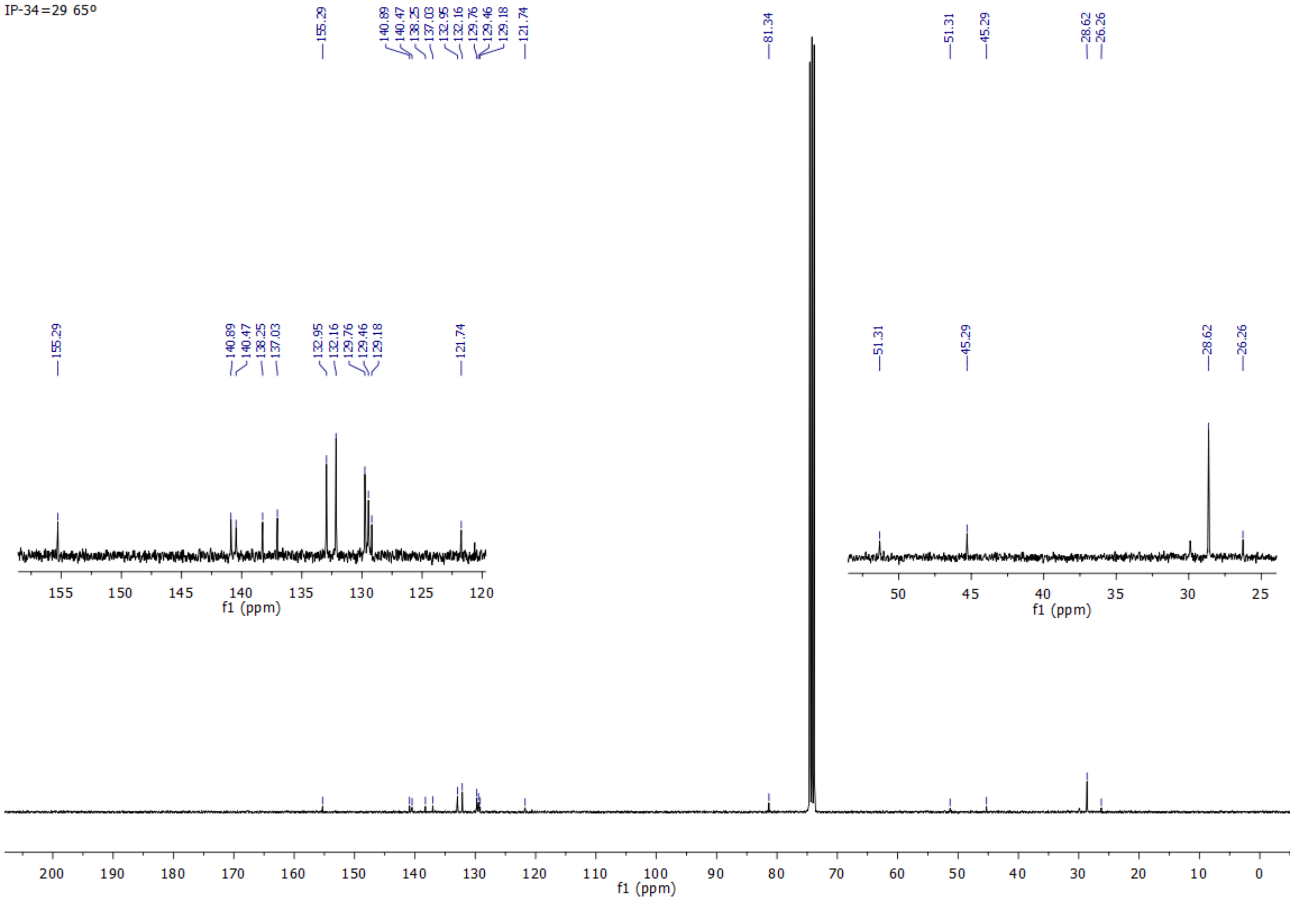

^{1}H NMR (300 MHz) spectrum of **6db** in $C_2D_2Cl_4$ at 65 ºC

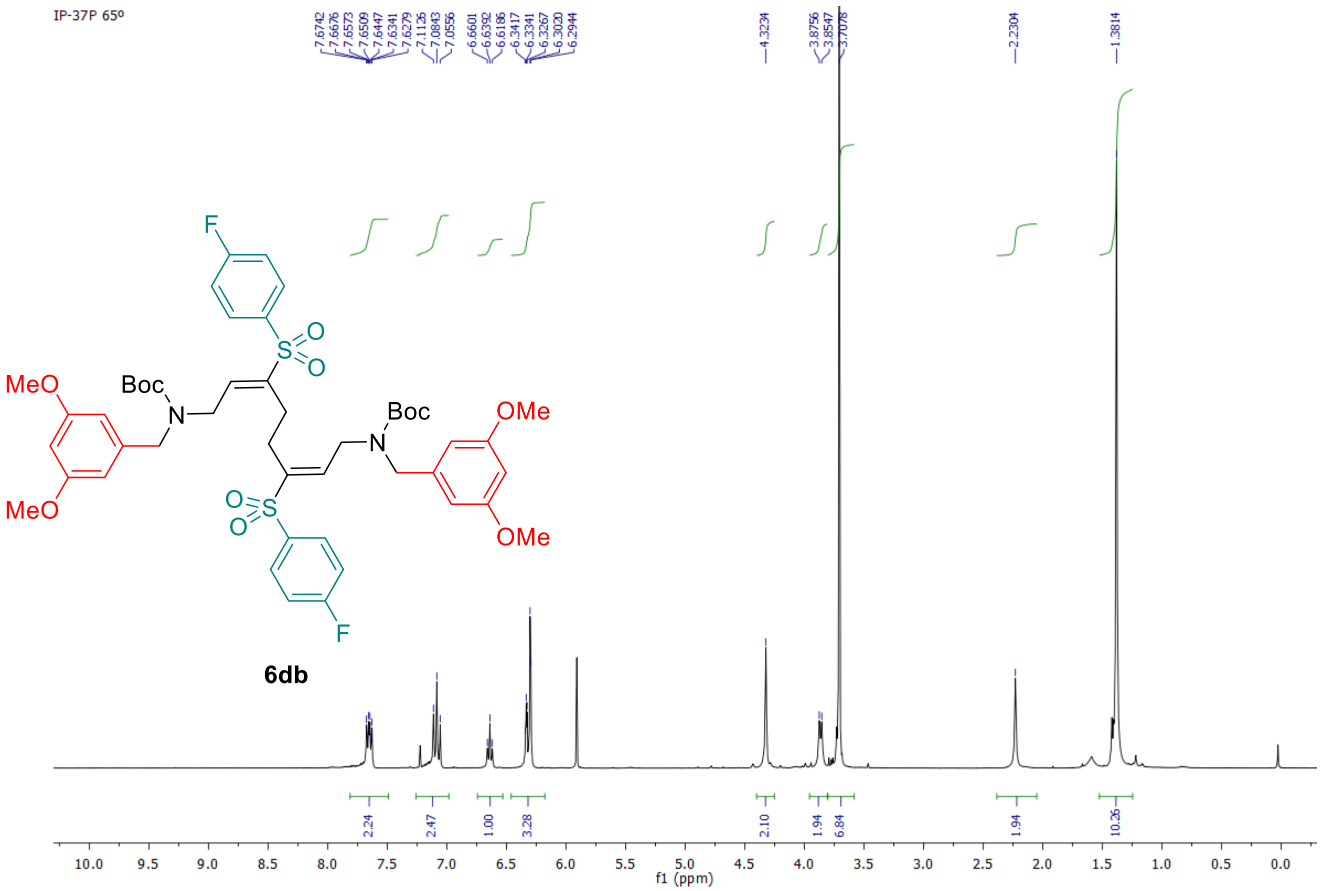

^{13}C NMR (75 MHz) spectrum of **6db** in $C_2D_2Cl_4$ at 65 ºC

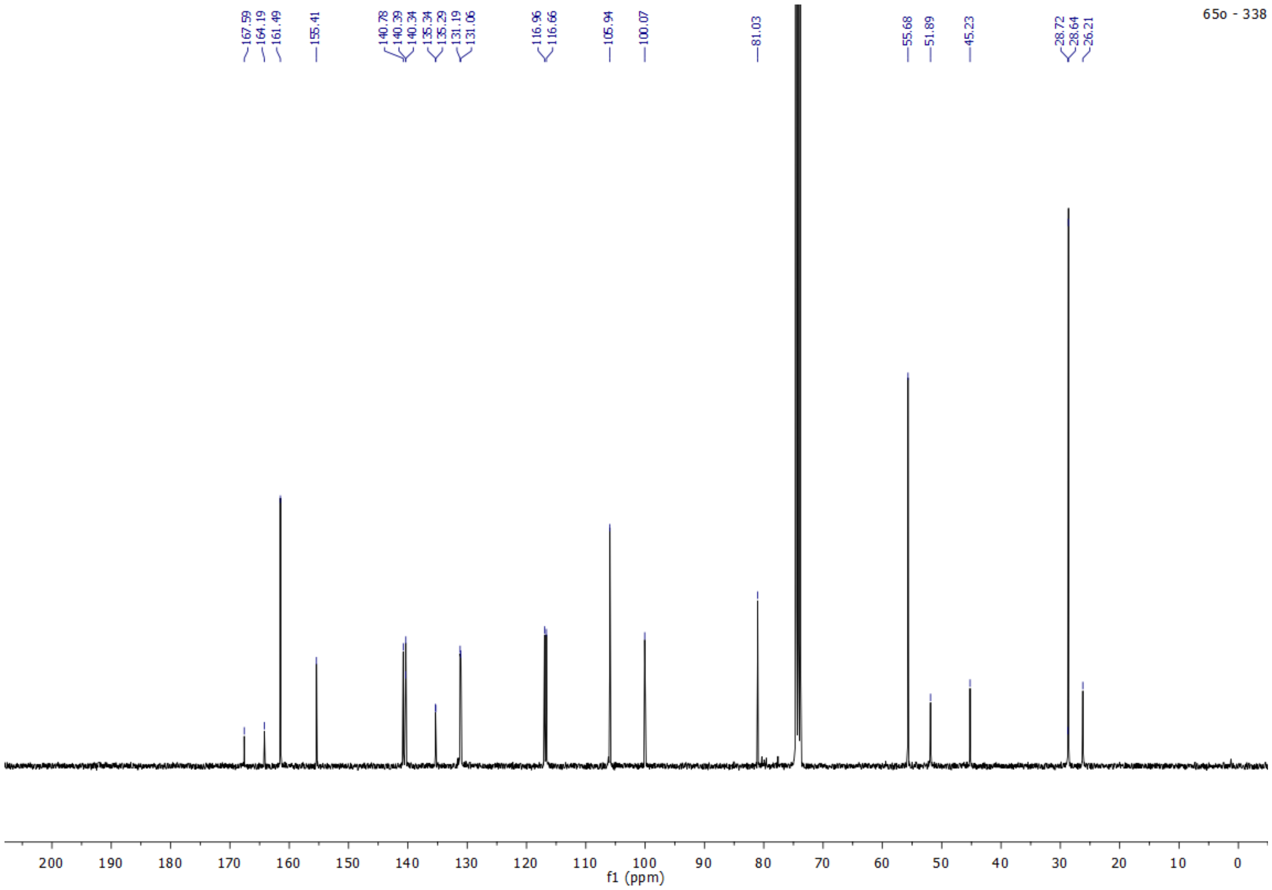

^{19}F NMR spectrum of **6db** in $C_2D_2Cl_4$ at 65 ºC

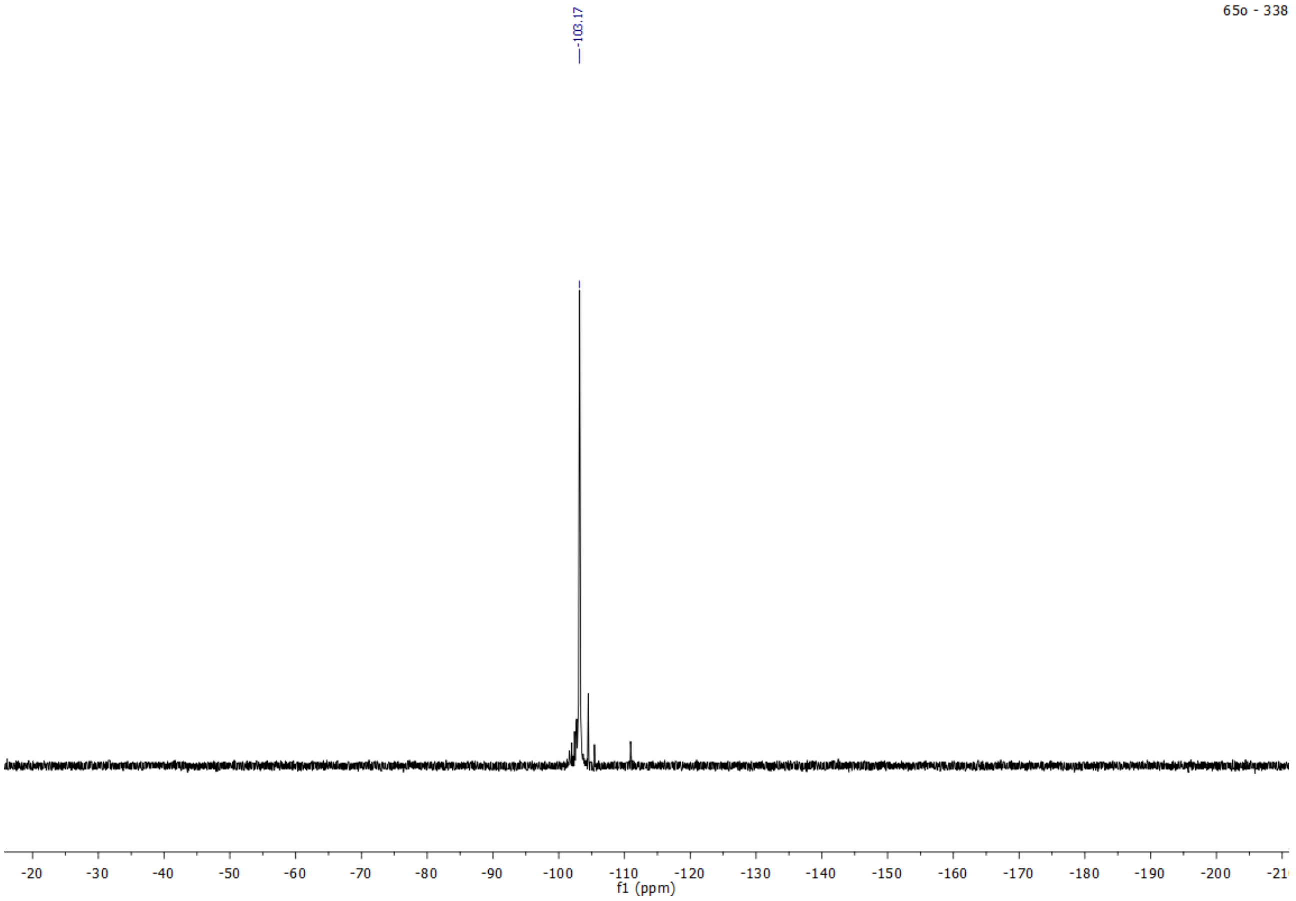

^{1}H NMR (300 MHz) spectrum of **6de** in $C_2D_2Cl_4$ at 65 ºC

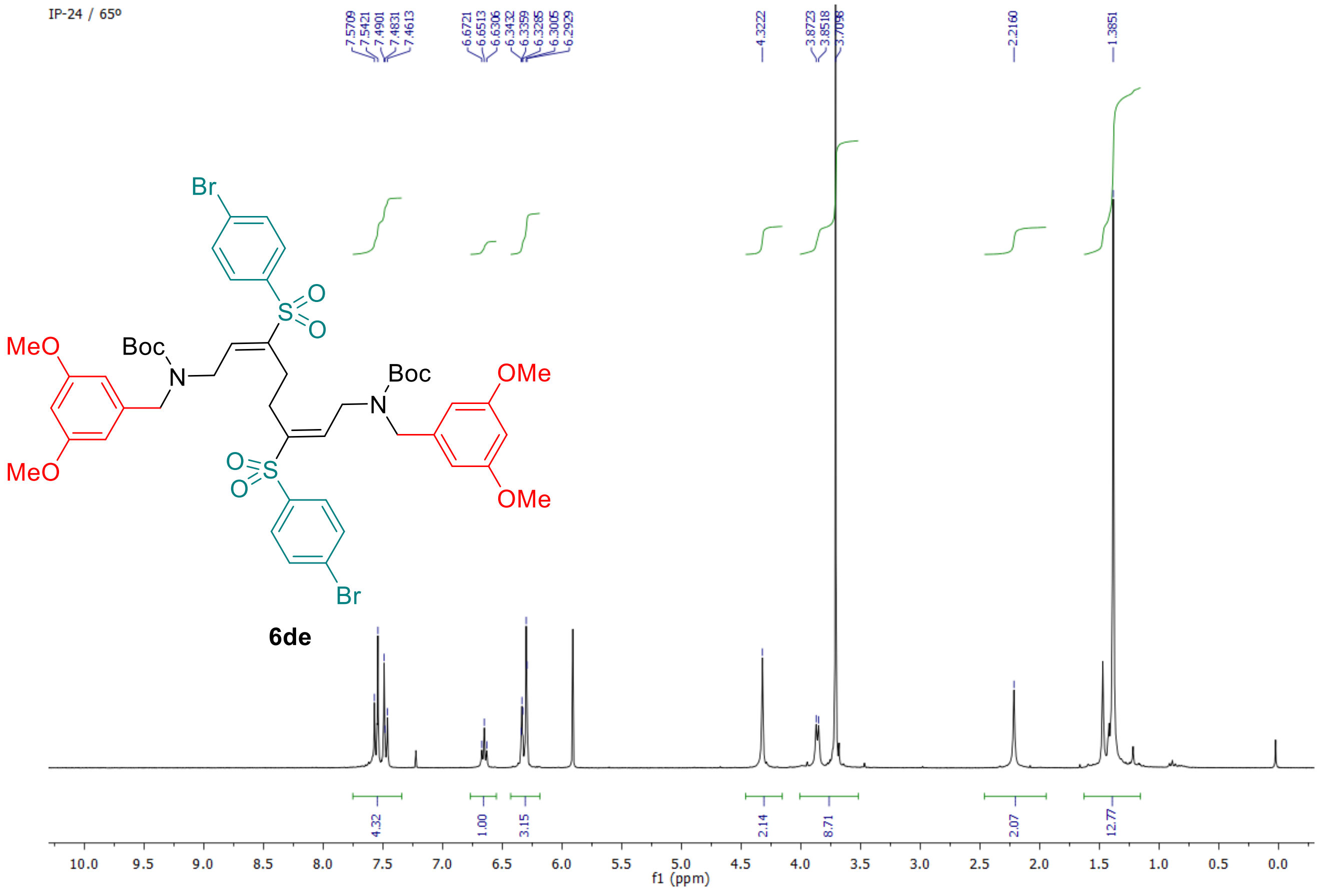

^{13}C NMR (75 MHz) spectrum of **6de** in $C_2D_2Cl_4$ at 65 ºC

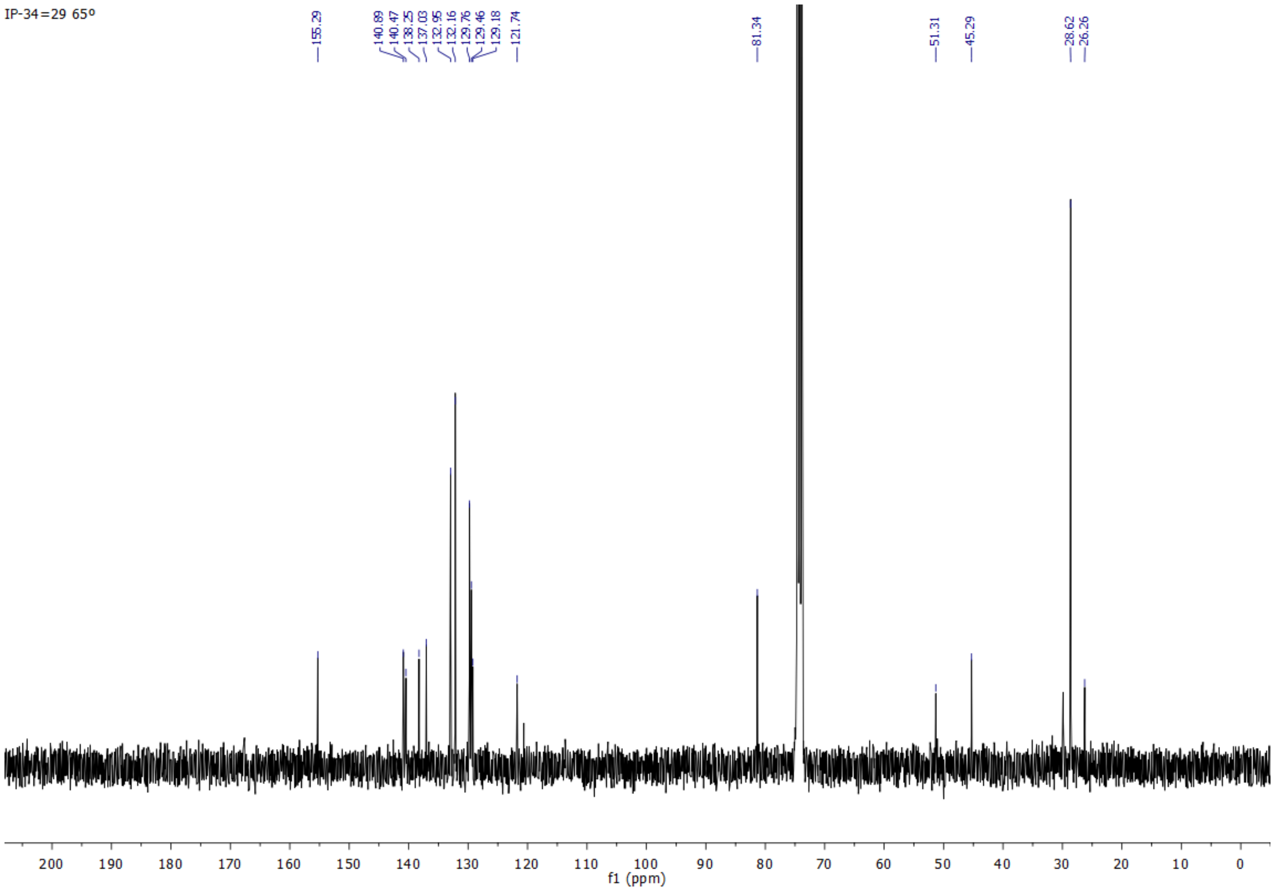

^{1}H NMR (300 MHz) spectrum of **6ea** in $C_2D_2Cl_4$ at 55 ºC

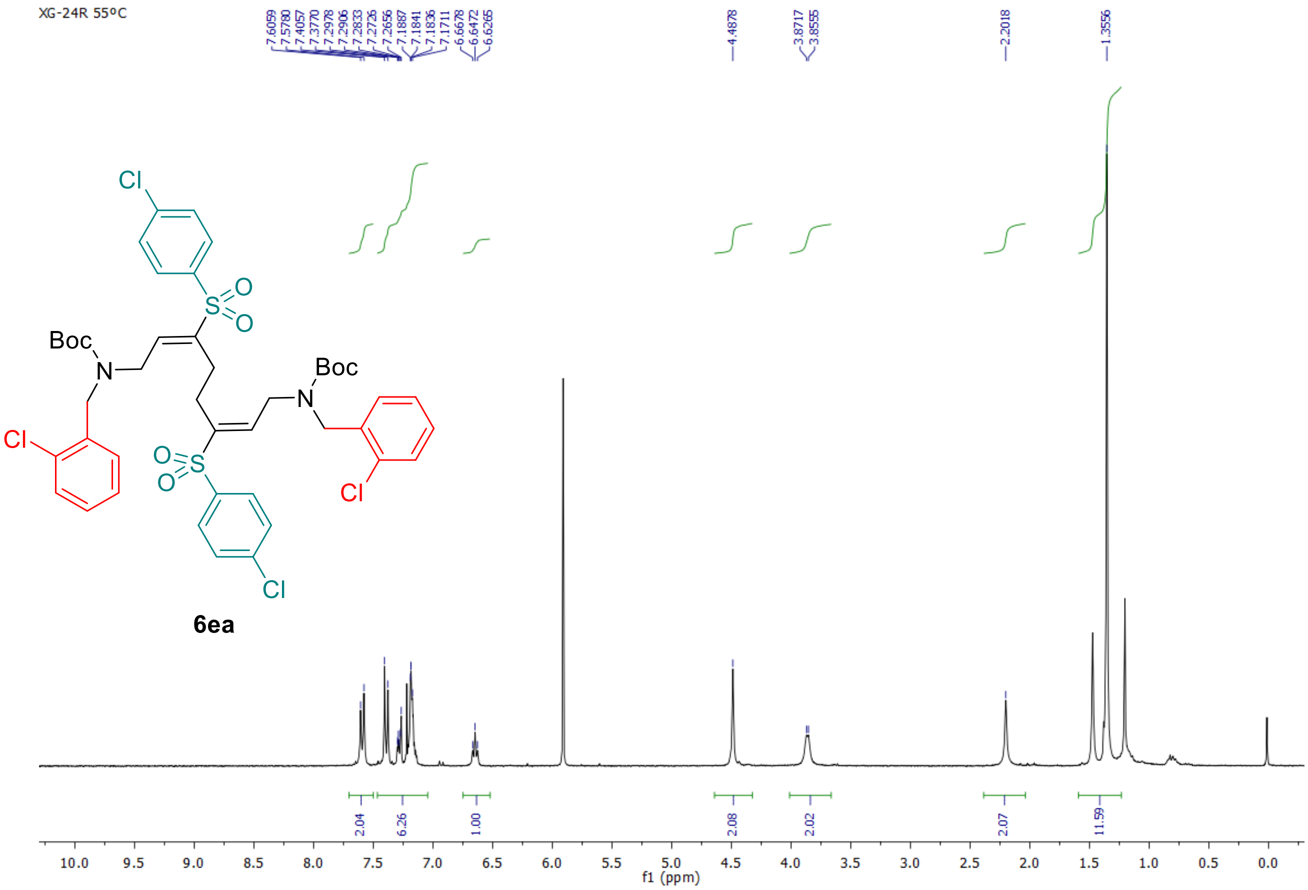

^{13}C NMR (75 MHz) spectrum of **6ea** in $C_2D_2Cl_4$ at 55 ºC

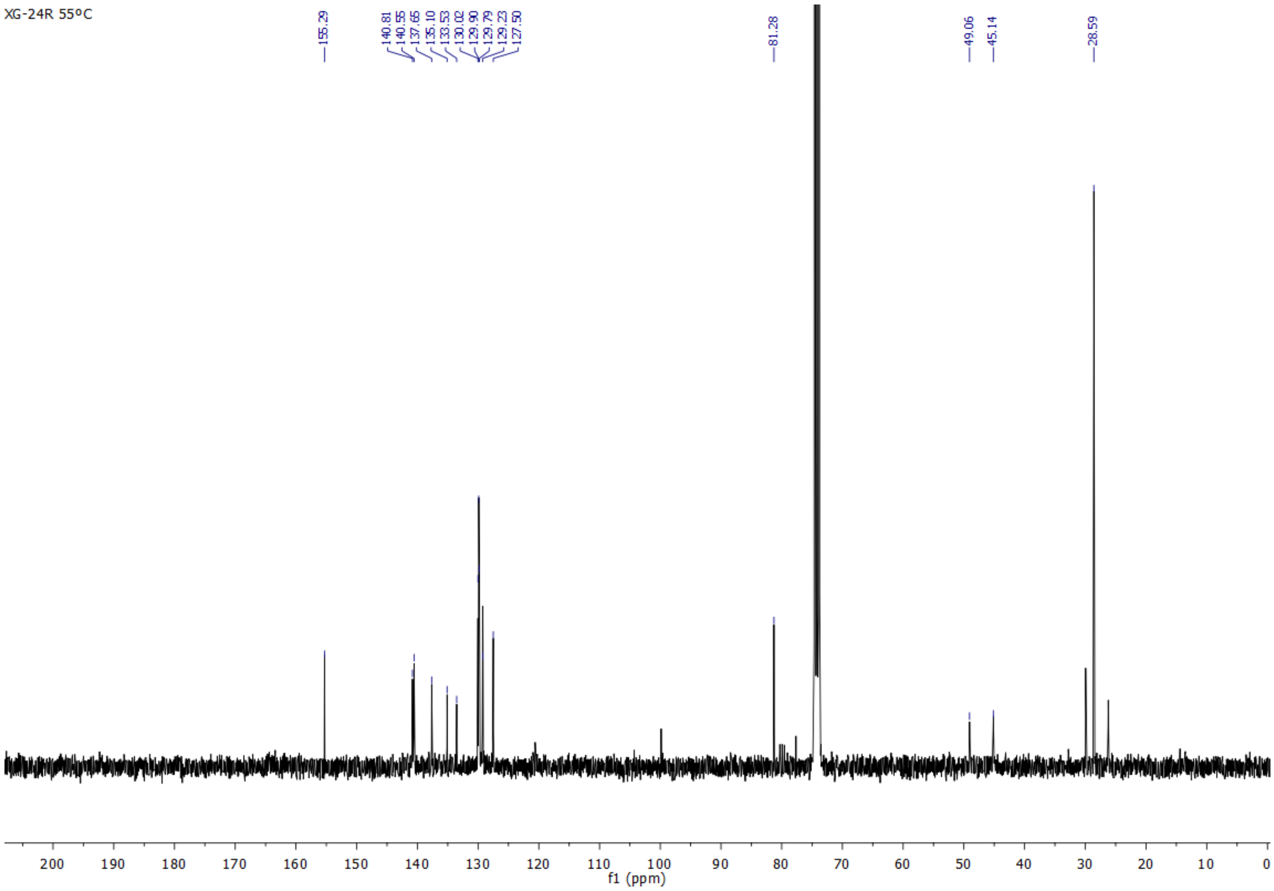

^{1}H NMR (300 MHz) spectrum of **6ec** in $C_2D_2Cl_4$ at 55 ºC

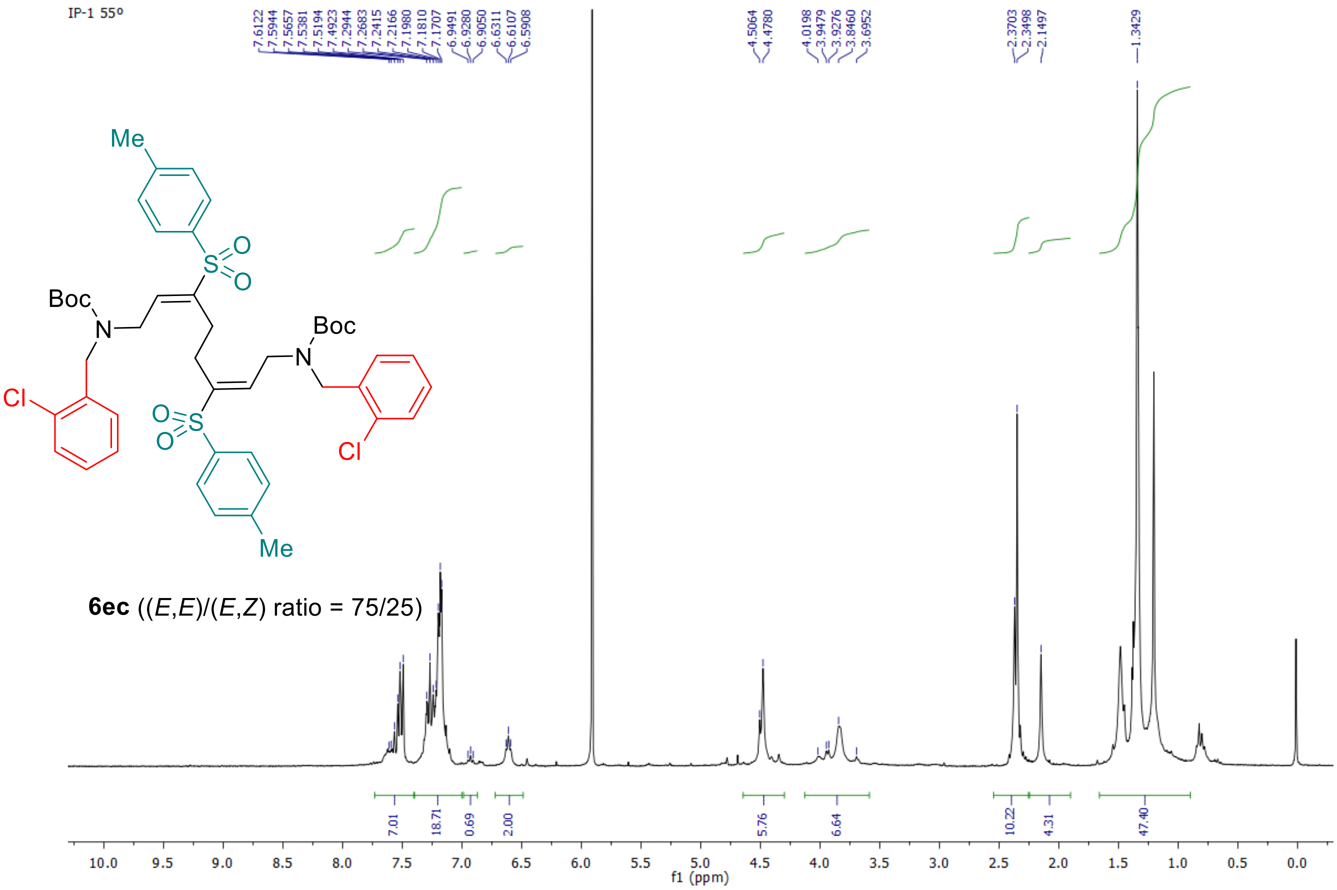

^{13}C NMR (75 MHz) spectrum of **6ec** in $C_2D_2Cl_4$ at 55 ºC

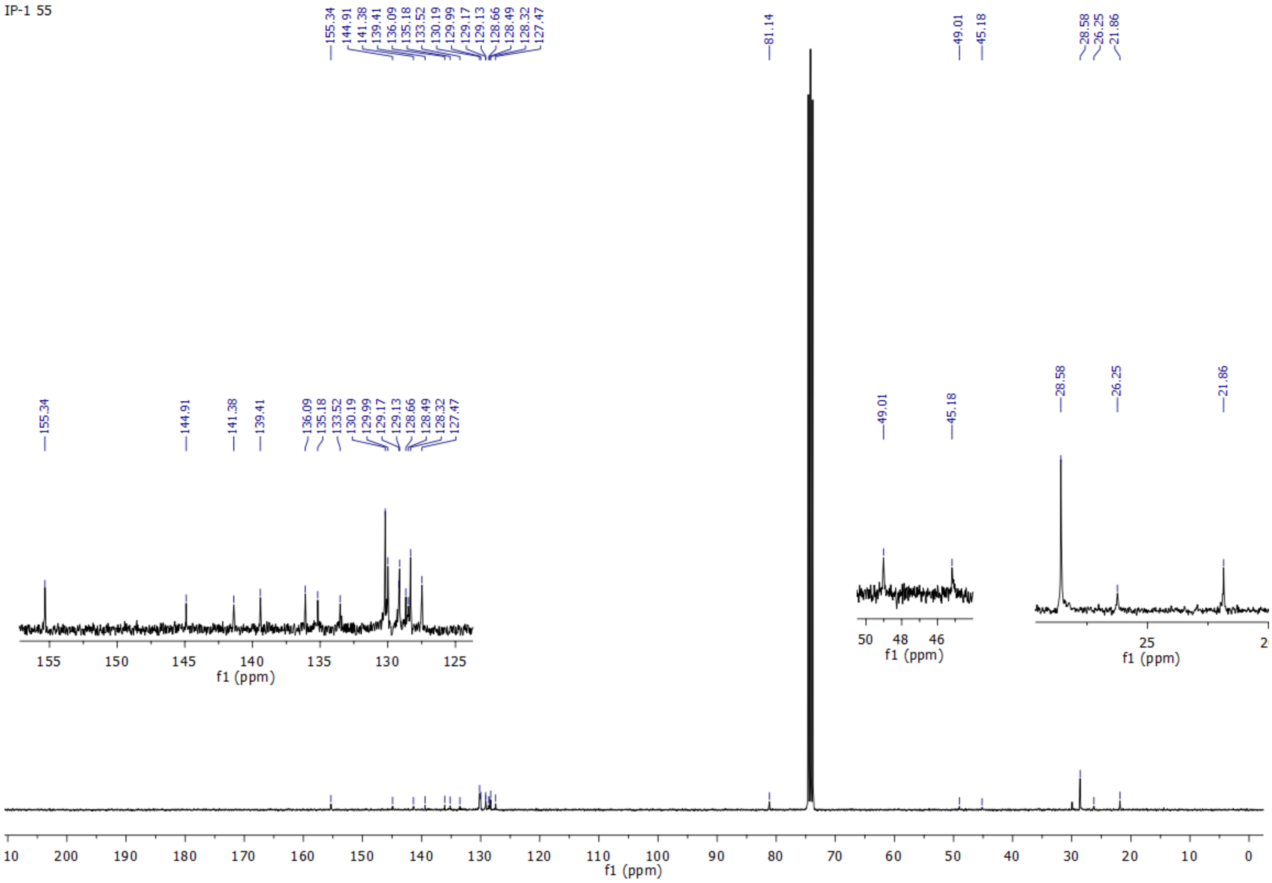

^{1}H NMR (300 MHz) spectrum of **6fa** in $C_2D_2Cl_4$ at 55 ºC

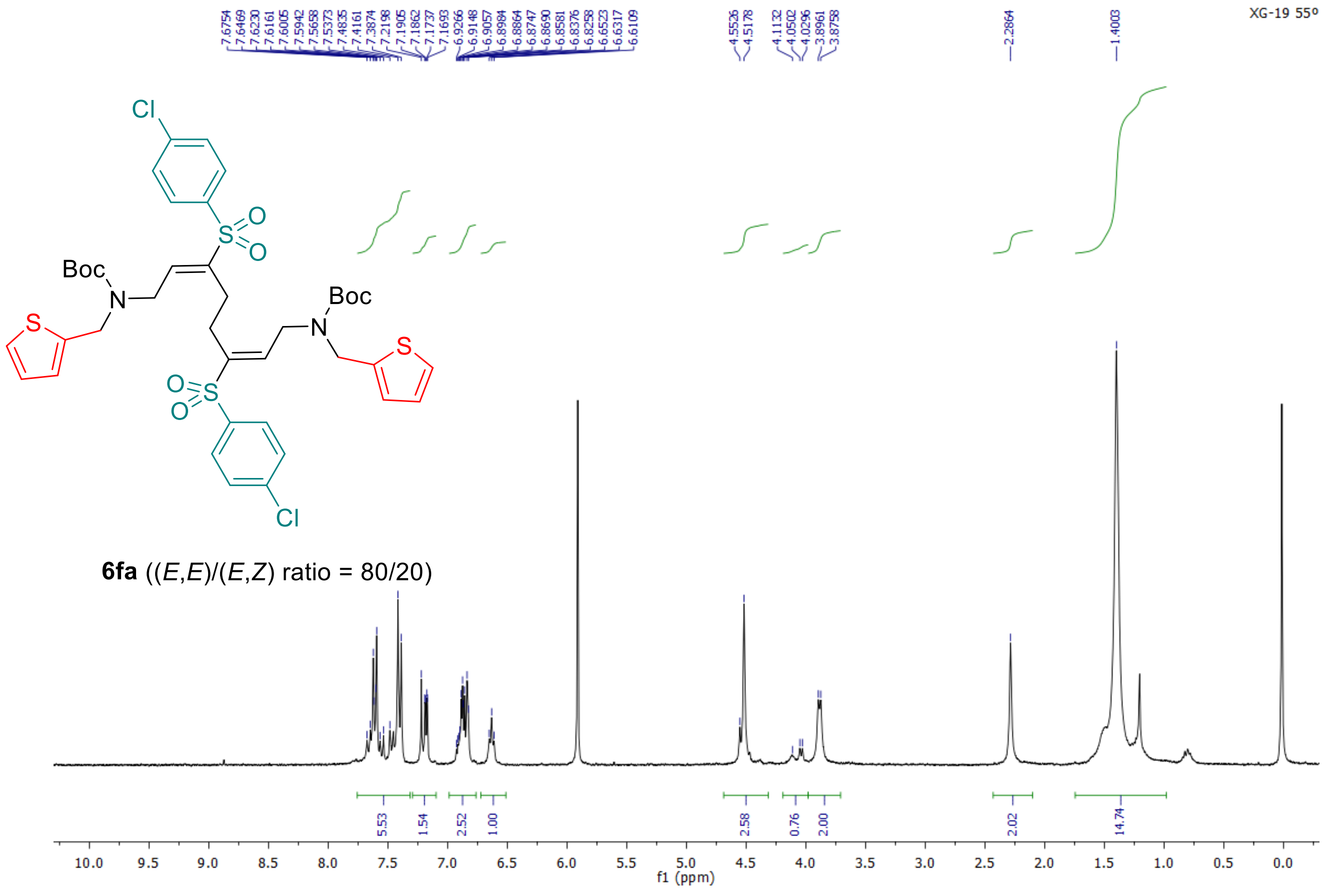

6fa ((*E*,*E*)/(*E*,*Z*) ratio = 80/20)

^{13}C NMR (75 MHz) spectrum of **6fa** in $C_2D_2Cl_4$ at 55 ºC

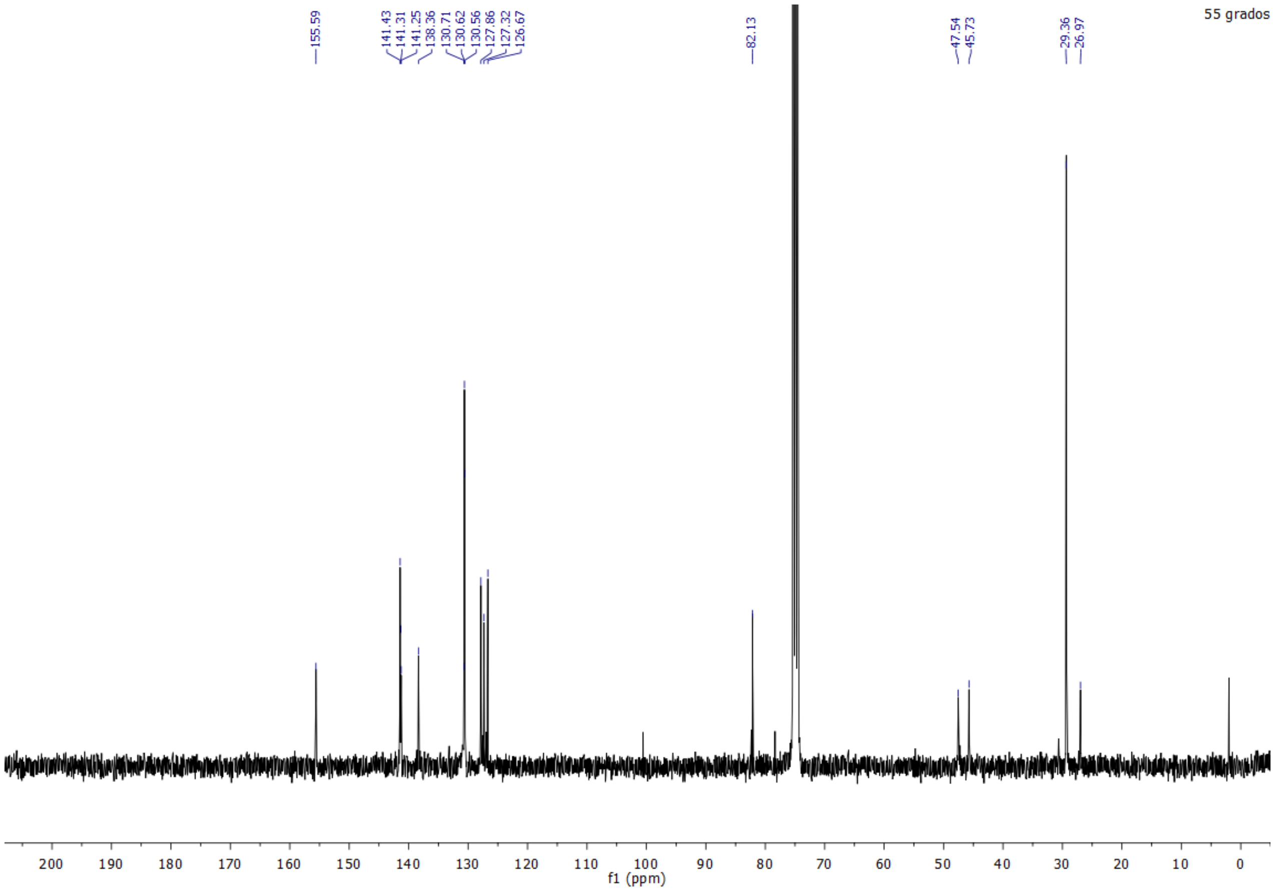

^{1}H NMR (300 MHz) spectrum of **7bae** in $C_2D_2Cl_4$ at 65 ºC

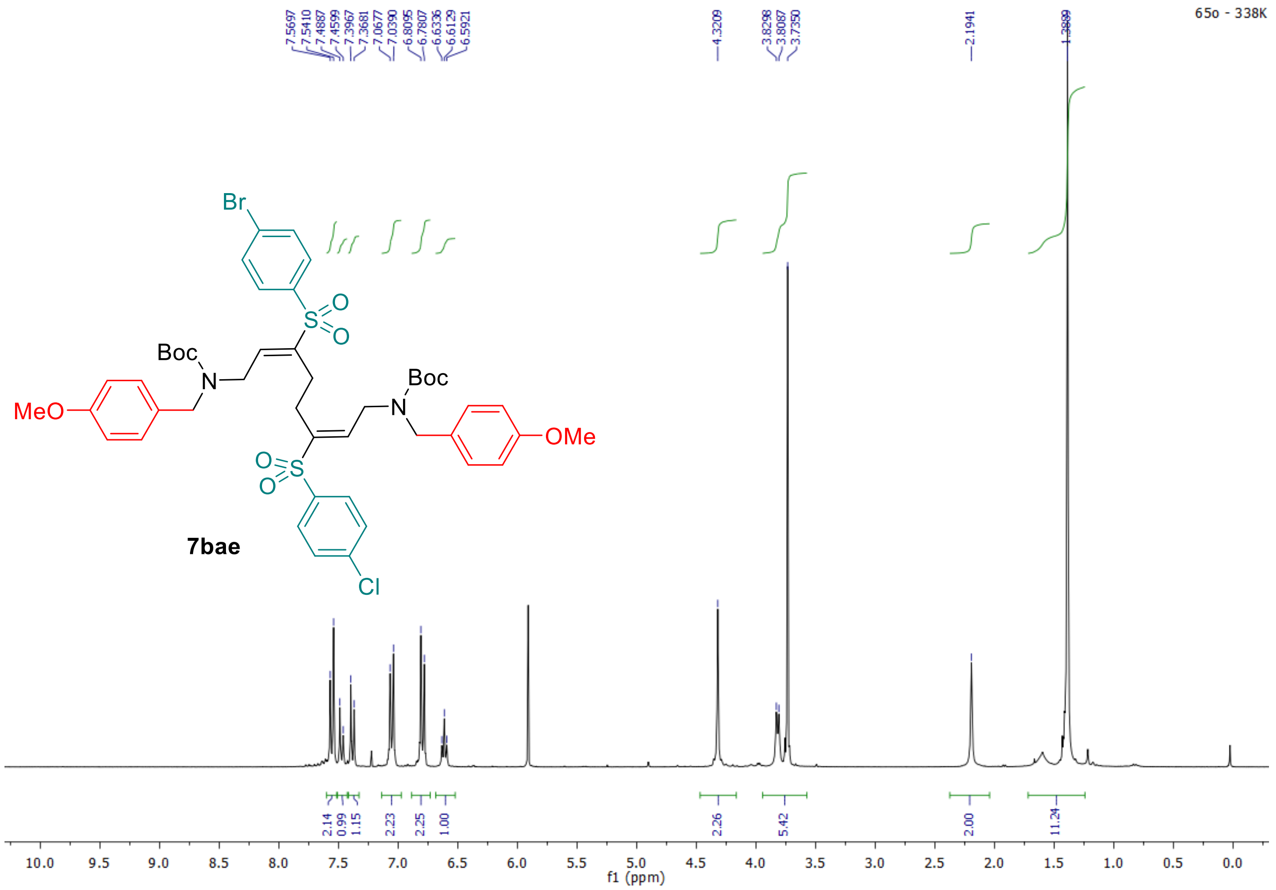

^{13}C NMR (75 MHz) spectrum of **7bae** in $C_2D_2Cl_4$ at 55 ºC

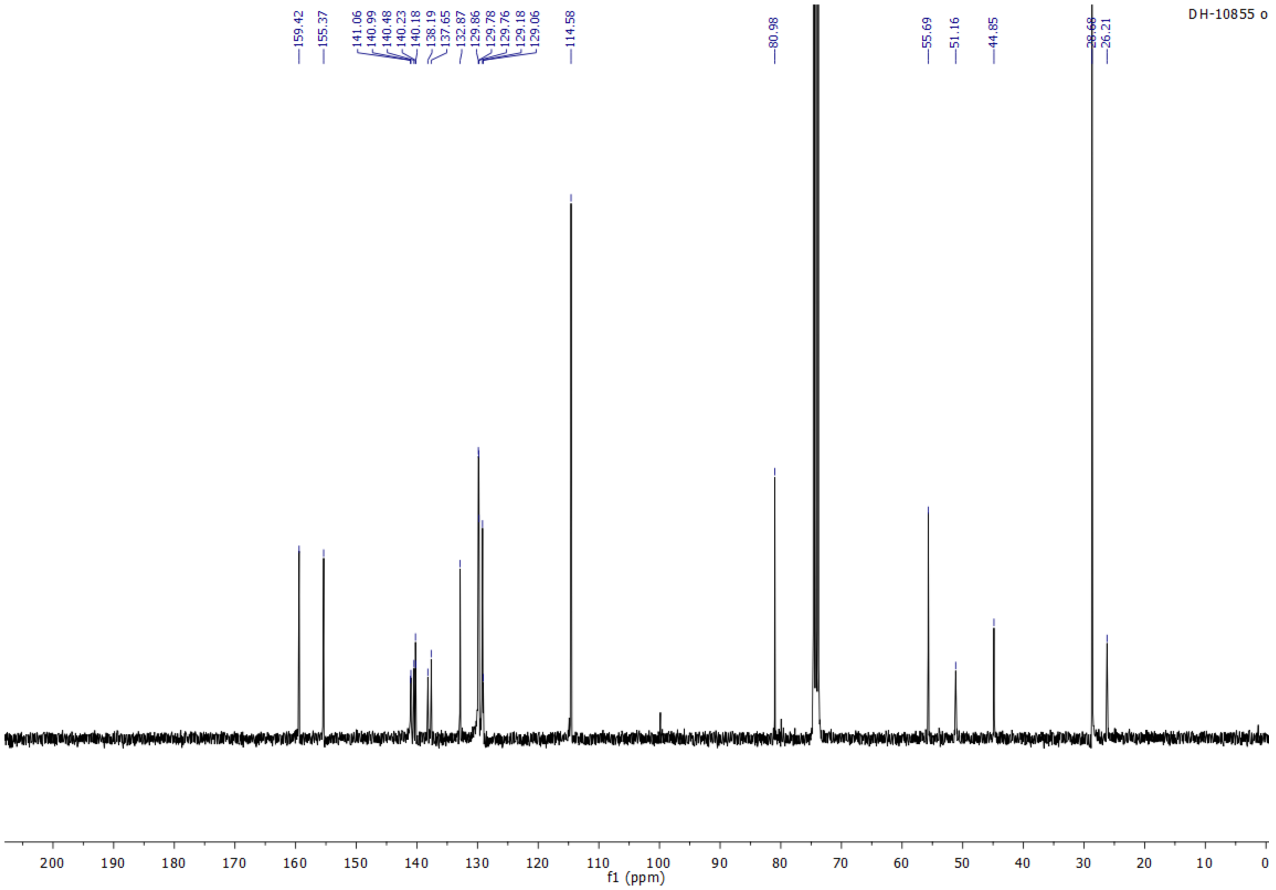

DEPT NMR (75 MHz) spectrum of **7bae** in $C_2D_2Cl_4$ at 55 ºC

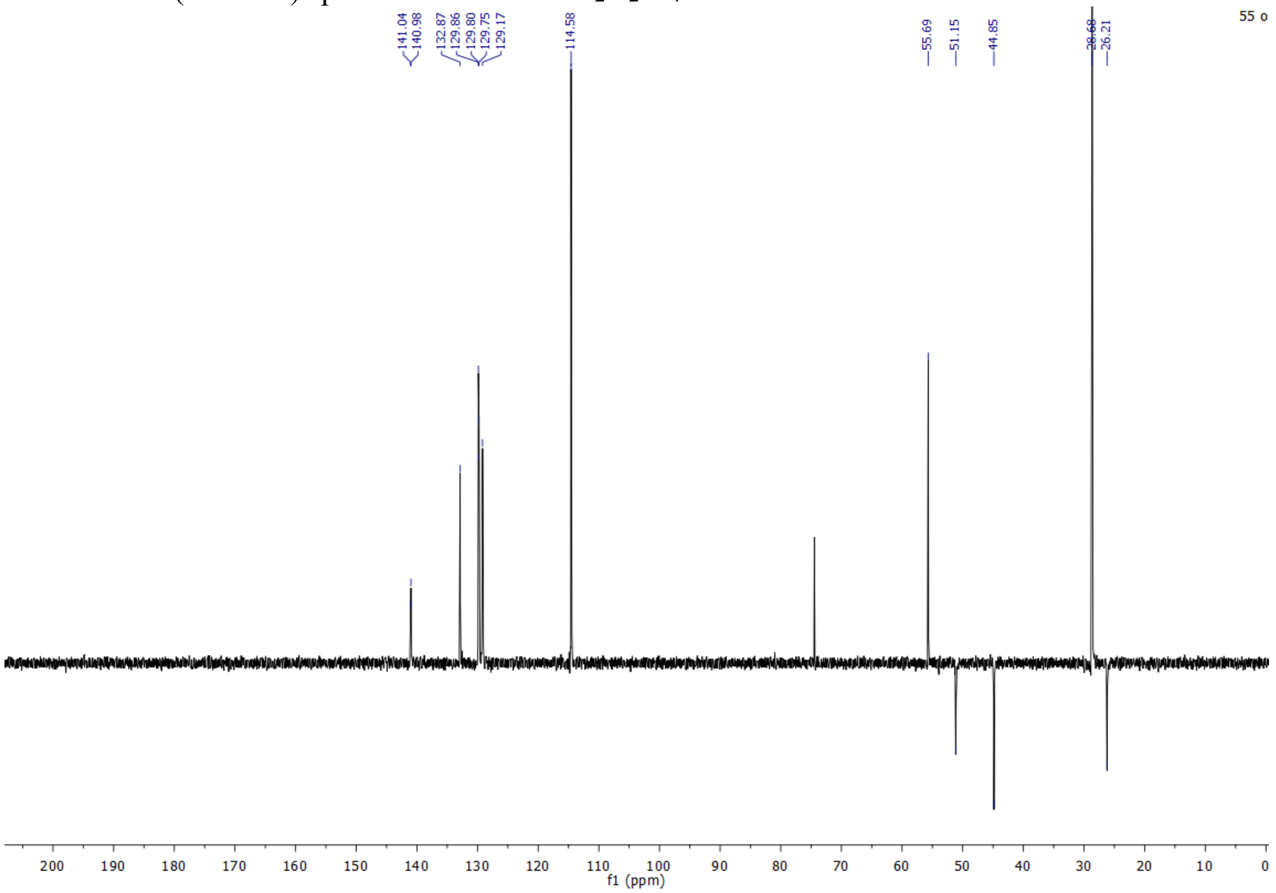

Two-dimensional HMQC NMR spectrum ^{1}H-^{13}C of **7bae** in $C_2D_2Cl_4$ at 55 ºC

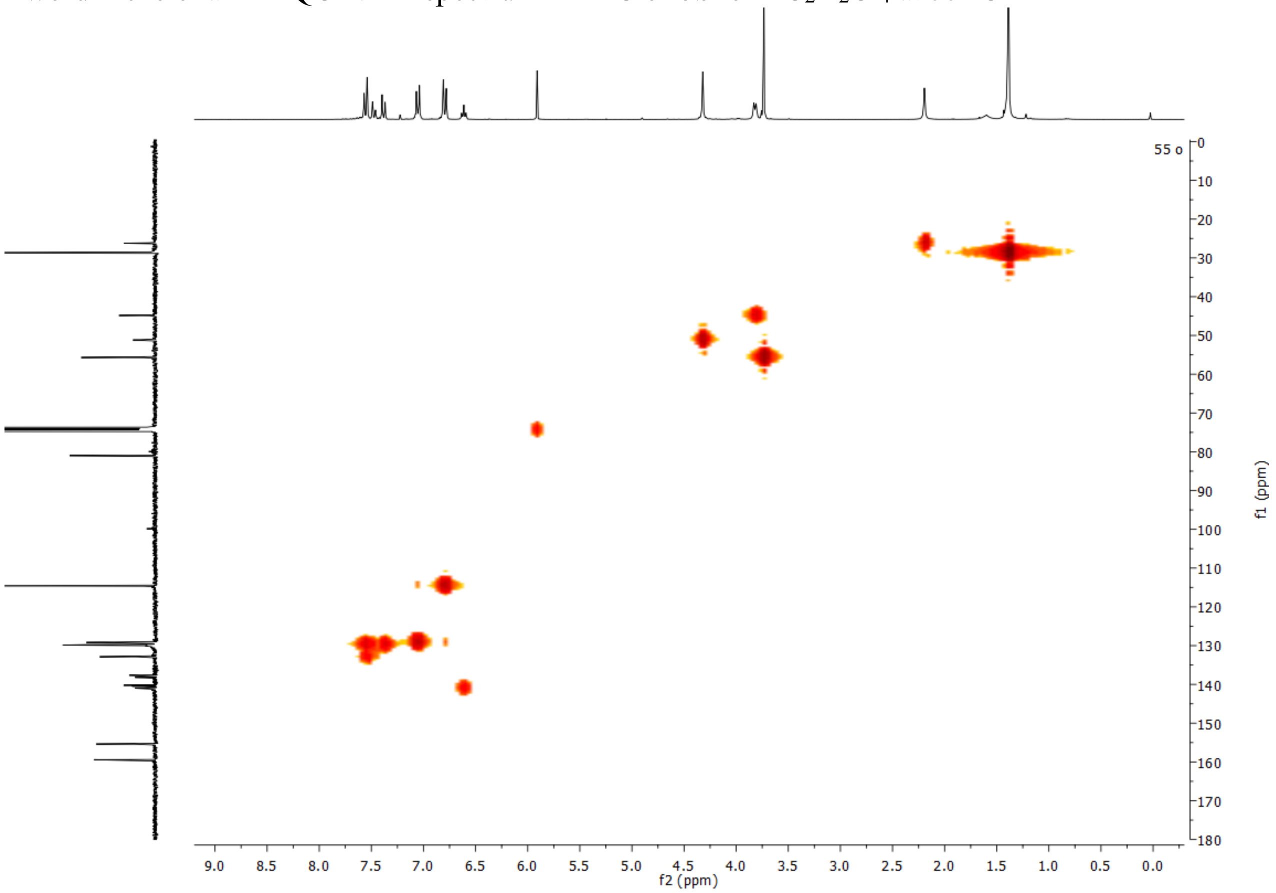

Two-dimensional HMQC NMR spectrum ^{1}H-^{13}C of **7bae** in $C_2D_2Cl_4$ at 55 ºC

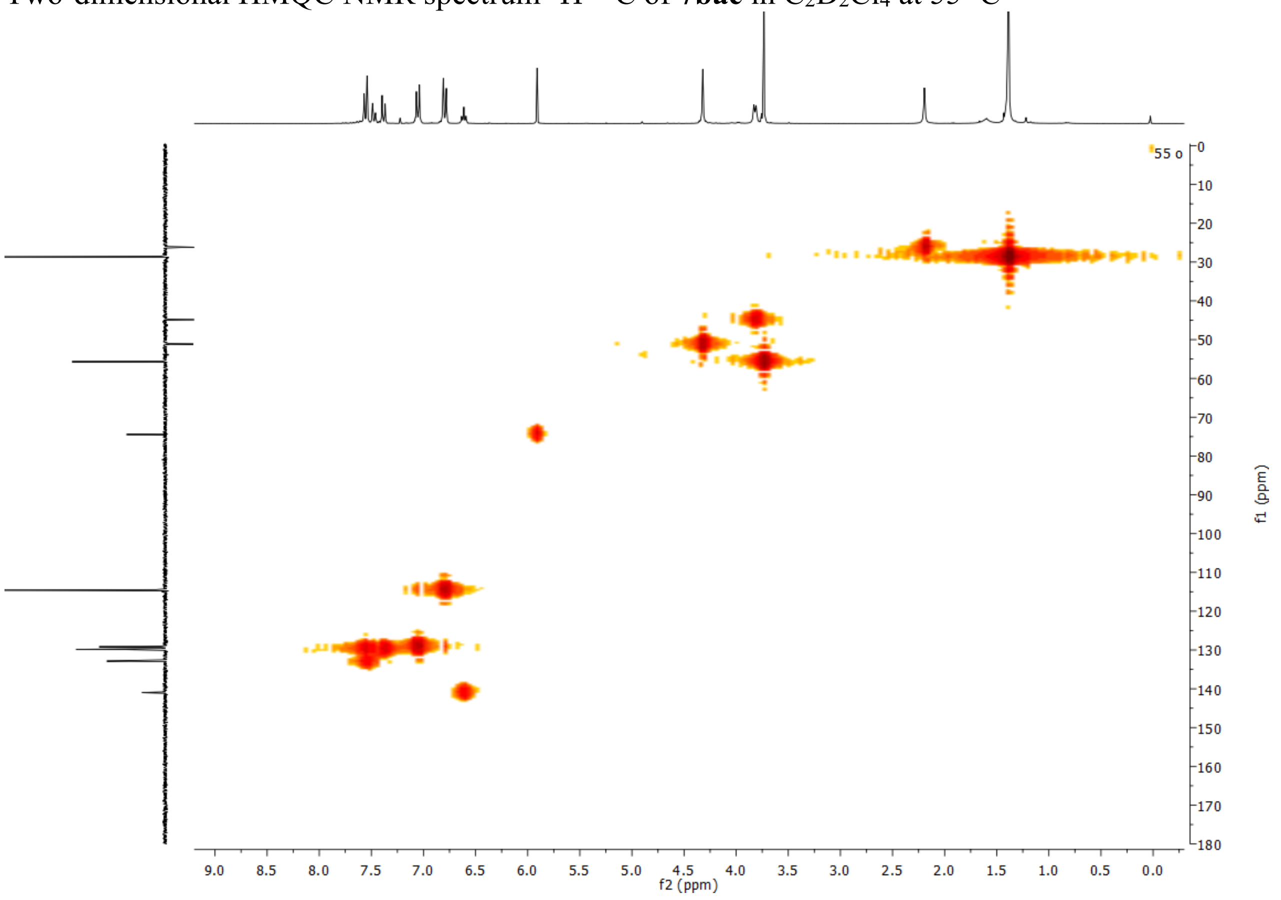

Two-dimensional HMBC NMR spectrum ^{1}H-^{13}C of **7bae** in $C_2D_2Cl_4$ at 55 ºC

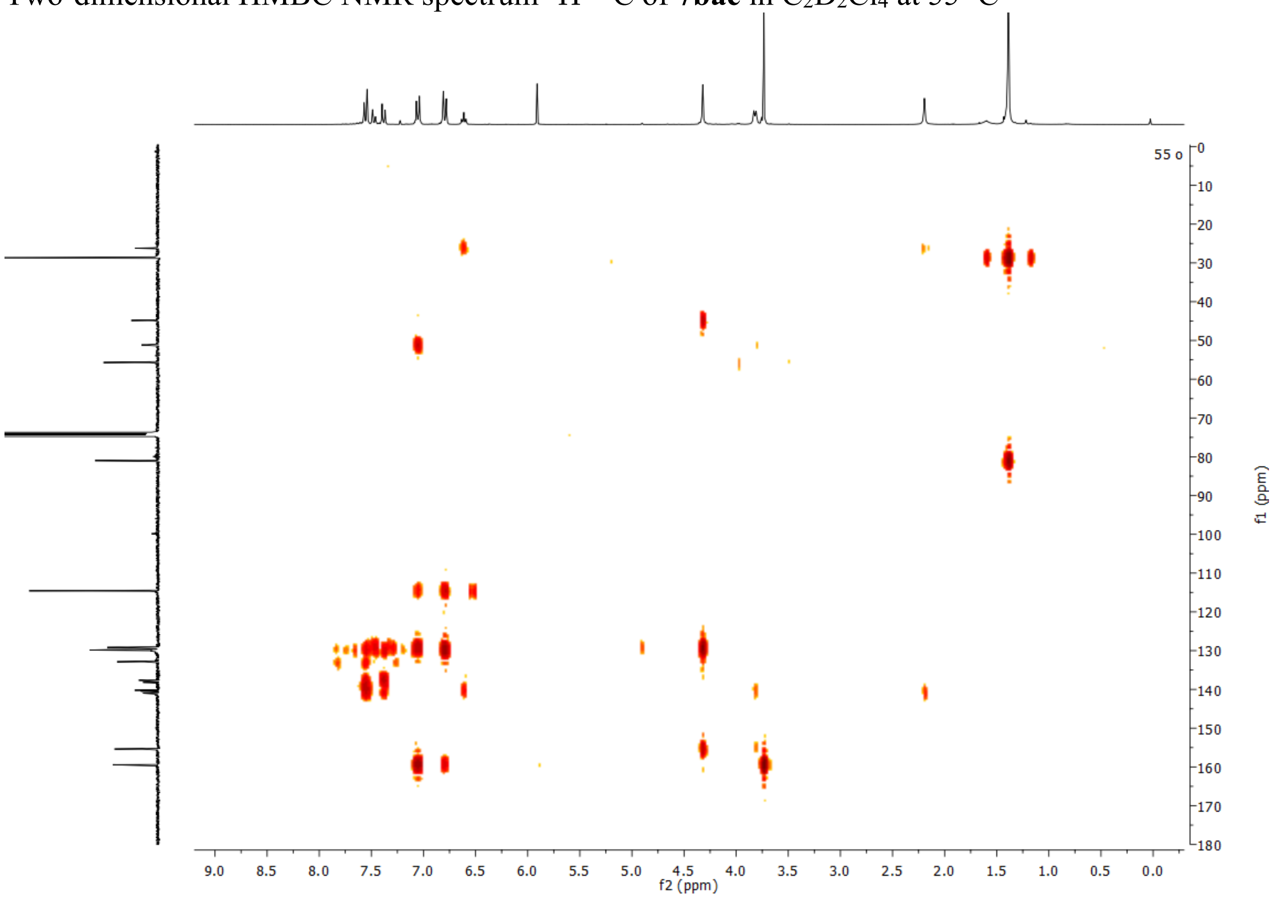

^{1}H NMR (300 MHz) spectrum of **7dae** in $C_2D_2Cl_4$ at 65 ºC

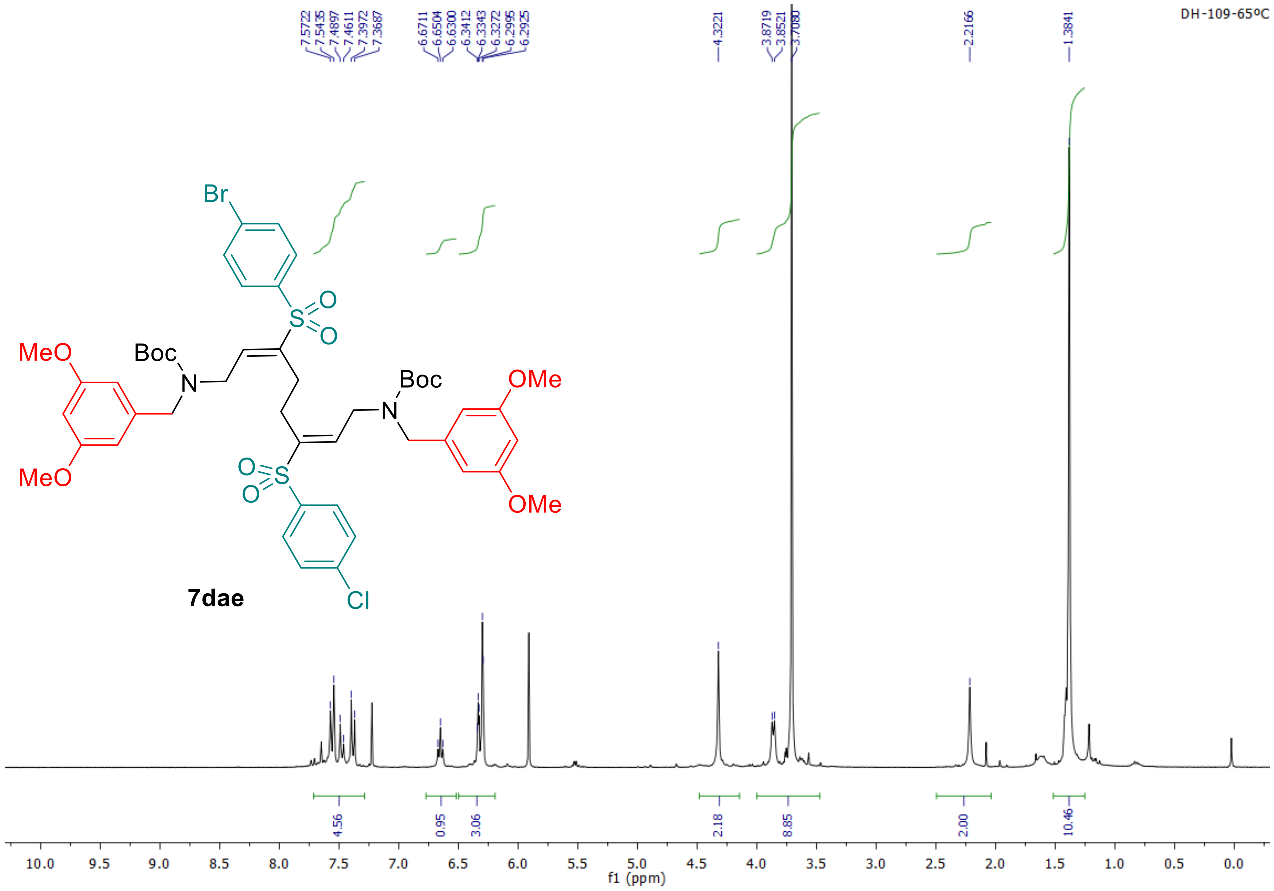

^{13}C NMR (75 MHz) spectrum of **7dae** in $C_2D_2Cl_4$ at 55 ºC

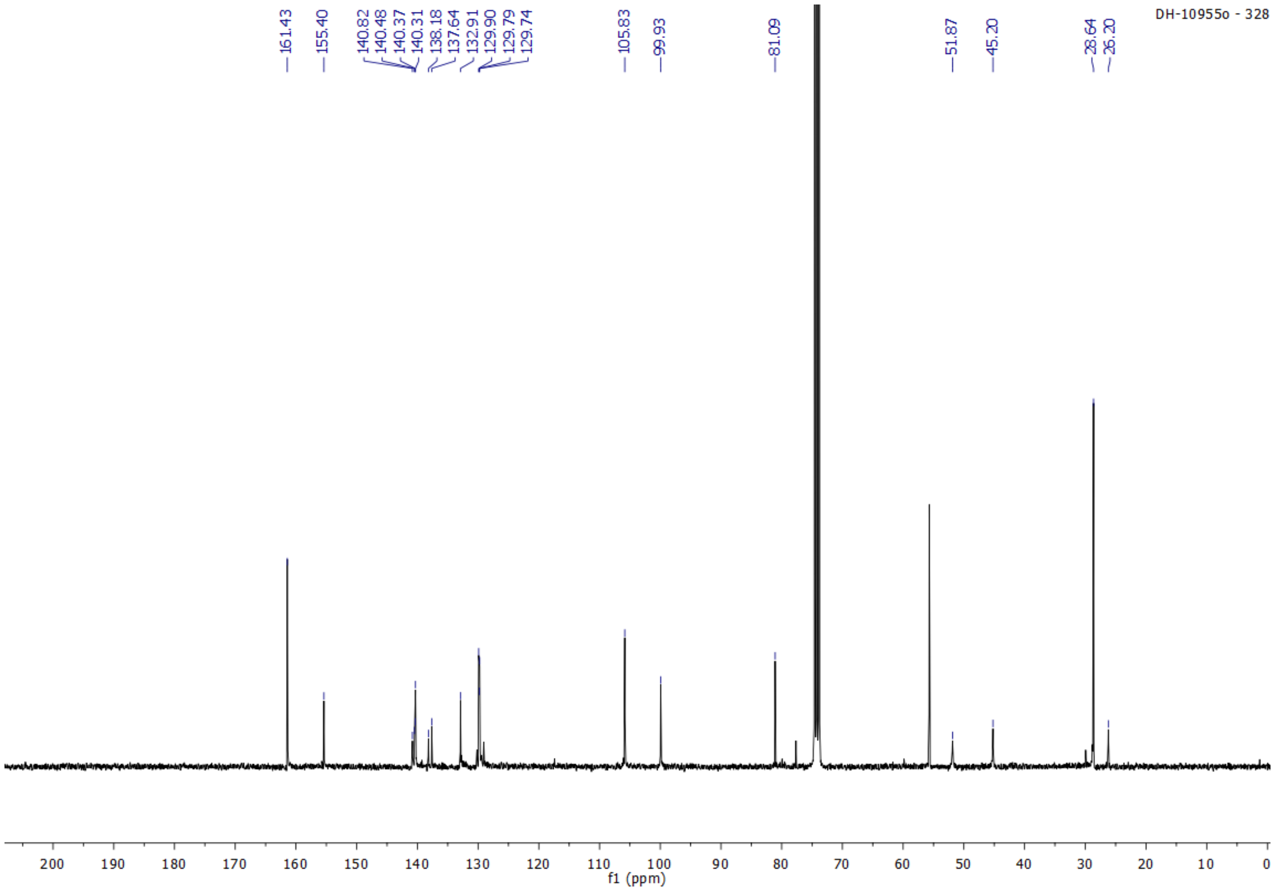

DEPT NMR (75 MHz) spectrum of **7dae** in $C_2D_2Cl_4$ at 55 ºC

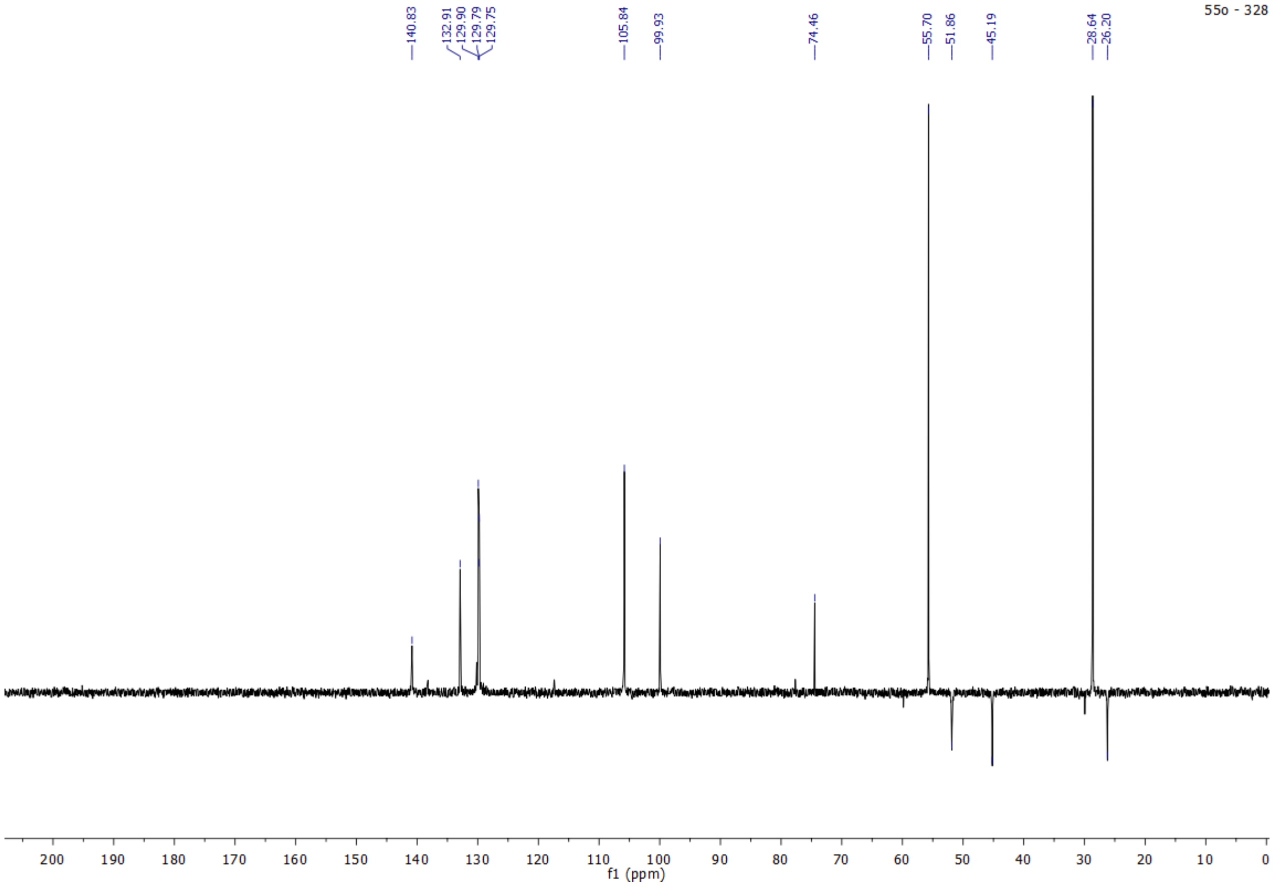

Two-dimensional HMQC NMR spectrum ^1H-^{13}C of **7dae** in $C_2D_2Cl_4$ at 55 ºC

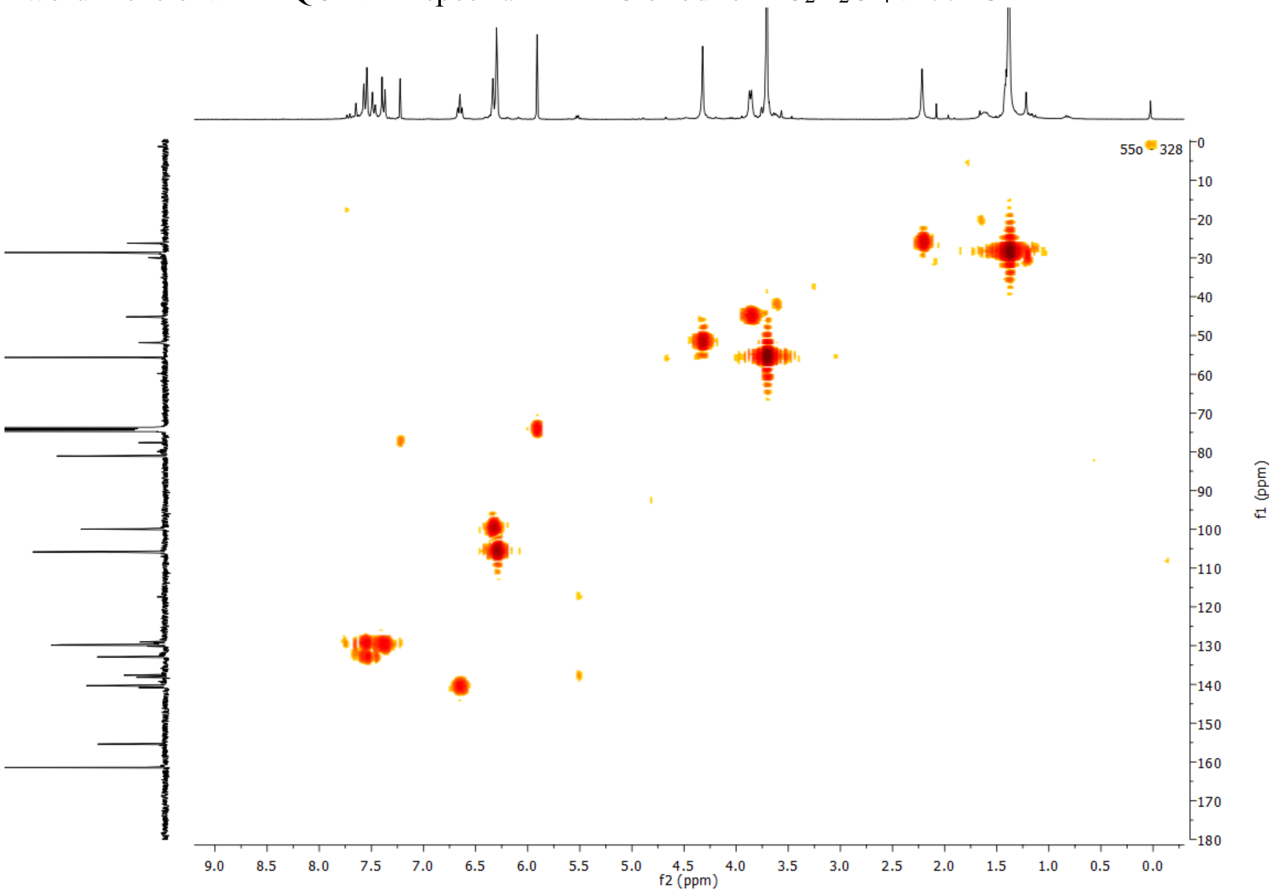

Two-dimensional HMQC NMR spectrum ^{1}H-^{13}C of **7dae** in $C_2D_2Cl_4$ at 55 ºC

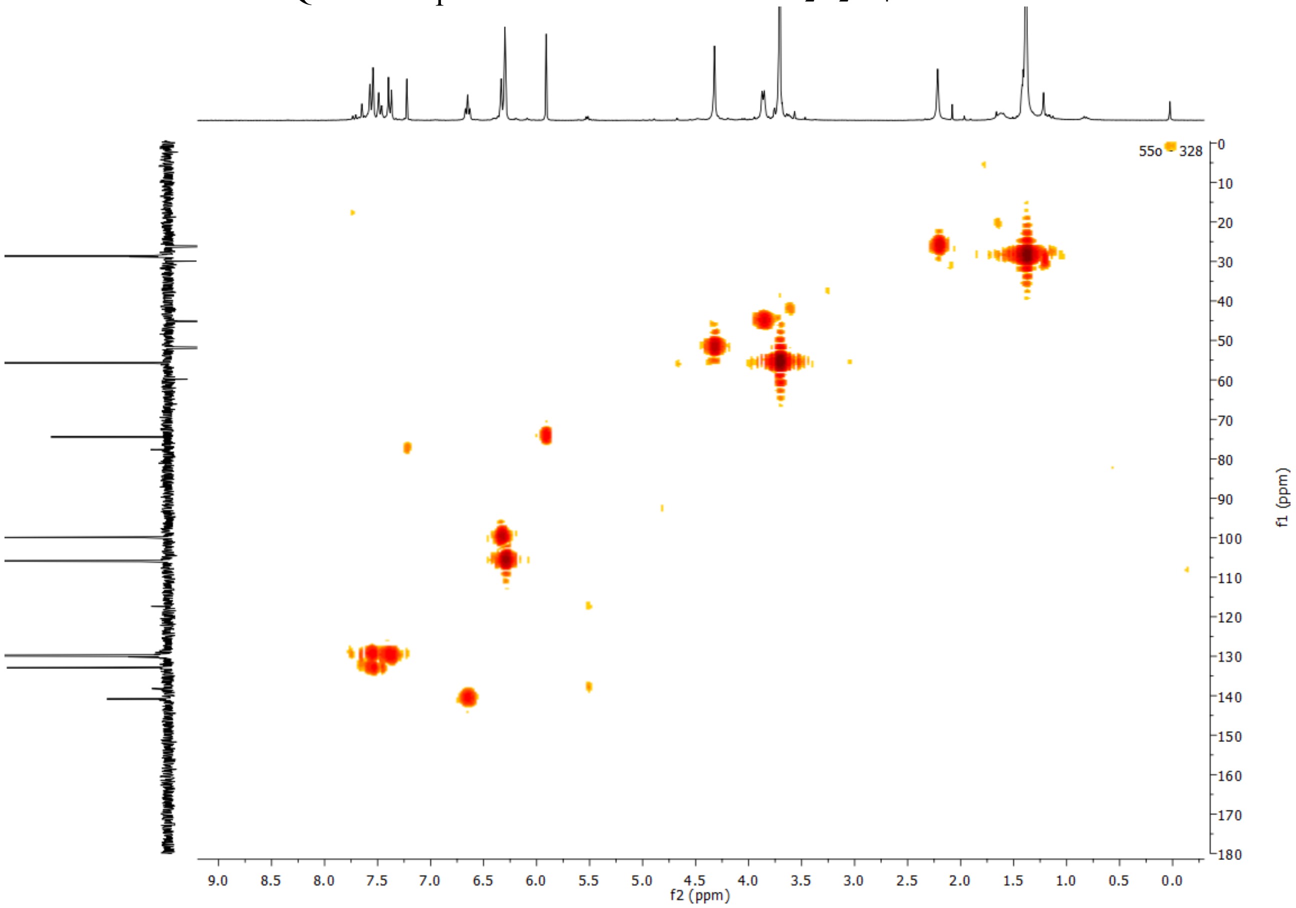

Two-dimensional HMBC NMR spectrum ^{1}H-^{13}C of **7dae** in $C_2D_2Cl_4$ at 55 ºC

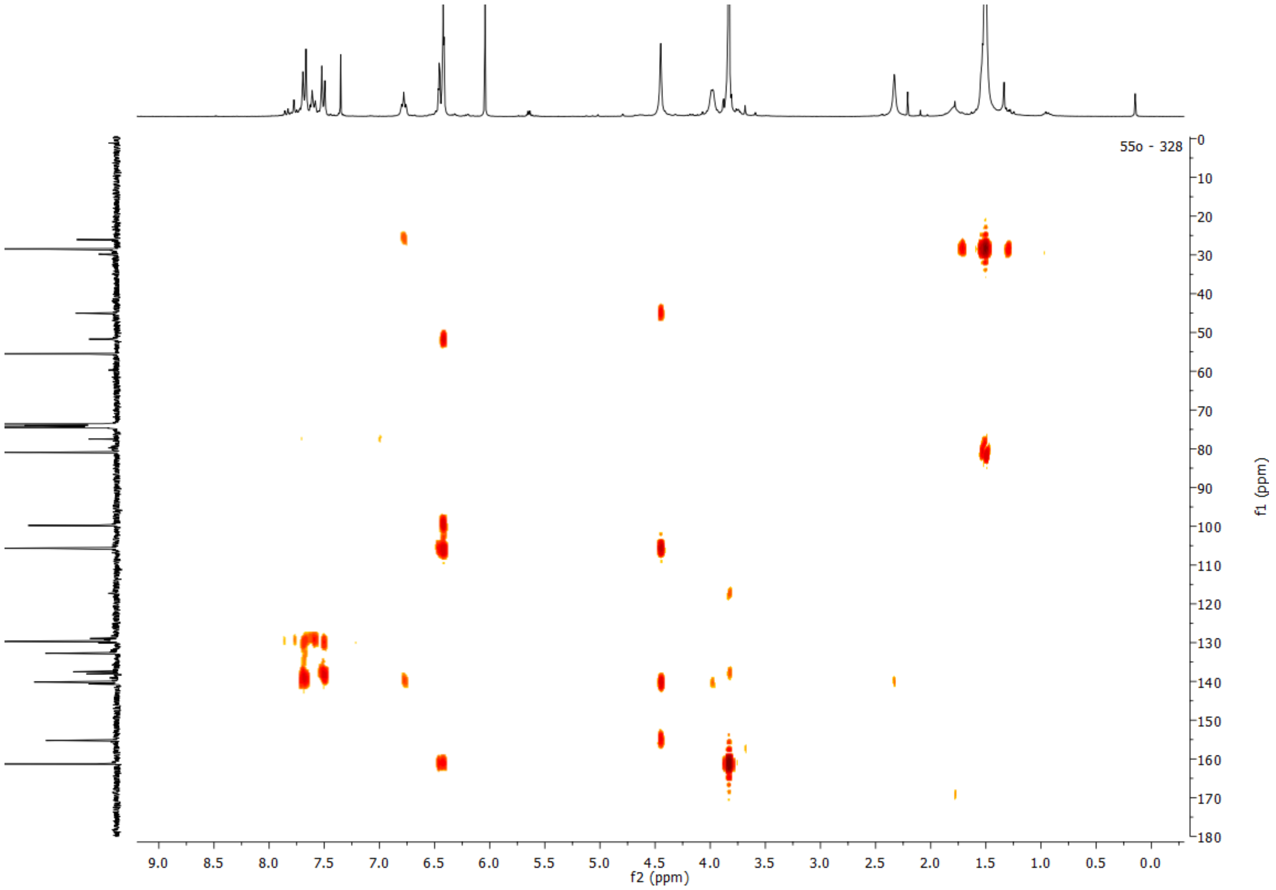

5.- Computational Study

Computational methods

Calculations were performed with Gaussian 16 at DFT level.[1] The geometries of all complexes here reported were optimized using the M06 hybrid functional[2] that accounts for dispersive interactions. Optimizations were carried out using the standard 6-31G(d) basis set for C, H, N, O and S. The LANL2DZ basis set, which includes the relativistic effective core potential (ECP) of Hay and Wadt and employs a split-valence (double-ζ) basis set, was used for Cu and Br.[3] Harmonic frequencies were calculated at the same level to characterize the stationary points and to determine the zero-point energies (ZPE). Gibbs free energy has been used throughout the schemes. The starting approximate geometries for the transition states (TS) were graphically located. Intrinsic reaction coordinate (IRC) studies were performed to confirm the relation of the transition states with the corresponding minima. Solvent effects were considered by performing optimizations in nitromethane or acetonitrile using the polarized continuum model (PCM). Scan of the potential energy surface was performed as implemented in Gaussian by elongation of the critical bond being involved in the elementary steps that are considered barrierless to support the absence of a transition state.

---

[1] Gaussian 09, Revision E.01, M. J. Frisch, G. W. Trucks, H. B. Schlegel, G. E. Scuseria, M. A. Robb, J. R. Cheeseman, G. Scalmani, V. Barone, B. Mennucci, G. A. Petersson, H. Nakatsuji, M. Caricato, X. Li, H. P. Hratchian, A. F. Izmaylov, J. Bloino, G. Zheng, J. L. Sonnenberg, M. Hada, M. Ehara, K. Toyota, R. Fukuda, J. Hasegawa, M. Ishida, T. Nakajima, Y. Honda, O. Kitao, H. Nakai, T. Vreven, J. A. Montgomery, Jr., J. E. Peralta, F. Ogliaro, M. Bearpark, J. J. Heyd, E. Brothers, K. N. Kudin, V. N. Staroverov, T. Keith, R. Kobayashi, J. Normand, K. Raghavachari, A. Rendell, J. C. Burant, S. S. Iyengar, J. Tomasi, M. Cossi, N. Rega, J. M. Millam, M. Klene, J. E. Knox, J. B. Cross, V. Bakken, C. Adamo, J. Jaramillo, R. Gomperts, R. E. Stratmann, O. Yazyev, A. J. Austin, R. Cammi, C. Pomelli, J. W. Ochterski, R. L. Martin, K. Morokuma, V. G. Zakrzewski, G. A. Voth, P. Salvador, J. J. Dannenberg, S. Dapprich, A. D. Daniels, O. Farkas, J. B. Foresman, J. V. Ortiz, J. Cioslowski, and D. J. Fox, Gaussian, Inc., Wallingford CT, 2013.

[2] Y. Zhao, D.G. Truhlar, *Theor Chem Account.* **2006**, *120*: 215–241.

[3] (a) A. D, Becke, *J. Chem. Phys*. **1993**, *98*, 5648- 5653. (b) A. D. Becke, *Phys. Rev. A* **1988**, *38*, 3098-3100. (c) C. Lee, W. Yang, R. G. Parr, *Phys. Rev. B* **1988**, *37*, 785-789.

Bromoheterocyclization of Allenes

Energy profile

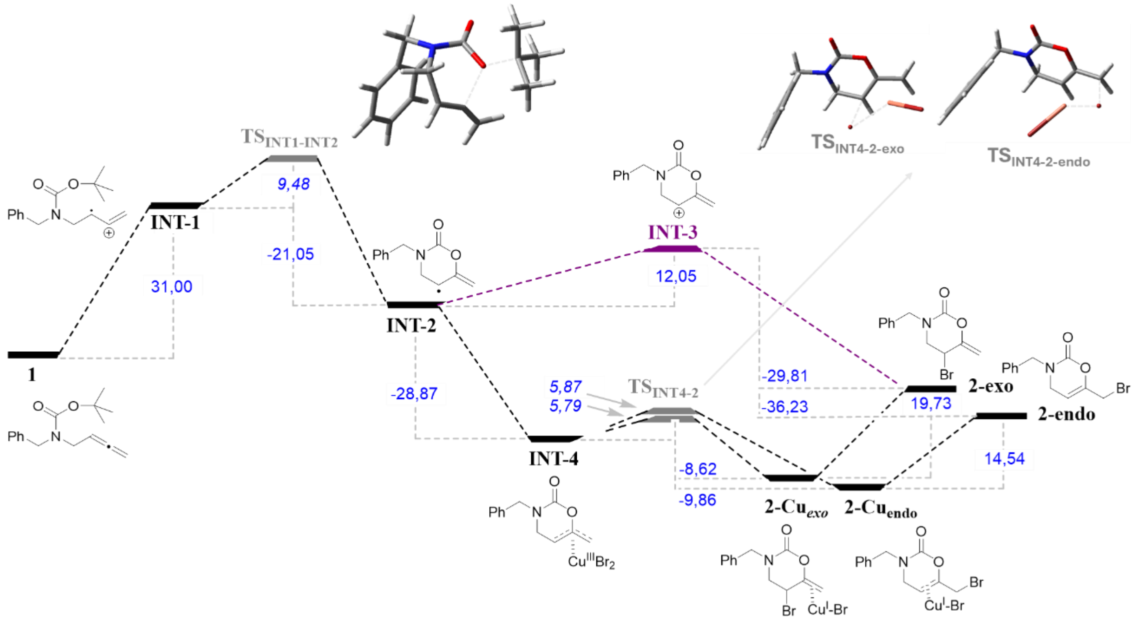

Scheme S1. *Energy profile for the formation of compounds **2** from allene **1a** (ΔG in kcal mol^{-1}, $ΔG_a$ in italics), calculated at M06/6-31G(d) (C,H,N,O,S), LANL2DZ (Cu) level in CH_3NO_2 (PCM).*

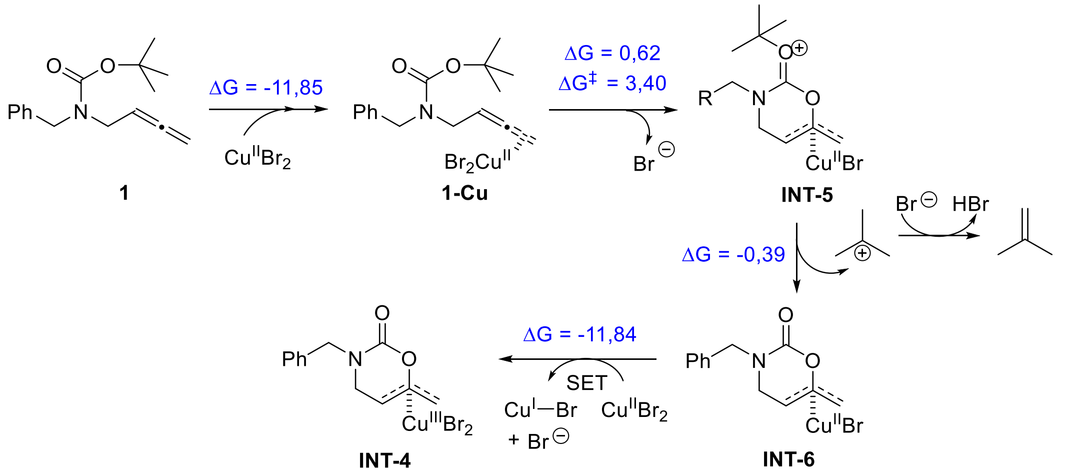

Scheme S2. *Alternative reaction mechanism for the $CuBr_2$-promoted formation of **INT-4** and DFT analysis including reaction free energies and activation energies (kcal/mol) calculated at M06/6-31G(d) (C,H,N,O), LANL2DZ (Cu,Br) level in CH_3NO_2 (PCM).*

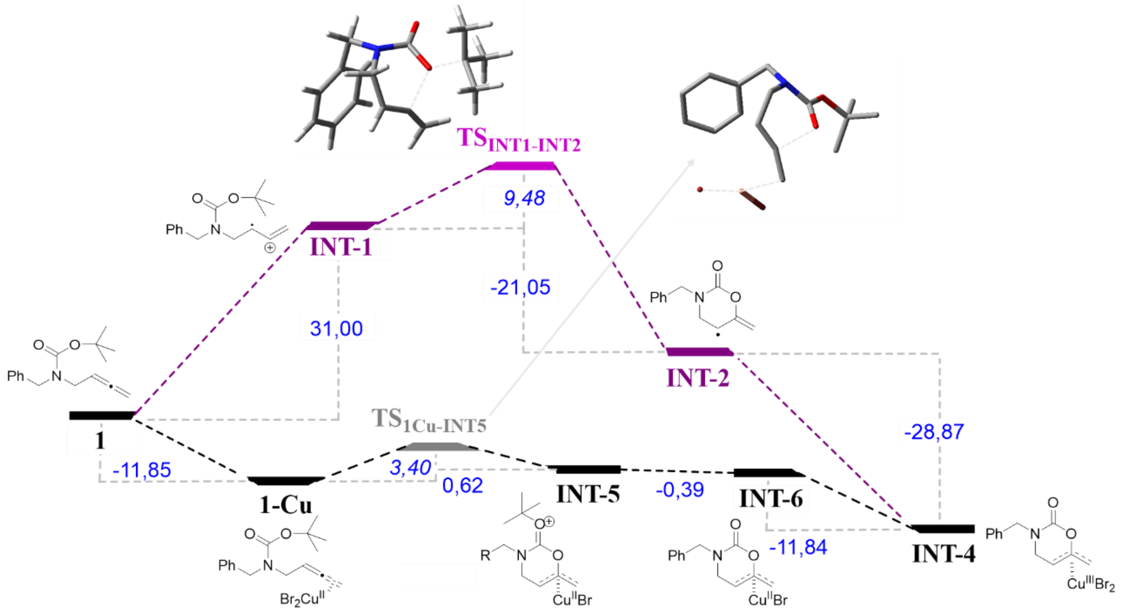

Scheme S3. *Energy profile for the alternative mechanism for the formation of **INT-4** (ΔG in kcal mol^{-1}, ΔG$_a$ in italics), calculated at M06/6-31G(d) (C,H,N,O,S), LANL2DZ (Cu) level in CH_3NO_2 (PCM).*

Atomic coordinates and energies for the stationary points

1

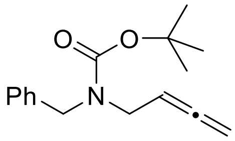

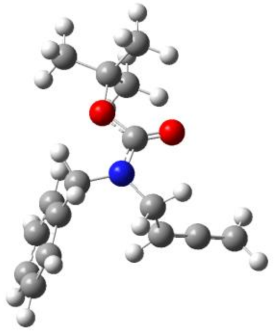

Center Number	Atomic Number	Atomic Type	Coordinates (Angstroms) X	Y	Z
1	6	0	-0.717489	1.772049	-1.324179
2	6	0	-1.438740	2.113699	-0.043056
3	1	0	-0.039213	2.587864	-1.595978
4	1	0	-1.441688	1.647709	-2.141818
5	1	0	-2.210941	1.417647	0.297020
6	7	0	0.046481	0.531143	-1.246303
7	6	0	-0.619460	-0.707043	-1.642535
8	1	0	0.144869	-1.482981	-1.740081
9	6	0	-1.696558	-1.125982	-0.674250
10	1	0	-1.054391	-0.547660	-2.639956
11	8	0	1.753699	1.617579	-0.201966
12	8	0	1.764684	-0.650881	-0.425022
13	6	0	3.028525	-0.850613	0.280612
14	6	0	3.192143	-2.359984	0.245101
15	1	0	3.237074	-2.721385	-0.790141
16	1	0	4.119905	-2.648719	0.754596
17	1	0	2.350397	-2.852091	0.750283
18	6	0	4.162868	-0.175392	-0.470346
19	1	0	4.183920	-0.516983	-1.513639
20	1	0	4.060308	0.913581	-0.454406

21	1	0	5.119500	-0.446245	-0.004463
22	6	0	2.911170	-0.367163	1.716031
23	1	0	2.798376	0.719471	1.766426
24	1	0	2.048628	-0.838392	2.207225
25	1	0	3.814444	-0.654152	2.270249
26	6	0	1.241699	0.576915	-0.582263
27	6	0	-1.170816	3.181545	0.661605
28	6	0	-0.897042	4.255137	1.351473
29	1	0	-0.140116	4.251552	2.136123
30	1	0	-1.413490	5.198377	1.171949
31	6	0	-3.043038	-1.044368	-1.025393
32	6	0	-4.037287	-1.365348	-0.102704
33	6	0	-3.688468	-1.771900	1.181166
34	6	0	-2.343079	-1.863949	1.537767
35	6	0	-1.354358	-1.543617	0.615496
36	1	0	-3.315343	-0.715519	-2.029713
37	1	0	-5.085674	-1.293065	-0.387621
38	1	0	-4.462900	-2.019701	1.905151
39	1	0	-2.066234	-2.187186	2.540074
40	1	0	-0.300284	-1.607626	0.891596

```
Zero-point correction=                           0.338381 (Hartree/Particle)
Thermal correction to Energy=                    0.358002
Thermal correction to Enthalpy=                  0.358946
Thermal correction to Gibbs Free Energy=         0.289320
Sum of electronic and zero-point Energies=          -826.580110
Sum of electronic and thermal Energies=             -826.560489
Sum of electronic and thermal Enthalpies=           -826.559545
Sum of electronic and thermal Free Energies=        -826.629172
```

Cu(II)Br$_2$

Center Number	Atomic Number	Atomic Type	Coordinates (Angstroms) X	Y	Z
1	29	0	0.000000	0.000000	0.885292
2	35	0	-0.000000	1.960336	-0.366764
3	35	0	-0.000000	-1.960336	-0.366764

```
Zero-point correction=                           0.001270 (Hartree/Particle)
Thermal correction to Energy=                    0.005912
Thermal correction to Enthalpy=                  0.006856
Thermal correction to Gibbs Free Energy=        -0.030933
Sum of electronic and zero-point Energies=          -222.520654
Sum of electronic and thermal Energies=             -222.516012
Sum of electronic and thermal Enthalpies=           -222.515068
Sum of electronic and thermal Free Energies=        -222.552857
```

Cu(I)Br

Center Number	Atomic Number	Atomic Type	Coordinates (Angstroms) X	Y	Z
1	29	0	0.000000	0.000000	-1.258979
2	35	0	0.000000	0.000000	1.043154

```
Zero-point correction=                           0.000624 (Hartree/Particle)
Thermal correction to Energy=                    0.003438
Thermal correction to Enthalpy=                  0.004382
Thermal correction to Gibbs Free Energy=        -0.023976
Sum of electronic and zero-point Energies=          -209.382607
Sum of electronic and thermal Energies=             -209.379793
Sum of electronic and thermal Enthalpies=           -209.378849
```

Sum of electronic and thermal Free Energies= -209.407207

Br⁻

Center Number	Atomic Number	Atomic Type	Coordinates (Angstroms) X	Y	Z
1	35	0	0.000000	0.000000	0.000000

Zero-point correction= 0.000000 (Hartree/Particle)
Thermal correction to Energy= 0.001416
Thermal correction to Enthalpy= 0.002360
Thermal correction to Gibbs Free Energy= -0.016176
Sum of electronic and zero-point Energies= -13.311509
Sum of electronic and thermal Energies= -13.310093
Sum of electronic and thermal Enthalpies= -13.309148
Sum of electronic and thermal Free Energies= -13.327684

INT-1

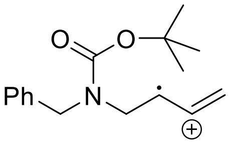

Center Number	Atomic Number	Atomic Type	Coordinates (Angstroms) X	Y	Z
1	6	0	-0.799716	1.710088	-1.375014
2	6	0	-1.406966	2.095367	-0.038518
3	1	0	-0.098310	2.481482	-1.707359
4	1	0	-1.574943	1.569318	-2.135546
5	1	0	-2.248523	1.479962	0.292930
6	7	0	-0.061988	0.462638	-1.259754
7	6	0	-0.663162	-0.787980	-1.702537
8	1	0	0.105293	-1.560097	-1.756438
9	6	0	-1.731235	-1.080494	-0.679081
10	1	0	-1.090725	-0.630896	-2.700137
11	8	0	1.712959	1.604770	-0.374859
12	8	0	1.674965	-0.673583	-0.386648
13	6	0	2.988586	-0.885825	0.286972
14	6	0	3.075808	-2.396566	0.348853
15	1	0	3.053858	-2.831010	-0.657948
16	1	0	4.016292	-2.687330	0.831730
17	1	0	2.245140	-2.813533	0.932503
18	6	0	4.088702	-0.303771	-0.577285
19	1	0	4.042314	-0.719504	-1.591493
20	1	0	4.032413	0.787697	-0.631955
21	1	0	5.057866	-0.577213	-0.141447
22	6	0	2.934179	-0.279991	1.675166
23	1	0	2.867439	0.811906	1.645539
24	1	0	2.079920	-0.678537	2.237903
25	1	0	3.850711	-0.553851	2.212576
26	6	0	1.214578	0.532144	-0.614101
27	6	0	-0.949405	3.068744	0.699633
28	6	0	-0.484824	4.044224	1.430776
29	1	0	0.260363	3.863706	2.205200
30	1	0	-0.824270	5.070502	1.294559
31	6	0	-3.087498	-0.891257	-0.984553
32	6	0	-4.049711	-1.157417	-0.025351
33	6	0	-3.665823	-1.571724	1.257486
34	6	0	-2.317259	-1.734077	1.575896
35	6	0	-1.349208	-1.473595	0.620207

36	1	0	-3.377522	-0.569574	-1.983821
37	1	0	-5.103925	-1.047607	-0.267570
38	1	0	-4.426931	-1.762603	2.010731
39	1	0	-2.025581	-2.056525	2.572200
40	1	0	-0.292615	-1.591475	0.856887

```
Zero-point correction=                           0.336543 (Hartree/Particle)
Thermal correction to Energy=                    0.356378
Thermal correction to Enthalpy=                  0.357323
Thermal correction to Gibbs Free Energy=         0.286919
Sum of electronic and zero-point Energies=          -826.348198
Sum of electronic and thermal Energies=             -826.328362
Sum of electronic and thermal Enthalpies=           -826.327418
Sum of electronic and thermal Free Energies=        -826.397822
```

INT-2

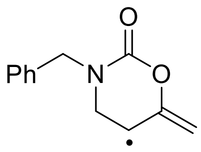

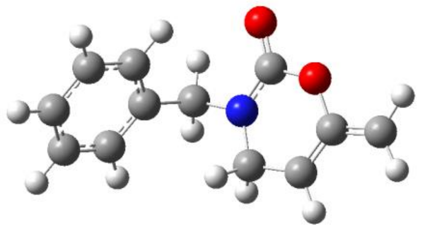

Center Number	Atomic Number	Atomic Type	Coordinates (Angstroms) X	Y	Z
1	6	0	0.976355	-1.390386	-0.401267
2	6	0	2.314040	-1.555611	0.201911
3	1	0	0.180177	-1.722815	0.292256
4	1	0	0.869914	-2.024499	-1.298401
5	1	0	2.687504	-2.555223	0.404569
6	7	0	0.733020	-0.008927	-0.791210
7	6	0	-0.526817	0.234967	-1.490900
8	1	0	-0.468766	1.241028	-1.917285
9	1	0	-0.587762	-0.483741	-2.321085
10	6	0	-1.725286	0.094947	-0.589238
11	8	0	2.616616	0.813572	0.296015
12	8	0	1.158841	2.221276	-0.571528
13	6	0	1.458484	1.059218	-0.374372
14	6	0	3.096727	-0.468646	0.529449
15	6	0	4.351874	-0.519219	1.096269
16	1	0	4.899335	0.391837	1.317920
17	1	0	4.801076	-1.480076	1.328023
18	6	0	-1.962866	1.046183	0.407464
19	6	0	-3.054118	0.915475	1.258322
20	6	0	-3.920719	-0.168628	1.122701
21	6	0	-3.689490	-1.120684	0.134924
22	6	0	-2.593735	-0.988484	-0.715545
23	1	0	-1.283103	1.893459	0.504567
24	1	0	-3.233828	1.663713	2.028667
25	1	0	-4.776455	-0.269054	1.788240
26	1	0	-4.361339	-1.970474	0.026132
27	1	0	-2.408262	-1.735237	-1.489329

```
Zero-point correction=                           0.215438 (Hartree/Particle)
Thermal correction to Energy=                    0.228397
Thermal correction to Enthalpy=                  0.229341
Thermal correction to Gibbs Free Energy=         0.173099
Sum of electronic and zero-point Energies=          -668.982926
Sum of electronic and thermal Energies=             -668.969967
Sum of electronic and thermal Enthalpies=           -668.969023
Sum of electronic and thermal Free Energies=        -669.025265
```

TS$_{INT1-INT2}$

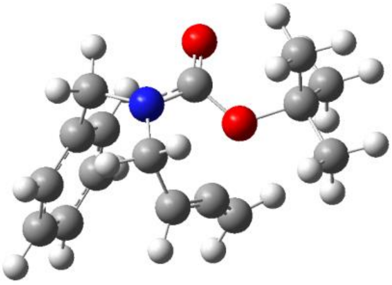

Imaginary frequency = -333.3489

Center Number	Atomic Number	Atomic Type	Coordinates (Angstroms) X	Y	Z
1	6	0	0.018616	-1.123558	1.745136
2	6	0	0.222035	0.346493	1.919253
3	1	0	-0.937189	-1.402497	2.216022
4	1	0	0.799067	-1.680207	2.272974
5	1	0	0.692411	0.750552	2.816865
6	7	0	0.017966	-1.530426	0.347512
7	6	0	1.279485	-2.054674	-0.213775
8	1	0	1.037132	-2.499464	-1.183863
9	6	0	2.272456	-0.930190	-0.336032
10	1	0	1.634566	-2.853178	0.447567
11	8	0	-1.028416	-1.234482	-1.681294
12	8	0	-1.554471	0.047104	0.138137
13	6	0	-3.014793	0.326015	-0.138186
14	6	0	-3.436106	1.199639	1.023018
15	1	0	-2.921776	2.168306	1.022009
16	1	0	-4.511778	1.396547	0.942627
17	1	0	-3.248101	0.696120	1.980194
18	6	0	-3.160573	1.019235	-1.474943
19	1	0	-2.634203	1.980766	-1.496667
20	1	0	-2.790594	0.392373	-2.291153
21	1	0	-4.225275	1.224936	-1.643762
22	6	0	-3.721163	-1.013999	-0.093566
23	1	0	-3.423380	-1.663064	-0.923531
24	1	0	-3.527826	-1.525480	0.858524
25	1	0	-4.801264	-0.839468	-0.171372
26	6	0	-0.864673	-0.962541	-0.517093
27	6	0	-0.233837	1.215885	0.989816
28	6	0	-0.143516	2.336929	0.285082
29	1	0	0.753879	2.940704	0.433549
30	1	0	-0.895345	2.689450	-0.413871
31	6	0	3.171054	-0.651980	0.695954
32	6	0	3.971311	0.487790	0.647076
33	6	0	3.882701	1.354467	-0.437694
34	6	0	3.001567	1.073451	-1.481129
35	6	0	2.201072	-0.062502	-1.430062
36	1	0	3.246876	-1.332786	1.545077
37	1	0	4.666324	0.695782	1.458418
38	1	0	4.506464	2.245572	-0.475806
39	1	0	2.939393	1.742162	-2.337725
40	1	0	1.504147	-0.277897	-2.240526

Zero-point correction= 0.335878 (Hartree/Particle)
Thermal correction to Energy= 0.354678
Thermal correction to Enthalpy= 0.355623
Thermal correction to Gibbs Free Energy= 0.288519

Sum of electronic and zero-point Energies= -826.335364
Sum of electronic and thermal Energies= -826.316563
Sum of electronic and thermal Enthalpies= -826.315619
Sum of electronic and thermal Free Energies= -826.382723

Cation 2-methylpropan-2-ylium

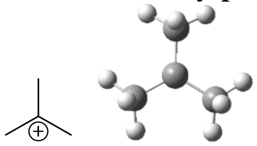

Center Number	Atomic Number	Atomic Type	Coordinates (Angstroms) X	Y	Z
1	6	0	0.000357	0.000409	-0.065831
2	6	0	1.364525	0.512064	-0.013948
3	6	0	-0.238385	-1.436698	-0.013872
4	6	0	-1.125933	0.924886	-0.013993
5	1	0	-1.311196	1.076660	1.068185
6	1	0	-2.047837	0.505950	-0.426386
7	1	0	-0.893693	1.910436	-0.426123
8	1	0	1.595065	0.580747	1.067887
9	1	0	1.461702	1.524880	-0.413834
10	1	0	2.099180	-0.177252	-0.439201
11	1	0	-0.294237	-1.669861	1.068252
12	1	0	0.589954	-2.027485	-0.413483
13	1	0	-1.202326	-1.728044	-0.439431

Zero-point correction= 0.116465 (Hartree/Particle)
Thermal correction to Energy= 0.122218
Thermal correction to Enthalpy= 0.123163
Thermal correction to Gibbs Free Energy= 0.087573
Sum of electronic and zero-point Energies= -157.377217
Sum of electronic and thermal Energies= -157.371464
Sum of electronic and thermal Enthalpies= -157.370520
Sum of electronic and thermal Free Energies= -157.406109

2-methylprop-1-ene

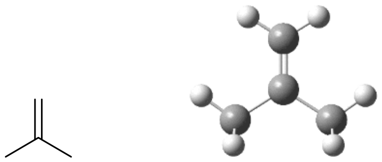

Center Number	Atomic Number	Atomic Type	Coordinates (Angstroms) X	Y	Z
1	6	0	0.000000	0.123319	0.000032
2	6	0	-0.000006	1.458228	0.000009
3	6	0	-1.266311	-0.677096	0.000005
4	6	0	1.266317	-0.677085	0.000005
5	1	0	1.314353	-1.335850	-0.880079
6	1	0	1.313949	-1.336638	0.879514
7	1	0	2.158106	-0.039144	0.000438
8	1	0	0.928400	2.029556	-0.000026
9	1	0	-0.928421	2.029545	-0.000044
10	1	0	-1.314273	-1.335980	-0.879992
11	1	0	-2.158104	-0.039159	0.000284

12	1	0	-1.314005	-1.336528	0.879601

Zero-point correction= 0.107513 (Hartree/Particle)
Thermal correction to Energy= 0.112832
Thermal correction to Enthalpy= 0.113776
Thermal correction to Gibbs Free Energy= 0.080221
Sum of electronic and zero-point Energies= -156.975382
Sum of electronic and thermal Energies= -156.970063
Sum of electronic and thermal Enthalpies= -156.969119
Sum of electronic and thermal Free Energies= -157.002674

HBr

Center Number	Atomic Number	Atomic Type	Coordinates (Angstroms) X	Y	Z
1	35	0	0.000000	0.000000	0.040701
2	1	0	-0.000000	-0.000000	-1.424546

Zero-point correction= 0.005499 (Hartree/Particle)
Thermal correction to Energy= 0.007860
Thermal correction to Enthalpy= 0.008804
Thermal correction to Gibbs Free Energy= -0.013780
Sum of electronic and zero-point Energies= -13.718964
Sum of electronic and thermal Energies= -13.716603
Sum of electronic and thermal Enthalpies= -13.715659
Sum of electronic and thermal Free Energies= -13.738243

INT-3

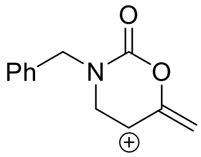

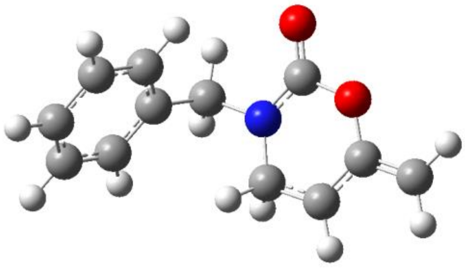

Center Number	Atomic Number	Atomic Type	Coordinates (Angstroms) X	Y	Z
1	6	0	0.944968	-1.227458	-0.659945
2	6	0	2.181299	-1.570674	-0.000820
3	1	0	0.115620	-1.702341	-0.087354
4	1	0	0.905186	-1.744788	-1.639083
5	1	0	2.462556	-2.615901	0.108363
6	7	0	0.732919	0.189082	-0.808228
7	6	0	-0.538427	0.571036	-1.443557
8	1	0	-0.474132	1.642883	-1.650376
9	1	0	-0.596953	0.042366	-2.404880
10	6	0	-1.715941	0.236968	-0.570024
11	8	0	2.665800	0.736314	0.382404
12	8	0	1.298696	2.340331	-0.289275
13	6	0	1.510370	1.155721	-0.259833
14	6	0	3.005331	-0.570686	0.496374
15	6	0	4.180686	-0.906051	1.111479
16	1	0	4.851777	-0.141509	1.497017
17	1	0	4.458909	-1.950194	1.222185
18	6	0	-1.995183	1.018091	0.555413
19	6	0	-3.062978	0.696041	1.384024

20	6	0	-3.860040	-0.412308	1.097950
21	6	0	-3.586187	-1.196054	-0.017893
22	6	0	-2.516067	-0.871666	-0.848882
23	1	0	-1.371355	1.886305	0.771785
24	1	0	-3.278868	1.313215	2.254288
25	1	0	-4.697107	-0.661598	1.747563
26	1	0	-4.206041	-2.061502	-0.244338
27	1	0	-2.302969	-1.481436	-1.728321

Zero-point correction= 0.216760 (Hartree/Particle)
Thermal correction to Energy= 0.229572
Thermal correction to Enthalpy= 0.230517
Thermal correction to Gibbs Free Energy= 0.175629
Sum of electronic and zero-point Energies= -668.782897
Sum of electronic and thermal Energies= -668.770084
Sum of electronic and thermal Enthalpies= -668.769140
Sum of electronic and thermal Free Energies= -668.824028

2-*exo*

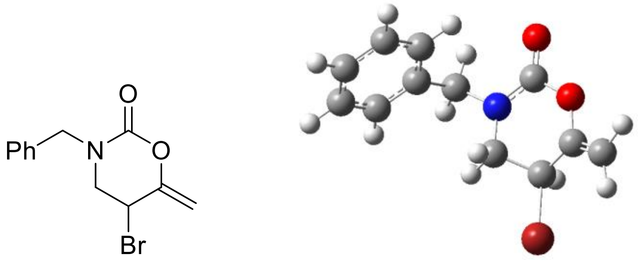

Center Number	Atomic Number	Atomic Type	Coordinates (Angstroms) X	Y	Z
1	6	0	0.434337	-0.298139	-0.507722
2	6	0	1.891129	0.102097	-0.439433
3	1	0	0.114054	-0.751546	0.444033
4	1	0	0.274975	-1.035333	-1.304957
5	1	0	2.248862	0.411989	-1.428699
6	7	0	-0.343084	0.888628	-0.821957
7	6	0	-1.641531	0.682203	-1.463800
8	1	0	-2.015712	1.672858	-1.740892
9	1	0	-1.473771	0.111468	-2.387982
10	6	0	-2.611538	-0.040155	-0.566912
11	8	0	1.154899	2.244926	0.323605
12	8	0	-0.704866	3.120105	-0.483062
13	6	0	-0.028386	2.122329	-0.347319
14	6	0	2.047161	1.206972	0.551137
15	6	0	2.911064	1.344767	1.547688
16	1	0	2.881132	2.233999	2.170897
17	1	0	3.657221	0.585662	1.752607
18	35	0	2.981013	-1.514800	0.005289
19	6	0	-3.102218	0.590499	0.580046
20	6	0	-3.980867	-0.074367	1.426878
21	6	0	-4.378820	-1.379018	1.136084
22	6	0	-3.892851	-2.013843	-0.002199
23	6	0	-3.009650	-1.346061	-0.848168
24	1	0	-2.793501	1.613298	0.800256
25	1	0	-4.361416	0.426552	2.315487
26	1	0	-5.068739	-1.898802	1.798685
27	1	0	-4.198540	-3.032956	-0.232872
28	1	0	-2.625663	-1.843556	-1.740010

Zero-point correction= 0.219994 (Hartree/Particle)
Thermal correction to Energy= 0.234170

Thermal correction to Enthalpy= 0.235114
Thermal correction to Gibbs Free Energy= 0.175690
Sum of electronic and zero-point Energies= -682.154918
Sum of electronic and thermal Energies= -682.140741
Sum of electronic and thermal Enthalpies= -682.139797
Sum of electronic and thermal Free Energies= -682.199222

2-*endo*

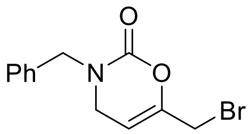

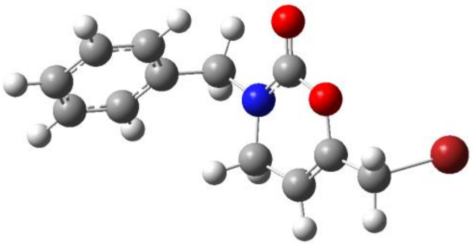

Center Number	Atomic Number	Atomic Type	Coordinates (Angstroms) X	Y	Z
1	6	0	-0.450715	-1.402364	-0.448929
2	6	0	0.873053	-1.569878	0.194877
3	1	0	-1.260567	-1.774058	0.203881
4	1	0	-0.509426	-1.997298	-1.375425
5	7	0	-0.697693	-0.005437	-0.785938
6	6	0	-1.951986	0.256770	-1.491417
7	1	0	-1.886088	1.268678	-1.902489
8	1	0	-2.009814	-0.449265	-2.332395
9	6	0	-3.155119	0.109344	-0.597486
10	8	0	1.186394	0.781106	0.325744
11	8	0	-0.256049	2.219331	-0.526414
12	6	0	0.028772	1.052301	-0.350827
13	6	0	1.602824	-0.513577	0.542548
14	6	0	-3.402455	1.059064	0.398223
15	6	0	-4.496575	0.922089	1.244267
16	6	0	-5.356411	-0.166791	1.104351
17	6	0	-5.115898	-1.117026	0.117030
18	6	0	-4.017287	-0.978574	-0.728787
19	1	0	-2.728167	1.910261	0.498902
20	1	0	-4.683935	1.669082	2.013944
21	1	0	-6.214557	-0.272267	1.765969
22	1	0	-5.782928	-1.970093	0.004573
23	1	0	-3.824992	-1.723356	-1.502800
24	6	0	2.906671	-0.569473	1.215121
25	1	0	3.173103	-1.582020	1.519465
26	1	0	2.983751	0.130231	2.049994
27	35	0	4.404311	0.025865	-0.040519
28	1	0	1.244602	-2.571915	0.387645

Zero-point correction= 0.220361 (Hartree/Particle)
Thermal correction to Energy= 0.234651
Thermal correction to Enthalpy= 0.235595
Thermal correction to Gibbs Free Energy= 0.175197
Sum of electronic and zero-point Energies= -682.164291
Sum of electronic and thermal Energies= -682.150001
Sum of electronic and thermal Enthalpies= -682.149057
Sum of electronic and thermal Free Energies= -682.209455

Scan of the potential energy surface from 2-exo separating C-Br bond

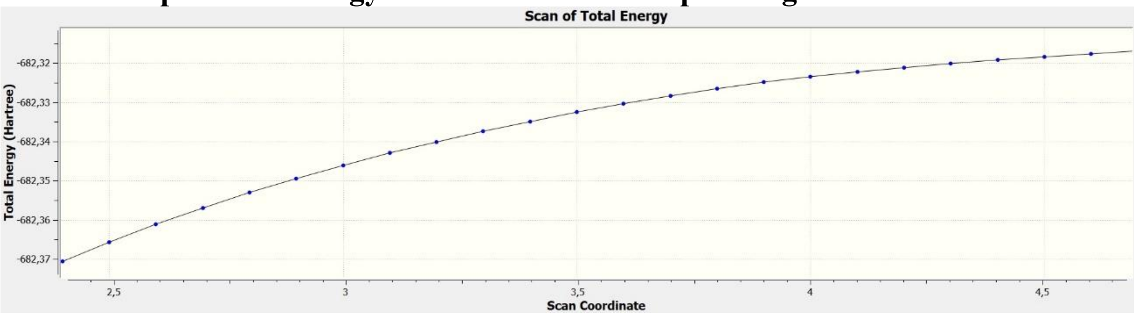

Initial point Final point

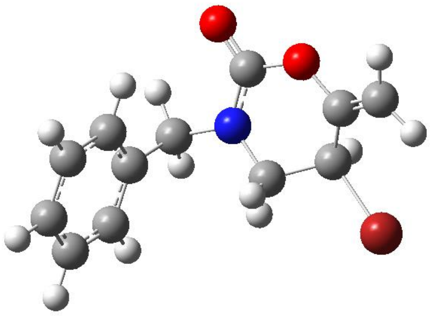

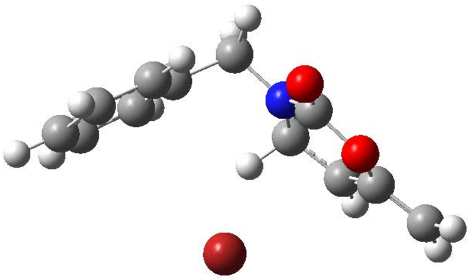

Scan of the potential energy surface from 2-endo separating C-Br bond

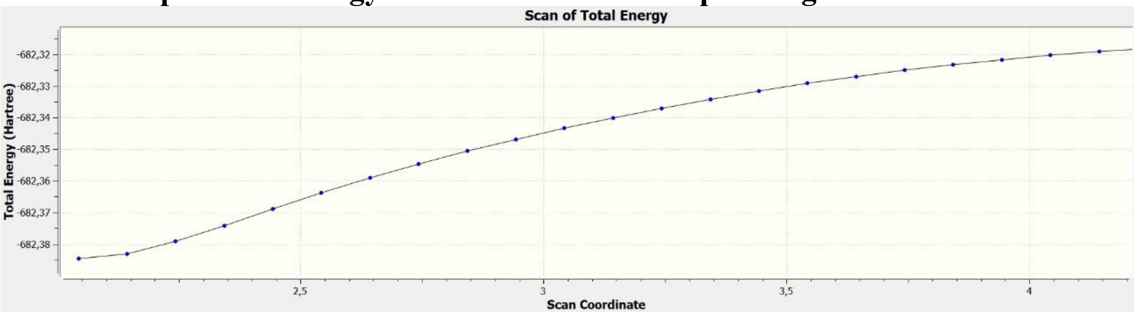

Initial point Final point

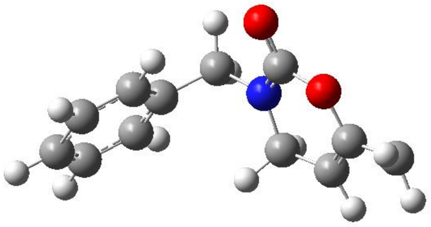

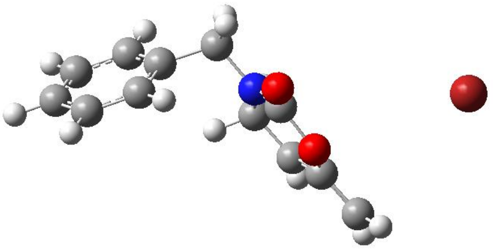

INT-4

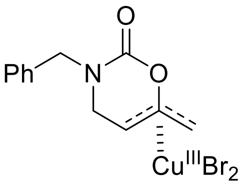

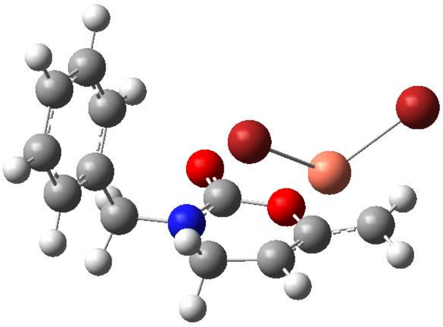

Center Number	Atomic Number	Atomic Type	Coordinates (Angstroms) X	Y	Z
1	6	0	-1.051866	1.411500	-1.521644
2	6	0	0.412324	1.391713	-1.587528
3	1	0	-1.437288	0.388627	-1.682647
4	1	0	-1.433522	2.025349	-2.358535
5	1	0	0.897192	1.055392	-2.503449
6	7	0	-1.542349	1.921058	-0.255622
7	6	0	-2.995852	1.815553	-0.057939
8	1	0	-3.249794	2.474758	0.777280
9	1	0	-3.480141	2.211875	-0.961573
10	6	0	-3.421605	0.397968	0.203266
11	8	0	0.575158	2.599500	0.469372
12	8	0	-1.192442	2.949485	1.755818
13	6	0	-3.112888	-0.203247	1.426301
14	6	0	-3.462774	-1.526417	1.665237
15	6	0	-4.127352	-2.260765	0.682992
16	6	0	-4.440452	-1.667170	-0.535371
17	6	0	-4.088668	-0.340149	-0.773307
18	1	0	-2.590881	0.376419	2.189332
19	1	0	-3.220064	-1.988597	2.620519
20	1	0	-4.401368	-3.297527	0.870311
21	1	0	-4.959587	-2.236395	-1.304441
22	1	0	-4.332599	0.128858	-1.727758
23	6	0	-0.798990	2.502661	0.708106
24	29	0	1.671873	-0.077144	-0.362031
25	35	0	0.052885	-1.803874	-0.567122
26	6	0	1.163929	2.028081	-0.600860
27	6	0	2.553004	1.829916	-0.555507
28	1	0	3.123632	2.178686	0.301287
29	1	0	3.091311	1.609315	-1.476719
30	35	0	3.646276	-0.847747	0.721998

Zero-point correction= 0.220717 (Hartree/Particle)
Thermal correction to Energy= 0.239002
Thermal correction to Enthalpy= 0.239946
Thermal correction to Gibbs Free Energy= 0.170546
Sum of electronic and zero-point Energies= -891.573955
Sum of electronic and thermal Energies= -891.555670
Sum of electronic and thermal Enthalpies= -891.554726
Sum of electronic and thermal Free Energies= -891.624127

2-Cu-exo

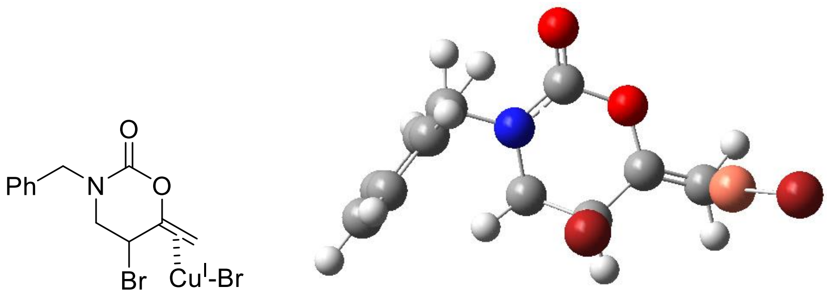

Center Number	Atomic Number	Atomic Type	Coordinates (Angstroms) X	Y	Z
1	6	0	-1.491376	1.717601	-1.230820
2	6	0	-0.126000	1.105710	-1.395367
3	1	0	-2.241477	1.134031	-1.777658
4	1	0	-1.475768	2.731256	-1.663608
5	1	0	0.227146	1.134836	-2.427727
6	7	0	-1.864971	1.757124	0.170577

7	6	0	-3.284137	1.570474	0.506714
8	1	0	-3.425183	1.963097	1.518318
9	1	0	-3.868148	2.195086	-0.182090
10	6	0	-3.688546	0.125882	0.408606
11	8	0	0.358885	2.049611	0.776432
12	8	0	-1.191637	1.872059	2.355473
13	6	0	-3.265813	-0.778986	1.386832
14	6	0	-3.577822	-2.128241	1.276910
15	6	0	-4.314902	-2.587814	0.185638
16	6	0	-4.744445	-1.692397	-0.788233
17	6	0	-4.433016	-0.338771	-0.674821
18	1	0	-2.683684	-0.413781	2.233918
19	1	0	-3.246874	-2.826057	2.044172
20	1	0	-4.557788	-3.645466	0.099029
21	1	0	-5.324565	-2.045559	-1.638974
22	1	0	-4.776017	0.365604	-1.434270
23	6	0	-0.967480	1.870958	1.169359
24	29	0	2.623806	0.392119	-0.147296
25	35	0	-0.197375	-0.872477	-0.929011
26	6	0	0.821089	1.787818	-0.469986
27	6	0	2.055665	2.249104	-0.824510
28	1	0	2.595649	2.913962	-0.151245
29	1	0	2.373733	2.180302	-1.863810
30	35	0	3.972520	-1.360094	0.527991

Zero-point correction= 0.221542 (Hartree/Particle)
Thermal correction to Energy= 0.239773
Thermal correction to Enthalpy= 0.240717
Thermal correction to Gibbs Free Energy= 0.169612
Sum of electronic and zero-point Energies= -891.585936
Sum of electronic and thermal Energies= -891.567706
Sum of electronic and thermal Enthalpies= -891.566762
Sum of electronic and thermal Free Energies= -891.637867

2-Cu-endo

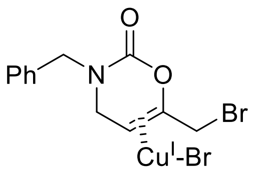

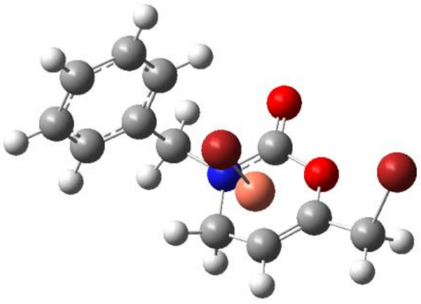

Center Number	Atomic Number	Atomic Type	Coordinates (Angstroms) X	Y	Z
1	6	0	-0.568706	1.851822	-1.678417
2	6	0	0.731494	1.121908	-1.739574
3	1	0	-1.388589	1.210464	-2.030109
4	1	0	-0.548970	2.740090	-2.331808
5	1	0	1.054261	0.678007	-2.681838
6	7	0	-0.862705	2.240975	-0.303546
7	6	0	-2.275393	2.473432	0.016992
8	1	0	-2.304164	3.112630	0.905025
9	1	0	-2.720722	3.034071	-0.816159
10	6	0	-2.997864	1.173415	0.245740
11	8	0	1.375174	2.122246	0.321273
12	8	0	-0.118922	2.929255	1.749339
13	6	0	-2.708369	0.414917	1.384596
14	6	0	-3.352499	-0.796304	1.604535

15	6	0	-4.286249	-1.267837	0.682155
16	6	0	-4.574330	-0.522113	-0.456223
17	6	0	-3.932229	0.696029	-0.672314
18	1	0	-1.979882	0.787987	2.106097
19	1	0	-3.125345	-1.377211	2.496798
20	1	0	-4.790333	-2.217199	0.854333
21	1	0	-5.302844	-0.885846	-1.178696
22	1	0	-4.164029	1.286455	-1.559935
23	6	0	0.065199	2.475902	0.645959
24	29	0	0.468892	-0.643936	-0.683048
25	35	0	-0.214397	-2.769481	-0.105073
26	6	0	1.651404	1.354154	-0.757200
27	6	0	3.070666	0.934368	-0.813057
28	1	0	3.736163	1.793193	-0.690987
29	1	0	3.300641	0.388487	-1.729267
30	35	0	3.519714	-0.300061	0.712094

```
Zero-point correction=                           0.222137 (Hartree/Particle)
Thermal correction to Energy=                    0.240377
Thermal correction to Enthalpy=                  0.241322
Thermal correction to Gibbs Free Energy=         0.170839
Sum of electronic and zero-point Energies=          -891.588540
Sum of electronic and thermal Energies=             -891.570299
Sum of electronic and thermal Enthalpies=           -891.569355
Sum of electronic and thermal Free Energies=        -891.639838
```

$TS_{INT4-2Cu-exo}$

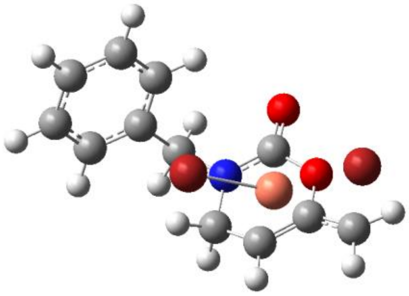

Imaginary frequency = -125.5042

Center Number	Atomic Number	Atomic Type	Coordinates (Angstroms) X	Y	Z
1	6	0	-1.285873	1.535174	-1.445060
2	6	0	0.150103	1.233662	-1.522194
3	1	0	-1.895353	0.719643	-1.853914
4	1	0	-1.431371	2.405230	-2.120108
5	1	0	0.572140	0.919974	-2.474742
6	7	0	-1.716709	1.845036	-0.101614
7	6	0	-3.154346	1.699612	0.174155
8	1	0	-3.353277	2.252009	1.097242
9	1	0	-3.698150	2.196759	-0.640758
10	6	0	-3.552893	0.255101	0.290783
11	8	0	0.450234	2.387399	0.559837
12	8	0	-1.209338	2.523627	2.021050
13	6	0	-3.193344	-0.472693	1.428184
14	6	0	-3.508475	-1.821939	1.526795
15	6	0	-4.183043	-2.458840	0.485157
16	6	0	-4.547868	-1.739855	-0.648183
17	6	0	-4.235892	-0.384948	-0.742632
18	1	0	-2.659101	0.029168	2.236204
19	1	0	-3.227739	-2.381609	2.417349
20	1	0	-4.427396	-3.516951	0.561424
21	1	0	-5.078507	-2.231869	-1.461382

22	1	0	-4.527322	0.182810	-1.627730
23	6	0	-0.893165	2.250129	0.889655
24	29	0	1.970496	-0.004482	-0.199189
25	35	0	-0.090549	-1.314718	-0.681479
26	6	0	0.997015	1.887657	-0.582356
27	6	0	2.363656	2.004778	-0.755607
28	1	0	2.955249	2.559711	-0.031727
29	1	0	2.810445	1.766900	-1.719675
30	35	0	3.952565	-0.996776	0.591945

Zero-point correction= 0.220811 (Hartree/Particle)
Thermal correction to Energy= 0.238445
Thermal correction to Enthalpy= 0.239389
Thermal correction to Gibbs Free Energy= 0.171690
Sum of electronic and zero-point Energies= -891.565778
Sum of electronic and thermal Energies= -891.548144
Sum of electronic and thermal Enthalpies= -891.547200
Sum of electronic and thermal Free Energies= -891.614899

TS$_{INT4-2Cu-endo}$

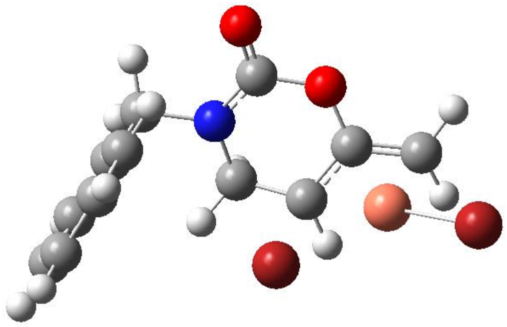

Imaginary frequency = -136.0008

Center Number	Atomic Number	Atomic Type	Coordinates (Angstroms) X	Y	Z
1	6	0	-0.794766	1.438297	-1.689837
2	6	0	0.643612	1.113586	-1.726576
3	1	0	-1.378487	0.518051	-1.856598
4	1	0	-1.045163	2.127003	-2.516126
5	1	0	1.057306	0.627665	-2.609822
6	7	0	-1.181133	2.017514	-0.413710
7	6	0	-2.627764	2.122784	-0.182922
8	1	0	-2.771358	2.874039	0.599569
9	1	0	-3.082911	2.506946	-1.106631
10	6	0	-3.231991	0.800797	0.204255
11	8	0	1.020070	2.394807	0.261978
12	8	0	-0.651254	3.081465	1.538701
13	6	0	-2.945592	0.249367	1.456319
14	6	0	-3.479520	-0.980267	1.821339
15	6	0	-4.306493	-1.672791	0.937012
16	6	0	-4.591824	-1.132420	-0.312479
17	6	0	-4.055487	0.101303	-0.676702
18	1	0	-2.303407	0.796775	2.147899
19	1	0	-3.255312	-1.400163	2.800462
20	1	0	-4.726258	-2.635378	1.223903
21	1	0	-5.234719	-1.669874	-1.007389
22	1	0	-4.281620	0.529056	-1.654581
23	6	0	-0.340215	2.528756	0.511582
24	29	0	1.340669	-0.468854	-0.355832
25	35	0	-0.238361	-2.200424	-0.518580
26	6	0	1.487761	1.643978	-0.772786

27	6	0	2.884007	1.372156	-0.739616
28	1	0	3.507565	1.967597	-0.081096
29	1	0	3.353282	0.928589	-1.614556
30	35	0	3.516896	-0.571564	0.847354

Zero-point correction= 0.219751 (Hartree/Particle)
Thermal correction to Energy= 0.237618
Thermal correction to Enthalpy= 0.238562
Thermal correction to Gibbs Free Energy= 0.169481
Sum of electronic and zero-point Energies= -891.564506
Sum of electronic and thermal Energies= -891.546639
Sum of electronic and thermal Enthalpies= -891.545695
Sum of electronic and thermal Free Energies= -891.614776

1-Cu

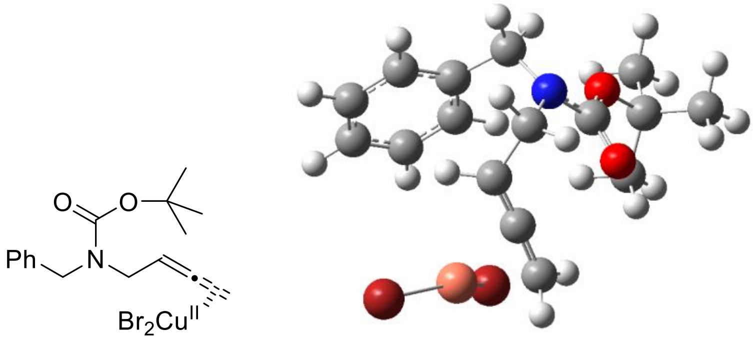

Center Number	Atomic Number	Atomic Type	Coordinates (Angstroms) X	Y	Z
1	6	0	1.058553	2.206821	1.902618
2	6	0	-0.196080	1.433947	1.514473
3	1	0	1.375855	1.936422	2.912435
4	1	0	0.847001	3.280471	1.870589
5	1	0	-0.914287	1.933920	0.859274
6	7	0	2.128187	1.920229	0.964793
7	6	0	2.151206	2.692972	-0.283122
8	1	0	3.043808	2.394464	-0.838898
9	6	0	0.893609	2.491276	-1.091181
10	1	0	2.266409	3.752156	-0.016803
11	8	0	2.380129	-0.082238	2.006799
12	8	0	3.456971	0.356166	0.049511
13	6	0	4.057421	-0.979097	-0.080663
14	6	0	4.763760	-0.888240	-1.420638
15	1	0	5.506181	-0.080456	-1.413710
16	1	0	5.279028	-1.832543	-1.634437
17	1	0	4.043129	-0.694961	-2.226240
18	6	0	5.053081	-1.201701	1.042247
19	1	0	5.799007	-0.396640	1.056933
20	1	0	4.554976	-1.245194	2.015829
21	1	0	5.578776	-2.151017	0.876721
22	6	0	2.975065	-2.044517	-0.123270
23	1	0	2.498841	-2.182711	0.852380
24	1	0	2.203295	-1.785977	-0.862776
25	1	0	3.424602	-2.998494	-0.427918
26	6	0	2.652766	0.652492	1.067458
27	6	0	-0.384688	0.179941	1.787674
28	6	0	-0.615184	-1.130178	1.992054
29	1	0	0.068619	-1.865317	1.558841
30	1	0	-1.267202	-1.464203	2.801753
31	6	0	-0.039941	3.520187	-1.214611
32	6	0	-1.256437	3.302979	-1.860447

33	6	0	-1.547725	2.050165	-2.390740
34	6	0	-0.614158	1.017937	-2.285850
35	6	0	0.598755	1.238137	-1.641219
36	1	0	0.181611	4.498092	-0.784754
37	1	0	-1.978385	4.113360	-1.942813
38	1	0	-2.499235	1.874837	-2.889719
39	1	0	-0.831600	0.036654	-2.706359
40	1	0	1.323241	0.425611	-1.549443
41	29	0	-1.922364	-1.027066	0.263575
42	35	0	-3.754126	0.342848	0.846934
43	35	0	-0.921567	-2.647286	-1.128285

Zero-point correction= 0.340680 (Hartree/Particle)
Thermal correction to Energy= 0.366269
Thermal correction to Enthalpy= 0.367214
Thermal correction to Gibbs Free Energy= 0.280649
Sum of electronic and zero-point Energies= -1049.140863
Sum of electronic and thermal Energies= -1049.115274
Sum of electronic and thermal Enthalpies= -1049.114329
Sum of electronic and thermal Free Energies= -1049.200894

TS$_{1Cu\text{-}INT5}$

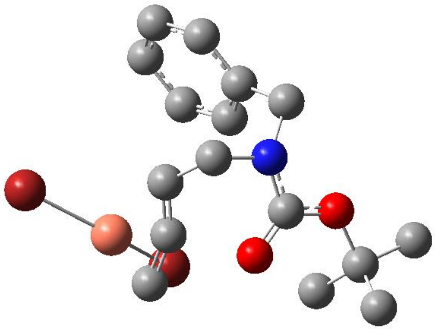

(No H)

Imaginary frequency = -264.4983

Center Number	Atomic Number	Atomic Type	Coordinates (Angstroms) X	Y	Z
1	6	0	-1.036258	2.374633	-1.775946
2	6	0	0.135039	1.436818	-1.641152
3	1	0	-1.445608	2.323147	-2.791881
4	1	0	-0.732450	3.405584	-1.579021
5	1	0	1.108187	1.812036	-1.312250
6	7	0	-2.074731	2.009868	-0.822115
7	6	0	-2.090008	2.727367	0.465242
8	1	0	-2.930131	2.338517	1.047245
9	6	0	-0.771812	2.548978	1.173923
10	1	0	-2.288206	3.785964	0.257658
11	8	0	-2.036873	-0.015618	-1.822656
12	8	0	-3.428463	0.384946	-0.068492
13	6	0	-3.997954	-0.981793	-0.006147
14	6	0	-4.897334	-0.892164	1.211220
15	1	0	-5.646140	-0.100991	1.083610
16	1	0	-5.419193	-1.845909	1.353888
17	1	0	-4.308713	-0.679075	2.112670
18	6	0	-4.800370	-1.238122	-1.265785
19	1	0	-5.566024	-0.463595	-1.398982
20	1	0	-4.159658	-1.266106	-2.153353
21	1	0	-5.306632	-2.207603	-1.175851
22	6	0	-2.899709	-2.004763	0.213663
23	1	0	-2.313848	-2.182832	-0.693046
24	1	0	-2.223811	-1.690823	1.021208
25	1	0	-3.358273	-2.955837	0.512946

26	6	0	-2.510168	0.732964	-0.939762
27	6	0	0.013405	0.140221	-1.787098
28	6	0	0.421037	-1.168331	-1.869499
29	1	0	-0.227881	-1.937561	-1.448474
30	1	0	1.043577	-1.471062	-2.714795
31	6	0	0.141422	3.599113	1.256216
32	6	0	1.404068	3.398638	1.812495
33	6	0	1.762539	2.142711	2.289475
34	6	0	0.852268	1.086864	2.220227
35	6	0	-0.407359	1.291123	1.667924
36	1	0	-0.135400	4.582559	0.874057
37	1	0	2.108436	4.226535	1.867541
38	1	0	2.750541	1.981559	2.717237
39	1	0	1.121729	0.101012	2.598648
40	1	0	-1.115825	0.460529	1.614460
41	29	0	1.803769	-1.087219	-0.248100
42	35	0	3.613650	0.233557	-1.027467
43	35	0	0.874035	-2.658583	1.259907

Zero-point correction= 0.339986 (Hartree/Particle)
Thermal correction to Energy= 0.364781
Thermal correction to Enthalpy= 0.365725
Thermal correction to Gibbs Free Energy= 0.281495
Sum of electronic and zero-point Energies= -1049.136978
Sum of electronic and thermal Energies= -1049.112183
Sum of electronic and thermal Enthalpies= -1049.111239
Sum of electronic and thermal Free Energies= -1049.195469

INT-5

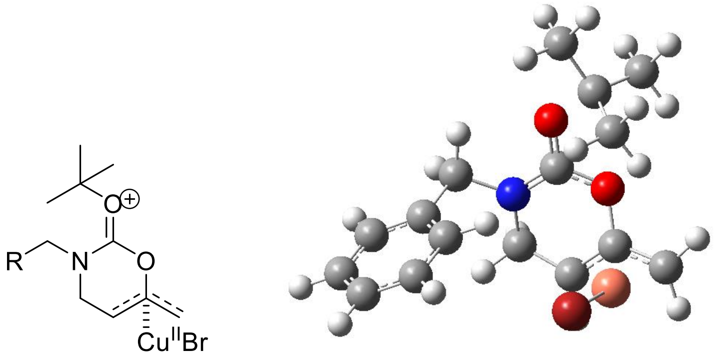

Center Number	Atomic Number	Atomic Type	Coordinates (Angstroms) X	Y	Z
1	6	0	-0.451092	-1.190597	2.374923
2	6	0	0.190246	0.116244	2.626504
3	1	0	-1.542428	-1.082924	2.321817
4	1	0	-0.236632	-1.911540	3.181031
5	1	0	-0.239102	0.812074	3.342495
6	7	0	-0.007408	-1.758260	1.094531
7	6	0	-0.935697	-2.697163	0.432224
8	1	0	-0.407457	-3.134211	-0.419766
9	1	0	-1.144848	-3.505462	1.144432
10	6	0	-2.198488	-1.990383	0.019671
11	8	0	1.924224	-0.512814	1.113082
12	8	0	1.600142	-2.073417	-0.445585
13	6	0	-2.138009	-0.804096	-0.715596
14	6	0	-3.306676	-0.157445	-1.099203
15	6	0	-4.546196	-0.696609	-0.759392
16	6	0	-4.611756	-1.877852	-0.027047
17	6	0	-3.440486	-2.518467	0.369240
18	1	0	-1.173105	-0.370834	-0.989608

19	1	0	-3.243769	0.772288	-1.662721
20	1	0	-5.461364	-0.191044	-1.061774
21	1	0	-5.576974	-2.298620	0.248014
22	1	0	-3.490042	-3.437096	0.953924
23	6	0	1.162656	-1.455075	0.581766
24	29	0	0.553565	2.051467	0.641312
25	35	0	-0.730001	2.906736	-1.067043
26	6	0	1.363055	0.453779	1.959210
27	6	0	2.071263	1.652641	2.011614
28	1	0	3.032951	1.734531	1.509347
29	1	0	1.825495	2.373648	2.790201
30	6	0	2.758120	-1.621909	-1.333622
31	6	0	4.046798	-1.794502	-0.563397
32	1	0	4.126441	-2.812306	-0.163839
33	1	0	4.881232	-1.636971	-1.257869
34	1	0	4.147846	-1.073920	0.253878
35	6	0	2.646790	-2.608869	-2.473960
36	1	0	2.757968	-3.637415	-2.111938
37	1	0	1.682250	-2.510993	-2.985607
38	1	0	3.445813	-2.407603	-3.196819
39	6	0	2.495545	-0.205190	-1.794194
40	1	0	1.486002	-0.106488	-2.214275
41	1	0	2.634016	0.534756	-0.997228
42	1	0	3.212861	0.032444	-2.589384

Zero-point correction= 0.341983 (Hartree/Particle)
Thermal correction to Energy= 0.364550
Thermal correction to Enthalpy= 0.365494
Thermal correction to Gibbs Free Energy= 0.286814
Sum of electronic and zero-point Energies= -1035.817049
Sum of electronic and thermal Energies= -1035.794482
Sum of electronic and thermal Enthalpies= -1035.793537
Sum of electronic and thermal Free Energies= -1035.872217

INT-6

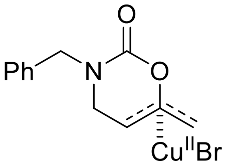

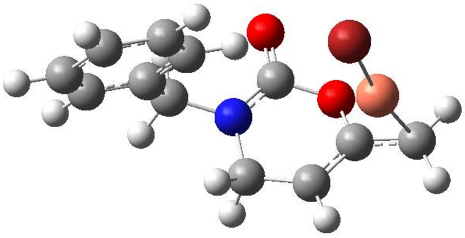

Center Number	Atomic Number	Atomic Type	Coordinates (Angstroms) X	Y	Z
1	6	0	-0.670469	1.917403	-1.436416
2	6	0	0.780586	1.704195	-1.577652
3	1	0	-1.220040	1.080831	-1.895559
4	1	0	-0.991322	2.826869	-1.977708
5	1	0	1.201850	1.378158	-2.525415
6	7	0	-1.063459	2.001789	-0.037690
7	6	0	-2.472251	1.760396	0.247312
8	1	0	-2.653104	2.098570	1.273679
9	1	0	-3.076503	2.397916	-0.414961
10	6	0	-2.858438	0.311902	0.074954
11	8	0	1.107288	2.447253	0.664726
12	8	0	-0.549315	2.496821	2.133996
13	6	0	-1.962076	-0.716074	0.371543
14	6	0	-2.344513	-2.047118	0.238996
15	6	0	-3.632440	-2.365698	-0.183924
16	6	0	-4.532030	-1.345293	-0.482053

17	6	0	-4.143605	-0.014583	-0.359705
18	1	0	-0.947465	-0.480385	0.699002
19	1	0	-1.625732	-2.834187	0.464874
20	1	0	-3.932618	-3.406930	-0.287854
21	1	0	-5.537935	-1.585553	-0.822334
22	1	0	-4.845738	0.783180	-0.604886
23	6	0	-0.234283	2.319690	0.980521
24	29	0	2.221483	-0.198005	-0.168233
25	35	0	1.705352	-2.412377	0.243020
26	6	0	1.630073	1.944164	-0.493645
27	6	0	3.004142	1.674457	-0.481625
28	1	0	3.596297	1.974432	0.380317
29	1	0	3.514213	1.516705	-1.431871

```
---------------------------------------------------------------------
Zero-point correction=                           0.217739 (Hartree/Particle)
Thermal correction to Energy=                    0.234624
Thermal correction to Enthalpy=                  0.235568
Thermal correction to Gibbs Free Energy=         0.167144
Sum of electronic and zero-point Energies=          -878.409018
Sum of electronic and thermal Energies=             -878.392133
Sum of electronic and thermal Enthalpies=           -878.391189
Sum of electronic and thermal Free Energies=        -878.459612
```

Sulfonylation/Self-Coupling of Allenes

Energy profile

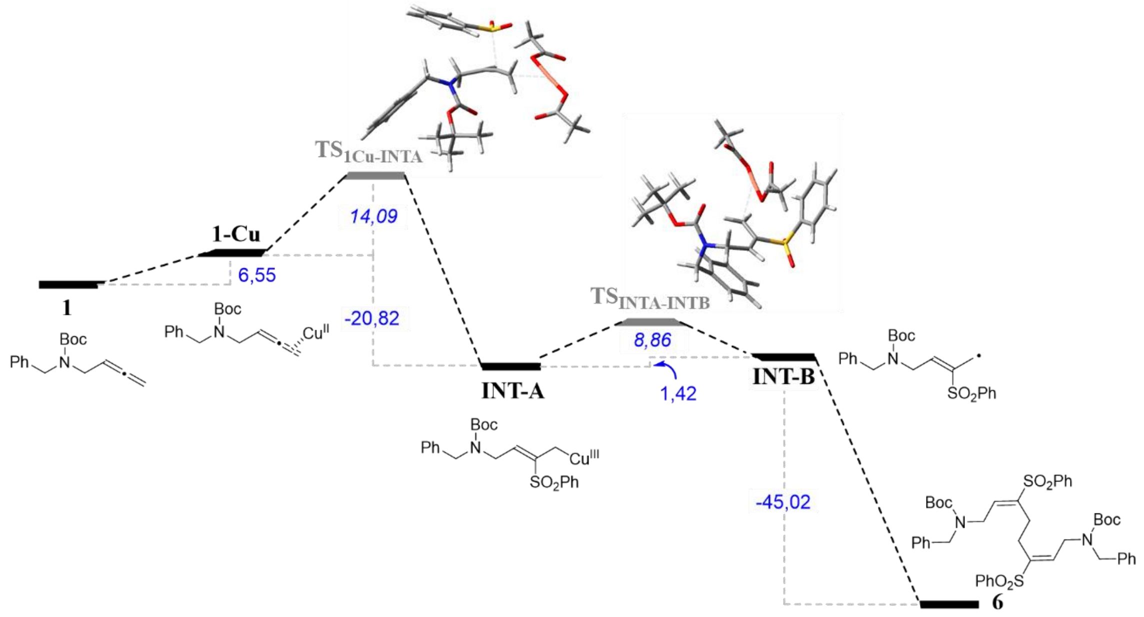

Scheme S4. *Energy profile for the formation of compound* ***6*** *from allene* ***1a*** *(ΔG in kcal mol^{-1}, ΔG_a in italics), calculated at M06/6-31G(d) (C,H,N,O,S), LANL2DZ (Cu) level in MeCN (PCM).*

Atomic coordinates and energies for the stationary points

1

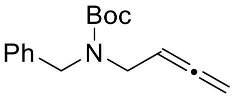

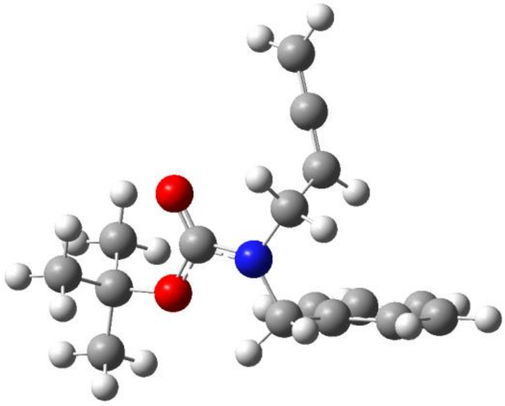

Center Number	Atomic Number	Atomic Type	Coordinates (Angstroms) X	Y	Z
1	6	0	-0.717836	1.771785	-1.324185
2	6	0	-1.439075	2.113305	-0.043027
3	1	0	-0.039859	2.587797	-1.596071
4	1	0	-1.442093	1.647220	-2.141749
5	1	0	-2.210791	1.416909	0.297436
6	7	0	0.046517	0.531097	-1.246315
7	6	0	-0.619175	-0.707300	-1.642274
8	1	0	0.145279	-1.483150	-1.739704
9	6	0	-1.696216	-1.126186	-0.674069
10	1	0	-1.054134	-0.548168	-2.639731
11	8	0	1.753514	1.617802	-0.201716
12	8	0	1.764859	-0.650623	-0.425156
13	6	0	3.028742	-0.850256	0.280445
14	6	0	3.192632	-2.359584	0.244703
15	1	0	3.237630	-2.720807	-0.790600
16	1	0	4.120454	-2.648226	0.754143

17	1	0	2.350991	-2.851952	0.749798
18	6	0	4.162995	-0.174750	-0.470408
19	1	0	4.184135	-0.516225	-1.513743
20	1	0	4.060275	0.914202	-0.454363
21	1	0	5.119660	-0.445514	-0.004537
22	6	0	2.911239	-0.366979	1.715915
23	1	0	2.798121	0.719620	1.766387
24	1	0	2.048810	-0.838476	2.207047
25	1	0	3.814577	-0.653752	2.270144
26	6	0	1.241684	0.577103	-0.582150
27	6	0	-1.171359	3.181308	0.661487
28	6	0	-0.898315	4.254976	1.351521
29	1	0	-0.141378	4.251774	2.136161
30	1	0	-1.415021	5.198036	1.171774
31	6	0	-3.042650	-1.044773	-1.025430
32	6	0	-4.037023	-1.365732	-0.102866
33	6	0	-3.688371	-1.772089	1.181102
34	6	0	-2.343025	-1.863943	1.537947
35	6	0	-1.354190	-1.543633	0.615809
36	1	0	-3.314842	-0.716015	-2.029815
37	1	0	-5.085375	-1.293584	-0.387950
38	1	0	-4.462903	-2.019862	1.904994
39	1	0	-2.066338	-2.187007	2.540354
40	1	0	-0.300149	-1.607439	0.892097

Zero-point correction= 0.338382 (Hartree/Particle)
Thermal correction to Energy= 0.358002
Thermal correction to Enthalpy= 0.358947
Thermal correction to Gibbs Free Energy= 0.289322
Sum of electronic and zero-point Energies= -826.580097
Sum of electronic and thermal Energies= -826.560477
Sum of electronic and thermal Enthalpies= -826.559533
Sum of electronic and thermal Free Energies= -826.629157

$Cu(OAC)_2$

Center Number	Atomic Number	Atomic Type	Coordinates (Angstroms) X	Y	Z
1	29	0	-0.000002	0.000005	-0.000035
2	8	0	1.657288	1.084844	-0.013814
3	6	0	2.323359	-0.000011	-0.015999
4	6	0	3.808909	-0.000017	0.009123
5	8	0	1.657275	-1.084861	-0.013826
6	8	0	-1.657282	-1.084832	0.013810
7	8	0	-1.657283	1.084890	0.013796
8	1	0	4.200739	-0.899405	-0.473840
9	1	0	4.144772	0.000393	1.053585
10	1	0	4.200751	0.899015	-0.474502
11	6	0	-2.323352	0.000030	0.016020
12	6	0	-3.808905	-0.000024	-0.009003
13	1	0	-4.200654	-0.898574	0.475599
14	1	0	-4.144834	-0.001630	-1.053443
15	1	0	-4.200767	0.899846	0.473029

Zero-point correction= 0.102886 (Hartree/Particle)
Thermal correction to Energy= 0.113884
Thermal correction to Enthalpy= 0.114828
Thermal correction to Gibbs Free Energy= 0.063836
Sum of electronic and zero-point Energies= -652.850446
Sum of electronic and thermal Energies= -652.839448
Sum of electronic and thermal Enthalpies= -652.838503
Sum of electronic and thermal Free Energies= -652.889496

1-Cu

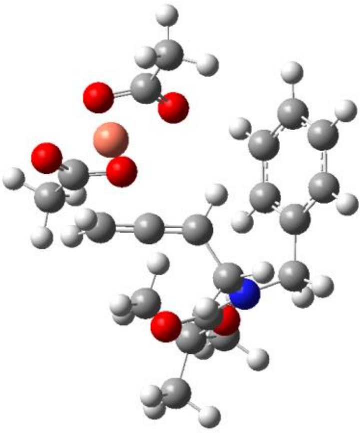

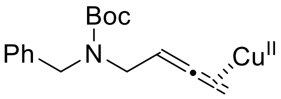

Center Number	Atomic Number	Atomic Type	Coordinates (Angstroms) X	Y	Z
1	6	0	-0.861735	1.328119	-2.358474
2	6	0	0.375191	0.787209	-1.683291
3	1	0	-1.094136	0.735856	-3.249504
4	1	0	-0.698776	2.367815	-2.672931
5	1	0	0.798345	1.361757	-0.854239
6	7	0	-2.018502	1.323779	-1.469547
7	6	0	-2.274658	2.526531	-0.680391
8	1	0	-3.260808	2.420198	-0.220172
9	6	0	-1.213460	2.782724	0.359547
10	1	0	-2.328705	3.376541	-1.375924
11	8	0	-2.301209	-0.906257	-1.831413
12	8	0	-3.492344	0.183050	-0.222209
13	6	0	-4.168774	-1.012369	0.278017
14	6	0	-5.010215	-0.464284	1.416997
15	1	0	-5.720507	0.286568	1.048199
16	1	0	-5.576855	-1.276682	1.888713
17	1	0	-4.372383	0.003168	2.178845
18	6	0	-5.056452	-1.605215	-0.801819
19	1	0	-5.743029	-0.841621	-1.190379
20	1	0	-4.465514	-2.006912	-1.629909
21	1	0	-5.658295	-2.416982	-0.372510
22	6	0	-3.149203	-2.007176	0.807231
23	1	0	-2.537968	-2.421932	0.000164
24	1	0	-2.489116	-1.521515	1.539617
25	1	0	-3.671512	-2.829627	1.313667
26	6	0	-2.588742	0.105378	-1.212116
27	6	0	0.919030	-0.355492	-2.005582
28	6	0	1.461355	-1.504338	-2.330970
29	1	0	1.102952	-2.445626	-1.910001
30	1	0	2.265648	-1.570789	-3.064797
31	6	0	-0.346865	3.867675	0.238680
32	6	0	0.674591	4.066667	1.166041
33	6	0	0.836038	3.176855	2.223173
34	6	0	-0.028015	2.089038	2.352085
35	6	0	-1.047008	1.895630	1.427347
36	1	0	-0.468473	4.559603	-0.596284
37	1	0	1.346476	4.916737	1.059207
38	1	0	1.634832	3.327575	2.947472
39	1	0	0.094880	1.388555	3.176932
40	1	0	-1.721192	1.042295	1.521050
41	29	0	2.462002	-1.151579	0.189766
42	8	0	3.044920	0.760041	0.209149

43	6	0	4.146616	0.392919	-0.311759
44	6	0	5.224262	1.372763	-0.613053
45	8	0	4.297047	-0.849434	-0.540489
46	8	0	2.078898	-3.084568	0.534220
47	8	0	0.773510	-1.483279	1.205708
48	1	0	5.829401	1.030682	-1.457359
49	1	0	5.879591	1.458807	0.262704
50	1	0	4.799409	2.359198	-0.818850
51	6	0	1.018481	-2.729717	1.142938
52	6	0	0.092761	-3.737089	1.727091
53	1	0	0.654144	-4.600662	2.095836
54	1	0	-0.589754	-4.089617	0.943299
55	1	0	-0.502978	-3.295454	2.530885

```
Zero-point correction=                           0.443422 (Hartree/Particle)
Thermal correction to Energy=                    0.474476
Thermal correction to Enthalpy=                  0.475421
Thermal correction to Gibbs Free Energy=         0.379551
Sum of electronic and zero-point Energies=          -1479.444340
Sum of electronic and thermal Energies=             -1479.413285
Sum of electronic and thermal Enthalpies=           -1479.412341
Sum of electronic and thermal Free Energies=        -1479.508210
```

$PhSO_2$·

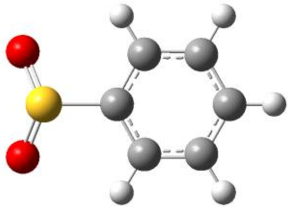

Center Number	Atomic Number	Atomic Type	Coordinates (Angstroms) X	Y	Z
1	6	0	0.095494	-0.000030	0.078866
2	6	0	0.762686	1.221431	0.058500
3	6	0	2.149593	1.210893	-0.033717
4	6	0	2.838120	0.000009	-0.078972
5	6	0	2.149638	-1.210874	-0.033704
6	6	0	0.762699	-1.221435	0.058505
7	1	0	0.206098	2.154965	0.093161
8	1	0	2.693948	2.151868	-0.070593
9	1	0	3.924070	0.000054	-0.146098
10	1	0	2.693961	-2.151864	-0.070524
11	1	0	0.206215	-2.155031	0.093167
12	16	0	-1.690317	0.000003	0.258725
13	8	0	-2.201778	1.283742	-0.270959
14	8	0	-2.201797	-1.283743	-0.270990

```
Zero-point correction=                           0.098344 (Hartree/Particle)
Thermal correction to Energy=                    0.106040
Thermal correction to Enthalpy=                  0.106984
Thermal correction to Gibbs Free Energy=         0.064414
Sum of electronic and zero-point Energies=           -779.816591
Sum of electronic and thermal Energies=              -779.808895
Sum of electronic and thermal Enthalpies=            -779.807950
Sum of electronic and thermal Free Energies=         -779.850520
```

INT-A

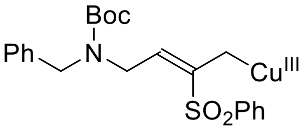

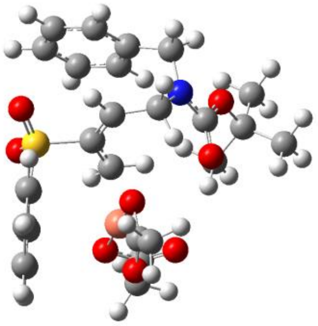

Center Number	Atomic Number	Atomic Type	Coordinates (Angstroms) X	Y	Z
1	6	0	1.007145	0.800243	2.250227
2	6	0	0.032303	1.528008	1.361998
3	1	0	0.582046	-0.187475	2.474486
4	1	0	1.095459	1.354163	3.193824
5	1	0	-0.434866	2.420345	1.783888
6	7	0	2.336212	0.603394	1.704874
7	6	0	3.176455	1.794841	1.566267
8	1	0	4.208219	1.464876	1.422346
9	6	0	2.705993	2.658287	0.428083
10	1	0	3.132384	2.348392	2.515607
11	8	0	1.959592	-1.619106	1.365259
12	8	0	3.929272	-0.653594	0.763943
13	6	0	4.447820	-1.832150	0.057103
14	6	0	5.793505	-1.340878	-0.445161
15	1	0	6.432633	-1.036112	0.393131
16	1	0	6.300995	-2.142789	-0.995178
17	1	0	5.668042	-0.483091	-1.118942
18	6	0	4.629392	-2.986512	1.025385
19	1	0	5.247945	-2.675405	1.877313
20	1	0	3.668877	-3.354830	1.396587
21	1	0	5.145547	-3.808751	0.512745
22	6	0	3.538738	-2.181129	-1.109877
23	1	0	2.573497	-2.583378	-0.783273
24	1	0	3.363087	-1.292955	-1.734510
25	1	0	4.035101	-2.935278	-1.734916
26	6	0	2.700190	-0.649160	1.283544
27	6	0	-0.353016	1.178623	0.104770
28	6	0	0.048435	0.036357	-0.638578
29	1	0	0.859881	-0.588878	-0.255578
30	6	0	2.012582	3.843657	0.667612
31	6	0	1.464115	4.568746	-0.390743
32	6	0	1.604539	4.106324	-1.693225
33	6	0	2.311803	2.927231	-1.941520
34	6	0	2.863812	2.212307	-0.887079
35	1	0	1.888794	4.194998	1.693659
36	1	0	0.912425	5.485458	-0.191541
37	1	0	1.163332	4.659743	-2.520197
38	1	0	2.429246	2.568371	-2.962766
39	1	0	3.407514	1.283908	-1.072708
40	1	0	-0.082631	0.049628	-1.720319
41	6	0	-3.098846	1.312842	-0.402401
42	6	0	-3.464448	0.308558	-1.298438
43	6	0	-4.589297	-0.461416	-1.014938
44	6	0	-5.328225	-0.217177	0.140539
45	6	0	-4.951202	0.794694	1.021845
46	6	0	-3.822567	1.563059	0.759590
47	1	0	-2.887948	0.141430	-2.207119
48	1	0	-4.888755	-1.250171	-1.701977
49	1	0	-6.207863	-0.820350	0.355837

50	1	0	-5.536969	0.984048	1.918809
51	1	0	-3.508845	2.352180	1.440271
52	16	0	-1.597336	2.222990	-0.683317
53	8	0	-1.321696	2.227674	-2.121443
54	8	0	-1.678247	3.480172	0.062182
55	29	0	-1.339462	-1.414545	-0.180875
56	8	0	-2.702565	-2.678344	0.942932
57	6	0	-2.457730	-1.875523	1.873275
58	6	0	-3.070355	-1.989283	3.228728
59	8	0	-1.636438	-0.906525	1.649016
60	8	0	0.173878	-3.370013	-0.924570
61	8	0	-1.223114	-1.988717	-1.969179
62	1	0	-3.525741	-1.033022	3.510959
63	1	0	-2.289163	-2.208268	3.966264
64	1	0	-3.822966	-2.781534	3.250140
65	6	0	-0.400791	-2.997744	-1.950290
66	6	0	-0.202261	-3.664347	-3.285189
67	1	0	0.455879	-4.531390	-3.186714
68	1	0	0.236827	-2.949759	-3.991473
69	1	0	-1.167668	-3.977016	-3.698632

Zero-point correction= 0.546243 (Hartree/Particle)
Thermal correction to Energy= 0.584176
Thermal correction to Enthalpy= 0.585120
Thermal correction to Gibbs Free Energy= 0.475028
Sum of electronic and zero-point Energies= -2259.320690
Sum of electronic and thermal Energies= -2259.282758
Sum of electronic and thermal Enthalpies= -2259.281813
Sum of electronic and thermal Free Energies= -2259.391905

TS$_{1Cu\text{-}INTA}$

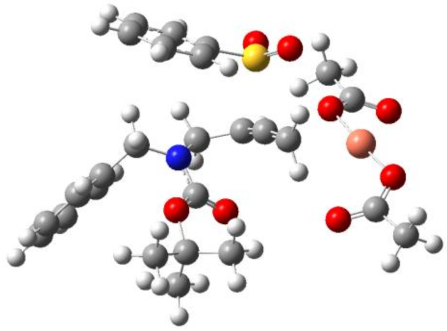

Imaginary frequency = -241.1966

Center Number	Atomic Number	Atomic Type	Coordinates (Angstroms) X	Y	Z
1	6	0	-0.864516	-0.918587	1.247201
2	6	0	0.575187	-0.751471	0.902861
3	1	0	-0.949743	-0.578061	2.294437
4	1	0	-1.124115	-1.988945	1.266956
5	1	0	1.333035	-1.002483	1.653905
6	7	0	-1.760942	-0.219852	0.361363
7	6	0	-3.026309	-0.825486	-0.071648
8	1	0	-2.911819	-1.911205	0.051655
9	6	0	-4.224073	-0.322084	0.692873
10	1	0	-3.157746	-0.641995	-1.145543
11	8	0	-0.692444	1.706950	0.936727
12	8	0	-2.466459	1.712453	-0.497207
13	6	0	-2.566163	3.173062	-0.611378
14	6	0	-3.737383	3.352282	-1.559934
15	1	0	-4.655763	2.937861	-1.123518
16	1	0	-3.895494	4.419792	-1.756950

17	1	0	-3.543443	2.847524	-2.515007
18	6	0	-2.891316	3.764265	0.748558
19	1	0	-3.762319	3.250447	1.180264
20	1	0	-2.048562	3.680588	1.441221
21	1	0	-3.143392	4.826132	0.632150
22	6	0	-1.291993	3.730240	-1.220321
23	1	0	-0.429125	3.600113	-0.559932
24	1	0	-1.083011	3.233699	-2.177579
25	1	0	-1.425365	4.802164	-1.416791
26	6	0	-1.583033	1.146446	0.314712
27	6	0	1.036472	-0.215030	-0.249500
28	6	0	1.510992	0.507717	-1.211724
29	1	0	1.566271	1.600415	-1.052762
30	6	0	-5.282577	0.288029	0.021442
31	6	0	-6.380220	0.779146	0.723169
32	6	0	-6.425603	0.667519	2.110166
33	6	0	-5.373424	0.056660	2.789363
34	6	0	-4.281007	-0.436798	2.083443
35	1	0	-5.236213	0.389913	-1.063791
36	1	0	-7.197605	1.256875	0.185278
37	1	0	-7.279814	1.055323	2.662426
38	1	0	-5.404980	-0.037974	3.873469
39	1	0	-3.464367	-0.919442	2.622430
40	1	0	1.928826	0.081239	-2.123193
41	6	0	-0.198463	-3.032297	-1.250570
42	6	0	-0.953914	-3.973037	-0.549599
43	6	0	-2.167512	-4.370096	-1.099692
44	6	0	-2.602021	-3.820637	-2.304066
45	6	0	-1.832238	-2.872459	-2.978635
46	6	0	-0.613890	-2.463243	-2.454609
47	1	0	-0.587222	-4.400101	0.380890
48	1	0	-2.771166	-5.113118	-0.584914
49	1	0	-3.553989	-4.135960	-2.724892
50	1	0	-2.178739	-2.455174	-3.920566
51	1	0	0.004009	-1.737000	-2.977450
52	16	0	1.367175	-2.556474	-0.593425
53	8	0	1.706588	-3.363046	0.563717
54	8	0	2.310118	-2.278204	-1.663157
55	29	0	3.992114	0.774517	0.238582
56	8	0	5.493945	-0.636568	2.126922
57	6	0	4.430755	-1.231556	1.930514
58	6	0	4.090508	-2.500042	2.679333
59	8	0	3.525013	-0.848162	1.088100
60	8	0	2.465426	3.255443	-0.462264
61	8	0	4.519895	2.353231	-0.614002
62	1	0	3.983814	-3.330787	1.971755
63	1	0	3.121811	-2.393661	3.184361
64	1	0	4.862109	-2.741523	3.415720
65	6	0	3.667003	3.304717	-0.766052
66	6	0	4.246492	4.558772	-1.385206
67	1	0	3.505067	5.362043	-1.418533
68	1	0	4.587744	4.341614	-2.405134
69	1	0	5.124625	4.890287	-0.818878

```
---------------------------------------------------------------------
Zero-point correction=                           0.541981 (Hartree/Particle)
Thermal correction to Energy=                    0.582819
Thermal correction to Enthalpy=                  0.583763
Thermal correction to Gibbs Free Energy=         0.464833
Sum of electronic and zero-point Energies=         -2259.259123
Sum of electronic and thermal Energies=            -2259.218285
Sum of electronic and thermal Enthalpies=          -2259.217341
Sum of electronic and thermal Free Energies=       -2259.336270
```

INT-B

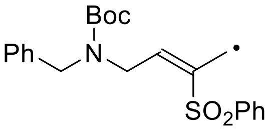

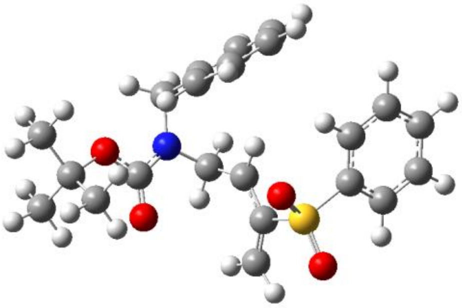

Center Number	Atomic Number	Atomic Type	Coordinates (Angstroms) X	Y	Z
1	6	0	-0.574530	-0.358902	-2.034290
2	6	0	0.386838	-0.590751	-0.913855
3	1	0	-0.821690	-1.289173	-2.556193
4	1	0	-0.132509	0.324180	-2.774999
5	1	0	0.655472	0.288610	-0.325698
6	7	0	-1.807695	0.267038	-1.562216
7	6	0	-1.876410	1.727505	-1.570924
8	1	0	-2.906246	2.013289	-1.340712
9	6	0	-0.903856	2.359354	-0.606241
10	1	0	-1.661691	2.066548	-2.594666
11	8	0	-2.518061	-1.746704	-0.776810
12	8	0	-3.656501	0.169611	-0.297373
13	6	0	-4.672752	-0.476657	0.533676
14	6	0	-5.532643	0.694843	0.973550
15	1	0	-5.973673	1.200977	0.105460
16	1	0	-6.345580	0.340614	1.619468
17	1	0	-4.934143	1.423700	1.536078
18	6	0	-5.486189	-1.452629	-0.297844
19	1	0	-5.894451	-0.948941	-1.183907
20	1	0	-4.881244	-2.305340	-0.619718
21	1	0	-6.328420	-1.824597	0.300207
22	6	0	-4.024011	-1.136423	1.738269
23	1	0	-3.401680	-1.986751	1.445842
24	1	0	-3.403712	-0.411379	2.282888
25	1	0	-4.807061	-1.491603	2.420917
26	6	0	-2.663680	-0.537830	-0.855185
27	6	0	0.925910	-1.819696	-0.551856
28	6	0	0.733261	-3.060872	-1.108796
29	1	0	0.047579	-3.197132	-1.942078
30	6	0	0.217159	3.043730	-1.073696
31	6	0	1.162843	3.548461	-0.183055
32	6	0	0.992859	3.368948	1.186397
33	6	0	-0.129686	2.690629	1.663223
34	6	0	-1.073044	2.192441	0.771987
35	1	0	0.356433	3.172205	-2.148249
36	1	0	2.036934	4.076470	-0.561109
37	1	0	1.734036	3.756640	1.884297
38	1	0	-0.267803	2.550612	2.734060
39	1	0	-1.948249	1.653981	1.140382
40	1	0	1.242594	-3.934406	-0.711860
41	6	0	3.359739	-0.710630	0.350231
42	6	0	4.354226	-1.263930	-0.453238
43	6	0	5.393854	-0.449098	-0.885111
44	6	0	5.427086	0.894786	-0.514880
45	6	0	4.424422	1.433039	0.287800
46	6	0	3.378106	0.628637	0.727294
47	1	0	4.314871	-2.317032	-0.727482
48	1	0	6.182036	-0.863340	-1.510012

49	1	0	6.243994	1.527626	-0.856049
50	1	0	4.455089	2.482436	0.575574
51	1	0	2.584634	1.029897	1.356428
52	16	0	2.015258	-1.744922	0.884498
53	8	0	2.539864	-3.090626	1.136890
54	8	0	1.303101	-1.037073	1.954036

```
Zero-point correction=                           0.439935 (Hartree/Particle)
Thermal correction to Energy=                    0.467603
Thermal correction to Enthalpy=                  0.468547
Thermal correction to Gibbs Free Energy=         0.379396
Sum of electronic and zero-point Energies=          -1606.439608
Sum of electronic and thermal Energies=             -1606.411941
Sum of electronic and thermal Enthalpies=           -1606.410996
Sum of electronic and thermal Free Energies=        -1606.500148
```

TS$_{INTA-INTB}$

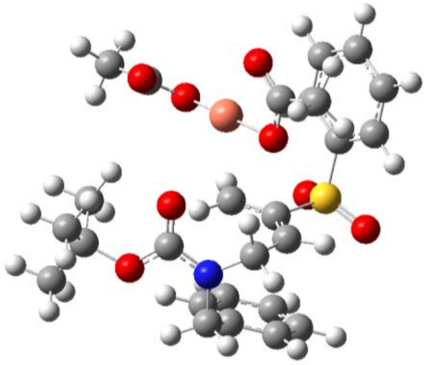

Imaginary frequency = -64.3340

Center Number	Atomic Number	Atomic Type	Coordinates (Angstroms) X	Y	Z
1	6	0	0.974132	1.051219	2.220414
2	6	0	-0.046761	1.700452	1.339476
3	1	0	0.511073	0.104779	2.543429
4	1	0	1.098173	1.673155	3.116685
5	1	0	-0.719028	2.387810	1.857632
6	7	0	2.275478	0.760666	1.666300
7	6	0	3.190381	1.897173	1.509288
8	1	0	4.187417	1.502016	1.303442
9	6	0	2.700926	2.794617	0.409199
10	1	0	3.229999	2.434812	2.467991
11	8	0	1.755114	-1.442076	1.397391
12	8	0	3.777625	-0.619003	0.752192
13	6	0	4.239093	-1.858038	0.111864
14	6	0	5.603949	-1.460829	-0.419242
15	1	0	6.262129	-1.143284	0.399498
16	1	0	6.068983	-2.315221	-0.925842
17	1	0	5.517011	-0.635712	-1.138576
18	6	0	4.365917	-2.962032	1.144333
19	1	0	4.998130	-2.632142	1.979143
20	1	0	3.388666	-3.263965	1.532696
21	1	0	4.842786	-3.835753	0.681406
22	6	0	3.307934	-2.228519	-1.029380
23	1	0	2.328028	-2.557735	-0.666660
24	1	0	3.178482	-1.374771	-1.711405
25	1	0	3.755821	-3.048335	-1.606362
26	6	0	2.558495	-0.527429	1.284489
27	6	0	-0.297767	1.510172	-0.001234

28	6	0	0.324717	0.603366	-0.849832
29	1	0	1.202524	0.045557	-0.542164
30	6	0	1.928029	3.917963	0.698288
31	6	0	1.309123	4.635941	-0.328875
32	6	0	1.452360	4.223661	-1.646370
33	6	0	2.236987	3.106354	-1.944231
34	6	0	2.862233	2.400908	-0.921736
35	1	0	1.805796	4.235082	1.735732
36	1	0	0.700517	5.506298	-0.090159
37	1	0	0.956021	4.767896	-2.446383
38	1	0	2.360088	2.788071	-2.978693
39	1	0	3.467841	1.521558	-1.149675
40	1	0	0.057735	0.547789	-1.903557
41	6	0	-3.068119	1.306841	-0.518497
42	6	0	-3.255720	0.298413	-1.464001
43	6	0	-4.295720	-0.605798	-1.274767
44	6	0	-5.123945	-0.492520	-0.160634
45	6	0	-4.922600	0.521810	0.773109
46	6	0	-3.880673	1.426361	0.603905
47	1	0	-2.612870	0.233803	-2.340478
48	1	0	-4.459308	-1.397554	-2.002134
49	1	0	-5.936644	-1.202755	-0.019198
50	1	0	-5.575563	0.604801	1.638915
51	1	0	-3.702607	2.221771	1.326470
52	16	0	-1.696324	2.417217	-0.703389
53	8	0	-1.426512	2.574778	-2.134057
54	8	0	-1.928919	3.586645	0.146294
55	29	0	-1.123920	-1.711939	-0.124211
56	8	0	-2.737641	-2.810916	1.238307
57	6	0	-2.504976	-1.808386	1.936973
58	6	0	-3.170424	-1.586044	3.262946
59	8	0	-1.643332	-0.929438	1.546662
60	8	0	-0.075143	-4.022683	-0.726882
61	8	0	-0.678312	-2.220206	-1.889537
62	1	0	-3.236037	-0.518934	3.497508
63	1	0	-2.571095	-2.068085	4.047193
64	1	0	-4.166823	-2.037046	3.272688
65	6	0	-0.189597	-3.416779	-1.798478
66	6	0	0.264303	-4.014025	-3.105607
67	1	0	0.549940	-5.061905	-2.977841
68	1	0	1.127737	-3.449356	-3.479977
69	1	0	-0.525137	-3.932465	-3.860410

Zero-point correction= 0.543156 (Hartree/Particle)
Thermal correction to Energy= 0.579259
Thermal correction to Enthalpy= 0.580203
Thermal correction to Gibbs Free Energy= 0.473453
Sum of electronic and zero-point Energies= -2259.308090
Sum of electronic and thermal Energies= -2259.271987
Sum of electronic and thermal Enthalpies= -2259.271043
Sum of electronic and thermal Free Energies= -2259.377793

6

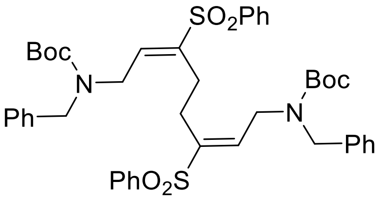

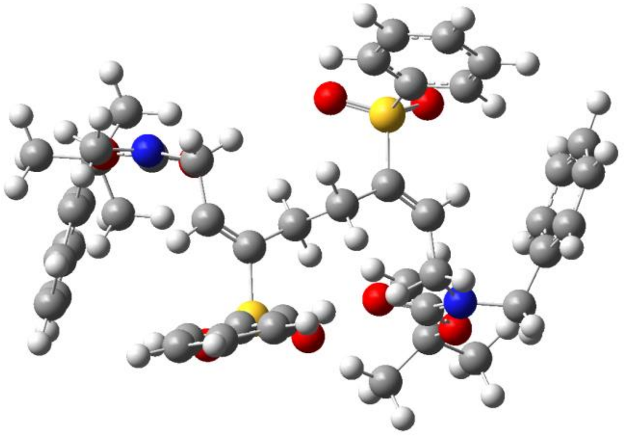

Center Number	Atomic Number	Atomic Type	Coordinates (Angstroms) X	Y	Z
1	6	0	-2.910719	1.106731	-1.296449
2	6	0	-2.767170	0.695103	0.141592
3	1	0	-1.942747	1.123027	-1.810046
4	1	0	-3.333518	2.120155	-1.346346
5	1	0	-3.672607	0.794075	0.746709
6	7	0	-3.819699	0.224237	-2.021215
7	6	0	-5.245547	0.540849	-1.996324
8	1	0	-5.736412	-0.074718	-2.755368
9	6	0	-5.875766	0.333821	-0.641118
10	1	0	-5.360499	1.590157	-2.303984
11	8	0	-2.124797	-1.254955	-2.386345
12	8	0	-4.272230	-1.766059	-2.944758
13	6	0	-3.968680	-3.110361	-3.438919
14	6	0	-5.336676	-3.626984	-3.846996
15	1	0	-5.774733	-2.992677	-4.628028
16	1	0	-5.249436	-4.648634	-4.237065
17	1	0	-6.016999	-3.640465	-2.985112
18	6	0	-3.047190	-3.029087	-4.642803
19	1	0	-3.474674	-2.364174	-5.404759
20	1	0	-2.055773	-2.660484	-4.363869
21	1	0	-2.939450	-4.028253	-5.084746
22	6	0	-3.394594	-3.963275	-2.320878
23	1	0	-2.408676	-3.608531	-2.007621
24	1	0	-4.067905	-3.959083	-1.452692
25	1	0	-3.300772	-4.999786	-2.670316
26	6	0	-3.311332	-0.974678	-2.449168
27	6	0	-1.654978	0.208075	0.695845
28	1	0	-0.470167	-0.225107	-1.008837
29	6	0	-0.318646	0.024674	0.048922
30	1	0	0.650757	1.565190	1.233485
31	1	0	0.222605	-0.816961	0.502828
32	6	0	-6.405326	1.411985	0.066223
33	6	0	-6.947536	1.229342	1.337154
34	6	0	-6.957266	-0.037109	1.913373
35	6	0	-6.424192	-1.120883	1.214325
36	6	0	-5.890930	-0.936432	-0.055884
37	1	0	-6.386822	2.406625	-0.381671
38	1	0	-7.357749	2.080130	1.879063
39	1	0	-7.375803	-0.181667	2.908730
40	1	0	-6.428951	-2.113660	1.661449
41	1	0	-5.475525	-1.783068	-0.605062
42	6	0	-2.574417	1.042583	3.267435
43	6	0	-1.825528	2.146304	3.670150

44	6	0	-2.468212	3.186856	4.329883
45	6	0	-3.838387	3.115645	4.577099
46	6	0	-4.573499	2.006025	4.168594
47	6	0	-3.942323	0.956363	3.508599
48	1	0	-0.754028	2.180753	3.480456
49	1	0	-1.898200	4.053937	4.656456
50	1	0	-4.336300	3.932990	5.095011
51	1	0	-5.642607	1.953335	4.366217
52	1	0	-4.502766	0.081043	3.181891
53	16	0	-1.750904	-0.288478	2.419906
54	8	0	-0.373125	-0.354988	2.926536
55	8	0	-2.602578	-1.479787	2.522439
56	6	0	3.022053	0.154389	1.629161
57	6	0	2.967727	0.547396	0.180351
58	1	0	2.024474	-0.087573	2.016453
59	1	0	3.397849	1.004440	2.218973
60	1	0	3.880493	0.398285	-0.400502
61	7	0	3.914996	-0.967325	1.872014
62	6	0	5.312416	-0.665317	2.189997
63	1	0	5.798814	-1.599048	2.483346
64	6	0	6.019680	-0.013661	1.028773
65	1	0	5.320172	0.002100	3.063589
66	8	0	2.337065	-2.378968	1.037772
67	8	0	4.424045	-3.128526	1.566104
68	6	0	4.190171	-4.509503	1.141945
69	6	0	5.525716	-5.172606	1.428561
70	1	0	5.771002	-5.106794	2.496144
71	1	0	5.485837	-6.231819	1.145746
72	1	0	6.328432	-4.690192	0.855232
73	6	0	3.092345	-5.138236	1.981614
74	1	0	3.326500	-5.039092	3.049693
75	1	0	2.121841	-4.673472	1.785222
76	1	0	3.024225	-6.208422	1.746139
77	6	0	3.886417	-4.556226	-0.345389
78	1	0	2.925375	-4.088671	-0.576866
79	1	0	4.676850	-4.046259	-0.912871
80	1	0	3.855397	-5.603009	-0.674871
81	6	0	3.471917	-2.193495	1.449466
82	6	0	1.898993	1.068370	-0.423600
83	6	0	0.543884	1.289111	0.175254
84	6	0	6.252617	1.360773	1.013975
85	6	0	6.777881	1.978284	-0.119997
86	6	0	7.075587	1.220938	-1.249053
87	6	0	6.865450	-0.158106	-1.234779
88	6	0	6.342598	-0.770553	-0.101881
89	1	0	6.004993	1.956456	1.894046
90	1	0	6.950118	3.053701	-0.121925
91	1	0	7.478401	1.701845	-2.138717
92	1	0	7.107220	-0.755116	-2.112479
93	1	0	6.160380	-1.846801	-0.091000
94	1	0	0.032258	2.133367	-0.311714
95	6	0	2.184652	3.262378	-2.120551
96	6	0	1.083416	4.034845	-2.474622
97	6	0	1.198905	5.421583	-2.436864
98	6	0	2.398319	6.010051	-2.045790
99	6	0	3.492792	5.221865	-1.690387
100	6	0	3.391778	3.837141	-1.724870
101	1	0	0.158280	3.551630	-2.783761
102	1	0	0.350015	6.042422	-2.714959
103	1	0	2.483253	7.094564	-2.016991
104	1	0	4.427493	5.689099	-1.387754
105	1	0	4.238114	3.205554	-1.452974
106	16	0	2.062174	1.487141	-2.146160
107	8	0	0.808507	1.129788	-2.823846

```
   108          8           0        3.330132    0.954977   -2.656814
 ---------------------------------------------------------------------
 Zero-point correction=                           0.890265 (Hartree/Particle)
 Thermal correction to Energy=                    0.944789
 Thermal correction to Enthalpy=                  0.945733
 Thermal correction to Gibbs Free Energy=         0.795308
 Sum of electronic and zero-point Energies=          -3212.977075
 Sum of electronic and thermal Energies=             -3212.922552
 Sum of electronic and thermal Enthalpies=           -3212.921607
 Sum of electronic and thermal Free Energies=        -3213.072032
```

Scan of the potential energy surface from 6 separating C-C bond

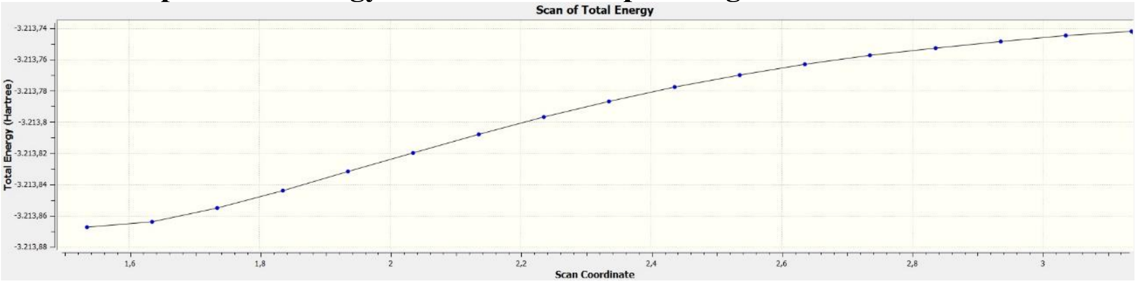

Initial point (H omitted)

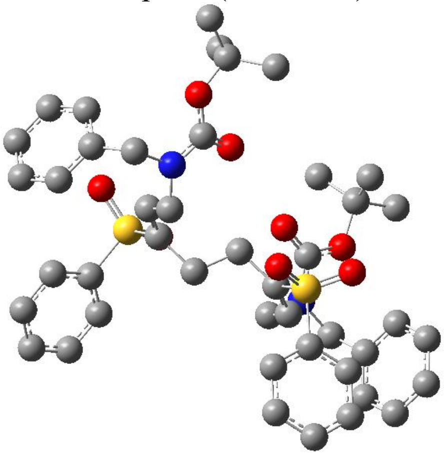

Final point (H omitted)

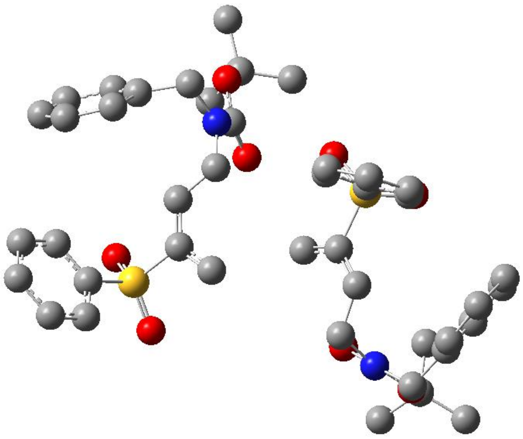

Supplement: Supplementary file 1 [file ol5c01990_si_001.pdf]
